# Aidi injection inhibits the migration and invasion of gefitinib-resistant lung adenocarcinoma cells by regulating the PLAT/FAK/AKT pathway

**DOI:** 10.1186/s13020-024-01054-1

**Published:** 2025-01-03

**Authors:** Jingyuan Zhang, Siyun Yang, Xiaodong Chen, Fanqin Zhang, Siyu Guo, Chao Wu, Tieshan Wang, Haojia Wang, Shan Lu, Chuanqi Qiao, Xiaoguang Sheng, Shuqi Liu, Xiaomeng Zhang, Hua Luo, Qinglin Li, Jiarui Wu

**Affiliations:** 1 Department of Clinical Chinese Pharmacy, School of Chinese Materia Medica, Beijing University of Chinese Medicine, Beijing, 102488 China; 2 Institute of Chinese Materia Medica, China Academy of Chinese Medical Sciences, Beijing, 100700 China; 3 Beijing Research Institute of Chinese Medicine, Beijing University of Chinese Medicine, Beijing, 102488 China; 4 Zhejiang Cancer Hospital, Hangzhou Institute of Medicine (HIM), Chinese Academy of Sciences, Hangzhou, 310022 Zhejiang China; 5 Macau Centre for Research and Development in Chinese Medicine, State Key Laboratory of Quality Research in Chinese Medicine, Institute of Chinese Medical Sciences, University of Macau, Macao, People’s Republic of China

**Keywords:** Non-small cell lung cancer, Aidi injection, Gefitinib resistance, PLAT/FAK/AKT pathway

## Abstract

**Background:**

With extended gefitinib treatment, the therapeutic effect in some non-small cell lung cancer (NSCLC) patients declined with the development of drug resistance. Aidi injection (ADI) is utilized in various cancers as a traditional Chinese medicine prescription. This study explores the molecular mechanism by which ADI, when combined with gefitinib, attenuates gefitinib resistance in PC9GR NSCLC cells.

**Methods:**

In vitro and in vivo pharmacological experiments were conducted in PC9GR cells and NSG mice with PC9GR cell-derived tumors, respectively. The molecular mechanism of ADI was further studied using whole-transcriptome sequencing technology. Bioinformatics and molecular biology methods were employed to validate the critical targets of ADI.

**Results:**

Firstly, ADI treatment alone and combined with gefitinib significantly inhibited the proliferation, migration, and invasion of PC9GR cells. Then, whole-transcriptome sequencing and bioinformatics analysis revealed that PLAT is a key target for the increased efficacy of ADI combined with gefitinib. Additionally, ADI downregulates the expression of PLAT, TNC, ITGB3, p-AKT, p-PI3K, and p-FAK. ADI inhibits the migration and invasion of PC9GR cells by regulating the PLAT/FAK/AKT pathway.

**Conclusions:**

Aidi injection inhibits the migration and invasion of gefitinib-resistant lung adenocarcinoma cells by regulating the PLAT/FAK/AKT pathway. This study provides essential evidence for elucidating the mechanism of ADI in synergistic therapy for lung cancer.

**Supplementary Information:**

The online version contains supplementary material available at 10.1186/s13020-024-01054-1.

## Introduction

Lung cancer is a clinical malignant tumor with high incidence and mortality [1]. Among lung cancer, non-small cell lung cancer (NSCLC) accounts for up to 80% to 85% of the diagnoses. Due to its asymptomatic nature and the lack of specific biomarkers, most lung cancer patients are diagnosed when at late stages, resulting in limited treatment options and a grim prognosis [2–5]. Exosomes offer a possibility for early diagnosis and treatment of cancer [6]. The selectivity of nanomaterials also provides an innovative approach to cancer therapy [7]. However, radiation therapy and chemotherapy remain the primary treatment methods for patients with advanced NSCLC. These treatments often have severe adverse effects and relatively limited effectiveness, meaning many patients do not benefit from them [8, 9]. Approximately 30% of NSCLC patients experience mutations in the epidermal growth factor receptor (EGFR) [10, 11]. For patients with EGFR mutations, molecularly targeted drugs such as tyrosine kinase inhibitors (TKIs) have become a new treatment strategy following surgery and radiotherapy, particularly for patients with advanced NSCLC [12, 13]. Gefitinib, a representative first-generation EGFR-TKI, inhibits tumor growth and metastasis by suppressing EGFR activity, thereby promoting tumor cell apoptosis. However, most patients develop drug resistance approximately 10–12 months after treatment with gefitinib, limiting its clinical use [14–16]. Exploring how to prolong the efficacy of TKIs has become a crucial issue for patients with EGFR-driven mutations in NSCLC. Some studies have shown that the mTOR/PI3K/AKT signaling pathway is involved in the synergistic inhibition of lenalidomide and gefitinib on lung adenocarcinoma (LUAD) progression and the reduction of gefitinib resistance [17]. The reciprocal positive interaction between the PI3K/AKT signaling pathway and Cx26 is involved in gefitinib resistance in NSCLC cells [18]. Inhibition of the PI3K/AKT signaling pathways effectively addresses the resistance observed in NSCLC cell lines to gefitinib [19]. The suppression of Yangyin Jiedu on the PI3K/AKT signaling pathway alleviates gefitinib resistance in NSCLC [20]. Trans-3,5,4´-trimethoxystilbene (TMS) also reduces NSCLC resistance to gefitinib by upregulating miR-345 and miR-498, which inhibits the MAPK/AKT/Bcl-2 pathway [21].

Combining Traditional Chinese Medicine (TCM) with Western Medicine provides a new approach to overcoming drug resistance to targeted therapies [22]. Aidi injection (ADI) is a TCM injection that clears heat and detoxifies, dissipates blood stasis and resolves nodules. It is primarily utilized for the treatment of various cancers. Its main components include Mylabris, Astragalus membranaceus (Fisch.) Bunge, Panax ginseng C. A. Mey., and Radix Acanthopanacis Senticosl [23]. Many studies have reported that Aidi injection has adjuvant therapeutic effect in various tumors, such as esophageal cancer, breast cancer, lung cancer, and pancreatic cancer [24–27]. The reported chemical components in ADI were 16, and all 23 compounds from experiments and databases were identified [27]. Our previous study has identified key pharmacological components and targets for Aidi injection in the treatment of pancreatic cancer. Including Cantharidin, Calycosin-7-glucoside, Chlorogenic acid, Sofraxidin, Formononetin, Astragaloside IV, and Astragaloside I, the compounds in Aidi injection were identified by UPLC-MS/MS [27]. For non-small cell lung cancer, clinical randomized controlled trials (RCTs) demonstrated that the combination of ADI and gefitinib enhanced treatment effectiveness and reduced toxicity. Previous studies have investigated the consequences of ADI in conjunction with gefitinib on the growth and apoptosis of NSCLC drug-resistant cell lines, exploring its role in reversing EGFR-TKI resistance [28, 29]. These findings suggest that ADI, in conjunction with gefitinib, may enhance the killing effect of gefitinib on tumor cells and increase drug sensitivity. However, the mechanisms of Traditional Chinese Medicine in combination therapy are complex. The mechanism of ADI increases NSCLC sensitivity to gefitinib has not been elucidated, so further systematic investigation is needed.

Building on previous research, this research aims to explore the effects of ADI on gefitinib-resistant NSCLC. Pharmacodynamic evaluations of gefitinib-resistant cells were conducted both in vitro and in vivo. Additionally, the mechanism underlying the sensitization of gefitinib-resistant NSCLC by ADI was investigated, further elucidating the role of TCM in enhancing therapeutic effects and reducing toxicity in combination therapy to provide a solid theoretical basis for its clinical use.

## Materials and methods

### Reagents

The Aidi injection (batch number 20220312) was provided free of charge by Guizhou Yibai Pharmaceutical Co., Ltd. Gefitinib (ZD1839) was purchased from AbMole with a concentration of 10 mM*1 mL (in DMSO). After the drug solutions were aliquoted, they were stored in a – 20 ℃ freezer and thawed at 4 ℃ as needed, avoiding repeated freeze–thaw cycles.

### Cell culture and treatment

Human lung cancer cells (PC9 cells) verified as authentic through STR identification were obtained from Zhejiang Meisen Cell Technology Co., Ltd. PC9GR cells were obtained from Shanghai Fuheng Biotechnology Co., Ltd. It is an acquired gefitinib-resistant cell line derived from the parental cell line PC9 through induced mutation. The parental cell line was authenticated through STR profiling. PC9 cells were cultured in RPMI 1640 containing 10% FBS and 1% Anti-Anti in a 37 ℃ incubator with 5% CO_2_. PC9GR cells were cultured in DMEM with 1 μg/mL gefitinib, 10% FBS and 1% Anti-Anti in a 37 ℃ incubator with 5% CO_2_. Transfected cells were obtained by transfecting a knockdown vector or a mixture of siRNAs for PLAT into cells.

### Cell proliferation experiment

Cell Counting Kit-8 (CCK-8) was purchased from Biorigin, and the BeyoClick^™^ EdU Cell Proliferation Kit was obtained from Beyotime. In brief, cells were cultured in 6-well plates at a density of 500 cells for each well in a 37 ℃ incubator with 5% CO_2_. The cells were cultured in different concentrations of ADI, with the medium being changed every three days. On day 15, the cells were fixed with 4% paraformaldehyde and stained with 0.1% crystal violet. The experimental results were investigated using an inverted microscope.

### Cell migration and invasion assay

Cells were planted in a 6-well plate at a density of 8 × 10^5^ cells for each well. After the cells reached confluence, a single scratch was made across the cell monolayer using a pipette tip. Subsequently, the cells were cultured in a medium without FBS and containing different drug concentrations. Photographs were taken at 0, 6, 12, 24, and 48 h after wounding. In the transwell assay, 1 × 10^5^ cells suspension was added into the upper chamber of a 24-well transwell plate with an 8 μm pore filter (Corning Inc., USA). The medium containing 10% FBS was added to the lower chamber. After 24 h, the cells were fixed with 4% paraformaldehyde and stained with 0.5% crystal violet. The experimental results were observed under an inverted microscope.

### Cell cycle analysis

Cell cycle changes were detected through Cell cycle kits (Beyotime Biotechnology Co., Ltd., China) and flow cytometry. In brief, cells were inoculated into 6-well plates at a density of 4 × 10^5^ cells for each well. After 24 h of treatment with different drugs, the cells were collected following trypsin digestion, centrifugation and resuspension. Then, precooled absolute ethanol was added for cell fixation overnight. Following removing the supernatant and washing the cells with PBS, the cell precipitate was collected again. Then, cells were incubated with the mixture of dyeing buffer: PI (20 X): RNA sea (50 X) = 50:2.5:1 in the dark at 4 ℃ for 30 min. Finally, the cell suspension was filtered and used for periodic detection.

### Animal models

Fifty male NSG mice (approximately 20 g, 5–8 weeks) were obtained from Beijing Weishanglide Biotechnology Co., Ltd. All procedures were in line with the animal ethics review guidelines of Beijing Weishang Lide Biotechnology Co., Ltd. (No.VS2126A00068). PC9GR cells were resuspended in DMEM without FBS at a density of 1 × 10^8^ cells/mL to establish a drug-resistant NSCLC xenograft model. 0.1 mL of the suspension was subsequently inoculated subcutaneously into the right axillary region of NSG mice. Vernier calipers were used to measure the length (a) and width (b) of the tumors. The tumor volume (V) was calculated using the formula V (mm^3^) = πab^2^/6. Once the tumor volume exceeded 100–150 mm^3^, a large mung bean lump was observed under the skin of the mice, which showed that the NSCLC drug-resistant tumor transplantation model was successfully constructed in NSG mice. Then, the mice were randomly grouped, and treatments were administered for 21 days. The mice received gavage and intraperitoneal injections of the drug every three days. During drug administration, the behavior of the mice was observed every day. Every three days, both the body weights and tumor diameters were assessed.

### HE staining and immunohistochemical staining

The slices were first dewaxed, hydrated, and washed in PBS for all staining techniques. Using standard protocols, tissues from the heart, liver, spleen, lung, kidney and tumors were stained with H&E. For immunohistochemistry, the sections were subjected to treatment with specific primary antibodies (against Ki67 and CD34) and incubated with 3% hydrogen peroxide. The slices were then stained with DAB chromogenic solution, treated with the corresponding secondary antibody DAB for two hours, and counterstained with hematoxylin. The in-situ cell death detection kit POD (Roche, Switzerland) was used for TUNEL staining. Finally, the sections were examined by confocal laser scanning microscopy (Nikon, Japan). The following antibodies were used: t-PA polyclonal antibody (1:300, Proteintech, 10147-1-AP, China), integrin beta 3 antibody (1:100, Affinity, AF6086, China), and anti-tenascin-C (200 μg/ml for IHC, Santa Cruz, Sc-25328, USA).

### Whole-transcriptome sequencing

After the total RNA was extracted, the integrity and total amount of RNA were evaluated via 1% agarose gel electrophoresis, NanoPhotometer and Agilent 2100 bioanalyzer. Six mRNA, lncRNA, and miRNA libraries were constructed using the Illumina TruSeqTM Small RNA Sample Prep Kit (New England Biolabs, E7530). The Illumina TruSeq Small RNA Kit formed six miRNA sequencing libraries for library construction. The resulting libraries were then sequenced using the Illumina NovaSeq 6000 SBS Kit v3-HS.

### Bioinformatics analysis

The limma package in R software was used to analyze the genetic differences between different comparison and control groups. The STRING database (https://cn.string-db.org/) was utilized to identify protein–protein interactions (PPIs). The PPI network was constructed using Cytoscape software, and topological analysis was performed on the network. Key modules within the network were examined using the cytoHubba and MCODE plug-ins. A hypergeometric distribution algorithm was used to calculate the significance of the enrichment of different genes or proteins at the entrances of the Gene Ontology (GO, http://geneontology.org/) and (KEGG, https://www.kegg.jp/) pathways.

The UCSC Xena (http://xena.ucsc.edu/) database provided the normalized gene expression matrix and clinical information data for various tumor and normal tissue samples. The relationship between PLAT expression and overall survival (OS) in LUAD patients was assessed via univariate analysis in the TCGA and Kaplan–Meier plotter (KM Plotter, http://www.kmplot.com/analysis/) databases. The OS analysis results for PLAT in patients with various cancers were obtained from the GEPIA2 database. The receiver operating characteristic (ROC) curve was constructed to predict the prognosis of LUAD patients with high/low expression of PLAT. Spearman's correlation analysis evaluated the genes related to PLAT in lung cancer and the differential genes between the high and low PLAT expression groups. Gene set enrichment analysis (GSEA) was performed in two groups using the database of hallmark gene sets from the MSigDB (https://www.gsea-msigdb.org/gsea/msigdb).

### Real-time quantitative PCR (RT‒qPCR) analysis

Under the instructions provided by the manufacturer, total RNA was obtained from cells using the RNA Easy Fast Cell Kit (Tiangen, China). A SpectraMax Quick Drop reader (United States) measured the quality of the total RNA, and 1 μg of total RNA was utilized for cDNA synthesis following the guidelines provided by the ReverTra Ace qPCR RT Kit (Toyobo, Japan). RT-qPCR was performed using SYBR Green Real-time PCR Master Mix (Toyobo, Japan). GAPDH and β-actin were used as controls. Primers from Guangzhou RiboBio Co., Ltd. (Guangzhou, China) sequences were used as follows:

β-Actin-Forward 5’-CCTGGCACCCAGCACAAT-3’; β-Actin-Reverse 5’-GGGCCGGACTCGTCATAC-3’. PLAT-Forward 5’-GGTCTGGAGAAGTCTGTAGAG-3’; PLAT-Reverse 5’-CCTAGACTGGATTCGTGACAA-3’. TNC-Forward 5’-GACAAGGACACAGATTCAGCCATC-3’; TNC-Reverse 5’-CAGGTTGACACGGTGACAGTTC-3’. ITGB3-Forward 5’-CCTTCACCAATATCACGTACCG-3’; ITGB3-Reverse 5’-CTCCCCACAAATACTGTCCTC-3’. FAK-Forward: 5’-CAGGGTCCGATTGGAAACCA-3’; FAK-Reverse 5’-CTGAAGCTTGACACCCTCGT-3’. AKT-Forward 5’-TACGAGATGATGTGCGGTCG-3’; AKT-Reverse 5’-CAGCCCTGAAAGCAAGGACT-3’. PI3K-Forward 5’-TGTTACTCAAGAAGCAGAAAGGGAAG-3’; PI3K-Reverse 5’-ACGGTTGCCTACTGGTTCAATTAC-3’.

### Western blot

Cells were lysed in RIPA and subsequently centrifuged for 20 min (12,000 rpm, 4 °C). The protein concentration was measured using the BCA protein assay kit (Solarbio, China). After combining total protein (10 μg) with 5 × sample buffer, it was boiled at 99 °C for 5 min before transferring onto 10% SDS-PAGE gels. Samples were electrotransferred from the gels to the NC membranes. After 2 h of blocking with 5% skimmed milk at room temperature, the NC membranes were incubated with antibody at 4 °C overnight. Then, the membranes were treated with the secondary antibody (rabbit, Proteintech, China). ImageJ software analyzed the protein band intensities and normalized them against the corresponding GAPDH. The primary antibodies were as follows: t-PA polyclonal antibodies (1:800, Proteintech), CD61/integrin beta 3 polyclonal antibody (1:2500, Proteintech), TNC/Tenascin-C polyclonal antibodies (1:800, Proteintech), Vimentin polyclonal antibodies (1:800, Proteintech), pan-AKT/1/2/3 antibody (1:1000, Affinity), phospho-pan-AKT1/2/3 (Thr308) antibody (1:1000, Affinity), PI3K p85 alpha antibody (1:1000, Affinity), phospho-PI3K p85 alpha (Tyr607) antibody (1:1000, Affinity), FAK antibody (1:1000, Affinity), phospho-FAK (Tyr397) antibody (1:1000, Affinity), E-cadherin antibody (1:1000, Affinity), N-cadherin antibody (1:1000, Affinity), GAPDH polyclonal antibodies (1:20000, Proteintech).

### Coimmunoprecipitation

After the cells were transfected with the specified constructs for 48 h, they were lysed in IP lysis buffer for one hour at 4 °C. Following centrifugation for 20 min, the cleared supernatant was rotated with antibodies and protein A beads (Invitrogen) for four hours at 4 °C. Then, the beads were washed four times with IP lysis buffer and eluted in 1X SDS buffer. The primary antibodies utilized included anti-PLAT (Proteintech), anti-ITGB3 (Cell Signaling Technology), anti-TNC (Santa Cruz), and anti-IgG (Abcam).

### Statistical analysis

The statistical processing software GraphPad Prism 7.0 statistically analyzed all the collected and recorded data. Unless otherwise specified, the data's average value and dispersion degree are represented as Mean ± SD. Unpaired Student's t-test was used to identify the differences between the two groups. One-way analysis of variance (ANOVA) and Tukey's honesty (Tukey's HSD) were used to identify the differences among multiple groups. *P* < 0.05 was considered statistically significant.

## Results

### Aidi injection inhibits the malignant biological behavior of PC9 and PC9GR cells

**Fig. 1:**
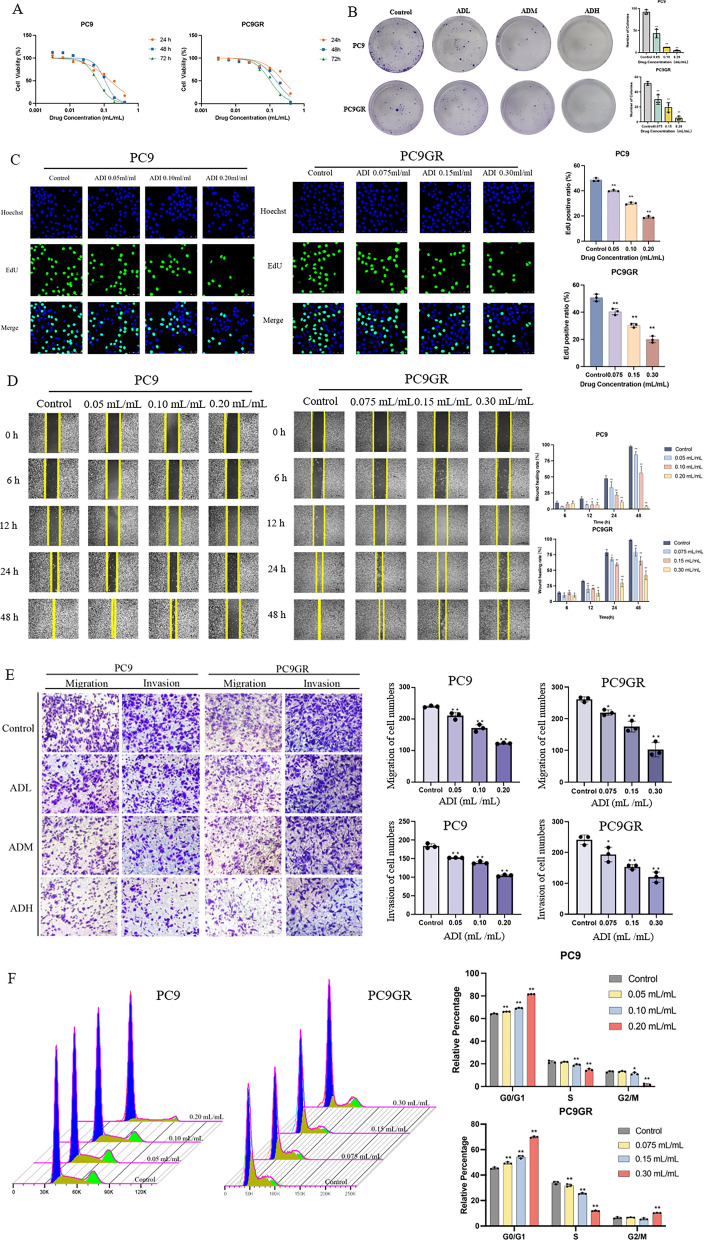
Aidi injection inhibits the malignant biological behavior of PC9 and PC9GR cells. **A** The inhibition of cell viability on PC9 and PC9GR cells by ADI; **B** the effect of ADI on the formation of PC9 and PC9GR cell colonies; **C** effect of ADI on the positive rate of EDU cells in PC9 and PC9GR cells (50 μm); **D** effect of ADI on the wound healing of PC9 and PC9GR cells; **E** effect of ADI on the migration and invasion of PC9 and PC9GR cells (100 μm); **F** three-dimensional diagram of the effect of ADI on PC9 and PC9GR cells cycle distribution and effect of ADI on the cell cycle of PC9 and PC9GR cells. ^*^P < 0.05; ^**^P < 0.01

In the CCK-8 cell proliferation inhibition experiment, ADI injection significantly inhibits proliferation in PC9 and PC9GR cells (Fig. 1A). The effect of inhibition increased with increasing drug dose and time (Fig. 1A). The values for half-maximal inhibitory concentration (IC_50_) of ADI in PC9 cells at 24, 48 and 72 h are 0.15 ± 0.006 mL/mL, 0.11 ± 0.004 mL/mL and 0.07 ± 0.004 mL/mL, respectively. The corresponding IC_50_ values of PC9GR cells are 0.24 ± 0.005 mL/mL, 0.15 ± 0.007 mL/mL and 0.10 ± 0.008 mL/mL. In plate colony formation assay, ADI alone inhibits the colony formation ability of PC9 and PC9GR cells. The inhibitory effect increased with increasing drug concentration (Fig. 1B). Based on the EdU proliferation assay results, ADI alone decreases the proportion of EdU-positive PC9 and PC9GR cells with the extent of reduction was proportional to the dose of the drug (Fig. 1C). In the wound healing experiment, when the concentration of ADI increased, the healing speed of the scratch significantly decreased (Fig. 1D). According to the Transwell experiments, ADI significantly inhibited migration and invasion of PC9 and PC9GR cells (Fig. 1E). The scratch assays and transwell proves that ADI effectively inhibits cell migration and invasion in vitro. The flow cytometry results indicated that ADI elevated the proportion of lung cancer cells in the G0/G1 phase, indicating cell cycle arrest (Fig. 1F).

### Aidi injection combined with gefitinib inhibited the malignant biological behavior of PC9GR cells

**Fig. 2:**
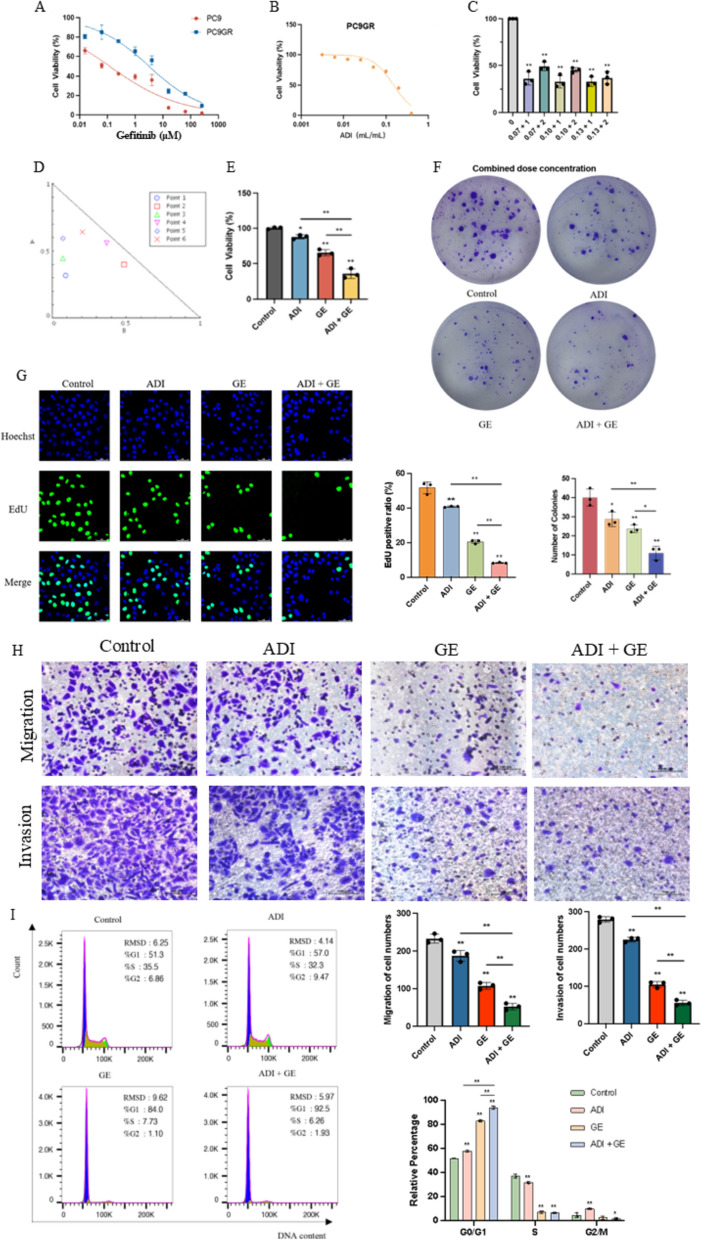
The combination of ADI and gefitinib inhibited the malignant biological behavior of PC9GR cells. **A** The effect of gefitinib on the viability of PC9 and PC9GR cells after 48 h; **B** the effect of ADI on the viability of PC9GR cells after 48 h; **C** the effect of ADI combined with gefitinib on the viability of PC9GR cells after 48 h; **D** CompuSyn software was used to analyze the equivalent effect of combined ADI and gefitinib at different concentrations; **E** effect of ADI in conjunction with gefitinib (the best combination regimen) on the viability of PC9GR cells; **F** effect of ADI in conjunction with gefitinib on the colony formation of PC9GR cells; **G** effect of ADI in conjunction with gefitinib on the positive rate of EDU cells in PC9GR cells (50 μm); **H** effect of ADI in conjunction with gefitinib on the migration and invasion of PC9GR cells (100 μm); **I** effect of ADI in conjunction with gefitinib on the cell cycle regulation of PC9GR and histogram of cell cycle regulation of PC9GR cells by ADI in conjunction with gefitinib. ^*^P < 0.05; ^**^P < 0.01

Gefitinib inhibits the proliferation of PC9GR cells, and its 48-h IC_50_ value is 3.14 ± 0.13 μM, which is 26 greater than PC9 cells (0.12 ± 0.04 μM) (Fig. 2A). When PC9GR cells are injected with ADI, the 48-h IC_50_ value is 0.15 ± 0.007 mL/mL (Fig. 2B). Analyzed using CompuSyn software, at specific concentrations the combination of Aidi and gefitinib had the most substantial synergistic effect (Fig. 2C, D). Specifically, when the concentration of ADI is 0.07 mL/mL and the concentration of gefitinib is 1 μM, the combination index (CI) is the lowest, indicating the combination had the most significant effect (Fig. 2E). Further horizontal comparison experiments revealed that ADI combined administration of gefitinib could reduce the concentration of gefitinib compared with that of gefitinib alone, thereby increasing the sensitivity of lung cancer resistant cells to gefitinib. Both the ADI monotherapy group and the gefitinib monotherapy group inhibit the colony formation ability of the cells. However, the inhibitory effect of combined administration is significantly greater (Fig. 2F). The proportion of EdU-positive cells was decreased in both the ADI monotherapy group and the gefitinib monotherapy group. However, the reduction effect in the combined treatment group is significantly greater (Fig. 2G). ADI monotherapy, gefitinib monotherapy, and combination therapy effectively inhibited cell migration and invasion. However, the effect of the combination was significantly greater than either of the two drugs administered alone (Fig. 2G). The inhibition of the migration and invasion was enhanced when ADI was combined with gefitinib, indicating that this combination enabled the use of lower concentrations of gefitinib to effectively inhibit the migration and invasion of drug-resistant lung cancer cells (Fig. 2H). The proportions of cells in the G0/G1 phase were effectively increased in the ADI monotherapy group, gefitinib monotherapy group and combined treatment group, indicating that cell cycle arrest occurred (Fig. 2I). Notably, the effect of the combination treatment was significantly more substantial than either of the drugs administered alone. In summary, the combination of ADI and gefitinib synergistically inhibits cell growth, migration and invasion, and its inhibitory effect is more remarkable than gefitinib administered alone. This finding provides a novel potential strategy for treating drug-resistant lung cancer.

### Aidi injection combined with gefitinib inhibited the resistance of transplanted lung tumors in mice

**Fig. 3:**
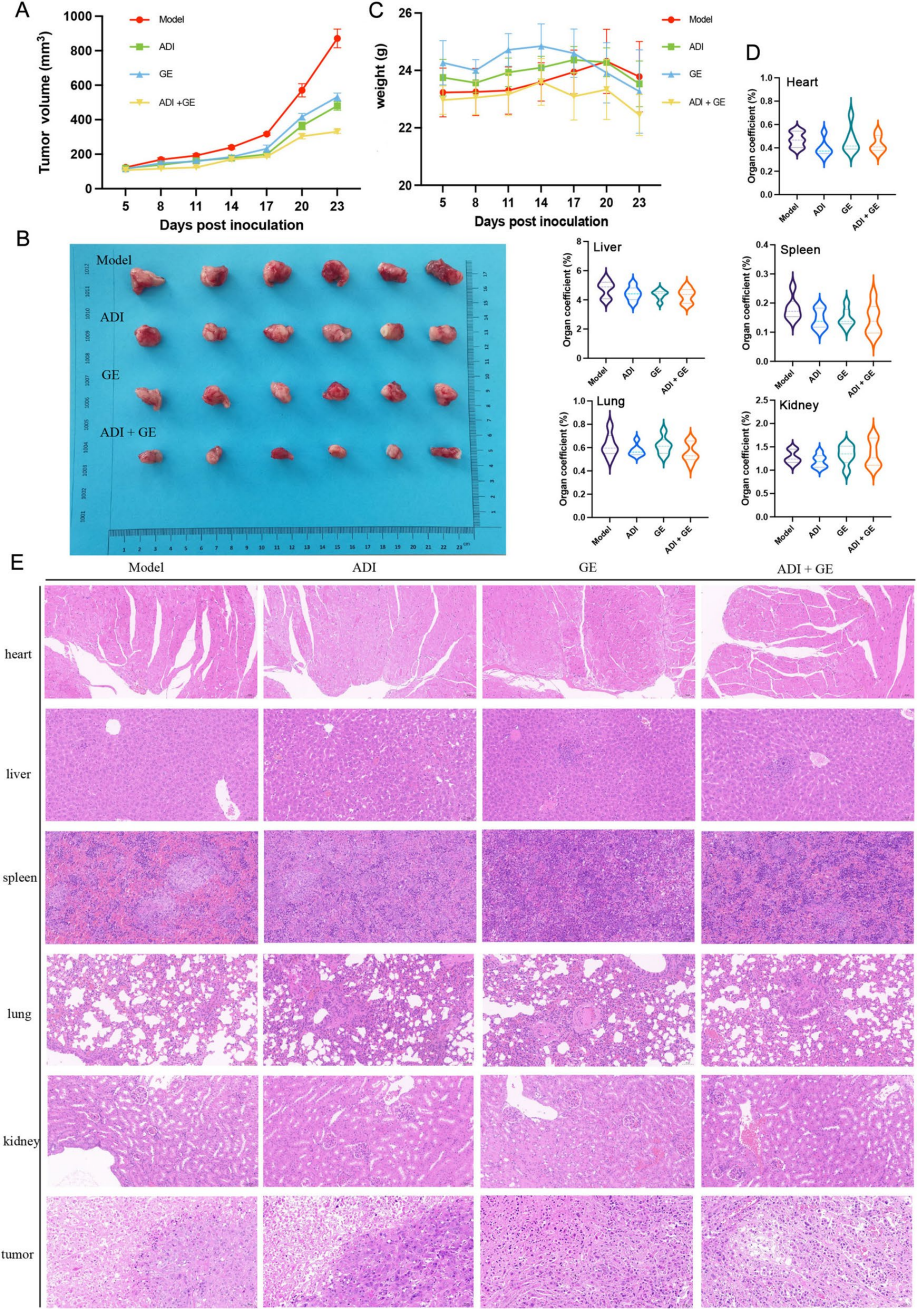
Aidi injection combined with gefitinib attenuated treatment resistance in transplanted lung tumors in mice. **A** The effect of different treatments on the PC9GR graft tumor volume in mice (n = 6); **B** the changes of the body weight in transplanted tumor mice; **C** effects of different treatments on the organ coefficient of mice (from left to right and from top to bottom, heart, liver, spleen, lung and kidney); **D** observation results of PC9GR transplanted tumor tissue in mice; **E** H&E staining of the mouse heart, liver, spleen, lung and kidney tissues (50 μm)

When the tumor volume increased to approximately 50–100 mm^3^ (day five after inoculation), a mouse NSCLC-resistant graft tumor model was successfully established. In the initial phase of drug administration, all the mice behaved normally. However, with the gradual expansion of tumor tissue, the behavioral status of the mice administered with ADI and ADI in conjunction with gefitinib was better compared with the model group and gefitinib group. Compared with the model group, the ADI monotherapy group, gefitinib monotherapy group, or combined treatment group effectively inhibits the growth of transplanted tumors in mice. Remarkably, the combined treatment group exhibited significantly more than the gefitinib-only group (Fig. 3A, B). There are no statistically significant differences in the weights of the groups of mice over time (Fig. 3C). On day 21 after administration, the mice were treated according to animal ethical standards, and their hearts, livers, spleens, lungs, and kidneys were removed and weighed. Organ coefficients were used as the evaluation criterion to evaluate the drug's potential toxicity to mice (Fig. 3D). No toxicity or side effects were detected in the heart, liver, spleen, lung or kidney of transplanted tumor model mice in either the single or combined treatment group (Fig. 3D). The histopathological examination of these mice did not reveal any apparent abnormalities (Fig. 3E). In addition, an experimental study assessed the cell proliferation index, and the ADI monotherapy group and gefitinib monotherapy group had a more remarkable ability to inhibit cell proliferation in tumor tissue compared with the model group (Fig. S1A, 1B). In contrast to the single treatment group, the combined treatment group exhibited a significant inhibitory effect. The ADI monotherapy group and the gefitinib monotherapy group reduced the microvascular density of the tumor tissue compared with the model group. Remarkably, the combined treatment group showed a decrease in the microvascular density of the tumor tissue (Fig. S1A, 1C). TUNEL staining experiments show that the ADI and gefitinib groups exhibit higher proportions of apoptotic cells in tumor tissues compared with the model group. In comparison, the combined treatment group had higher proportions of apoptotic cells than the single-treatment groups (Fig. S1A, 1D).

### Synergistic mechanism of Aidi injection based on whole-transcriptome data for gefitinib treatment of non-small cell lung cancer

**Fig. 4:**
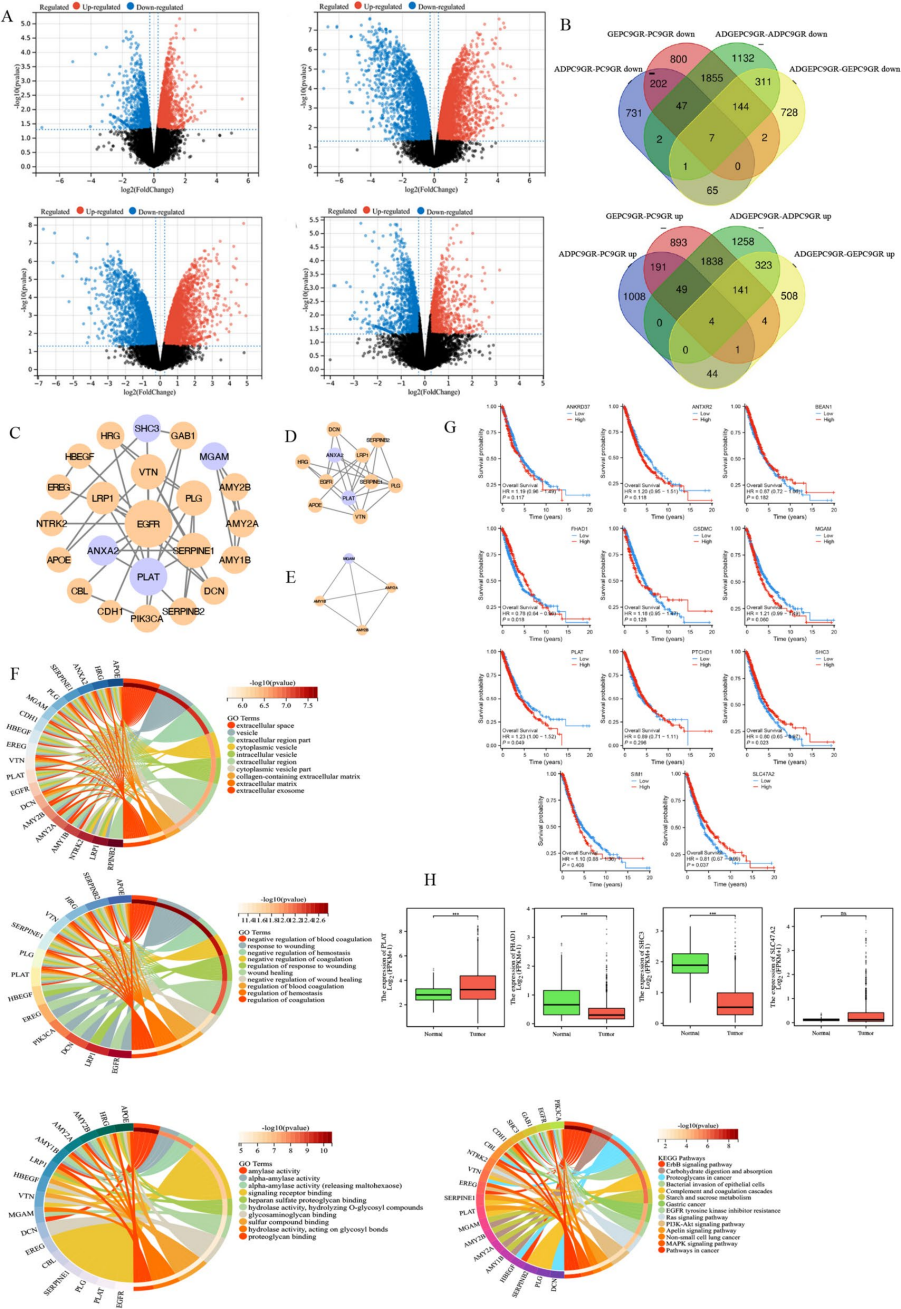
Synergistic mechanism of Aidi injection based on whole-transcriptome data for gefitinib treatment of non-small cell lung cancer. **A** The volcano map of related differential genes in drug-resistant lung cancer cells (PC9GR), drug-resistant ADI-treated lung cancer cells (ADPC9GR), drug-resistant gefitinib-treated lung cancer cells (GEPC9GR) and drug-resistant ADI and gefitinib-treated lung cancer cells (ADGEPC9GR); **B** Venn diagram analysis revealed downregulated and upregulated mRNAs in the ADPC9GR-PC9GR, ADPC9GR-ADPC9GR, GEPC9GR-PC9GR and ADGEPC9GR-GEPC9GR; **C**-**E** The interaction analysis of the differential genes (**F**) GO and KEGG enrichment analysis; **G** Survival data of lung cancer patients in the TCGA cohort; **H** The expression of PLAT in tumor tissues in the TCGA database

Whole transcriptomic sequencing was performed on four subgroups of drug-resistant lung cancer cells (PC9GR), drug-resistant ADI-treated lung cancer cells (ADPC9GR), drug-resistant gefitinib-treated lung cancer cells (GEPC9GR) and drug-resistant ADI and gefitinib-treated lung cancer cells (ADGEPC9GR). Based on transcriptome sequencing analysis, 2352 differentially expressed mRNAs were identified in the ADPC9GR-PC9GR comparison, including 1297 upregulated genes and 1055 downregulated genes (Fig. 4A). In the ADGEPC9GR-ADPC9GR comparison, 7112 differential genes were detected, including 3613 upregulated and 3499 downregulated genes (Fig. 4A). In the GEPC9GR-PC9GR comparison, 6180 differential genes were detected, including 3121 upregulated and 3057 downregulated genes (Fig. 4A). In the ADGEPC9GR-GEPC9GR comparison, 2283 differential genes were detected, including 1025 upregulated and 1258 downregulated genes (Fig. 4A). Venn diagram analysis revealed downregulated and upregulated mRNAs in the ADPC9GR-PC9GR, ADPC9GR-ADPC9GR, GEPC9GR-PC9GR and ADGEPC9GR-GEPC9GR. Seven common differentially expressed genes in downregulated mRNAs (ANTXR2, GSDMC, SHC3, MGAM, FHAD1, PLAT, and BEAN1) and four common differentially expressed genes in upregulated mRNAs (PTCHD1, ANKRD37, SIM1, SLC47A2) were identified (Fig. 4B). The results of the interaction analysis of the differential genes showed that the biological functions and signaling pathways associated with these differential genes, indicating that the mechanism of ADI intervention in resistant lung cancer cells is not limited to a few specific genes or a single pathway but involves complex signaling network interactions (Fig. 4C–E). Among the CC terms, the differential genes were enriched in categories such as extracellular space, cytoplasmic sac, and intracellular vesicle. Among the MF terms, the differential genes were enriched in categories such as amylase activity, α-amylase activity, and proteoglycan binding. Among the BP terms, the differential genes were enriched in categories such as coagulation regulation, wound healing, and hemostasis regulation. The KEGG terms were enriched in the PI3K-AKT signaling pathway, ErbB signaling pathway, complement and coagulation cascade, EGFR tyrosine kinase inhibitor resistance and other pathways (Fig. 4F). Survival data of lung cancer patients stratified by the expression of critical genes in the TCGA cohort were analyzed, and the PLAT was found to be a prognostic risk factor (Fig. 4G). PLAT is significantly upregulated in tumor tissues in the TCGA database (*P* < 0.05) (Fig. 4H). In summary, PLAT was upregulated in tumor tissues in the TCGA database, and the omics sequencing data for PC9GR-PC9 was downregulated after mono- or combined therapeutic administrations. The trend after administration was contrary to the pathological trend. PLAT may be the key gene involved in the synergistic effect of ADI combined with gefitinib, and further analysis was conducted to verify this hypothesis.

### Analysis of PLAT as a key prognostic marker of lung cancer

**Fig. 5:**
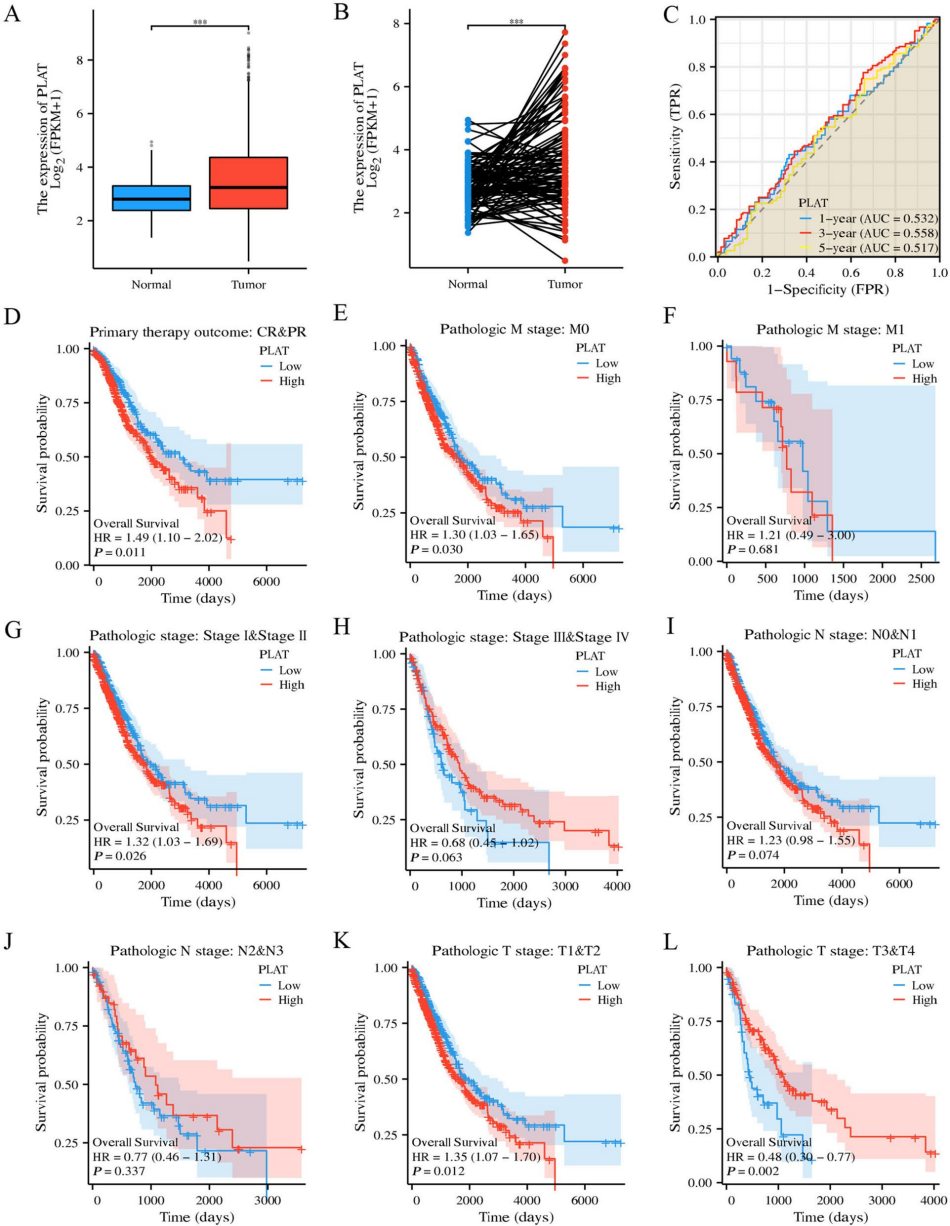
PLAT as a key prognostic marker of lung cancer. **A**-**B** The mRNA expression of PLAT in lung cancer tissues in the TCGA database; **C** Prognostic analysis of PLAT high expression; **D**–**L** The correlation between high expression of PLAT and higher **M** stage and pathological stage of lung cancer

**Fig. 6:**
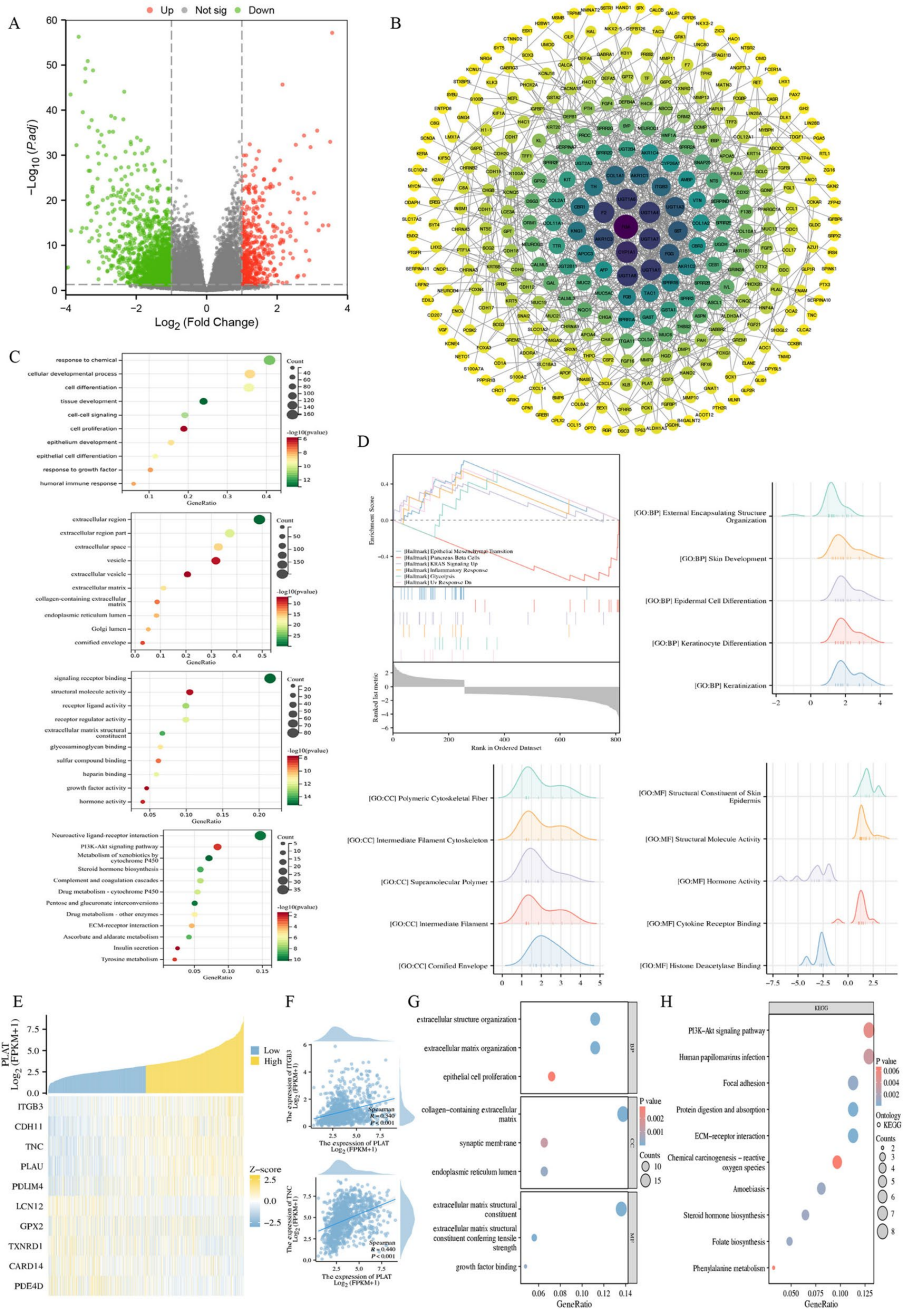
The analysis of difference in high and low PLAT expression according to the median PLAT expression score. **A** The volcano map of differential genes between the high- and low-PLAT expression groups; **B** The protein interaction network with differentially expressed genes related to PLAT; **C** Enrichment analysis of the interacting differentially expressed genes; **D** Gene set enrichment analysis (GSEA) of the differential genes between the high and low PLAT expression groups; **E** Correlation analysis of genes coexpressed with PLAT in lung cancer; **F** PLAT was coexpressed with ITGB3 and TNC; **G**-**H** Enrichment analysis of differential genes coexpressed with PLAT

The data obtained from the TCGA database revealed that the mRNA expression of PLAT in lung cancer tissues is significantly higher than in adjacent normal tissues (Fig. 5A, B). High PLAT expression was associated with poor tumor prognosis (Fig. 5C). Furthermore, a significant correlation was observed between high expression of PLAT and higher M stage and pathological stage of lung cancer (Fig. 5D-L). The overall survival of lung cancer patients with high PLAT expression is short by survival analyses. Time-dependent ROC curve analysis reveals that PLAT had a particular prognostic value. TCGA patients were divided into a high or low expression group according to the median PLAT expression score. There were 821 coding genes whose expression differed between the high- and low-PLAT expression groups (Fig. 6A). The interactions with differentially expressed genes related to PLAT were further analyzed. A protein interaction network was constructed, and six critical modules with scores more substantial than four were obtained via the MCODE plug-in (Fig. 6B). Enrichment analysis of the interacting differentially expressed genes was performed (Fig. 6C). The differential genes were enriched in the PI3K-Akt signaling pathway, the neuroactive ligand-receptor interaction, and cytochrome P450 of heterobiomass metabolism terms. Gene set enrichment analysis (GSEA) was conducted on the differential genes between the high and low PLAT expression groups. The results reveal that differential genes are significantly enriched in epithelial interstitial transformation and KRAS signaling in the high PLAT expression group (Fig. 6D). Correlation analysis of genes coexpressed with PLAT in lung cancer was undertaken via Spearman correlation analysis, and 902 genes coexpressed with PLAT were screened according to |Cor|> 0.3, *P* < 0.05, and further combined with PLAT-related differential genes. A total of 138 differential genes (the top 10 were ITGB3, CDH11, TNC, PLAU, PDLIM4, LCN12, GPX2, TXNRD1, CARD14 and PDE4D) are coexpressed with PLAT (Fig. 6E). High PLAT expression was significantly associated with a poor tumor prognosis, and PLAT was also coexpressed with ITGB3 and TNC (Fig. 6F). The differential genes coexpressed with PLAT are significantly enriched in the ECM receptor interaction pathway, focus adhesion pathway, PI3K-Akt signaling pathway and other pathways (Fig. 6G, H).

### The mechanism of action of PLAT in gefitinib-resistant non-small cell lung cancer

**Fig. 7:**
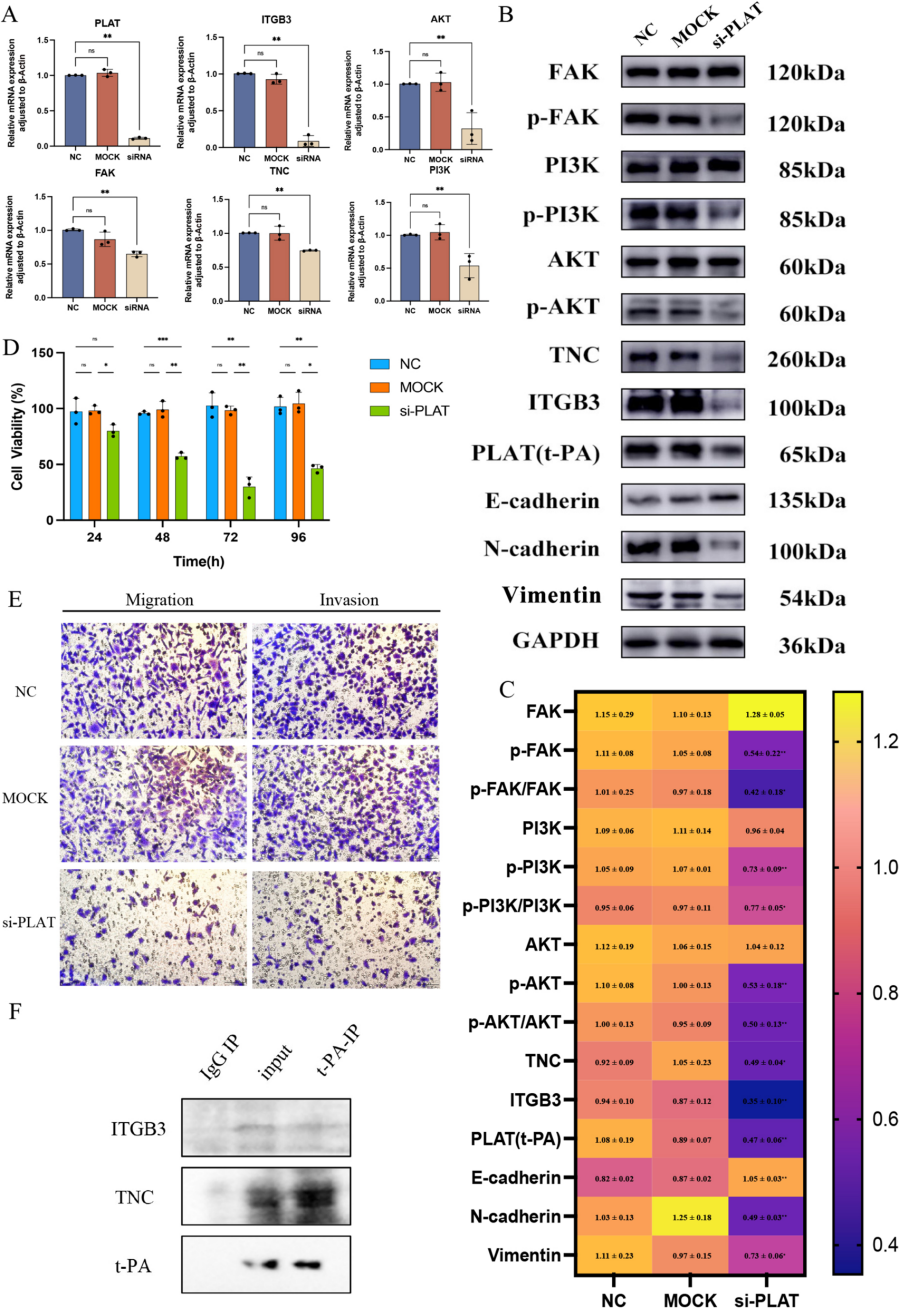
The mechanism of action of PLAT in gefitinib-resistant NSCLC. **A** The mRNA expression of PLAT, ITGB3, TNC, FAK, AKT and PI3K after PLAT knockdown; **B**-**C** protein expression bands of t-PA, ITGB3, TNC, FAK, AKT, PI3K, p-FAK, p-AKT, p-PI3K, Vimentin, E-cadherin, N-cadherin and protein expression heatmap after PLAT knockdown; **D** the cell viability of PC9GR by PLAT knockdown; **E** the migration and invasion of PLAT knockdown (100 μm); **F** combination of PLAT (t-PA) with ITGB3 and TNC. ^*^P < 0.05; ^**^P < 0.01

PLAT knockdown in PC9GR cells decreased the mRNA expression of ITGB3, AKT, FAK, TNC, PI3K and the protein expression of TNC, ITGB3, p-FAK, p-PI3K, and p-AKT (Fig. 7A-C). The protein expression of Vimentin and N-cadherin were decreased, while E-cadherin was increased (Fig. 7B). The cell viability of cells decreased after PLAT knockdown at 24, 48, 72 and 96 h (Fig. 7D). The migration and invasion of cells were also inhibited after PLAT knockdown (Fig. 7E). The CoIP results revealed an interaction between PLAT (t-PA) and TNC (Fig. 7F). These results suggest that PLAT regulates proliferation, migration and invasion and confers gefitinib resistance via TNC/FAK/AKT signaling.

### Aidi injection alone and combined with gefitinib inhibits PC9GR cell migration and invasion by regulating PLAT

**Fig. 8:**
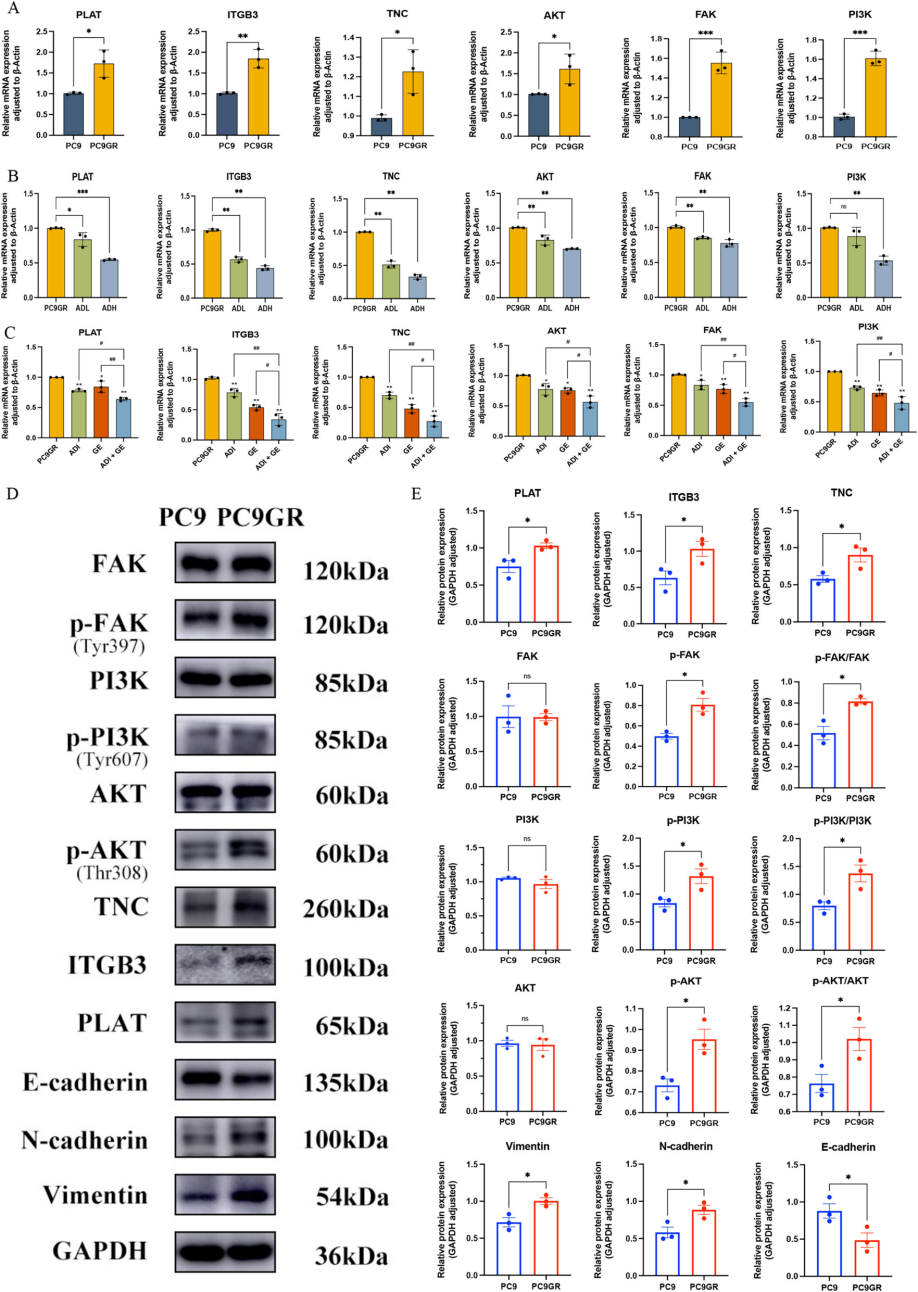
The mRNA and protein expression in PC9 and PC9GR cells and the changes of mRNA and protein expression in PC9GR cells after different drug treatments. **A** mRNA expression levels of PLAT, ITGB3, TNC, FAK, AKT and PI3K in PC9 and PC9GR cells; **B** the effect of ADI on the mRNA expression of PLAT, ITGB3, TNC, FAK and PI3K in PC9GR cells; **C** the effect of ADI combined with gefitinib on the mRNA expression of PLAT, ITGB3, TNC, FAK, AKT and, PI3K in PC9GR cells; **D** bands of PLAT (t-PA), ITGB3, TNC, FAK, AKT, PI3K, p-FAK, p-AKT, p-PI3K, Vimentin, E-cadherin, N-cadherin in PC9 and PC9GR cells; **E** the protein expression of PLAT (t-PA), ITGB3, TNC, FAK, AKT, PI3K, p-FAK, p-AKT, p-PI3K, Vimentin, E-cadherin, N-cadherin in PC9 and PC9GR cells. ^*^P < 0.05; ^**^P < 0.01

**Fig. 9:**
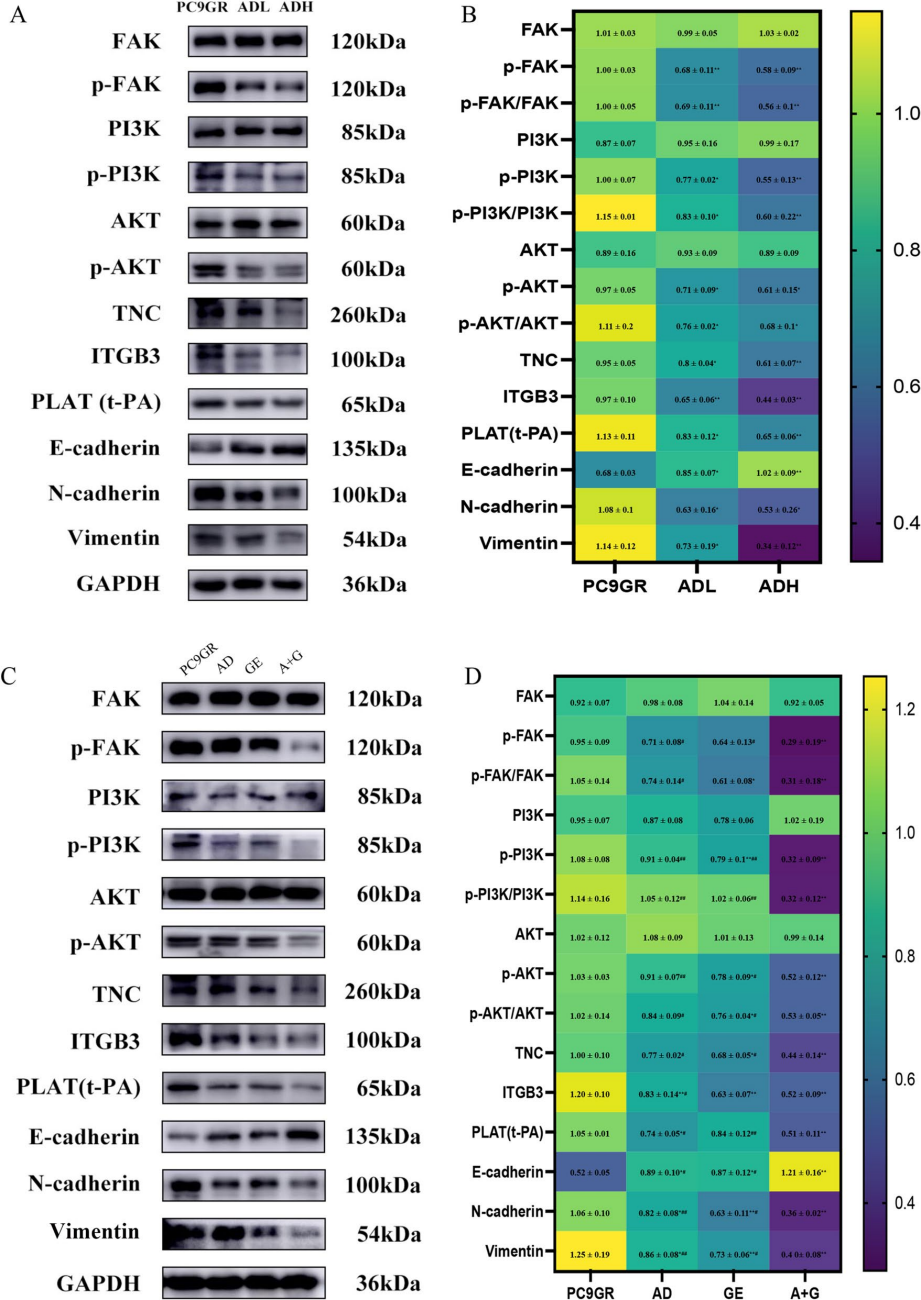
The influence of Aidi injection and its combination with gefitinib on crucial genes and proteins. **A** The protein expression of PLAT (t-PA), ITGB3, TNC, FAK, AKT, PI3K, p-FAK, p-AKT, p-PI3K, Vimentin, E-cadherin, and N-cadherin in PC9GR after ADI treatment; **B** heatmap of protein expression of PLAT (t-PA), ITGB3, TNC, FAK, AKT, PI3K, p-FAK, p-AKT, p-PI3K, Vimentin, E-cadherin, and N-cadherin in PC9GR cells; **C** the protein expression of PLAT (t-PA), ITGB3, TNC, FAK, AKT, PI3K, p-FAK, p-AKT, p-PI3K, Vimentin, E-cadherin, and N-cadherin in PC9GR cells after ADI, gefitinib and ADI combined with gefitinib treatment; **D** expression heatmap of PLAT (t-PA), ITGB3, TNC, FAK, AKT, PI3K, p-FAK, p-AKT, p-PI3K, Vimentin, E-cadherin, and N-cadherin proteins in PC9GR cells after ADI, gefitinib and ADI combined with gefitinib treatment

Compared with those in PC9 lung cancer cells, the mRNA expression of PLAT, ITGB3, TNC, AKT, FAK and PI3K were upregulated in PC9GR (Fig. 8A). After treatment with Aidi injection Low-dose (ADL) and Aidi injection High-dose (ADH) in PC9GR cells, the mRNA expression of PLAT, ITGB3, TNC, AKT, FAK and PI3K were downregulated (Fig. 8B). Compared with gefitinib administration alone, the difference was statistically significant after gefitinib combined with ADI, indicating that ADI combined with gefitinib could inhibit the migration and invasion of gefitinib-resistant lung cancer cells by interfering with PLAT expression (Fig. 8C). Compared with those in PC9 lung cancer cells, the protein expression of Vimentin and N-cadherin, which are related to migration and invasion, were increased (Fig. 8D, E). A reduction in E-cadherin expression indicated that gefitinib resistance increased the degree of lung cancer cell malignancy and enhanced migration and invasion ability of tumor cells (Fig. 8D, E). After adding ADI to gefitinib-resistant PC9GR cells, the protein expression of PLAT, TNC, ITGB3, p-FAK, p-PI3K, p-AKT, Vimentin and N-cadherin decreased (Fig. 9A, B). The expression of E-cadherin was increased. ADI inhibited the migration and invasion of PC9GR by interfering with PLAT (Fig. 9A, B). At the coordinated dose, after ADI combined with gefitinib treatment in PC9GR cells, the expression of PLAT, TNC, ITGB3, p-FAK, p-PI3K, p-AKT, Vimentin and N-cadherin decreased (Fig. 9C, D). Nevertheless, the expression of E-cadherin was increased (Fig. 9C, D). The immunohistochemical staining results of the tumor tissue showed that the ratio of PLAT-, TNC- and ITGB3-positive cells decreased in the ADI combined with the gefitinib group. Compared with the gefitinib monotherapy group, a significant decrease was detected in the combined treatment group (Fig. S2A-D). The results indicate that ADI combined with gefitinib could synergistically inhibit the migration and invasion of gefitinib-resistant lung cancer cells by inhibiting PLAT. This evidence further supports that ADI inhibits PC9GR cell migration and invasion by regulating the PLAT/FAK/AKT pathway.

## Discussion

This study combined histological data with biological experimental methods to study the mechanism by which ADI inhibits gefitinib resistance in NSCLC in vivo and in vitro. In vitro cell experiments revealed the inhibitory effect of ADI alone and in combination with gefitinib on lung cancer cells. A model of gefitinib-resistant transplanted lung cancer tumors in mice was established in vivo. The results show that the ADI and gefitinib groups and combining the two therapies inhibit the growth of transplanted tumors in mice. The drug combination group had a greater inhibition compared with the gefitinib group. Whole-transcriptome sequencing and bioinformatics data analysis were performed on PC9, PC9GR, ADPC9GR, GEPC9GR and ADGEPC9GR. The crucial genes involved in the synergistic effect of ADI and gefitinib in NSCLC were identified, and the underlying molecular regulatory mechanism was further explored. PLAT is upregulated in tumor tissues in the TCGA database analysis, and the histological sequencing data analysis of PC9GR-PC9 confirmed that it is downregulated after mono- or combination drug administration. Combined treatment reversed the upregulation of PLAT detected in the analysis of pathological specimens, so PLAT may be the key gene involved in increasing the efficacy of ADI combined with gefitinib. Systematic bioinformatics analysis confirmed the potential of PLAT as a biomarker in lung cancer and other cancers. High expression of PLAT is significantly related to a poor tumor prognosis. In our research, PC9GR cells had higher expression of PLAT compared with PC9. At the same time, PLAT is coexpressed with ITGB3 and TNC. As demonstrated using RT‒qPCR and Western blotting, ADI inhibits the migration and invasion of PC9GR cells by regulating the PLAT/FAK/AKT pathway.

Plasminogen activator (PA) is a serine protease that catalyzes the conversion of plasminogen to plasmin. Currently, u-PA and t-PA are two different types of PA. The two kinds of PA have different physiological functions. u-PA mediates the process of tissue remodeling under various normal and pathological conditions, whereas t-PA primarily plays a role in the intravascular dissolution of blood clots [30–32]. In addition to its physiological functions, PA contributes to multifarious malignant transformation processes, including cell migration and proliferation, tumor invasion and metastasis [31, 33]. Elevated levels of PA occur in many human tumor types, and these changes are related to the poor prognosis of breast cancer [33–39], lung cancer [40], bladder cancer [41] and gastric cancer [42, 43]. The massive body of research indicates that activating the plasminogen system is crucial for the invasion of tumor cells [44]. Previous studies reported that t-PA is necessary for the migration and invasion phenotype of tumors and tumor-related angiogenesis in vivo [45]. Tumor growth depends on nutrients in the local microenvironment, which necessitates the establishment of a sufficient vascular network. The formation of functional microvessels occurs gradually during tumor progression and is characterized by endothelial cell migration, invasion and proliferation [46, 47]. t-PA influences the angiogenesis network related to tumor growth, but the mechanism by which t-PA promotes or induces tumor-related angiogenesis is still unclear. t-PA might enhance the availability of growth factors within the extracellular matrix for endothelial cells, indicating that the changes in endogenous t-PA levels could influence the process of cell differentiation and tumor progression [45].

Integrin is a cell receptor that interacts with the extracellular matrix (ECM) to activate downstream intracellular signal transduction pathways. Integrin β3 (ITGB3), also known as GP3A or CD61, is a highly studied integrin family member. It can bind to various extracellular matrix proteins, promoting cell proliferation and migration. ITGB3 plays a variety of essential functions in the progression of cancer and molecular reprogramming in the microenvironment [48]. Meantime, some studies have shown that ITGB3 is closely associated with drug resistance [49–51]. The NRP1-ITGB3 axis mediates the chemical resistance of breast cancer cells [52]. Other studies have shown that there is a positive relationship between the expression of ALK and integrin β3 in NSCLC patients with ALK rearrangements, and patients with NSCLC with ALK rearrangements can be treated with ALK inhibitors [53]. The overexpression of ITGB3 also contributes to resistance to EGFR inhibition via a complex formed by ITGB3/KRAS/RalB and the activation of TBK1 and NF-κB mediated by the complex [54].

Tenascin-C (TNC) is specifically and transiently expressed as an extracellular matrix glycoprotein during tissue injury. TNC plays various roles in tissue injury, mediating inflammation and fibrosis, thus achieving effective tissue repair. In addition, TNC is crucial for heart and artery injury, tumor angiogenesis and metastasis, and regulating stem cell behavior [55, 56]. High TNC expression is associated with a poor prognosis in cancers such as glioblastoma, breast cancer and colorectal cancer. High TNC levels are also associated with angiogenesis transition, high tumor vascular density and vascular leakage [57]. TNC also contributes to tumor survival, proliferation, invasion and lung metastasis [58].

Integrin αvβ3 interacts with extracellular matrix proteins, promoting proliferation and migration via phosphorylating downstream signals of the focal adhesion kinase pathway (FAK) [59]. TNC exhibits elevated expression during the process of organogenesis. TNC protein is re-expressed in oncogenesis, which undergoes tissue remodeling. It has been reported that the deposition of TNC increases in the tumor matrix of most malignant epithelial tumors. TNC expression is induced by the interaction between stromal cells and epithelial (cancer) [60]. TNC deposited in the cancer stroma regulates the cell behavior of two cell types through interactions between cells and the ECM mediated by integrins. When integrins bind to extracellular ligands, they trigger intracellular signals [61]. Additionally, integrins collaborate with growth factor-coupled receptors and G protein-coupled receptors, which modulate biological processes like apoptosis, cell proliferation and differentiation, cell migration and invasion [62]. Wang et al. [63] showed that the reduction of TNC levels in tumors hinders the biological behavior of human umbilical vein endothelial cells (HUVECs) via the ITGB3/FAK/Akt signaling pathway. Naik et al. [52] described elevated baseline NRP-1 as associated with upregulated TNC/ITGB3 signal transduction, and NRP-1 overexpression in BT-474 increased cell sensitivity to adriamycin/cyclophosphamide. Previous studies showed that ITGB3 expression is related to tumor invasion and metastasis in breast cancer through mechanisms involving FAK and Akt signal transduction [64–66]. In addition, Nagaharu et al. [67] reported that TNC combines with the integrins ανβ1 and ανβ6 through Src-mediated FAK signaling to trigger the cytoplasmic translocation of E-cadherin and β-catenin, which promotes migration. Adhesion plaque acts as a scaffold for many signal transduction pathways as a subcellular structure, involving integrins or mechanical forces exerted on cells [68]. Many molecules in the focal adhesion complex are involved in downstream signal transduction pathways, such as the AKT1, MAPK/ERK and Wnt signaling pathways [69–71]. Apoptosis, cell proliferation, cell migration and angiogenesis may all be affected by the focal adhesion complex [72–74].

## Conclusion

In summary, ADI effectively inhibits the growth and metastasis of non-small cell lung cancer, enhancing the immune response and protecting normal tissues from damage. The molecular mechanism by which ADI inhibits NSCLC metastasis involves the regulation of the PLAT/FAK/AKT pathway to suppress the migration and invasion of gefitinib-resistant PC9GR cells. The outcomes of this study provide preclinical evidence that ADI has the potential to play an essential role in treating NSCLC.

## Supplementary Information

Additional file 1.

Additional file 2.

Additional file 3.

## Data Availability

Data are available upon request to the corresponding authors.

## Abbreviations

ADI: Aidi injection
ADL: Aidi injection low-dose
ADM: Aidi injection medium-dose
ADH: Aidi injection high-dose
CCK-8: Cell counting Kit-8
ECM: Extracellular matrix
EGFR: Epidermal growth factor receptor
LUAD: Lung adenocarcinoma
NSCLC: Non-small cell lung cancer
PA: Plasminogen activator
RCTs: Randomized controlled trials
ROC: Receiver operating characteristic
RT‒qPCR: Real-time quantitative PCR
TCM: Traditional Chinese medicine
TKIs: Tyrosine kinase inhibitors
TMS: Trans-3,5,4´-trimethoxystilbene
TNC: Tenascin-C
