# Correction: Aidi injection inhibits the migration and invasion of gefitinib-resistant lung adenocarcinoma cells by regulating the PLAT/FAK/AKT pathway

**DOI:** 10.1186/s13020-025-01070-9

**Published:** 2025-02-10

**Authors:** Jingyuan Zhang, Siyun Yang, Xiaodong Chen, Fanqin Zhang, Siyu Guo, Chao Wu, Tieshan Wang, Haojia Wang, Shan Lu, Chuanqi Qiao, Xiaoguang Sheng, Shuqi Liu, Xiaomeng Zhang, Hua Luo, Qinglin Li, Jiarui Wu

**Affiliations:** 1 Department of Clinical Chinese Pharmacy, School of Chinese Materia Medica, Beijing University of Chinese Medicine, Beijing, 102488 China; 2 Institute of Chinese Materia Medica, China Academy of Chinese Medical Sciences, Beijing, 100700 China; 3 Beijing Research Institute of Chinese Medicine, Beijing University of Chinese Medicine, Beijing, 102488 China; 4 Zhejiang Cancer Hospital, Hangzhou Institute of Medicine (HIM), Chinese Academy of Sciences, Hangzhou, 310022 Zhejiang China; 5 Macau Centre for Research and Development in Chinese Medicine, State Key Laboratory of Quality Research in Chinese Medicine, Institute of Chinese Medical Sciences, University of Macau, Macao, People’s Republic of China

**Correction: Chinese Medicine (2025) 20:2** 10.1186/s13020-024-01054-1

Following publication of the original article [1], the authors found that the background color of the histograms in panels C, E and F in Fig. 1 is incomplete.

The wrong Fig. 1 was:

**Fig. 1:**
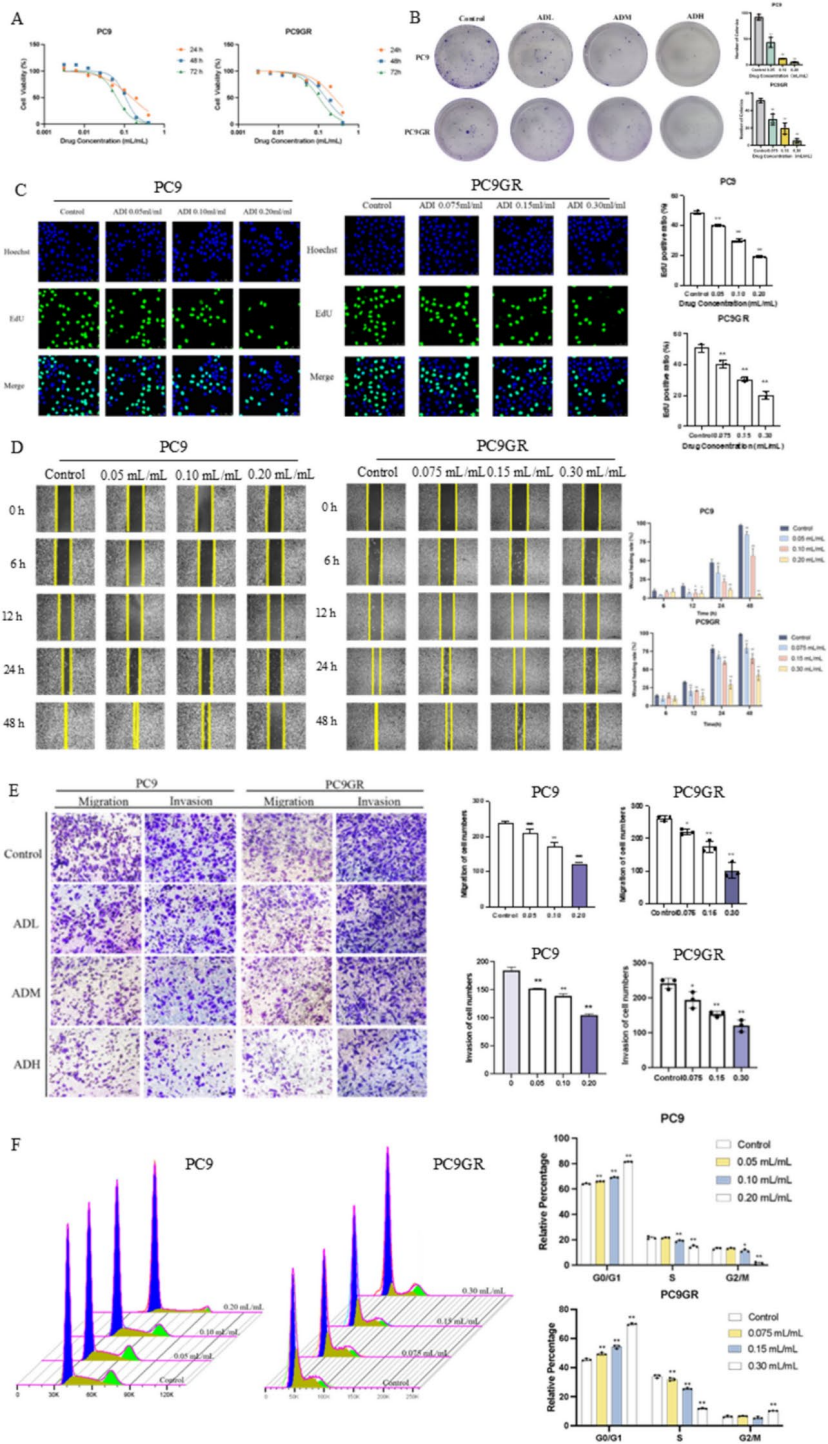
Aidi injection inhibits the malignant biological behavior of PC9 and PC9GR cells. **A** The inhibition of cell viability on PC9 and PC9GR cells by ADI; **B** the effect of ADI on the formation of PC9 and PC9GR cell colonies; **C** effect of ADI on the positive rate of EDU cells in PC9 and PC9GR cells (50 μm); **D** effect of ADI on the wound healing of PC9 and PC9GR cells; **E** effect of ADI on the migration and invasion of PC9 and PC9GR cells (100 μm); **F** three-dimensional diagram of the effect of ADI on PC9 and PC9GR cells cycle distribution and effect of ADI on the cell cycle of PC9 and PC9GR cells. ^*^P < 0.05; ^**^P < 0.01

The correct Fig. 1 should be:

**Fig. 1:**
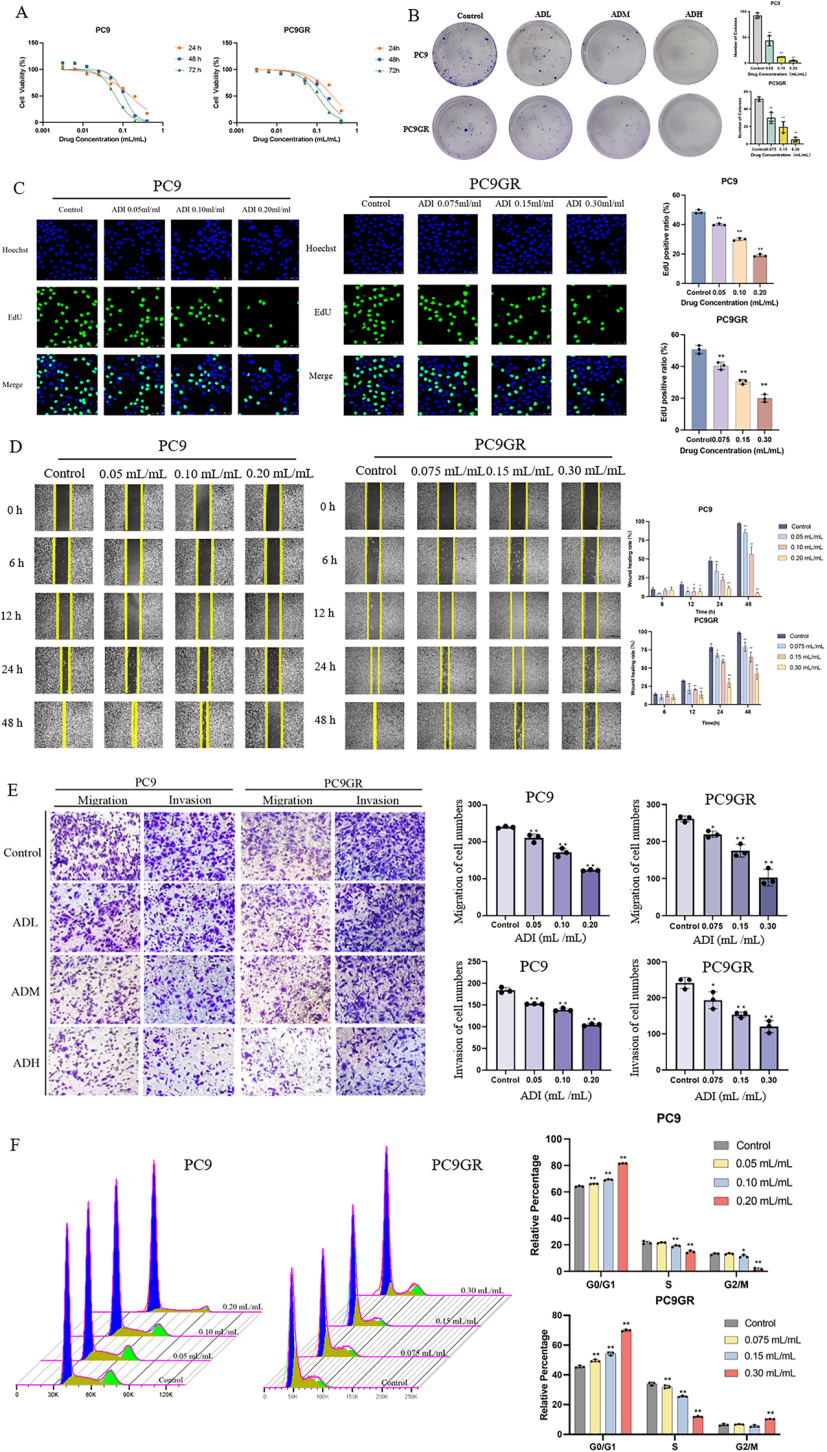
Aidi injection inhibits the malignant biological behavior of PC9 and PC9GR cells. **A** The inhibition of cell viability on PC9 and PC9GR cells by ADI; **B** the effect of ADI on the formation of PC9 and PC9GR cell colonies; **C** effect of ADI on the positive rate of EDU cells in PC9 and PC9GR cells (50 μm); **D** effect of ADI on the wound healing of PC9 and PC9GR cells; **E** effect of ADI on the migration and invasion of PC9 and PC9GR cells (100 μm); **F** three-dimensional diagram of the effect of ADI on PC9 and PC9GR cells cycle distribution and effect of ADI on the cell cycle of PC9 and PC9GR cells. ^*^P < 0.05; ^**^P < 0.01

The original article [1] has been updated.
