# Supplementary figures and images for: Aidi injection inhibits the migration and invasion of gefitinib-resistant lung adenocarcinoma cells by regulating the PLAT/FAK/AKT pathway
Source: Chin Med. 2025 Jan 3;20:2. doi: 10.1186/s13020-024-01054-1 (PMC11699780; doi:10.1186/s13020-024-01054-1)

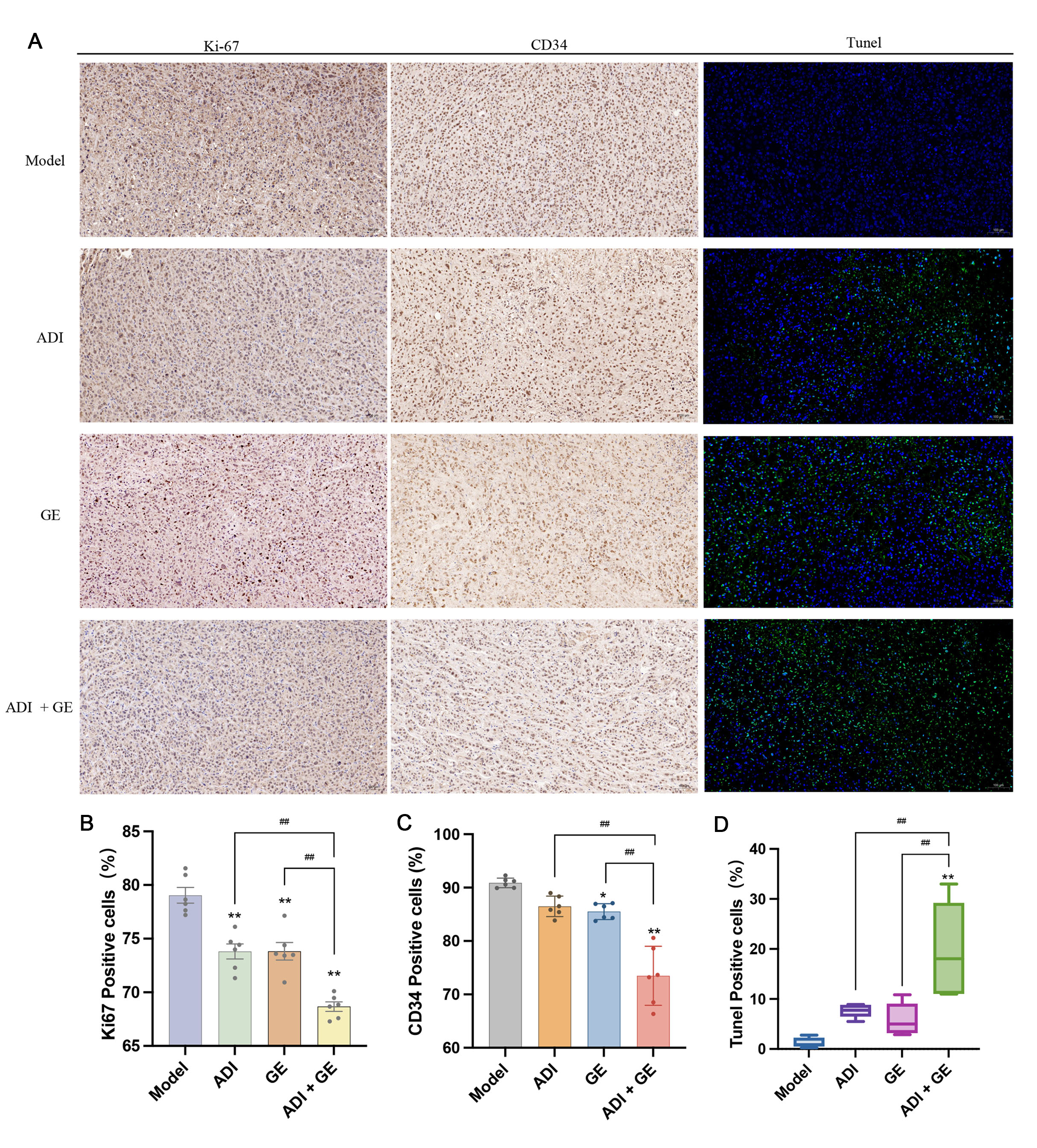

Supplement: Supplementary file 1 — Additional file 1. [file 13020_2024_1054_MOESM1_ESM.jpg]

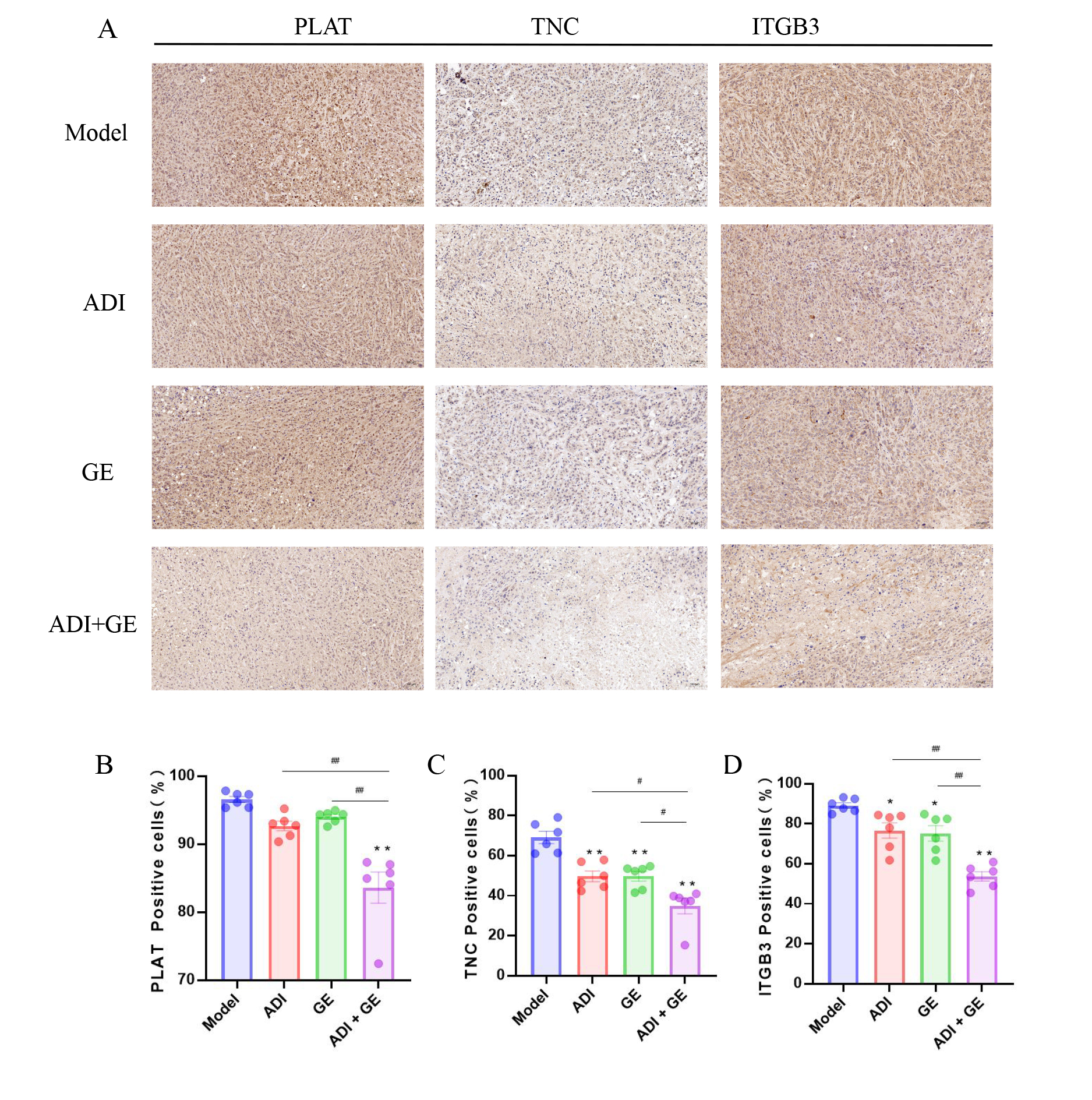

Supplement: Supplementary file 2 — Additional file 2. [file 13020_2024_1054_MOESM2_ESM.jpg]
