# Supplementary material for: Aidi injection inhibits the migration and invasion of gefitinib-resistant lung adenocarcinoma cells by regulating the PLAT/FAK/AKT pathway
Source: Chin Med. 2025 Jan 3;20:2. doi: 10.1186/s13020-024-01054-1 (PMC11699780; doi:10.1186/s13020-024-01054-1)
Supplement: Supplementary file 3 — Additional file 3. [file 13020_2024_1054_MOESM3_ESM.docx]

**Table S1.** **GSEA analysis of PLAT-related signatures in TCGA.**

| ID | Description | setSize | enrichmentScore | NES | pvalue | p.adjust | qvalue | rank | leading_edge |
| --- | --- | --- | --- | --- | --- | --- | --- | --- | --- |
| HALLMARK_EPITHELIAL_MESENCHYMAL_TRANSITION | HALLMARK_EPITHELIAL_MESENCHYMAL_TRANSITION | 30 | 0.65927561 | 3.97085795 | 1E-10 | 2.2E-09 | 1.5789E-09 | 254 | tags=97%, list=31%, signal=69% |
| HALLMARK_PANCREAS_BETA_CELLS | HALLMARK_PANCREAS_BETA_CELLS | 11 | -0.6686766 | -2.1647357 | 0.0003211 | 0.00353206 | 0.00253497 | 127 | tags=73%, list=16%, signal=62% |
| HALLMARK_KRAS_SIGNALING_UP | HALLMARK_KRAS_SIGNALING_UP | 18 | 0.40242959 | 1.96516204 | 0.00803599 | 0.05893056 | 0.04229466 | 251 | tags=72%, list=31%, signal=51% |
| HALLMARK_INFLAMMATORY_RESPONSE | HALLMARK_INFLAMMATORY_RESPONSE | 8 | 0.54767759 | 1.80048144 | 0.01652921 | 0.06462078 | 0.04637855 | 245 | tags=88%, list=30%, signal=62% |
| HALLMARK_GLYCOLYSIS | HALLMARK_GLYCOLYSIS | 8 | 0.54534161 | 1.79280196 | 0.01762385 | 0.06462078 | 0.04637855 | 374 | tags=100%, list=46%, signal=55% |
| HALLMARK_UV_RESPONSE_DN | HALLMARK_UV_RESPONSE_DN | 7 | 0.57172747 | 1.77700215 | 0.01292299 | 0.06462078 | 0.04637855 | 252 | tags=86%, list=31%, signal=60% |
| HALLMARK_HYPOXIA | HALLMARK_HYPOXIA | 8 | -0.537712 | -1.557695 | 0.04531722 | 0.14242555 | 0.10221929 | 196 | tags=62%, list=24%, signal=48% |

**Table S2. KEGG analysis of PLAT-related signatures in TCGA.**

| ID | Description | GeneRatio | pvalue | p.adjust | qvalue | geneID | Count |
| --- | --- | --- | --- | --- | --- | --- | --- |
| hsa00980 | Metabolism of xenobiotics by cytochrome P450 | 17/238 | 6.00E-11 | 9.08E-09 | 8.01E-09 | ALDH1A3/GSTA2/ALDH3A1/GSTA1/UGT2A3/UGT2B11/UGT2B4/CBR3/CBR1/AKR1C1/UGT1A3/UGT1A1/UGT1A4/UGT1A6/UGT1A7/UGT1A8/CYP1A1 | 17 |
| hsa04080 | Neuroactive ligand-receptor interaction | 35/238 | 1.03E-10 | 9.08E-09 | 8.01E-09 | CALCB/CCKBR/CRHR2/GALR1/GH2/GLP2R/GRIK3/MLNR/NTSR2/PTGFR/PTH2R/SSTR1/UCN2/CCKAR/CGA/GABRG3/GLP1R/TAC3/ADORA1/CALCA/CHRNA3/CHRNA5/CHRNB2/GABRA1/PRSS2/CHRNA9/GABBR2/PTH/GRIN2A/GAL/NTS/TAC1/KNG1/SST/F2 | 35 |
| hsa00040 | Pentose and glucuronate interconversions | 12/238 | 1.27E-10 | 9.08E-09 | 8.01E-09 | KL/AKR1B10/UGDH/UGT2A3/UGT2B11/UGT2B4/UGT1A3/UGT1A1/UGT1A4/UGT1A6/UGT1A7/UGT1A8 | 12 |
| hsa00140 | Steroid hormone biosynthesis | 14/238 | 1.71E-09 | 8.29E-08 | 7.31E-08 | UGT2A3/UGT2B11/UGT2B4/AKR1C4/AKR1C2/AKR1C1/UGT1A3/AKR1C3/UGT1A1/UGT1A4/UGT1A6/UGT1A7/UGT1A8/CYP1A1 | 14 |
| hsa05204 | Chemical carcinogenesis | 16/238 | 1.93E-09 | 8.29E-08 | 7.31E-08 | ALDH1A3/GSTA2/ALDH3A1/GSTA1/UGT2A3/UGT2B11/UGT2B4/AKR1C2/CBR1/UGT1A3/UGT1A1/UGT1A4/UGT1A6/UGT1A7/UGT1A8/CYP1A1 | 16 |
| hsa00053 | Ascorbate and aldarate metabolism | 10/238 | 2.70E-09 | 9.67E-08 | 8.52E-08 | UGDH/UGT2A3/UGT2B11/UGT2B4/UGT1A3/UGT1A1/UGT1A4/UGT1A6/UGT1A7/UGT1A8 | 10 |
| hsa04610 | Complement and coagulation cascades | 14/238 | 7.38E-08 | 2.27E-06 | 2.00E-06 | C8G/C8A/F7/PLAT/PLAU/F13B/PROC/SERPIND1/VTN/FGB/KNG1/FGG/F2/FGA | 14 |
| hsa00982 | Drug metabolism - cytochrome P450 | 13/238 | 1.74E-07 | 4.67E-06 | 4.12E-06 | ALDH1A3/GSTA2/ALDH3A1/GSTA1/UGT2A3/UGT2B11/UGT2B4/UGT1A3/UGT1A1/UGT1A4/UGT1A6/UGT1A7/UGT1A8 | 13 |
| hsa00860 | Porphyrin and chlorophyll metabolism | 9/238 | 3.24E-06 | 7.66E-05 | 6.75E-05 | UGT2A3/UGT2B11/UGT2B4/UGT1A3/UGT1A1/UGT1A4/UGT1A6/UGT1A7/UGT1A8 | 9 |
| hsa00983 | Drug metabolism - other enzymes | 12/238 | 3.56E-06 | 7.66E-05 | 6.75E-05 | GSTA2/CES1/GSTA1/UGT2A3/UGT2B11/UGT2B4/UGT1A3/UGT1A1/UGT1A4/UGT1A6/UGT1A7/UGT1A8 | 12 |
| hsa00830 | Retinol metabolism | 11/238 | 4.19E-06 | 8.20E-05 | 7.23E-05 | UGT2A3/UGT2B11/UGT2B4/CYP26A1/UGT1A3/UGT1A1/UGT1A4/UGT1A6/UGT1A7/UGT1A8/CYP1A1 | 11 |
| hsa00790 | Folate biosynthesis | 7/238 | 8.22E-06 | 0.00013592 | 0.00011978 | ALPI/TPH2/PAH/AKR1B10/CBR1/TH/AKR1C3 | 7 |
| hsa04950 | Maturity onset diabetes of the young | 7/238 | 8.22E-06 | 0.00013592 | 0.00011978 | FOXA3/HNF4A/RFX6/PAX4/HNF1A/NEUROD1/NEUROG3 | 7 |
| hsa04512 | ECM-receptor interaction | 11/238 | 5.99E-05 | 0.00091962 | 0.00081044 | TNC/DMP1/COMP/IBSP/ITGA11/THBS2/COL2A1/COL1A2/VTN/COL1A1/ITGB3 | 11 |
| hsa04974 | Protein digestion and absorption | 11/238 | 0.0001214 | 0.00174005 | 0.00153346 | PGA5/SLC6A19/SLC7A9/PRSS2/COL12A1/COL10A1/COL5A1/COL11A1/COL2A1/COL1A2/COL1A1 | 11 |
| hsa00340 | Histidine metabolism | 5/238 | 0.00050911 | 0.00684113 | 0.00602891 | ALDH1A3/AOC1/CNDP1/HAL/ALDH3A1 | 5 |
| hsa00360 | Phenylalanine metabolism | 4/238 | 0.00139369 | 0.01762607 | 0.01553338 | ALDH1A3/DDC/ALDH3A1/PAH | 4 |
| hsa04971 | Gastric acid secretion | 8/238 | 0.00176462 | 0.02107743 | 0.01857496 | CCKBR/KCNK2/ATP4A/KCNK10/CALML3/CALML5/GAST/SST | 8 |
| hsa00350 | Tyrosine metabolism | 5/238 | 0.00415035 | 0.04696444 | 0.04138849 | ALDH1A3/DDC/ALDH3A1/HGD/TH | 5 |
| hsa04151 | PI3K-Akt signaling pathway | 20/238 | 0.00484126 | 0.05204356 | 0.04586458 | GH2/TNC/EREG/GNG4/PCK1/FGF21/G6PC/FGF16/FGF5/COMP/FGF4/IBSP/ITGA11/THBS2/COL2A1/KIT/COL1A2/VTN/COL1A1/ITGB3 | 20 |
| hsa05226 | Gastric cancer | 11/238 | 0.00517928 | 0.05302592 | 0.04673031 | REG4/FZD10/CDH17/FGF21/FGF16/FGF5/WNT16/WNT7A/FGF4/CDX2/MUC2 | 11 |
| hsa00480 | Glutathione metabolism | 6/238 | 0.00641627 | 0.06270441 | 0.0552597 | G6PD/ODC1/GSTA2/GCLC/GPX2/GSTA1 | 6 |
| hsa04657 | IL-17 signaling pathway | 8/238 | 0.00671581 | 0.06277821 | 0.05532474 | CXCL6/CCL17/CSF2/MMP13/MMP3/S100A7/DEFB4A/MUC5AC | 8 |
| hsa04744 | Phototransduction | 4/238 | 0.00925524 | 0.08291151 | 0.07306767 | GNAT1/GRK1/CALML3/CALML5 | 4 |

**Table S3. GO analysis of PLAT-related signatures in TCGA.**

| ID | Description | GeneRatio | pvalue | p.adjust | qvalue | geneID | Count | ONT |
| --- | --- | --- | --- | --- | --- | --- | --- | --- |
| GO:0005576 | extracellular region | 197/403 | 1.49E-30 | 7.06E-28 | 5.52E-28 | ADGRV1/ALPI/C8G/CA6/CALCB/CDHR2/CEL/CLCA2/CPN1/EDIL3/GH2/GREB1/GUCA2B/IGFBP6/INHA/KERA/LCN12/LCN9/MSMB/NETO1/NRG4/ODAPH/OMD/OPTC/PGA5/PSG2/PSG4/PTGFR/PTX3/REG4/SERPINA10/SLC5A5/SLC6A19/SPINK1/SPINK4/SPX/SRPX2/TNC/UCN2/VGF/WFDC12/WFDC5/ZG16/ALDH1A3/ANGPTL3/ANO1/AOC1/AQP1/ATP4A/AZU1/BPIFB2/BPIFB6/CGA/CILP/CNDP1/COL8A2/CXCL14/CXCL6/DEFB126/DLK1/DSC3/ELANE/ENO3/EREG/GNG4/H2AW/KLK3/MMP10/NEB/NOTUM/PCSK2/RNASE7/S100B/SFRP4/SPAG11B/SYT4/TAC3/TDGF1/UMOD/APOF/BPIFA1/C8A/CALCA/CCL1/CFHR5/CSF2/DEFA6/F7/FCGBP/FGFBP1/FGL1/G6PD/GREM2/MATN3/MMP11/PCK1/PRSS2/SCG3/TGFBI/THPO/BPIFA2/CDH11/COL12A1/DDC/DEFA5/FGF21/GDF5/GREM1/GSTA2/H4C1/H4C13/HAPLN1/IGFBP1/KRT5/KRT6B/MMP13/NT5E/PLAT/PLAU/PPBP/SCG2/TF/TFF3/TXNRD1/ALDH3A1/APOA4/DMP1/FGF16/FGF5/GDNF/GPT/HGD/KRT14/MMP3/MUC13/MUC19/S100A7/TFF1/WNT16/WNT7A/APOA5/COMP/DEFB1/DEFB4A/FGF4/H4C6/IBSP/KL/ORM2/PTH/AKR1B10/CHGA/COL10A1/F13B/ASPN/CALML3/CALML5/COL5A1/DSG3/GAL/IVL/MUC6/ORM1/PROC/SERPINA7/THBS2/MUC2/MUC5AC/NTS/SERPIND1/UGDH/COL11A1/COL2A1/GAST/GSTA1/KIT/SPRR3/TTR/AFP/AKR1C4/AMBP/CBR3/COL1A2/VTN/APOC3/FGB/TAC1/CBR1/KNG1/AKR1C1/COL1A1/ITGB3/FGG/SST/AKR1C3/F2/FGA | 197 | CC |
| GO:0044421 | extracellular region part | 150/403 | 5.15E-21 | 1.22E-18 | 9.54E-19 | ADGRV1/C8G/CA6/CDHR2/CEL/EDIL3/GREB1/GUCA2B/IGFBP6/INHA/KERA/MSMB/OMD/OPTC/PGA5/PTX3/SERPINA10/SLC5A5/SLC6A19/SPINK1/SPX/SRPX2/TNC/VGF/ZG16/ALDH1A3/ANGPTL3/ANO1/AOC1/AQP1/ATP4A/AZU1/BPIFB2/CGA/CILP/COL8A2/CXCL14/DLK1/ELANE/ENO3/EREG/GNG4/H2AW/KLK3/MMP10/NEB/PCSK2/RNASE7/S100B/SFRP4/SYT4/TAC3/TDGF1/UMOD/APOF/BPIFA1/C8A/CALCA/CCL1/CSF2/F7/FCGBP/FGFBP1/FGL1/G6PD/GREM2/MMP11/PCK1/PRSS2/TGFBI/THPO/BPIFA2/CDH11/COL12A1/DDC/FGF21/GREM1/GSTA2/H4C1/H4C13/HAPLN1/KRT5/KRT6B/MMP13/NT5E/PLAT/PLAU/SCG2/TF/TXNRD1/ALDH3A1/APOA4/DMP1/FGF16/FGF5/GPT/HGD/KRT14/MMP3/MUC13/S100A7/WNT16/WNT7A/APOA5/COMP/DEFB1/DEFB4A/H4C6/IBSP/KL/ORM2/CHGA/COL10A1/ASPN/CALML3/COL5A1/DSG3/GAL/IVL/ORM1/SERPINA7/THBS2/MUC2/MUC5AC/SERPIND1/UGDH/COL11A1/COL2A1/GSTA1/KIT/SPRR3/TTR/AFP/AKR1C4/AMBP/CBR3/COL1A2/VTN/APOC3/FGB/CBR1/KNG1/AKR1C1/COL1A1/ITGB3/FGG/SST/AKR1C3/F2/FGA | 150 | CC |
| GO:0031012 | extracellular matrix | 45/403 | 3.65E-17 | 5.77E-15 | 4.51E-15 | EDIL3/KERA/OMD/OPTC/PTX3/SRPX2/TNC/ZG16/CILP/COL8A2/ELANE/MMP10/F7/MMP11/PRSS2/TGFBI/COL12A1/GREM1/HAPLN1/MMP13/APOA4/DMP1/MMP3/S100A7/WNT7A/COMP/ORM2/COL10A1/ASPN/COL5A1/ORM1/THBS2/MUC2/COL11A1/COL2A1/AMBP/COL1A2/VTN/APOC3/FGB/KNG1/COL1A1/FGG/F2/FGA | 45 | CC |
| GO:0005615 | extracellular space | 132/403 | 1.98E-16 | 2.35E-14 | 1.83E-14 | ADGRV1/C8G/CA6/CDHR2/CEL/EDIL3/GREB1/GUCA2B/IGFBP6/INHA/MSMB/OMD/PGA5/PTX3/SERPINA10/SLC5A5/SLC6A19/SPINK1/SPX/SRPX2/TNC/VGF/ALDH1A3/ANGPTL3/ANO1/AOC1/AQP1/ATP4A/AZU1/BPIFB2/CGA/CILP/CXCL14/DLK1/ELANE/ENO3/EREG/GNG4/H2AW/KLK3/NEB/PCSK2/RNASE7/S100B/SFRP4/TAC3/TDGF1/UMOD/APOF/BPIFA1/C8A/CALCA/CCL1/CSF2/FCGBP/FGFBP1/FGL1/G6PD/GREM2/PCK1/PRSS2/TGFBI/THPO/BPIFA2/CDH11/COL12A1/DDC/FGF21/GREM1/GSTA2/H4C1/H4C13/KRT5/KRT6B/NT5E/PLAT/PLAU/SCG2/TF/TXNRD1/ALDH3A1/APOA4/FGF16/FGF5/GPT/HGD/KRT14/MUC13/S100A7/WNT16/WNT7A/APOA5/COMP/DEFB1/DEFB4A/H4C6/IBSP/KL/ORM2/CHGA/CALML3/DSG3/GAL/IVL/ORM1/SERPINA7/MUC5AC/SERPIND1/UGDH/COL2A1/GSTA1/KIT/SPRR3/TTR/AFP/AKR1C4/AMBP/CBR3/COL1A2/VTN/APOC3/FGB/CBR1/KNG1/AKR1C1/COL1A1/ITGB3/FGG/SST/AKR1C3/F2/FGA | 132 | CC |
| GO:0010817 | regulation of hormone levels | 47/397 | 5.80E-17 | 2.74E-13 | 2.13E-13 | GALR1/INHA/IYD/KCNC2/SLC5A5/STXBP5L/SYBU/VGF/VSNL1/ALDH1A3/ANO1/BMP6/CASR/CGA/GLP1R/PCSK2/ABCC8/ADORA1/CHRNA3/HNF4A/RFX6/PPARGC1A/ABCC2/AKR1B10/GAL/HNF1A/NEUROD1/SERPINA7/CES1/SNAP25/TTR/UGT2B11/AFP/AKR1C4/CYP26A1/AKR1C2/FGB/TAC1/AKR1C1/FGG/UGT1A3/AKR1C3/UGT1A1/UGT1A7/UGT1A8/CYP1A1/FGA | 47 | BP |
| GO:0009605 | response to external stimulus | 113/397 | 1.41E-16 | 3.33E-13 | 2.59E-13 | ADGRV1/C8G/CCL15/CD207/CPN1/DPYSL5/GKN2/KCNK2/LHX1/MAG/PIK3C2G/PTGFR/PTX3/REG4/RGR/SLC10A2/SLC6A19/SPX/SSTR1/TNC/TRPM8/VGF/WFDC12/ALDH1A3/ANO1/AOC1/AQP1/AZU1/BMP6/BPIFB2/CASR/CXCL14/CXCL6/DEFB126/ELANE/FOXA3/GNAT1/GRK1/KCNC1/KIF5C/KLK3/LHX2/LMX1A/RET/RNASE7/SPAG11B/ADORA1/BPIFA1/C8A/CALCA/CCL1/CCL17/CFHR5/CSF2/DEFA6/F7/G6PD/HMGA2/ODC1/PCK1/PRSS2/SNAI2/TPH2/BPIFA2/DEFA5/FGF21/G6PC/GDF5/GREM1/NT5E/PLAT/PLAU/PPBP/SCG2/ALDH3A1/CHRNA9/FGF16/GCLC/GDNF/LCE3A/MMP3/OTX2/PPARGC1A/S100A7/ABCC2/DEFB1/DEFB4A/FGF4/KRT20/PTH/CHGA/GRIN2A/ASCL1/NQO1/PROC/SERPIND1/COL11A1/KIT/CYP26A1/VTN/FGB/TAC1/KNG1/TH/COL1A1/ITGB3/FGG/SST/AKR1C3/F2/UGT1A1/CYP1A1/FGA | 113 | BP |
| GO:0005102 | signaling receptor binding | 80/372 | 5.09E-16 | 3.61E-13 | 2.77E-13 | CALCB/CCKBR/CCL15/EDIL3/HAO1/IGFBP6/INHA/MAG/NETO1/NR0B1/NRG4/PPP1R1B/SPX/SRPX2/UCN2/VGF/ADAM2/ANGPTL3/BMP6/CASR/CGA/CXCL14/CXCL6/EREG/PRAME/S100B/TAC3/TDGF1/ADORA1/APOF/CALCA/CCL1/CCL17/CDH17/CSF2/F7/GREM2/TGFBI/THPO/CHGB/FGF21/GDF5/GREM1/HNF4A/IGFBP1/KLB/PLAT/PPBP/SCG2/TF/DMP1/FGF16/FGF5/GDNF/PPARGC1A/S100A7/TFF1/WNT7A/APOA5/COMP/DEFB1/DEFB4A/FGF4/IBSP/KL/PTH/COL5A1/GAL/NTS/COL2A1/TTR/VTN/APOC3/FGB/KNG1/ITGB3/FGG/SST/F2/FGA | 80 | MF |
| GO:0005796 | Golgi lumen | 21/403 | 4.04E-15 | 3.62E-13 | 2.83E-13 | KERA/OMD/ZG16/CGA/UMOD/DEFA6/F7/MMP11/DEFA5/MUC13/MUC19/MUC21/WNT7A/DEFB1/DEFB4A/MUC6/PROC/MUC2/MUC5AC/VTN/F2 | 21 | CC |
| GO:0005788 | endoplasmic reticulum lumen | 34/403 | 4.59E-15 | 3.62E-13 | 2.83E-13 | SERPINA10/TNC/VGF/BPIFB2/COL8A2/ENAM/NOTUM/F7/MATN3/SCG3/CHGB/COL12A1/IGFBP1/SCG2/TF/APOA4/DMP1/WNT7A/APOA5/COL10A1/COL5A1/PROC/CES1/SERPIND1/COL11A1/COL2A1/AFP/COL1A2/VTN/KNG1/COL1A1/FGG/F2/FGA | 34 | CC |
| GO:0005201 | extracellular matrix structural constituent | 25/372 | 8.45E-15 | 3.00E-12 | 2.30E-12 | EDIL3/OPTC/SRPX2/TNC/CILP/COL8A2/MATN3/TGFBI/COL12A1/HAPLN1/COMP/COL10A1/ASPN/COL5A1/MUC6/THBS2/MUC5AC/COL11A1/COL2A1/COL1A2/VTN/FGB/COL1A1/FGG/FGA | 25 | MF |
| GO:0048018 | receptor ligand activity | 37/372 | 1.06E-13 | 2.52E-11 | 1.93E-11 | CALCB/CCL15/INHA/NRG4/SPX/UCN2/VGF/ANGPTL3/BMP6/CGA/CXCL14/CXCL6/TDGF1/CALCA/CCL1/CSF2/GREM2/THPO/CHGB/FGF21/GDF5/GREM1/PPBP/SCG2/FGF16/FGF5/GDNF/TFF1/WNT7A/FGF4/KL/PTH/GAL/NTS/TTR/SST/F2 | 37 | MF |
| GO:0010469 | regulation of signaling receptor activity | 47/397 | 2.30E-14 | 3.62E-11 | 2.82E-11 | CALCB/CCL15/GH2/INHA/NETO1/NRG4/SPX/UCN2/VGF/ANGPTL3/BMP6/CGA/CXCL14/CXCL6/EREG/TDGF1/ADORA1/CALCA/CCL1/CCL17/CSF2/GREM2/NEFL/THPO/CHGB/FGF21/GDF5/GREM1/PLAU/PPBP/SCG2/FGF16/FGF5/GDNF/PPARGC1A/TFF1/WNT7A/FGF4/KL/PTH/GRIN2A/GAL/NTS/GAST/TTR/SST/F2 | 47 | BP |
| GO:0009888 | tissue development | 95/397 | 9.75E-14 | 1.15E-10 | 8.97E-11 | ADGRV1/CDHR2/HAND1/KCNK2/LHX1/NKX3-2/NR0B1/ODAPH/OMD/PAX7/TNC/TNMD/ZIC3/ALDH1A3/BMP6/CASR/DSC3/ENAM/ENO3/EREG/LHX2/NKX2-5/RET/S100B/SFRP4/TP63/UMOD/FOXN4/G6PD/HMGA2/INSM1/PTF1A/SNAI2/TGFBI/COL12A1/GDF5/GREM1/GSTA2/HAND2/HNF4A/IGFBP1/KRT5/KRT6B/MMP13/RFX6/TXNRD1/DMP1/GDNF/KRT14/LCE3A/PHOX2B/PPARGC1A/S100A7/WNT16/WNT7A/APOA5/COMP/FGF4/IBSP/KL/KRT20/PTH/CDX2/ASCL1/ASPN/CALML5/COL5A1/DSG3/GAL/IVL/NEUROD1/NEUROG3/PROC/SPRR2A/SPRR2F/SPRR2G/CES1/SPRR2B/SPRR2E/COL11A1/COL2A1/GSTA1/SPRR2D/SPRR3/COL1A2/SPRR1A/VTN/AKR1C2/SPRR1B/CBR1/AKR1C1/COL1A1/ITGB3/AKR1C3/CYP1A1 | 95 | BP |
| GO:0062023 | collagen-containing extracellular matrix | 35/403 | 2.06E-12 | 1.40E-10 | 1.09E-10 | EDIL3/OMD/SRPX2/TNC/ZG16/CILP/COL8A2/ELANE/F7/TGFBI/COL12A1/GREM1/HAPLN1/APOA4/S100A7/COMP/ORM2/COL10A1/ASPN/COL5A1/ORM1/THBS2/MUC2/COL11A1/COL2A1/AMBP/COL1A2/VTN/APOC3/FGB/KNG1/COL1A1/FGG/F2/FGA | 35 | CC |
| GO:0030545 | receptor regulator activity | 37/372 | 8.57E-13 | 1.52E-10 | 1.17E-10 | CALCB/CCL15/INHA/NRG4/SPX/UCN2/VGF/ANGPTL3/BMP6/CGA/CXCL14/CXCL6/TDGF1/CALCA/CCL1/CSF2/GREM2/THPO/CHGB/FGF21/GDF5/GREM1/PPBP/SCG2/FGF16/FGF5/GDNF/TFF1/WNT7A/FGF4/KL/PTH/GAL/NTS/TTR/SST/F2 | 37 | MF |
| GO:0008201 | heparin binding | 22/372 | 1.32E-12 | 1.87E-10 | 1.44E-10 | CCL15/CEL/REG4/SERPINA10/ANGPTL3/AOC1/AZU1/CXCL6/ELANE/FGFBP1/GREM2/H1-1/APOA5/COMP/FGF4/COL5A1/THBS2/SERPIND1/COL11A1/VTN/KNG1/F2 | 22 | MF |
| GO:0065008 | regulation of biological quality | 143/397 | 2.77E-13 | 2.62E-10 | 2.04E-10 | ADGRV1/CALCB/CCKBR/CCL15/CDHR2/CEL/CLDN18/CPLX2/GALR1/GRIK3/GUCA2B/INHA/IYD/KCNC2/KCNE4/LRFN2/MAG/NETO1/NTSR2/PPP1R1B/PTH2R/PTX3/RHAG/SERPINA10/SLC5A5/SPX/SRPX2/STXBP5L/SYBU/TRPM8/VGF/VSNL1/ALDH1A3/ANGPTL3/ANO1/AQP1/AZU1/BMP6/CASR/CGA/CXCL6/ELANE/FOXA3/GLDC/GLP1R/KCNC1/KCNK3/NEB/PCSK2/RET/S100B/SFRP4/SYT4/TP63/UMOD/ABCC8/ADORA1/BPIFA1/CALCA/CCL1/CHRNA3/CHRNA5/CHRNB2/F7/G6PD/LIN28A/NEFL/PCK1/SLC18A3/TPH2/DDC/DEFA5/FGF21/G6PC/H4C1/H4C13/HNF4A/PLAT/PLAU/RFX6/TF/APOA4/CHAT/CHRNA9/GCLC/GDNF/MUC13/PAH/PHOX2B/PPARGC1A/S100A7/WNT7A/ABCC2/APOA5/COMP/H4C6/KL/PTH/AKR1B10/CHGA/F13B/GRIN2A/GAL/HNF1A/MUC6/NEUROD1/NQO1/PROC/SERPINA7/SYP/THBS2/CES1/MUC2/NTS/SERPIND1/SNAP25/COL2A1/KIT/TTR/UGT2B11/AFP/AKR1C4/COL1A2/CYP26A1/VTN/AKR1C2/APOC3/FGB/TAC1/KNG1/TH/AKR1C1/COL1A1/ITGB3/FGG/UGT1A3/AKR1C3/F2/UGT1A1/UGT1A7/UGT1A8/CYP1A1/FGA | 143 | BP |
| GO:0019730 | antimicrobial humoral response | 19/397 | 1.85E-12 | 1.45E-09 | 1.13E-09 | AZU1/BPIFB2/CXCL6/DEFB126/ELANE/KLK3/RNASE7/BPIFA1/DEFA6/PRSS2/BPIFA2/DEFA5/S100A7/DEFB1/DEFB4A/CHGA/FGB/F2/FGA | 19 | BP |
| GO:0005539 | glycosaminoglycan binding | 24/372 | 1.25E-11 | 1.48E-09 | 1.13E-09 | CCL15/CEL/REG4/SERPINA10/ANGPTL3/AOC1/AZU1/CXCL6/ELANE/RNASE7/FGFBP1/GREM2/H1-1/HAPLN1/APOA5/COMP/FGF4/COL5A1/THBS2/SERPIND1/COL11A1/VTN/KNG1/F2 | 24 | MF |
| GO:0048513 | animal organ development | 133/397 | 3.05E-12 | 2.06E-09 | 1.60E-09 | ADGRV1/CCKBR/CLDN18/EMX2/HAND1/INHA/KCNC2/KCNK2/KERA/LHX1/NKX3-2/NR0B1/ODAPH/OMD/PAX7/PIR/PTX3/RHAG/SRY/SSTR1/TNC/VGF/ZFP42/ZIC3/ALDH1A3/AQP1/BMP6/CASR/CCKAR/COL8A2/CXCL14/DSC3/ENAM/EREG/GNAT1/KCNC1/KCNK3/LHX2/LMX1A/NEB/NEUROD4/NKX2-5/RET/S100B/SFRP4/SOX1/SOX3/SYT4/TDGF1/TP63/UMOD/CDH17/CHRNB2/CSF2/F7/FGL1/FOXN4/G6PD/HMGA2/INSM1/KIF1A/LIN28A/NEFL/ODC1/PHOX2A/PTF1A/SNAI2/TGFBI/THPO/FOXG1/GDF5/GREM1/H4C1/H4C13/HAND2/HNF4A/KRT5/KRT6B/MMP13/RFX6/CHRNA9/DMP1/FGF16/GDNF/KRT14/LCE3A/OTX2/PHOX2B/PPARGC1A/S100A7/WNT16/WNT7A/COMP/FGF4/H4C6/IBSP/KL/KRT20/PAX4/PTH/CDX2/GRIN2A/ASCL1/ASPN/COL5A1/DSG3/GAL/HNF1A/ITGA11/IVL/NEUROD1/NEUROG3/SPRR2A/SPRR2F/SPRR2G/SPRR2B/SPRR2E/COL11A1/COL2A1/KIT/SPRR2D/SPRR3/AFP/COL1A2/CYP26A1/SPRR1A/VTN/SPRR1B/TH/COL1A1/AKR1C3/UGT1A1/CYP1A1 | 133 | BP |
| GO:0042221 | response to chemical | 162/397 | 4.65E-12 | 2.75E-09 | 2.14E-09 | ADGRV1/CA6/CCL15/CLDN18/CPN1/CRHR2/DPYSL5/EMX2/GALR1/GH2/GLP2R/GUCY2C/IYD/KCNC2/KCNK2/LHX1/NR0B1/PIK3C2G/PPP1R1B/PTGFR/RHAG/SLC5A5/SLC6A19/SPINK4/SPX/SSTR1/SYBU/TNC/TNMD/UCN2/VGF/VSNL1/ANGPTL3/ANO1/AOC1/AQP1/AZU1/BMP6/CASR/CILP/CXCL14/CXCL6/ELANE/ENO3/EREG/FZD10/GABRG3/GLDC/GLP1R/GNAT1/KCNC1/KCNK3/KIF5C/LHX2/LMX1A/NKX2-5/RET/S100B/SFRP4/SH3GL2/SOX1/TDGF1/TP63/CALCA/CCL1/CCL17/CHRNA3/CHRNA5/CHRNB2/CSF2/F7/FGFBP1/G6PD/GABRA1/GREM2/HMGA2/LIN28A/NEFL/PCK1/SLC18A3/SNAI2/SRXN1/THPO/TPH2/CACNA1S/DDC/FGF21/G6PC/GDF5/GREM1/GSTA2/HNF4A/IGFBP1/KLB/MMP13/PLAU/RFX6/SCG2/TF/TXNRD1/ALDH3A1/APOA4/FGF16/FGF5/GCLC/GDNF/KRT14/MMP3/OTX2/PHOX2B/PPARGC1A/S100A7/TFF1/WNT16/WNT7A/ABCC2/COMP/DEFB1/DEFB4A/FGF4/IBSP/KL/PAX4/PTH/AKR1B10/CHGA/GRIN2A/ASCL1/ASPN/GAL/GPX2/NEUROD1/NQO1/SYP/CES1/SERPIND1/COL2A1/GSTA1/KIT/UGT2B11/AKR1C4/CBR3/COL1A2/CYP26A1/VTN/AKR1C2/FGB/TAC1/TH/AKR1C1/COL1A1/ITGB3/FGG/SST/UGT1A3/AKR1C3/UGT1A1/UGT1A6/UGT1A7/UGT1A8/CYP1A1/FGA | 162 | BP |
| GO:0009719 | response to endogenous stimulus | 78/397 | 5.50E-12 | 2.89E-09 | 2.25E-09 | CPN1/CRHR2/GH2/GLP2R/KCNC2/LHX1/NR0B1/PTGFR/SLC5A5/SSTR1/TNC/TNMD/UCN2/VGF/ANGPTL3/AOC1/AQP1/BMP6/CASR/CILP/EREG/GLP1R/KCNC1/NKX2-5/S100B/SFRP4/SH3GL2/TDGF1/TP63/CHRNA3/CHRNB2/F7/FGFBP1/GABRA1/GREM2/NEFL/PCK1/SNAI2/TPH2/CACNA1S/DDC/FGF21/GDF5/GREM1/HNF4A/IGFBP1/KLB/ALDH3A1/FGF16/FGF5/GCLC/OTX2/PHOX2B/PPARGC1A/TFF1/WNT7A/ABCC2/COMP/FGF4/KL/PTH/ASCL1/ASPN/GAL/NQO1/COL2A1/KIT/AKR1C4/COL1A2/AKR1C2/FGB/TAC1/TH/AKR1C1/COL1A1/SST/AKR1C3/UGT1A1 | 78 | BP |
| GO:0007267 | cell-cell signaling | 76/397 | 7.97E-12 | 3.76E-09 | 2.93E-09 | CCL15/CPLX2/CTNND2/GALR1/GRIK3/IGFBP6/INHA/KCNC2/LHX1/LRFN2/NETO1/STXBP5L/SYBU/SYT5/TNC/VGF/VSNL1/ANO1/BMP6/CASR/CXCL14/CXCL6/EREG/FZD10/GLP1R/KCNK3/NKX2-5/NOTUM/S100B/SFRP4/SYT4/TP63/WIF1/ABCC8/ADORA1/CCL17/CHRNA3/CHRNA5/CHRNB2/GABRA1/HMGA2/SLC18A3/SNAI2/CDH11/FGF21/GDF5/GREM1/HNF4A/IGFBP1/PLAT/RFX6/CHAT/CHRNA9/FGF16/FGF5/GABBR2/GDNF/KCNQ2/WNT16/WNT7A/FGF4/GRIN2A/GAL/HNF1A/NEUROD1/NQO1/SYP/SNAP25/KIT/FGB/TAC1/TH/COL1A1/FGG/SST/FGA | 76 | BP |
| GO:0070887 | cellular response to chemical stimulus | 123/397 | 1.13E-11 | 4.87E-09 | 3.79E-09 | ADGRV1/CLDN18/CRHR2/GH2/GLP2R/IYD/KCNC2/KCNK2/LHX1/NR0B1/PTGFR/SLC5A5/SSTR1/SYBU/TNC/TNMD/UCN2/VSNL1/ANO1/AOC1/AQP1/AZU1/BMP6/CASR/CILP/CXCL14/CXCL6/EREG/FZD10/GLDC/GLP1R/KCNC1/KCNK3/NKX2-5/RET/S100B/SFRP4/SH3GL2/SOX1/TDGF1/TP63/CALCA/CCL1/CHRNA3/CHRNB2/CSF2/F7/FGFBP1/G6PD/GABRA1/GREM2/HMGA2/LIN28A/PCK1/SNAI2/SRXN1/THPO/TPH2/CACNA1S/DDC/FGF21/GDF5/GREM1/GSTA2/HNF4A/IGFBP1/KLB/RFX6/SCG2/TF/TXNRD1/ALDH3A1/APOA4/FGF16/FGF5/GCLC/GDNF/MMP3/OTX2/PHOX2B/PPARGC1A/S100A7/WNT16/WNT7A/ABCC2/COMP/FGF4/IBSP/KL/PTH/AKR1B10/CHGA/ASPN/GPX2/NEUROD1/NQO1/SYP/CES1/COL2A1/GSTA1/KIT/UGT2B11/AKR1C4/CBR3/COL1A2/CYP26A1/VTN/AKR1C2/FGB/TAC1/TH/AKR1C1/COL1A1/ITGB3/FGG/SST/UGT1A3/AKR1C3/UGT1A1/UGT1A6/UGT1A7/UGT1A8/CYP1A1 | 123 | BP |
| GO:0001533 | cornified envelope | 12/403 | 9.80E-11 | 5.81E-09 | 4.54E-09 | DSC3/DSG3/IVL/SPRR2A/SPRR2F/SPRR2G/SPRR2B/SPRR2E/SPRR2D/SPRR3/SPRR1A/SPRR1B | 12 | CC |
| GO:0048731 | system development | 163/397 | 3.61E-11 | 1.42E-08 | 1.11E-08 | ADGRV1/CCKBR/CLDN18/CPLX2/CTNND2/DPYSL5/EMX2/HAND1/INHA/KCNC2/KCNK2/KERA/LHX1/MAG/NKX3-2/NR0B1/NRG4/ODAPH/OMD/PAX7/PIR/PTX3/RHAG/SRPX2/SRY/SSTR1/SYBU/TNC/VGF/ZFP42/ZIC3/ALDH1A3/ANGPTL3/AQP1/AZU1/BEX1/BMP6/CASR/CCKAR/COL8A2/CXCL14/DSC3/ENAM/EREG/FZD10/GNAT1/KCNC1/KCNK3/KIF5C/KLK3/LHX2/LMX1A/NEB/NEUROD4/NKX2-5/PCSK2/RET/S100B/SFRP4/SH3GL2/SOX1/SOX3/SYT4/TDGF1/TP63/UMOD/ADORA1/CDH17/CHRNA3/CHRNB2/CSF2/F7/FGFBP1/FGL1/FOXN4/G6PD/GABRA1/HMGA2/INSM1/KIF1A/LIN28A/MATN3/NEFL/ODC1/PHOX2A/PTF1A/SNAI2/TGFBI/THPO/CDH11/FOXG1/GDF5/GREM1/H4C1/H4C13/HAND2/HNF4A/KRT5/KRT6B/MMP13/RFX6/SCG2/CDH9/CHRNA9/DMP1/FGF16/FGF5/GDNF/KCNQ2/KRT14/LCE3A/OTX2/PHOX2B/PPARGC1A/S100A7/WNT16/WNT7A/COMP/FGF4/H4C6/IBSP/KL/KRT20/PAX4/PTH/CDX2/COL10A1/GRIN2A/ASCL1/ASPN/COL5A1/DSG3/GAL/HNF1A/ITGA11/IVL/NEUROD1/NEUROG3/SPRR2A/SPRR2F/SPRR2G/THBS2/SNAP25/SPRR2B/SPRR2E/COL11A1/COL2A1/KIT/SPRR2D/SPRR3/AFP/COL1A2/CYP26A1/SPRR1A/VTN/SPRR1B/TH/COL1A1/ITGB3/AKR1C3/F2/UGT1A1/CYP1A1 | 163 | BP |
| GO:0052695 | cellular glucuronidation | 9/397 | 4.81E-11 | 1.75E-08 | 1.36E-08 | UGT2A3/UGT2B11/UGT2B4/UGT1A3/UGT1A1/UGT1A4/UGT1A6/UGT1A7/UGT1A8 | 9 | BP |
| GO:0007586 | digestion | 19/397 | 1.01E-10 | 3.39E-08 | 2.64E-08 | ALPI/CCKBR/CEL/GUCA2B/GUCY2C/PGA5/PIR/UCN2/AQP1/PRSS2/MUC13/PPARGC1A/MUC6/NEUROD1/MUC2/AKR1C2/TAC1/AKR1C1/SST | 19 | BP |
| GO:0043062 | extracellular structure organization | 32/397 | 1.70E-10 | 5.35E-08 | 4.17E-08 | PTX3/TNC/COL8A2/ELANE/MMP10/MATN3/MMP11/PRSS2/COL12A1/GREM1/HAPLN1/MMP13/APOA4/DMP1/MMP3/APOA5/COMP/IBSP/COL10A1/COL5A1/ITGA11/COL11A1/COL2A1/TTR/COL1A2/VTN/APOC3/FGB/COL1A1/ITGB3/FGG/FGA | 32 | BP |
| GO:1901681 | sulfur compound binding | 23/372 | 6.65E-10 | 6.74E-08 | 5.17E-08 | CCL15/CEL/REG4/SERPINA10/ANGPTL3/AOC1/AZU1/CXCL6/ELANE/OGDHL/FGFBP1/GREM2/H1-1/APOA5/COMP/FGF4/COL5A1/THBS2/SERPIND1/COL11A1/VTN/KNG1/F2 | 23 | MF |
| GO:0030154 | cell differentiation | 141/397 | 2.55E-10 | 7.53E-08 | 5.87E-08 | ADGRV1/ANKS4B/CDHR2/CLDN18/CPLX2/CTNND2/DPYSL5/EMX2/GLIS1/GTSF1/HAND1/INHA/LHX1/MAG/MYPN/NKX3-2/NR0B1/PAX7/PIR/PTX3/RHAG/SPINK1/TNC/TNMD/ZIC3/AZU1/BEX1/BMP6/CCKAR/CXCL14/DLK1/DSC3/EREG/FZD10/GNAT1/KIF5C/LHX2/LMX1A/NEUROD4/NKX2-5/OCA2/PIWIL2/PRAME/RET/S100B/SFRP4/SH3GL2/SOX1/SOX3/SYT4/TDGF1/TP63/WIF1/CCL17/CDH17/CHRNA3/CHRNB2/CSF2/FOXN4/G6PD/HMGA2/INSM1/LIN28A/MMP11/NEFL/PHOX2A/PTF1A/SNAI2/TGFBI/THPO/CDH11/COL12A1/GDF5/GREM1/GSTA2/H4C1/H4C13/HAND2/HNF4A/KRT5/KRT6B/RFX6/FGF5/GDNF/KRT14/LCE3A/OTX2/PHOX2B/PPARGC1A/S100A7/TFF1/WNT16/WNT7A/COMP/DEFB1/FGF4/H4C6/IBSP/KRT20/PAX4/PTH/CDX2/GRIN2A/ASCL1/COL5A1/DSG3/GAL/HNF1A/ITGA11/IVL/NEUROD1/NEUROG3/PROC/SPRR2A/SPRR2F/SPRR2G/CES1/SNAP25/SPRR2B/SPRR2E/COL11A1/COL2A1/GSTA1/KIT/SPRR2D/SPRR3/SPRR1A/VTN/AKR1C2/FGB/SPRR1B/CBR1/TH/AKR1C1/COL1A1/ITGB3/FGG/AKR1C3/F2/CYP1A1/FGA | 141 | BP |
| GO:0030855 | epithelial cell differentiation | 46/397 | 3.50E-10 | 9.49E-08 | 7.39E-08 | CDHR2/LHX1/NKX3-2/NR0B1/BMP6/DSC3/EREG/SFRP4/TP63/INSM1/GREM1/GSTA2/HNF4A/KRT5/KRT6B/RFX6/GDNF/KRT14/LCE3A/S100A7/WNT16/WNT7A/KRT20/CDX2/ASCL1/DSG3/IVL/NEUROD1/NEUROG3/PROC/SPRR2A/SPRR2F/SPRR2G/CES1/SPRR2B/SPRR2E/GSTA1/SPRR2D/SPRR3/SPRR1A/AKR1C2/SPRR1B/CBR1/AKR1C1/AKR1C3/CYP1A1 | 46 | BP |
| GO:0009410 | response to xenobiotic stimulus | 26/397 | 3.62E-10 | 9.49E-08 | 7.39E-08 | PPP1R1B/AOC1/AQP1/CHRNB2/F7/CACNA1S/HNF4A/ALDH3A1/GCLC/PPARGC1A/ABCC2/GRIN2A/NQO1/CES1/UGT2B11/CBR3/CYP26A1/TAC1/TH/AKR1C1/UGT1A3/UGT1A1/UGT1A6/UGT1A7/UGT1A8/CYP1A1 | 26 | BP |
| GO:0030198 | extracellular matrix organization | 29/397 | 4.03E-10 | 1.00E-07 | 7.80E-08 | PTX3/TNC/COL8A2/ELANE/MMP10/MATN3/MMP11/PRSS2/COL12A1/GREM1/HAPLN1/MMP13/DMP1/MMP3/COMP/IBSP/COL10A1/COL5A1/ITGA11/COL11A1/COL2A1/TTR/COL1A2/VTN/FGB/COL1A1/ITGB3/FGG/FGA | 29 | BP |
| GO:0042445 | hormone metabolic process | 23/397 | 4.88E-10 | 1.15E-07 | 8.98E-08 | IYD/SLC5A5/ALDH1A3/BMP6/CGA/PCSK2/PPARGC1A/AKR1B10/GAL/CES1/TTR/UGT2B11/AFP/AKR1C4/CYP26A1/AKR1C2/AKR1C1/UGT1A3/AKR1C3/UGT1A1/UGT1A7/UGT1A8/CYP1A1 | 23 | BP |
| GO:0006063 | uronic acid metabolic process | 9/397 | 7.33E-10 | 1.57E-07 | 1.23E-07 | UGT2A3/UGT2B11/UGT2B4/UGT1A3/UGT1A1/UGT1A4/UGT1A6/UGT1A7/UGT1A8 | 9 | BP |
| GO:0019585 | glucuronate metabolic process | 9/397 | 7.33E-10 | 1.57E-07 | 1.23E-07 | UGT2A3/UGT2B11/UGT2B4/UGT1A3/UGT1A1/UGT1A4/UGT1A6/UGT1A7/UGT1A8 | 9 | BP |
| GO:0034754 | cellular hormone metabolic process | 17/397 | 8.47E-10 | 1.74E-07 | 1.35E-07 | ALDH1A3/BMP6/PPARGC1A/AKR1B10/TTR/UGT2B11/AFP/AKR1C4/CYP26A1/AKR1C2/AKR1C1/UGT1A3/AKR1C3/UGT1A1/UGT1A7/UGT1A8/CYP1A1 | 17 | BP |
| GO:0009914 | hormone transport | 27/397 | 1.65E-09 | 3.25E-07 | 2.53E-07 | GALR1/INHA/KCNC2/STXBP5L/SYBU/VGF/VSNL1/ANO1/BMP6/CASR/GLP1R/ABCC8/ADORA1/CHRNA3/HNF4A/RFX6/ABCC2/GAL/HNF1A/NEUROD1/SERPINA7/SNAP25/TTR/FGB/TAC1/FGG/FGA | 27 | BP |
| GO:0060429 | epithelium development | 62/397 | 1.74E-09 | 3.28E-07 | 2.56E-07 | CDHR2/HAND1/LHX1/NKX3-2/NR0B1/PAX7/TNC/ZIC3/ALDH1A3/BMP6/CASR/DSC3/EREG/LHX2/NKX2-5/RET/SFRP4/TP63/UMOD/FOXN4/INSM1/SNAI2/GREM1/GSTA2/HAND2/HNF4A/KRT5/KRT6B/RFX6/GDNF/KRT14/LCE3A/S100A7/WNT16/WNT7A/KRT20/CDX2/ASCL1/COL5A1/DSG3/GAL/IVL/NEUROD1/NEUROG3/PROC/SPRR2A/SPRR2F/SPRR2G/CES1/SPRR2B/SPRR2E/COL2A1/GSTA1/SPRR2D/SPRR3/SPRR1A/AKR1C2/SPRR1B/CBR1/AKR1C1/AKR1C3/CYP1A1 | 62 | BP |
| GO:0048869 | cellular developmental process | 142/397 | 2.41E-09 | 4.37E-07 | 3.41E-07 | ADGRV1/ANKS4B/CDHR2/CLDN18/CPLX2/CTNND2/DPYSL5/EMX2/GLIS1/GTSF1/HAND1/INHA/LHX1/MAG/MYPN/NKX3-2/NR0B1/PAX7/PIR/PTX3/RHAG/SPINK1/TNC/TNMD/ZIC3/AZU1/BEX1/BMP6/CCKAR/CXCL14/DLK1/DSC3/ENAM/EREG/FZD10/GNAT1/KIF5C/LHX2/LMX1A/NEUROD4/NKX2-5/OCA2/PIWIL2/PRAME/RET/S100B/SFRP4/SH3GL2/SOX1/SOX3/SYT4/TDGF1/TP63/WIF1/CCL17/CDH17/CHRNA3/CHRNB2/CSF2/FOXN4/G6PD/HMGA2/INSM1/LIN28A/MMP11/NEFL/PHOX2A/PTF1A/SNAI2/TGFBI/THPO/CDH11/COL12A1/GDF5/GREM1/GSTA2/H4C1/H4C13/HAND2/HNF4A/KRT5/KRT6B/RFX6/FGF5/GDNF/KRT14/LCE3A/OTX2/PHOX2B/PPARGC1A/S100A7/TFF1/WNT16/WNT7A/COMP/DEFB1/FGF4/H4C6/IBSP/KRT20/PAX4/PTH/CDX2/GRIN2A/ASCL1/COL5A1/DSG3/GAL/HNF1A/ITGA11/IVL/NEUROD1/NEUROG3/PROC/SPRR2A/SPRR2F/SPRR2G/CES1/SNAP25/SPRR2B/SPRR2E/COL11A1/COL2A1/GSTA1/KIT/SPRR2D/SPRR3/SPRR1A/VTN/AKR1C2/FGB/SPRR1B/CBR1/TH/AKR1C1/COL1A1/ITGB3/FGG/AKR1C3/F2/CYP1A1/FGA | 142 | BP |
| GO:0070268 | cornification | 16/397 | 3.44E-09 | 5.92E-07 | 4.61E-07 | DSC3/KRT5/KRT6B/KRT14/KRT20/DSG3/IVL/SPRR2A/SPRR2F/SPRR2G/SPRR2B/SPRR2E/SPRR2D/SPRR3/SPRR1A/SPRR1B | 16 | BP |
| GO:0071495 | cellular response to endogenous stimulus | 63/397 | 3.51E-09 | 5.92E-07 | 4.61E-07 | CRHR2/GH2/GLP2R/LHX1/NR0B1/PTGFR/SLC5A5/SSTR1/TNC/TNMD/UCN2/AOC1/AQP1/BMP6/CASR/CILP/GLP1R/NKX2-5/SFRP4/SH3GL2/TDGF1/TP63/CHRNA3/CHRNB2/FGFBP1/GABRA1/GREM2/PCK1/SNAI2/CACNA1S/DDC/FGF21/GDF5/GREM1/HNF4A/IGFBP1/KLB/FGF16/FGF5/GCLC/OTX2/PHOX2B/PPARGC1A/WNT7A/ABCC2/COMP/FGF4/KL/PTH/ASPN/COL2A1/KIT/AKR1C4/COL1A2/AKR1C2/FGB/TAC1/TH/AKR1C1/COL1A1/SST/AKR1C3/UGT1A1 | 63 | BP |
| GO:0005179 | hormone activity | 15/372 | 6.96E-09 | 6.17E-07 | 4.73E-07 | CALCB/INHA/SPX/UCN2/VGF/CGA/CALCA/THPO/CHGB/KL/PTH/GAL/NTS/TTR/SST | 15 | MF |
| GO:0071466 | cellular response to xenobiotic stimulus | 19/397 | 4.28E-09 | 6.98E-07 | 5.43E-07 | AOC1/AQP1/CACNA1S/HNF4A/ALDH3A1/PPARGC1A/ABCC2/NQO1/CES1/UGT2B11/CBR3/CYP26A1/AKR1C1/UGT1A3/UGT1A1/UGT1A6/UGT1A7/UGT1A8/CYP1A1 | 19 | BP |
| GO:0031982 | vesicle | 128/403 | 1.65E-08 | 8.69E-07 | 6.79E-07 | ADGRV1/C8G/CA6/CD207/CDHR2/CEL/EDIL3/GH2/GREB1/GUCA2B/IYD/KCNC2/NMNAT2/OMD/PGA5/PTX3/RAB3C/SERPINA10/SLC5A5/SLC6A19/SPINK1/SPX/STXBP5L/SYBU/SYT5/VGF/ZG16/ALDH1A3/ANGPTL3/ANO1/AOC1/AQP1/AZU1/BMP6/BPIFB2/CD1A/CILP/ELANE/ENO3/EREG/GNG4/H2AW/KCNK9/KLK3/NEB/OCA2/PCSK2/RET/SH3GL2/SYT4/UMOD/C8A/F7/FCGBP/G6PD/GABRA1/H1-1/KIF1A/PCK1/PRSS2/SCG3/SLC18A3/TGFBI/BPIFA2/CDH11/COL12A1/DDC/DEFA5/GSTA2/H4C1/H4C13/KRT5/KRT6B/NT5E/PLAT/PLAU/PPBP/SCG2/TF/TFF3/TXNRD1/APOA4/GPT/HGD/KRT14/S100A7/WNT7A/COMP/DEFB1/H4C6/IBSP/KL/ORM2/CHGA/GRIN2A/CALML3/CALML5/DSG3/IVL/ORM1/SERPINA7/SYP/THBS2/MUC5AC/NTS/SERPIND1/SNAP25/UGDH/GSTA1/KIT/SPRR3/TTR/AKR1C4/AMBP/COL1A2/VTN/APOC3/FGB/CBR1/KNG1/TH/AKR1C1/COL1A1/ITGB3/FGG/AKR1C3/F2/FGA | 128 | CC |
| GO:0005198 | structural molecule activity | 39/372 | 1.43E-08 | 1.13E-06 | 8.66E-07 | CLDN18/CLDN9/EDIL3/OPTC/SRPX2/TNC/CILP/CLDN8/COL8A2/MYL1/NEB/MATN3/MYBPH/NEFL/TGFBI/COL12A1/HAPLN1/KRT5/KRT6B/KRT14/COMP/KRT20/COL10A1/ASPN/COL5A1/MUC6/THBS2/MUC5AC/SPRR2E/COL11A1/COL2A1/SPRR3/COL1A2/SPRR1A/VTN/FGB/COL1A1/FGG/FGA | 39 | MF |
| GO:0042573 | retinoic acid metabolic process | 8/397 | 8.59E-09 | 1.35E-06 | 1.05E-06 | ALDH1A3/CYP26A1/UGT1A3/AKR1C3/UGT1A1/UGT1A7/UGT1A8/CYP1A1 | 8 | BP |
| GO:0015020 | glucuronosyltransferase activity | 9/372 | 1.91E-08 | 1.36E-06 | 1.04E-06 | UGT2A3/UGT2B11/UGT2B4/UGT1A3/UGT1A1/UGT1A4/UGT1A6/UGT1A7/UGT1A8 | 9 | MF |
| GO:0008083 | growth factor activity | 17/372 | 2.44E-08 | 1.57E-06 | 1.21E-06 | INHA/NRG4/VGF/ANGPTL3/BMP6/TDGF1/CSF2/THPO/FGF21/GDF5/PPBP/FGF16/FGF5/GDNF/TFF1/FGF4/F2 | 17 | MF |
| GO:0023061 | signal release | 31/397 | 1.06E-08 | 1.61E-06 | 1.26E-06 | GALR1/INHA/KCNC2/STXBP5L/SYBU/VGF/VSNL1/ANO1/BMP6/CASR/GLP1R/SYT4/ABCC8/ADORA1/CHRNA3/CHRNA5/CHRNB2/SLC18A3/HNF4A/RFX6/CHAT/WNT7A/GAL/HNF1A/NEUROD1/SYP/SNAP25/FGB/TAC1/FGG/FGA | 31 | BP |
| GO:0009725 | response to hormone | 49/397 | 1.12E-08 | 1.64E-06 | 1.28E-06 | CPN1/CRHR2/GH2/GLP2R/NR0B1/PTGFR/SLC5A5/SSTR1/TNC/UCN2/VGF/ANGPTL3/AQP1/BMP6/EREG/GLP1R/S100B/SFRP4/TP63/CHRNA3/CHRNB2/F7/NEFL/PCK1/TPH2/FGF21/HNF4A/IGFBP1/ALDH3A1/GCLC/PPARGC1A/TFF1/WNT7A/ABCC2/KL/PTH/GAL/NQO1/KIT/AKR1C4/AKR1C2/FGB/TAC1/TH/AKR1C1/COL1A1/SST/AKR1C3/UGT1A1 | 49 | BP |
| GO:0019748 | secondary metabolic process | 12/397 | 1.15E-08 | 1.64E-06 | 1.28E-06 | OCA2/DDC/ABCC2/AKR1B10/AKR1C4/AKR1C2/TH/AKR1C1/AKR1C3/UGT1A7/UGT1A8/CYP1A1 | 12 | BP |
| GO:0001523 | retinoid metabolic process | 14/397 | 1.25E-08 | 1.73E-06 | 1.35E-06 | ALDH1A3/APOA4/AKR1B10/TTR/AKR1C4/CYP26A1/APOC3/AKR1C1/UGT1A3/AKR1C3/UGT1A1/UGT1A7/UGT1A8/CYP1A1 | 14 | BP |
| GO:0006959 | humoral immune response | 24/397 | 1.32E-08 | 1.78E-06 | 1.38E-06 | C8G/CPN1/AZU1/BPIFB2/CXCL6/DEFB126/ELANE/KLK3/RNASE7/BPIFA1/C8A/CFHR5/DEFA6/PRSS2/BPIFA2/DEFA5/S100A7/DEFB1/DEFB4A/CHGA/VTN/FGB/F2/FGA | 24 | BP |
| GO:0070848 | response to growth factor | 41/397 | 1.75E-08 | 2.29E-06 | 1.78E-06 | KCNC2/LHX1/TNC/TNMD/BMP6/CASR/CILP/KCNC1/NKX2-5/SFRP4/SH3GL2/TDGF1/FGFBP1/GREM2/SNAI2/DDC/FGF21/GDF5/GREM1/KLB/FGF16/FGF5/GCLC/OTX2/PHOX2B/PPARGC1A/WNT7A/COMP/FGF4/IBSP/KL/PTH/ASCL1/ASPN/COL2A1/COL1A2/VTN/TAC1/TH/COL1A1/ITGB3 | 41 | BP |
| GO:0046983 | protein dimerization activity | 59/372 | 4.38E-08 | 2.54E-06 | 1.94E-06 | CLEC2A/H2BW1/HAND1/INHA/KLRF2/MAG/MYCN/NR0B1/SYT5/TRPM8/ALDH1A3/ANO1/AOC1/BMP6/CASR/ENO3/GLDC/H2AW/NEUROD4/NKX2-5/S100B/SH3GL2/SYT4/ADORA1/CFHR5/CHRNB2/DEFA6/G6PD/GREM2/KIF1A/NEFL/ODC1/PTF1A/DEFA5/GREM1/H4C1/H4C13/HAND2/HNF4A/APOA4/GABBR2/GCLC/GDNF/MUC13/H4C6/ASCL1/HNF1A/NEUROD1/NEUROG3/KIT/TTR/AMBP/FGG/UGT1A3/UGT1A1/UGT1A4/UGT1A6/UGT1A7/UGT1A8 | 59 | MF |
| GO:0005261 | cation channel activity | 22/372 | 4.64E-08 | 2.54E-06 | 1.94E-06 | GRIK3/KCNC2/KCNK2/SCN3A/TRPM8/ANO1/AQP1/KCNC1/KCNK3/KCNK9/ABCC8/CHRNA3/CHRNA5/CHRNB2/KCNJ18/KCNK10/CACNA1S/CHRNA9/KCNQ2/KCNQ5/GRIN2A/SNAP25 | 22 | MF |
| GO:0006805 | xenobiotic metabolic process | 15/397 | 2.04E-08 | 2.60E-06 | 2.03E-06 | AOC1/HNF4A/ALDH3A1/NQO1/CES1/UGT2B11/CBR3/CYP26A1/AKR1C1/UGT1A3/UGT1A1/UGT1A6/UGT1A7/UGT1A8/CYP1A1 | 15 | BP |
| GO:0061448 | connective tissue development | 22/397 | 2.60E-08 | 3.23E-06 | 2.51E-06 | HAND1/NKX3-2/PAX7/TNMD/BMP6/CASR/HMGA2/SNAI2/TGFBI/GDF5/GREM1/HAND2/MMP13/PPARGC1A/WNT7A/COMP/FGF4/PTH/COL5A1/COL11A1/COL2A1/COL1A1 | 22 | BP |
| GO:0061844 | antimicrobial humoral immune response mediated by antimicrobial peptide | 11/397 | 2.76E-08 | 3.34E-06 | 2.60E-06 | CXCL6/DEFB126/ELANE/KLK3/RNASE7/BPIFA1/DEFA5/S100A7/DEFB1/DEFB4A/F2 | 11 | BP |
| GO:0016101 | diterpenoid metabolic process | 14/397 | 2.90E-08 | 3.42E-06 | 2.66E-06 | ALDH1A3/APOA4/AKR1B10/TTR/AKR1C4/CYP26A1/APOC3/AKR1C1/UGT1A3/AKR1C3/UGT1A1/UGT1A7/UGT1A8/CYP1A1 | 14 | BP |
| GO:0010038 | response to metal ion | 26/397 | 3.38E-08 | 3.89E-06 | 3.03E-06 | ADGRV1/KCNC2/AOC1/AQP1/BMP6/CASR/KCNC1/KCNK3/G6PD/TPH2/TF/GCLC/KRT14/PPARGC1A/TFF1/ABCC2/PTH/ASCL1/NQO1/KIT/FGB/TH/FGG/AKR1C3/CYP1A1/FGA | 26 | BP |
| GO:1901700 | response to oxygen-containing compound | 68/397 | 3.67E-08 | 4.12E-06 | 3.21E-06 | CLDN18/CRHR2/GH2/GLP2R/KCNC2/PPP1R1B/PTGFR/SLC5A5/SYBU/TNC/VGF/VSNL1/ANO1/AOC1/AQP1/BMP6/CASR/CXCL6/ELANE/EREG/FZD10/GLDC/GLP1R/RET/CHRNA3/CHRNB2/CSF2/F7/G6PD/LIN28A/NEFL/PCK1/SNAI2/FGF21/HNF4A/IGFBP1/MMP13/RFX6/ALDH3A1/APOA4/GCLC/MMP3/PPARGC1A/S100A7/TFF1/WNT7A/ABCC2/KL/PAX4/PTH/AKR1B10/GRIN2A/ASCL1/GAL/NEUROD1/NQO1/AKR1C4/COL1A2/CYP26A1/AKR1C2/TAC1/TH/AKR1C1/COL1A1/SST/AKR1C3/UGT1A1/CYP1A1 | 68 | BP |
| GO:0048878 | chemical homeostasis | 51/397 | 3.76E-08 | 4.13E-06 | 3.21E-06 | CALCB/CCKBR/CCL15/GALR1/RHAG/SPX/STXBP5L/SYBU/TRPM8/VGF/VSNL1/ANGPTL3/ANO1/AQP1/BMP6/CASR/ELANE/FOXA3/GLP1R/KCNK3/SFRP4/TP63/UMOD/ADORA1/BPIFA1/CALCA/CCL1/LIN28A/PCK1/FGF21/G6PC/HNF4A/RFX6/TF/APOA4/CHRNA9/GCLC/PPARGC1A/ABCC2/APOA5/KL/PTH/GRIN2A/HNF1A/NEUROD1/APOC3/TAC1/KNG1/TH/AKR1C1/F2 | 51 | BP |
| GO:0022839 | ion gated channel activity | 22/372 | 1.13E-07 | 5.69E-06 | 4.36E-06 | CLCA2/GRIK3/KCNC2/KCNK2/SCN3A/ANO1/AQP1/KCNC1/KCNK3/KCNK9/CHRNA3/CHRNA5/CHRNB2/GABRA1/KCNJ18/KCNK10/CACNA1S/CHRNA9/KCNQ2/KCNQ5/GRIN2A/SNAP25 | 22 | MF |
| GO:0022836 | gated channel activity | 22/372 | 1.20E-07 | 5.69E-06 | 4.36E-06 | CLCA2/GRIK3/KCNC2/KCNK2/SCN3A/ANO1/AQP1/KCNC1/KCNK3/KCNK9/CHRNA3/CHRNA5/CHRNB2/GABRA1/KCNJ18/KCNK10/CACNA1S/CHRNA9/KCNQ2/KCNQ5/GRIN2A/SNAP25 | 22 | MF |
| GO:0050829 | defense response to Gram-negative bacterium | 12/397 | 5.52E-08 | 5.93E-06 | 4.62E-06 | AZU1/CXCL6/DEFB126/ELANE/RNASE7/DEFA5/LCE3A/S100A7/DEFB1/DEFB4A/CHGA/F2 | 12 | BP |
| GO:0046883 | regulation of hormone secretion | 22/397 | 5.75E-08 | 6.03E-06 | 4.70E-06 | GALR1/INHA/KCNC2/STXBP5L/SYBU/VSNL1/ANO1/BMP6/CASR/GLP1R/ABCC8/ADORA1/CHRNA3/HNF4A/RFX6/GAL/NEUROD1/SNAP25/FGB/TAC1/FGG/FGA | 22 | BP |
| GO:0005267 | potassium channel activity | 13/372 | 1.51E-07 | 6.71E-06 | 5.14E-06 | GRIK3/KCNC2/KCNK2/AQP1/KCNC1/KCNK3/KCNK9/ABCC8/KCNJ18/KCNK10/KCNQ2/KCNQ5/SNAP25 | 13 | MF |
| GO:0046879 | hormone secretion | 24/397 | 6.94E-08 | 7.12E-06 | 5.55E-06 | GALR1/INHA/KCNC2/STXBP5L/SYBU/VGF/VSNL1/ANO1/BMP6/CASR/GLP1R/ABCC8/ADORA1/CHRNA3/HNF4A/RFX6/GAL/HNF1A/NEUROD1/SNAP25/FGB/TAC1/FGG/FGA | 24 | BP |
| GO:1903561 | extracellular vesicle | 83/403 | 1.64E-07 | 7.35E-06 | 5.74E-06 | ADGRV1/C8G/CA6/CDHR2/CEL/EDIL3/GREB1/GUCA2B/OMD/PGA5/SERPINA10/SLC5A5/SLC6A19/SPINK1/ALDH1A3/ANO1/AOC1/AQP1/AZU1/BPIFB2/CILP/ELANE/ENO3/GNG4/H2AW/KLK3/NEB/SYT4/UMOD/C8A/FCGBP/G6PD/PCK1/TGFBI/BPIFA2/CDH11/COL12A1/DDC/GSTA2/H4C1/H4C13/KRT5/KRT6B/NT5E/PLAT/PLAU/TF/TXNRD1/APOA4/GPT/HGD/KRT14/WNT7A/COMP/DEFB1/H4C6/KL/ORM2/CALML3/DSG3/IVL/ORM1/SERPINA7/MUC5AC/SERPIND1/UGDH/GSTA1/SPRR3/TTR/AKR1C4/AMBP/COL1A2/VTN/APOC3/FGB/CBR1/KNG1/AKR1C1/ITGB3/FGG/AKR1C3/F2/FGA | 83 | CC |
| GO:0043230 | extracellular organelle | 83/403 | 1.71E-07 | 7.35E-06 | 5.74E-06 | ADGRV1/C8G/CA6/CDHR2/CEL/EDIL3/GREB1/GUCA2B/OMD/PGA5/SERPINA10/SLC5A5/SLC6A19/SPINK1/ALDH1A3/ANO1/AOC1/AQP1/AZU1/BPIFB2/CILP/ELANE/ENO3/GNG4/H2AW/KLK3/NEB/SYT4/UMOD/C8A/FCGBP/G6PD/PCK1/TGFBI/BPIFA2/CDH11/COL12A1/DDC/GSTA2/H4C1/H4C13/KRT5/KRT6B/NT5E/PLAT/PLAU/TF/TXNRD1/APOA4/GPT/HGD/KRT14/WNT7A/COMP/DEFB1/H4C6/KL/ORM2/CALML3/DSG3/IVL/ORM1/SERPINA7/MUC5AC/SERPIND1/UGDH/GSTA1/SPRR3/TTR/AKR1C4/AMBP/COL1A2/VTN/APOC3/FGB/CBR1/KNG1/AKR1C1/ITGB3/FGG/AKR1C3/F2/FGA | 83 | CC |
| GO:0006720 | isoprenoid metabolic process | 15/397 | 7.50E-08 | 7.54E-06 | 5.87E-06 | ALDH1A3/APOA4/AKR1B10/TTR/AKR1C4/CYP26A1/APOC3/TH/AKR1C1/UGT1A3/AKR1C3/UGT1A1/UGT1A7/UGT1A8/CYP1A1 | 15 | BP |
| GO:1901615 | organic hydroxy compound metabolic process | 32/397 | 8.49E-08 | 8.36E-06 | 6.51E-06 | CEL/HAO1/IYD/MTMR7/SLC5A5/ALDH1A3/ANGPTL3/BMP6/OCA2/APOF/CHRNB2/G6PD/INSM1/PCK1/SNAI2/TPH2/DDC/HAND2/APOA4/PAH/PTH/AKR1B10/GRIN2A/CES1/KIT/TTR/AKR1C4/AKR1C2/TH/AKR1C1/AKR1C3/CYP1A1 | 32 | BP |
| GO:0070062 | extracellular exosome | 82/403 | 2.12E-07 | 8.38E-06 | 6.55E-06 | ADGRV1/C8G/CA6/CDHR2/CEL/EDIL3/GREB1/GUCA2B/OMD/PGA5/SERPINA10/SLC5A5/SLC6A19/SPINK1/ALDH1A3/ANO1/AOC1/AQP1/AZU1/BPIFB2/CILP/ELANE/ENO3/GNG4/H2AW/KLK3/NEB/UMOD/C8A/FCGBP/G6PD/PCK1/TGFBI/BPIFA2/CDH11/COL12A1/DDC/GSTA2/H4C1/H4C13/KRT5/KRT6B/NT5E/PLAT/PLAU/TF/TXNRD1/APOA4/GPT/HGD/KRT14/WNT7A/COMP/DEFB1/H4C6/KL/ORM2/CALML3/DSG3/IVL/ORM1/SERPINA7/MUC5AC/SERPIND1/UGDH/GSTA1/SPRR3/TTR/AKR1C4/AMBP/COL1A2/VTN/APOC3/FGB/CBR1/KNG1/AKR1C1/ITGB3/FGG/AKR1C3/F2/FGA | 82 | CC |
| GO:0006721 | terpenoid metabolic process | 14/397 | 9.14E-08 | 8.81E-06 | 6.86E-06 | ALDH1A3/APOA4/AKR1B10/TTR/AKR1C4/CYP26A1/APOC3/AKR1C1/UGT1A3/AKR1C3/UGT1A1/UGT1A7/UGT1A8/CYP1A1 | 14 | BP |
| GO:0009812 | flavonoid metabolic process | 6/397 | 9.43E-08 | 8.91E-06 | 6.94E-06 | PPARGC1A/UGT1A3/UGT1A1/UGT1A7/UGT1A8/CYP1A1 | 6 | BP |
| GO:0003357 | noradrenergic neuron differentiation | 5/397 | 1.06E-07 | 9.60E-06 | 7.48E-06 | INSM1/PHOX2A/HAND2/PHOX2B/ASCL1 | 5 | BP |
| GO:0052697 | xenobiotic glucuronidation | 5/397 | 1.06E-07 | 9.60E-06 | 7.48E-06 | UGT2B11/UGT1A3/UGT1A1/UGT1A7/UGT1A8 | 5 | BP |
| GO:0010646 | regulation of cell communication | 123/397 | 1.17E-07 | 1.04E-05 | 8.12E-06 | ADGRV1/CALCB/CCL15/CPLX2/CTNND2/GALR1/GH2/GRIK3/IGFBP6/INHA/IRS4/KCNC2/LRFN2/NETO1/NR0B1/NRG4/SPINK1/SPX/STXBP5L/SYBU/UCN2/VGF/VSNL1/ANGPTL3/ANO1/BMP6/CASR/CGA/CILP/CXCL14/CXCL6/DLK1/ELANE/EREG/FZD10/GLP1R/GNAT1/GNG4/GRK1/NKX2-5/NOTUM/PRAME/RET/S100B/SFRP4/SH3GL2/SYT4/TAF1L/TDGF1/TP63/WIF1/ABCC8/ADORA1/CALCA/CCL1/CCL17/CHRNA3/CHRNA5/CHRNB2/CSF2/F7/FGFBP1/GREM2/LIN28A/NEFL/SNAI2/THPO/CDH11/CHGB/FGF21/GDF5/GREM1/HAND2/HNF4A/IGFBP1/KLB/MAGEA1/PLAT/PLAU/PPBP/RFX6/SCG2/CHRNA9/FGF16/FGF5/GCLC/GDNF/OTX2/PPARGC1A/S100A7/TFF1/WNT16/WNT7A/FGF4/KL/PTH/CHGA/GRIN2A/ASCL1/ASPN/GAL/NEUROD1/SYP/NTS/SNAP25/COL2A1/GAST/KIT/TTR/AMBP/CYP26A1/VTN/AKR1C2/APOC3/FGB/TAC1/COL1A1/ITGB3/FGG/SST/AKR1C3/F2/FGA | 123 | BP |
| GO:1900047 | negative regulation of hemostasis | 10/397 | 1.19E-07 | 1.04E-05 | 8.12E-06 | PLAT/PLAU/COMP/PROC/VTN/FGB/KNG1/FGG/F2/FGA | 10 | BP |
| GO:0051216 | cartilage development | 18/397 | 1.29E-07 | 1.11E-05 | 8.64E-06 | HAND1/NKX3-2/PAX7/BMP6/HMGA2/SNAI2/TGFBI/GDF5/GREM1/HAND2/MMP13/WNT7A/COMP/FGF4/PTH/COL11A1/COL2A1/COL1A1 | 18 | BP |
| GO:0042742 | defense response to bacterium | 21/397 | 1.38E-07 | 1.16E-05 | 9.05E-06 | VGF/WFDC12/AZU1/CXCL6/DEFB126/ELANE/KLK3/RNASE7/SPAG11B/BPIFA1/BPIFA2/DEFA5/PPBP/LCE3A/S100A7/DEFB1/DEFB4A/CHGA/FGB/F2/FGA | 21 | BP |
| GO:0071774 | response to fibroblast growth factor | 16/397 | 1.44E-07 | 1.19E-05 | 9.27E-06 | LHX1/TNC/CASR/KCNC1/TDGF1/FGFBP1/FGF21/KLB/FGF16/FGF5/GCLC/OTX2/FGF4/KL/PTH/COL1A1 | 16 | BP |
| GO:0005216 | ion channel activity | 24/372 | 2.90E-07 | 1.21E-05 | 9.27E-06 | CLCA2/GRIK3/KCNC2/KCNK2/SCN3A/TRPM8/ANO1/AQP1/KCNC1/KCNK3/KCNK9/ABCC8/CHRNA3/CHRNA5/CHRNB2/GABRA1/KCNJ18/KCNK10/CACNA1S/CHRNA9/KCNQ2/KCNQ5/GRIN2A/SNAP25 | 24 | MF |
| GO:0032787 | monocarboxylic acid metabolic process | 35/397 | 1.56E-07 | 1.27E-05 | 9.88E-06 | ACOT12/CEL/FADS6/HAO1/ALDH1A3/ANGPTL3/ENO3/HAL/OGDHL/PCK1/APOA4/PPARGC1A/ABCC2/APOA5/CES1/GSTA1/KIT/UGT2A3/UGT2B11/UGT2B4/AKR1C4/CYP26A1/AKR1C2/APOC3/CBR1/TH/AKR1C1/UGT1A3/AKR1C3/UGT1A1/UGT1A4/UGT1A6/UGT1A7/UGT1A8/CYP1A1 | 35 | BP |
| GO:0008324 | cation transmembrane transporter activity | 30/372 | 3.32E-07 | 1.31E-05 | 1.00E-05 | GRIK3/KCNC2/KCNK2/RHAG/SCN3A/SLC10A2/SLC17A2/SLC5A5/SLC6A19/TRPM8/ANO1/AQP1/KCNC1/KCNK3/KCNK9/OCA2/ABCC8/CHRNA3/CHRNA5/CHRNB2/KCNJ18/KCNK10/SLC18A3/CACNA1S/TF/CHRNA9/KCNQ2/KCNQ5/GRIN2A/SNAP25 | 30 | MF |
| GO:0043588 | skin development | 28/397 | 1.65E-07 | 1.32E-05 | 1.03E-05 | DSC3/EREG/LHX2/TP63/KRT5/KRT6B/KRT14/LCE3A/S100A7/WNT16/COMP/KRT20/COL5A1/DSG3/GAL/IVL/SPRR2A/SPRR2F/SPRR2G/SPRR2B/SPRR2E/SPRR2D/SPRR3/COL1A2/SPRR1A/SPRR1B/COL1A1/AKR1C3 | 28 | BP |
| GO:0048485 | sympathetic nervous system development | 7/397 | 2.22E-07 | 1.74E-05 | 1.36E-05 | TP63/INSM1/PHOX2A/HAND2/GDNF/PHOX2B/ASCL1 | 7 | BP |
| GO:0007423 | sensory organ development | 32/397 | 2.25E-07 | 1.74E-05 | 1.36E-05 | ADGRV1/KCNK2/KERA/LHX1/NKX3-2/ALDH1A3/BMP6/COL8A2/CXCL14/GNAT1/KCNK3/LHX2/NEUROD4/RET/SOX1/SOX3/FOXN4/PTF1A/HAND2/CHRNA9/PHOX2B/WNT16/WNT7A/PAX4/ASCL1/COL5A1/NEUROD1/COL11A1/COL2A1/KIT/TH/CYP1A1 | 32 | BP |
| GO:0009753 | response to jasmonic acid | 4/397 | 2.38E-07 | 1.76E-05 | 1.37E-05 | AKR1C4/AKR1C2/AKR1C1/AKR1C3 | 4 | BP |
| GO:0071395 | cellular response to jasmonic acid stimulus | 4/397 | 2.38E-07 | 1.76E-05 | 1.37E-05 | AKR1C4/AKR1C2/AKR1C1/AKR1C3 | 4 | BP |
| GO:0023051 | regulation of signaling | 123/397 | 2.41E-07 | 1.76E-05 | 1.37E-05 | ADGRV1/CALCB/CCL15/CPLX2/CTNND2/GALR1/GH2/GRIK3/IGFBP6/INHA/IRS4/KCNC2/LRFN2/NETO1/NR0B1/NRG4/SPINK1/SPX/STXBP5L/SYBU/UCN2/VGF/VSNL1/ANGPTL3/ANO1/BMP6/CASR/CGA/CILP/CXCL14/CXCL6/DLK1/ELANE/EREG/FZD10/GLP1R/GNAT1/GNG4/GRK1/NKX2-5/NOTUM/PRAME/RET/S100B/SFRP4/SH3GL2/SYT4/TAF1L/TDGF1/TP63/WIF1/ABCC8/ADORA1/CALCA/CCL1/CCL17/CHRNA3/CHRNA5/CHRNB2/CSF2/F7/FGFBP1/GREM2/LIN28A/NEFL/SNAI2/THPO/CDH11/CHGB/FGF21/GDF5/GREM1/HAND2/HNF4A/IGFBP1/KLB/MAGEA1/PLAT/PLAU/PPBP/RFX6/SCG2/CHRNA9/FGF16/FGF5/GCLC/GDNF/OTX2/PPARGC1A/S100A7/TFF1/WNT16/WNT7A/FGF4/KL/PTH/CHGA/GRIN2A/ASCL1/ASPN/GAL/NEUROD1/SYP/NTS/SNAP25/COL2A1/GAST/KIT/TTR/AMBP/CYP26A1/VTN/AKR1C2/APOC3/FGB/TAC1/COL1A1/ITGB3/FGG/SST/AKR1C3/F2/FGA | 123 | BP |
| GO:0010033 | response to organic substance | 110/397 | 2.42E-07 | 1.76E-05 | 1.37E-05 | CLDN18/CPN1/CRHR2/GH2/GLP2R/KCNC2/LHX1/NR0B1/PPP1R1B/PTGFR/SLC5A5/SSTR1/SYBU/TNC/TNMD/UCN2/VGF/VSNL1/ANGPTL3/ANO1/AOC1/AQP1/BMP6/CASR/CILP/CXCL6/ELANE/EREG/FZD10/GLDC/GLP1R/KCNC1/NKX2-5/RET/S100B/SFRP4/SH3GL2/SOX1/TDGF1/TP63/CHRNA3/CHRNB2/CSF2/F7/FGFBP1/G6PD/GABRA1/GREM2/LIN28A/NEFL/PCK1/SNAI2/THPO/TPH2/CACNA1S/DDC/FGF21/GDF5/GREM1/GSTA2/HNF4A/IGFBP1/KLB/MMP13/RFX6/ALDH3A1/APOA4/FGF16/FGF5/GCLC/MMP3/OTX2/PHOX2B/PPARGC1A/S100A7/TFF1/WNT7A/ABCC2/COMP/FGF4/IBSP/KL/PAX4/PTH/AKR1B10/GRIN2A/ASCL1/ASPN/GAL/NEUROD1/NQO1/SYP/COL2A1/KIT/AKR1C4/COL1A2/CYP26A1/VTN/AKR1C2/FGB/TAC1/TH/AKR1C1/COL1A1/ITGB3/FGG/SST/AKR1C3/UGT1A1/CYP1A1 | 110 | BP |
| GO:0022838 | substrate-specific channel activity | 24/372 | 4.73E-07 | 1.77E-05 | 1.36E-05 | CLCA2/GRIK3/KCNC2/KCNK2/SCN3A/TRPM8/ANO1/AQP1/KCNC1/KCNK3/KCNK9/ABCC8/CHRNA3/CHRNA5/CHRNB2/GABRA1/KCNJ18/KCNK10/CACNA1S/CHRNA9/KCNQ2/KCNQ5/GRIN2A/SNAP25 | 24 | MF |
| GO:0003008 | system process | 82/397 | 2.65E-07 | 1.90E-05 | 1.48E-05 | ADGRV1/CA6/CCKBR/CEL/GALR1/GUCA2B/INHA/KCNE4/KCNK2/KERA/MAG/MYBPC1/NETO1/NTSR2/PPP1R1B/RGR/SPX/TRPM8/ADAM2/ALDH1A3/ANO1/AQP1/AZU1/BMP6/CASR/GLP1R/GNAT1/GRK1/KCNK3/LMX1A/MYL1/NEB/NKX2-5/S100B/SYT4/UMOD/ADORA1/CHRNA3/CHRNA5/CHRNB2/FOXN4/G6PD/KCNK10/MYBPH/NEFL/SNAI2/TGFBI/CACNA1S/HAND2/CHRNA9/GCLC/GDNF/MUC13/PHOX2B/PPARGC1A/WNT7A/COMP/CHGA/GRIN2A/GAL/HNF1A/MUC6/NEUROD1/CES1/MUC2/NTS/SNAP25/COL11A1/COL2A1/KIT/CBR3/COL1A2/FGB/TAC1/KNG1/TH/AKR1C1/COL1A1/FGG/AKR1C3/UGT1A7/FGA | 82 | BP |
| GO:0015267 | channel activity | 25/372 | 6.86E-07 | 2.43E-05 | 1.86E-05 | CLCA2/GRIK3/KCNC2/KCNK2/RHAG/SCN3A/TRPM8/ANO1/AQP1/KCNC1/KCNK3/KCNK9/ABCC8/CHRNA3/CHRNA5/CHRNB2/GABRA1/KCNJ18/KCNK10/CACNA1S/CHRNA9/KCNQ2/KCNQ5/GRIN2A/SNAP25 | 25 | MF |
| GO:0022803 | passive transmembrane transporter activity | 25/372 | 7.21E-07 | 2.43E-05 | 1.86E-05 | CLCA2/GRIK3/KCNC2/KCNK2/RHAG/SCN3A/TRPM8/ANO1/AQP1/KCNC1/KCNK3/KCNK9/ABCC8/CHRNA3/CHRNA5/CHRNB2/GABRA1/KCNJ18/KCNK10/CACNA1S/CHRNA9/KCNQ2/KCNQ5/GRIN2A/SNAP25 | 25 | MF |
| GO:0015075 | ion transmembrane transporter activity | 36/372 | 7.53E-07 | 2.43E-05 | 1.86E-05 | CLCA2/GRIK3/KCNC2/KCNK2/RHAG/SCN3A/SLC10A2/SLC17A2/SLC5A5/SLC6A19/SLC7A9/TRPM8/ANO1/AQP1/KCNC1/KCNK3/KCNK9/OCA2/ABCC8/CHRNA3/CHRNA5/CHRNB2/GABRA1/KCNJ18/KCNK10/SLC18A3/SLCO1A2/CACNA1S/TF/CHRNA9/KCNQ2/KCNQ5/ABCC2/GRIN2A/SNAP25/AKR1C4 | 36 | MF |
| GO:0043025 | neuronal cell body | 28/403 | 6.98E-07 | 2.55E-05 | 1.99E-05 | CTNND2/DPYSL5/GRIK3/INHA/KCNC2/KCNK2/PPP1R1B/SYT5/CASR/GNAT1/KCNC1/KIF5C/PCSK2/RET/S100B/SH3GL2/SYT4/ADORA1/CHRNA3/KIF1A/DDC/PPARGC1A/ASCL1/GAL/NQO1/TAC1/TH/SST | 28 | CC |
| GO:0009617 | response to bacterium | 34/397 | 3.66E-07 | 2.58E-05 | 2.01E-05 | GKN2/PTGFR/REG4/SLC10A2/VGF/WFDC12/AZU1/BMP6/CXCL6/DEFB126/ELANE/KLK3/RNASE7/SPAG11B/BPIFA1/CSF2/PCK1/BPIFA2/DEFA5/PPBP/LCE3A/PPARGC1A/S100A7/ABCC2/DEFB1/DEFB4A/CHGA/FGB/TAC1/TH/F2/UGT1A1/CYP1A1/FGA | 34 | BP |
| GO:0015079 | potassium ion transmembrane transporter activity | 13/372 | 8.54E-07 | 2.63E-05 | 2.01E-05 | GRIK3/KCNC2/KCNK2/AQP1/KCNC1/KCNK3/KCNK9/ABCC8/KCNJ18/KCNK10/KCNQ2/KCNQ5/SNAP25 | 13 | MF |
| GO:0015318 | inorganic molecular entity transmembrane transporter activity | 34/372 | 8.88E-07 | 2.63E-05 | 2.01E-05 | CLCA2/GRIK3/KCNC2/KCNK2/SCN3A/SLC10A2/SLC17A2/SLC5A5/SLC6A19/SLC7A9/TRPM8/ANO1/AQP1/KCNC1/KCNK3/KCNK9/OCA2/ABCC8/CHRNA3/CHRNA5/CHRNB2/GABRA1/KCNJ18/KCNK10/SLCO1A2/CACNA1S/TF/CHRNA9/KCNQ2/KCNQ5/ABCC2/GRIN2A/SNAP25/AKR1C4 | 34 | MF |
| GO:0001101 | response to acid chemical | 24/397 | 3.81E-07 | 2.65E-05 | 2.06E-05 | PTGFR/TNC/AQP1/BMP6/FZD10/GLDC/RET/F7/PCK1/FGF21/GCLC/PPARGC1A/ABCC2/ASCL1/NQO1/AKR1C4/COL1A2/CYP26A1/AKR1C2/TH/AKR1C1/COL1A1/SST/AKR1C3 | 24 | BP |
| GO:0019752 | carboxylic acid metabolic process | 48/397 | 4.53E-07 | 3.10E-05 | 2.42E-05 | ACOT12/CEL/FADS6/HAO1/IYD/ALDH1A3/ANGPTL3/ENO3/GLDC/HAL/OGDHL/ODC1/PCK1/TPH2/DDC/GPT2/HNF4A/APOA4/GCLC/GPT/HGD/PAH/PPARGC1A/ABCC2/APOA5/NQO1/CES1/UGDH/GSTA1/KIT/UGT2A3/UGT2B11/UGT2B4/AKR1C4/CYP26A1/AKR1C2/APOC3/CBR1/TH/AKR1C1/UGT1A3/AKR1C3/UGT1A1/UGT1A4/UGT1A6/UGT1A7/UGT1A8/CYP1A1 | 48 | BP |
| GO:0030199 | collagen fibril organization | 9/397 | 5.16E-07 | 3.48E-05 | 2.71E-05 | MMP11/COL12A1/GREM1/COMP/COL5A1/COL11A1/COL2A1/COL1A2/COL1A1 | 9 | BP |
| GO:0006082 | organic acid metabolic process | 51/397 | 5.83E-07 | 3.88E-05 | 3.02E-05 | ACOT12/CEL/FADS6/HAO1/IYD/KERA/OMD/ALDH1A3/ANGPTL3/ENO3/GLDC/HAL/OGDHL/ODC1/PCK1/TPH2/DDC/G6PC/GPT2/HNF4A/APOA4/GCLC/GPT/HGD/PAH/PPARGC1A/ABCC2/APOA5/NQO1/CES1/UGDH/GSTA1/KIT/UGT2A3/UGT2B11/UGT2B4/AKR1C4/CYP26A1/AKR1C2/APOC3/CBR1/TH/AKR1C1/UGT1A3/AKR1C3/UGT1A1/UGT1A4/UGT1A6/UGT1A7/UGT1A8/CYP1A1 | 51 | BP |
| GO:0030638 | polyketide metabolic process | 5/397 | 6.11E-07 | 3.90E-05 | 3.04E-05 | AKR1B10/AKR1C4/AKR1C2/AKR1C1/AKR1C3 | 5 | BP |
| GO:0044597 | daunorubicin metabolic process | 5/397 | 6.11E-07 | 3.90E-05 | 3.04E-05 | AKR1B10/AKR1C4/AKR1C2/AKR1C1/AKR1C3 | 5 | BP |
| GO:0044598 | doxorubicin metabolic process | 5/397 | 6.11E-07 | 3.90E-05 | 3.04E-05 | AKR1B10/AKR1C4/AKR1C2/AKR1C1/AKR1C3 | 5 | BP |
| GO:0031667 | response to nutrient levels | 29/397 | 6.23E-07 | 3.92E-05 | 3.06E-05 | SLC6A19/SPX/SSTR1/TNC/VGF/AOC1/CASR/FOXA3/F7/G6PD/PCK1/SNAI2/TPH2/FGF21/G6PC/ALDH3A1/GCLC/PPARGC1A/KRT20/PTH/ASCL1/NQO1/CYP26A1/TH/COL1A1/SST/AKR1C3/UGT1A1/CYP1A1 | 29 | BP |
| GO:0008284 | positive regulation of cell proliferation | 45/397 | 7.54E-07 | 4.59E-05 | 3.58E-05 | CCKBR/GLP2R/LHX1/PAX7/PTGFR/TNC/AQP1/BMP6/CASR/CGA/ELANE/EREG/LHX2/NKX2-5/PRAME/S100B/TDGF1/TP63/CHRNB2/CSF2/FGFBP1/HMGA2/INSM1/LIN28A/ODC1/THPO/FGF21/GREM1/KLB/SCG2/ALDH3A1/FGF16/FGF5/GDNF/PPARGC1A/WNT7A/FGF4/PTH/CDX2/ASCL1/AKR1C2/TAC1/ITGB3/AKR1C3/F2 | 45 | BP |
| GO:0031016 | pancreas development | 11/397 | 7.59E-07 | 4.59E-05 | 3.58E-05 | NKX3-2/ZIC3/BMP6/INSM1/PTF1A/HNF4A/RFX6/PAX4/HNF1A/NEUROD1/NEUROG3 | 11 | BP |
| GO:1900046 | regulation of hemostasis | 11/397 | 7.59E-07 | 4.59E-05 | 3.58E-05 | F7/PLAT/PLAU/COMP/PROC/VTN/FGB/KNG1/FGG/F2/FGA | 11 | BP |
| GO:0046873 | metal ion transmembrane transporter activity | 23/372 | 1.63E-06 | 4.62E-05 | 3.54E-05 | GRIK3/KCNC2/KCNK2/SCN3A/SLC10A2/SLC17A2/SLC5A5/SLC6A19/TRPM8/AQP1/KCNC1/KCNK3/KCNK9/ABCC8/KCNJ18/KCNK10/CACNA1S/TF/CHRNA9/KCNQ2/KCNQ5/GRIN2A/SNAP25 | 23 | MF |
| GO:0031406 | carboxylic acid binding | 16/372 | 1.71E-06 | 4.68E-05 | 3.59E-05 | LCN12/MAG/CASR/PCK1/DDC/HAPLN1/HNF4A/GCLC/CYP26A1/AKR1C2/TH/AKR1C1/UGT1A3/UGT1A1/UGT1A7/UGT1A8 | 16 | MF |
| GO:0009636 | response to toxic substance | 30/397 | 8.19E-07 | 4.89E-05 | 3.81E-05 | CLDN18/GUCY2C/IYD/KCNC2/PPP1R1B/TNC/AQP1/KCNC1/CHRNA3/CHRNA5/CHRNB2/G6PD/NEFL/SRXN1/DDC/TXNRD1/APOA4/PPARGC1A/ABCC2/PTH/AKR1B10/GRIN2A/GPX2/NQO1/CES1/GSTA1/TH/COL1A1/UGT1A1/CYP1A1 | 30 | BP |
| GO:0022890 | inorganic cation transmembrane transporter activity | 27/372 | 1.89E-06 | 4.96E-05 | 3.80E-05 | GRIK3/KCNC2/KCNK2/SCN3A/SLC10A2/SLC17A2/SLC5A5/SLC6A19/TRPM8/ANO1/AQP1/KCNC1/KCNK3/KCNK9/ABCC8/CHRNA3/CHRNA5/CHRNB2/KCNJ18/KCNK10/CACNA1S/TF/CHRNA9/KCNQ2/KCNQ5/GRIN2A/SNAP25 | 27 | MF |
| GO:0043436 | oxoacid metabolic process | 50/397 | 8.41E-07 | 4.97E-05 | 3.87E-05 | ACOT12/CEL/FADS6/HAO1/IYD/KERA/OMD/ALDH1A3/ANGPTL3/ENO3/GLDC/HAL/OGDHL/ODC1/PCK1/TPH2/DDC/GPT2/HNF4A/APOA4/GCLC/GPT/HGD/PAH/PPARGC1A/ABCC2/APOA5/NQO1/CES1/UGDH/GSTA1/KIT/UGT2A3/UGT2B11/UGT2B4/AKR1C4/CYP26A1/AKR1C2/APOC3/CBR1/TH/AKR1C1/UGT1A3/AKR1C3/UGT1A1/UGT1A4/UGT1A6/UGT1A7/UGT1A8/CYP1A1 | 50 | BP |
| GO:0043177 | organic acid binding | 16/372 | 1.99E-06 | 5.04E-05 | 3.86E-05 | LCN12/MAG/CASR/PCK1/DDC/HAPLN1/HNF4A/GCLC/CYP26A1/AKR1C2/TH/AKR1C1/UGT1A3/UGT1A1/UGT1A7/UGT1A8 | 16 | MF |
| GO:0008202 | steroid metabolic process | 23/397 | 8.66E-07 | 5.05E-05 | 3.93E-05 | CEL/NR0B1/ANGPTL3/BMP6/CGA/APOF/G6PD/SNAI2/G6PC/APOA4/PPARGC1A/GAL/CES1/KIT/UGT2B11/AFP/AKR1C4/AKR1C2/AKR1C1/AKR1C3/UGT1A1/UGT1A8/CYP1A1 | 23 | BP |
| GO:0010035 | response to inorganic substance | 31/397 | 8.85E-07 | 5.10E-05 | 3.97E-05 | ADGRV1/KCNC2/AOC1/AQP1/BMP6/CASR/KCNC1/KCNK3/CSF2/G6PD/NEFL/TPH2/TF/APOA4/GCLC/KRT14/MMP3/PPARGC1A/TFF1/ABCC2/PTH/ASCL1/NQO1/KIT/FGB/TH/COL1A1/FGG/AKR1C3/CYP1A1/FGA | 31 | BP |
| GO:0030216 | keratinocyte differentiation | 22/397 | 9.12E-07 | 5.19E-05 | 4.04E-05 | DSC3/EREG/TP63/KRT5/KRT6B/KRT14/LCE3A/S100A7/WNT16/KRT20/DSG3/IVL/SPRR2A/SPRR2F/SPRR2G/SPRR2B/SPRR2E/SPRR2D/SPRR3/SPRR1A/SPRR1B/AKR1C3 | 22 | BP |
| GO:0001972 | retinoic acid binding | 6/372 | 2.27E-06 | 5.57E-05 | 4.27E-05 | LCN12/CYP26A1/UGT1A3/UGT1A1/UGT1A7/UGT1A8 | 6 | MF |
| GO:0030020 | extracellular matrix structural constituent conferring tensile strength | 8/372 | 2.51E-06 | 5.95E-05 | 4.56E-05 | COL8A2/COL12A1/COL10A1/COL5A1/COL11A1/COL2A1/COL1A2/COL1A1 | 8 | MF |
| GO:0005583 | fibrillar collagen trimer | 5/403 | 1.90E-06 | 5.99E-05 | 4.68E-05 | COL5A1/COL11A1/COL2A1/COL1A2/COL1A1 | 5 | CC |
| GO:0098643 | banded collagen fibril | 5/403 | 1.90E-06 | 5.99E-05 | 4.68E-05 | COL5A1/COL11A1/COL2A1/COL1A2/COL1A1 | 5 | CC |
| GO:0071363 | cellular response to growth factor stimulus | 36/397 | 1.09E-06 | 6.05E-05 | 4.72E-05 | LHX1/TNMD/BMP6/CASR/CILP/NKX2-5/SFRP4/SH3GL2/TDGF1/FGFBP1/GREM2/SNAI2/DDC/FGF21/GDF5/GREM1/KLB/FGF16/FGF5/GCLC/OTX2/PHOX2B/PPARGC1A/WNT7A/COMP/FGF4/IBSP/KL/ASPN/COL2A1/COL1A2/VTN/TAC1/TH/COL1A1/ITGB3 | 36 | BP |
| GO:0001501 | skeletal system development | 29/397 | 1.09E-06 | 6.05E-05 | 4.72E-05 | CLDN18/HAND1/INHA/LHX1/NKX3-2/PAX7/BMP6/SFRP4/TP63/HMGA2/MATN3/SNAI2/TGFBI/THPO/CDH11/GDF5/GREM1/HAND2/MMP13/WNT7A/COMP/FGF4/PTH/COL10A1/COL11A1/COL2A1/KIT/COL1A2/COL1A1 | 29 | BP |
| GO:0030195 | negative regulation of blood coagulation | 9/397 | 1.11E-06 | 6.08E-05 | 4.73E-05 | PLAT/PLAU/PROC/VTN/FGB/KNG1/FGG/F2/FGA | 9 | BP |
| GO:0016137 | glycoside metabolic process | 6/397 | 1.15E-06 | 6.13E-05 | 4.78E-05 | AKR1B10/AKR1C4/AKR1C2/TH/AKR1C1/AKR1C3 | 6 | BP |
| GO:0052696 | flavonoid glucuronidation | 4/397 | 1.17E-06 | 6.13E-05 | 4.78E-05 | UGT1A3/UGT1A1/UGT1A7/UGT1A8 | 4 | BP |
| GO:0071798 | response to prostaglandin D | 4/397 | 1.17E-06 | 6.13E-05 | 4.78E-05 | PTGFR/TNC/AKR1C2/AKR1C3 | 4 | BP |
| GO:0071799 | cellular response to prostaglandin D stimulus | 4/397 | 1.17E-06 | 6.13E-05 | 4.78E-05 | PTGFR/TNC/AKR1C2/AKR1C3 | 4 | BP |
| GO:0030647 | aminoglycoside antibiotic metabolic process | 5/397 | 1.20E-06 | 6.23E-05 | 4.85E-05 | AKR1B10/AKR1C4/AKR1C2/AKR1C1/AKR1C3 | 5 | BP |
| GO:0048598 | embryonic morphogenesis | 32/397 | 1.28E-06 | 6.57E-05 | 5.11E-05 | HAND1/LHX1/NKX3-2/ZIC3/ALDH1A3/LHX2/NKX2-5/RET/TP63/FOXN4/GREM2/HMGA2/COL12A1/GDF5/GREM1/HAND2/HNF4A/TXNRD1/CHRNA9/GDNF/OTX2/WNT16/WNT7A/FGF4/COL5A1/NEUROD1/UGDH/COL11A1/COL2A1/VTN/TH/ITGB3 | 32 | BP |
| GO:0009653 | anatomical structure morphogenesis | 91/397 | 1.43E-06 | 7.25E-05 | 5.65E-05 | CTNND2/DPYSL5/EMX2/HAND1/LHX1/MAG/MYPN/NKX3-2/ODAPH/PAX7/SRPX2/TNC/TNMD/ZIC3/ALDH1A3/ANGPTL3/AQP1/BMP6/CASR/CCKAR/COL8A2/ENAM/EREG/GNAT1/KIF5C/KLK3/LHX2/LMX1A/NKX2-5/RET/S100B/SFRP4/SH3GL2/SOX1/SYT4/TDGF1/TP63/CHRNA3/CHRNB2/FGFBP1/FGL1/FOXN4/GREM2/HMGA2/NEFL/PHOX2A/PTF1A/SNAI2/TGFBI/CDH11/COL12A1/GDF5/GREM1/HAND2/HNF4A/MMP13/SCG2/TXNRD1/CHRNA9/DMP1/FGF16/GDNF/OTX2/PHOX2B/PPARGC1A/S100A7/WNT16/WNT7A/COMP/FGF4/PAX4/CDX2/ASPN/COL5A1/GAL/NEUROD1/NEUROG3/THBS2/UGDH/COL11A1/COL2A1/KIT/COL1A2/VTN/FGB/TH/COL1A1/ITGB3/FGG/F2/FGA | 91 | BP |
| GO:0002062 | chondrocyte differentiation | 12/397 | 1.56E-06 | 7.82E-05 | 6.09E-05 | NKX3-2/BMP6/HMGA2/SNAI2/TGFBI/GDF5/GREM1/WNT7A/COMP/PTH/COL11A1/COL2A1 | 12 | BP |
| GO:0042493 | response to drug | 45/397 | 1.63E-06 | 8.11E-05 | 6.32E-05 | CLDN18/EMX2/GALR1/KCNC2/PPP1R1B/RHAG/SPINK4/TNC/AOC1/AQP1/ENO3/GABRG3/KCNC1/KCNK3/RET/CHRNA3/CHRNA5/CHRNB2/F7/G6PD/HMGA2/NEFL/SLC18A3/CACNA1S/DDC/FGF21/ALDH3A1/GCLC/GDNF/MMP3/PPARGC1A/ABCC2/PAX4/PTH/GRIN2A/ASCL1/GAL/NEUROD1/NQO1/TAC1/TH/COL1A1/SST/UGT1A1/CYP1A1 | 45 | BP |
| GO:0031214 | biomineral tissue development | 15/397 | 1.76E-06 | 8.65E-05 | 6.74E-05 | ADGRV1/ODAPH/OMD/BMP6/ENAM/GREM1/MMP13/DMP1/COMP/IBSP/KL/PTH/ASPN/COL1A2/COL1A1 | 15 | BP |
| GO:0008283 | cell proliferation | 75/397 | 1.81E-06 | 8.80E-05 | 6.86E-05 | CCKBR/EMX2/GLP2R/GUCY2C/IGFBP6/INHA/KCNK2/PAX7/PTGFR/SSTR1/TNC/AQP1/BMP6/CASR/CGA/COL8A2/ELANE/EREG/GNAT1/LHX2/NEUROD4/NKX2-5/OCA2/PRAME/S100B/SFRP4/TDGF1/TP63/UMOD/ADORA1/CHRNB2/CSF2/FGFBP1/FGL1/HMGA2/INSM1/KIF1A/LIN28A/ODC1/SNAI2/TGFBI/THPO/FGF21/GDF5/GREM1/HAND2/HNF4A/KLB/PLAU/SCG2/TXNRD1/ALDH3A1/FGF16/FGF5/GDNF/PHOX2B/PPARGC1A/TFF1/WNT16/WNT7A/COMP/FGF4/PTH/CDX2/ASCL1/GAL/KIT/VTN/AKR1C2/TAC1/ITGB3/SST/AKR1C3/F2/CYP1A1 | 75 | BP |
| GO:0098992 | neuronal dense core vesicle | 4/403 | 3.10E-06 | 9.18E-05 | 7.17E-05 | STXBP5L/SYT5/SYT4/SCG2 | 4 | CC |
| GO:0001503 | ossification | 25/397 | 2.03E-06 | 9.77E-05 | 7.61E-05 | ADGRV1/GLIS1/OMD/TNC/BMP6/CASR/TP63/SNAI2/CDH11/GDF5/GREM1/HAND2/MMP13/DMP1/COMP/IBSP/KL/PTH/ASPN/ITGA11/COL11A1/COL2A1/COL1A2/TAC1/COL1A1 | 25 | BP |
| GO:0006950 | response to stress | 124/397 | 2.11E-06 | 0.00010055 | 7.83E-05 | ANKS4B/C8G/CD207/CLEC2A/CPN1/HAO1/KCNC2/KCNK2/KLRF2/MAG/NR0B1/PAX7/PTX3/SERPINA10/SSTR1/TNC/TRPM8/VGF/WFDC12/ANO1/AOC1/AQP1/AZU1/BMP6/CASR/CXCL6/DEFB126/ELANE/ENO3/EREG/FOXA3/FZD10/GLP1R/H2AW/KCNK3/KLK3/RET/RNASE7/S100B/SPAG11B/TDGF1/TP63/UMOD/ADORA1/BPIFA1/C8A/CCL1/CFHR5/CHRNB2/CSF2/DEFA6/F7/G6PD/HMGA2/NEFL/PCK1/SNAI2/SRXN1/BPIFA2/DEFA5/FGF21/H4C1/H4C13/HAND2/HNF4A/IGFBP1/NT5E/PLAT/PLAU/PPBP/SCG2/TXNRD1/ALDH3A1/APOA4/GCLC/LCE3A/MMP3/MUC13/MUC19/MUC21/PPARGC1A/S100A7/TFF1/WNT16/WNT7A/ABCC2/APOA5/COMP/DEFB1/DEFB4A/H4C6/KRT20/ORM2/CHGA/F13B/GRIN2A/COL5A1/GAL/GPX2/MUC6/NQO1/ORM1/PROC/MUC2/MUC5AC/SERPIND1/KIT/SPRR3/AMBP/COL1A2/VTN/FGB/TAC1/KNG1/TH/COL1A1/ITGB3/FGG/SST/AKR1C3/F2/UGT1A1/CYP1A1/FGA | 124 | BP |
| GO:0006935 | chemotaxis | 31/397 | 2.16E-06 | 0.00010214 | 7.96E-05 | CCL15/DPYSL5/LHX1/PIK3C2G/AZU1/CASR/CXCL14/CXCL6/ELANE/KIF5C/LHX2/LMX1A/RET/CALCA/CCL1/CCL17/F7/GREM1/PLAU/SCG2/FGF16/GDNF/OTX2/S100A7/DEFB1/DEFB4A/FGF4/CHGA/SERPIND1/KIT/ITGB3 | 31 | BP |
| GO:0050819 | negative regulation of coagulation | 9/397 | 2.21E-06 | 0.00010322 | 8.04E-05 | PLAT/PLAU/PROC/VTN/FGB/KNG1/FGG/F2/FGA | 9 | BP |
| GO:0042330 | taxis | 31/397 | 2.33E-06 | 0.0001075 | 8.37E-05 | CCL15/DPYSL5/LHX1/PIK3C2G/AZU1/CASR/CXCL14/CXCL6/ELANE/KIF5C/LHX2/LMX1A/RET/CALCA/CCL1/CCL17/F7/GREM1/PLAU/SCG2/FGF16/GDNF/OTX2/S100A7/DEFB1/DEFB4A/FGF4/CHGA/SERPIND1/KIT/ITGB3 | 31 | BP |
| GO:0014070 | response to organic cyclic compound | 42/397 | 2.36E-06 | 0.0001075 | 8.37E-05 | CPN1/KCNC2/NR0B1/PPP1R1B/PTGFR/SLC5A5/TNC/VGF/AOC1/AQP1/BMP6/CASR/GLDC/S100B/TP63/CHRNB2/F7/G6PD/GABRA1/NEFL/PCK1/SNAI2/TPH2/CACNA1S/HNF4A/ALDH3A1/APOA4/GCLC/PPARGC1A/WNT7A/ABCC2/PAX4/PTH/ASCL1/NQO1/TAC1/TH/COL1A1/SST/AKR1C3/UGT1A1/CYP1A1 | 42 | BP |
| GO:0009991 | response to extracellular stimulus | 29/397 | 2.37E-06 | 0.0001075 | 8.37E-05 | SLC6A19/SPX/SSTR1/TNC/VGF/AOC1/CASR/FOXA3/F7/G6PD/PCK1/SNAI2/TPH2/FGF21/G6PC/ALDH3A1/GCLC/PPARGC1A/KRT20/PTH/ASCL1/NQO1/CYP26A1/TH/COL1A1/SST/AKR1C3/UGT1A1/CYP1A1 | 29 | BP |
| GO:0006811 | ion transport | 62/397 | 2.53E-06 | 0.00011369 | 8.86E-05 | CA6/CLCA2/GRIK3/KCNC2/KCNE4/KCNK2/KCNU1/LCN12/NETO1/RHAG/SCN3A/SLC10A2/SLC17A2/SLC5A5/SLC6A19/SLC7A9/SPINK1/SPX/TRPM8/ANO1/AQP1/ATP4A/CASR/KCNC1/KCNK3/KCNK9/NKX2-5/OCA2/SFRP4/SYT4/UNC80/ABCC8/ADORA1/BPIFA1/CHRNA3/CHRNB2/CSF2/G6PD/GABRA1/KCNJ18/KCNK10/NEFL/SLC18A3/SLCO1A2/CACNA1S/G6PC/TF/APOA4/CHRNA9/GDNF/KCNQ2/KCNQ5/PPARGC1A/ABCC2/PTH/GRIN2A/GAL/SNAP25/AKR1C4/APOC3/AKR1C1/F2 | 62 | BP |
| GO:0033293 | monocarboxylic acid binding | 9/372 | 5.07E-06 | 0.00011622 | 8.91E-05 | LCN12/HNF4A/CYP26A1/AKR1C2/AKR1C1/UGT1A3/UGT1A1/UGT1A7/UGT1A8 | 9 | MF |
| GO:0099503 | secretory vesicle | 42/403 | 4.29E-06 | 0.00011968 | 9.36E-05 | PTX3/RAB3C/SPX/STXBP5L/SYT5/ZG16/AOC1/AZU1/ELANE/KCNK9/PCSK2/SH3GL2/SYT4/KIF1A/PRSS2/SCG3/DDC/DEFA5/PLAT/PLAU/PPBP/SCG2/TF/TFF3/S100A7/ORM2/CHGA/GRIN2A/CALML5/ORM1/SYP/THBS2/SNAP25/KIT/TTR/FGB/KNG1/TH/COL1A1/ITGB3/FGG/FGA | 42 | CC |
| GO:0009913 | epidermal cell differentiation | 23/397 | 3.11E-06 | 0.00013846 | 0.00010785 | DSC3/EREG/SFRP4/TP63/KRT5/KRT6B/KRT14/LCE3A/S100A7/WNT16/KRT20/DSG3/IVL/SPRR2A/SPRR2F/SPRR2G/SPRR2B/SPRR2E/SPRR2D/SPRR3/SPRR1A/SPRR1B/AKR1C3 | 23 | BP |
| GO:0007268 | chemical synaptic transmission | 32/397 | 3.31E-06 | 0.00014362 | 0.00011187 | CPLX2/GRIK3/LRFN2/NETO1/STXBP5L/SYBU/SYT5/KCNK3/S100B/SYT4/ADORA1/CHRNA3/CHRNA5/CHRNB2/GABRA1/SLC18A3/CDH11/PLAT/CHAT/CHRNA9/GABBR2/GDNF/KCNQ2/WNT7A/GRIN2A/NQO1/SYP/SNAP25/KIT/TAC1/TH/SST | 32 | BP |
| GO:0098916 | anterograde trans-synaptic signaling | 32/397 | 3.31E-06 | 0.00014362 | 0.00011187 | CPLX2/GRIK3/LRFN2/NETO1/STXBP5L/SYBU/SYT5/KCNK3/S100B/SYT4/ADORA1/CHRNA3/CHRNA5/CHRNB2/GABRA1/SLC18A3/CDH11/PLAT/CHAT/CHRNA9/GABBR2/GDNF/KCNQ2/WNT7A/GRIN2A/NQO1/SYP/SNAP25/KIT/TAC1/TH/SST | 32 | BP |
| GO:0042593 | glucose homeostasis | 18/397 | 3.31E-06 | 0.00014362 | 0.00011187 | STXBP5L/SYBU/VGF/VSNL1/ANO1/CASR/FOXA3/LIN28A/PCK1/FGF21/G6PC/HNF4A/RFX6/GCLC/PPARGC1A/HNF1A/NEUROD1/TH | 18 | BP |
| GO:0051552 | flavone metabolic process | 4/397 | 3.44E-06 | 0.00014787 | 0.00011518 | PPARGC1A/UGT1A1/UGT1A7/UGT1A8 | 4 | BP |
| GO:0015850 | organic hydroxy compound transport | 18/397 | 3.53E-06 | 0.00014872 | 0.00011585 | CEL/GALR1/SLC10A2/AQP1/BMP6/SYT4/CHRNB2/SLCO1A2/APOA4/GDNF/ABCC2/CHGA/GAL/CES1/AKR1C4/APOC3/TAC1/AKR1C1 | 18 | BP |
| GO:0033500 | carbohydrate homeostasis | 18/397 | 3.53E-06 | 0.00014872 | 0.00011585 | STXBP5L/SYBU/VGF/VSNL1/ANO1/CASR/FOXA3/LIN28A/PCK1/FGF21/G6PC/HNF4A/RFX6/GCLC/PPARGC1A/HNF1A/NEUROD1/TH | 18 | BP |
| GO:0046903 | secretion | 63/397 | 3.58E-06 | 0.00014983 | 0.00011671 | CCKBR/CEL/CPLX2/GALR1/GUCA2B/INHA/KCNC2/KLRF2/PTX3/SPX/STXBP5L/SYBU/VGF/VSNL1/ANO1/AOC1/AQP1/AZU1/BMP6/CASR/ELANE/GLP1R/SYT4/ABCC8/ADORA1/CCL1/CHRNA3/CHRNA5/CHRNB2/CSF2/PRSS2/SCG3/SLC18A3/HNF4A/PLAU/PPBP/RFX6/SCG2/TF/CHAT/GDNF/S100A7/WNT7A/ABCC2/COMP/KRT20/ORM2/CHGA/CALML5/GAL/HNF1A/NEUROD1/ORM1/SYP/SNAP25/KIT/TTR/FGB/TAC1/KNG1/ITGB3/FGG/FGA | 63 | BP |
| GO:0044057 | regulation of system process | 32/397 | 3.68E-06 | 0.00015259 | 0.00011886 | GALR1/INHA/MAG/NETO1/SPX/AQP1/BMP6/CASR/GLP1R/KCNK3/NKX2-5/S100B/ADORA1/CHRNA3/FOXN4/G6PD/MYBPH/CACNA1S/HAND2/PHOX2B/PPARGC1A/WNT7A/CHGA/GRIN2A/GAL/NEUROD1/KIT/FGB/TAC1/TH/FGG/FGA | 32 | BP |
| GO:0005178 | integrin binding | 12/372 | 6.98E-06 | 0.00015494 | 0.00011876 | EDIL3/ADAM2/ANGPTL3/CASR/CDH17/TGFBI/DMP1/COMP/IBSP/COL5A1/VTN/ITGB3 | 12 | MF |
| GO:0099537 | trans-synaptic signaling | 32/397 | 3.95E-06 | 0.00016176 | 0.00012601 | CPLX2/GRIK3/LRFN2/NETO1/STXBP5L/SYBU/SYT5/KCNK3/S100B/SYT4/ADORA1/CHRNA3/CHRNA5/CHRNB2/GABRA1/SLC18A3/CDH11/PLAT/CHAT/CHRNA9/GABBR2/GDNF/KCNQ2/WNT7A/GRIN2A/NQO1/SYP/SNAP25/KIT/TAC1/TH/SST | 32 | BP |
| GO:0007186 | G protein-coupled receptor signaling pathway | 48/397 | 3.97E-06 | 0.00016176 | 0.00012601 | ADGRV1/CALCB/CCKBR/CRHR2/GALR1/GLP2R/GPR26/GRIK3/KCNK2/MLNR/NTSR2/PTGFR/PTH2R/SSTR1/UCN2/ANO1/AZU1/CASR/CCKAR/CGA/CXCL6/EREG/FZD10/GLP1R/GNAT1/GNG4/GRK1/TAC3/ADORA1/CALCA/CCL1/PPBP/GABBR2/DEFB1/DEFB4A/PTH/CHGA/GAL/SYP/NTS/GAST/AKR1C2/APOC3/TAC1/KNG1/SST/AKR1C3/F2 | 48 | BP |
| GO:0005249 | voltage-gated potassium channel activity | 9/372 | 7.73E-06 | 0.000164 | 0.0001257 | KCNC2/KCNK2/KCNC1/KCNK3/KCNK9/KCNJ18/KCNQ2/KCNQ5/SNAP25 | 9 | MF |
| GO:0004033 | aldo-keto reductase (NADP) activity | 6/372 | 7.85E-06 | 0.000164 | 0.0001257 | ALDH3A1/AKR1B10/AKR1C4/AKR1C2/AKR1C1/AKR1C3 | 6 | MF |
| GO:0042592 | homeostatic process | 67/397 | 4.13E-06 | 0.00016658 | 0.00012976 | ADGRV1/CALCB/CCKBR/CCL15/CLDN18/GALR1/INHA/PTH2R/RHAG/SPX/STXBP5L/SYBU/TRPM8/VGF/VSNL1/ANGPTL3/ANO1/AQP1/BMP6/CASR/CXCL6/ELANE/FOXA3/GLP1R/KCNK3/SFRP4/TP63/UMOD/ADORA1/BPIFA1/CALCA/CCL1/G6PD/LIN28A/PCK1/FGF21/G6PC/H4C1/H4C13/HNF4A/RFX6/TF/APOA4/CHRNA9/GCLC/MUC13/PHOX2B/PPARGC1A/ABCC2/APOA5/H4C6/KL/PTH/GRIN2A/HNF1A/MUC6/NEUROD1/MUC2/COL2A1/KIT/APOC3/TAC1/KNG1/TH/AKR1C1/ITGB3/F2 | 67 | BP |
| GO:0046887 | positive regulation of hormone secretion | 13/397 | 4.16E-06 | 0.00016658 | 0.00012976 | GALR1/INHA/SYBU/VSNL1/ANO1/BMP6/CASR/RFX6/GAL/FGB/TAC1/FGG/FGA | 13 | BP |
| GO:0042803 | protein homodimerization activity | 38/372 | 8.36E-06 | 0.00016961 | 0.00013 | CLEC2A/HAND1/KLRF2/MAG/NR0B1/TRPM8/ALDH1A3/AOC1/CASR/ENO3/GLDC/NKX2-5/S100B/SH3GL2/SYT4/CFHR5/DEFA6/G6PD/GREM2/KIF1A/ODC1/DEFA5/GREM1/HAND2/HNF4A/APOA4/GDNF/MUC13/ASCL1/KIT/AMBP/FGG/UGT1A3/UGT1A1/UGT1A4/UGT1A6/UGT1A7/UGT1A8 | 38 | MF |
| GO:0048583 | regulation of response to stimulus | 134/397 | 4.35E-06 | 0.00017279 | 0.00013459 | ADGRV1/C8G/CALCB/CCL15/CPN1/CTNND2/FCER1A/GALR1/GH2/IGFBP6/INHA/IRS4/KCNK2/NETO1/NR0B1/NRG4/PLPP4/SPINK1/SPX/SYBU/UCN2/VGF/VSNL1/ANGPTL3/ANO1/AZU1/BMP6/CASR/CD1A/CGA/CILP/CXCL14/CXCL6/DLK1/ELANE/EREG/FZD10/GNAT1/GNG4/GRK1/NKX2-5/NOTUM/PRAME/RET/S100B/SFRP4/SH3GL2/TAF1L/TDGF1/TP63/WIF1/ADORA1/C8A/CALCA/CCL1/CCL17/CFHR5/CHRNA3/CHRNB2/CSF2/F7/FGFBP1/FGL1/G6PD/GREM2/HMGA2/LIN28A/NEFL/SNAI2/THPO/CHGB/FGF21/GDF5/GREM1/HAND2/IGFBP1/KLB/MAGEA1/NT5E/PLAT/PLAU/PPBP/RFX6/SCG2/CHRNA9/FGF16/FGF5/GCLC/GDNF/MMP3/MUC13/MUC19/MUC21/OTX2/PPARGC1A/S100A7/TFF1/WNT16/WNT7A/FGF4/KL/PTH/CHGA/GRIN2A/ASCL1/ASPN/GAL/MUC6/NEUROD1/PROC/SYP/MUC2/MUC5AC/NTS/COL2A1/GAST/KIT/TTR/AMBP/COL1A2/CYP26A1/VTN/AKR1C2/APOC3/FGB/TAC1/KNG1/COL1A1/ITGB3/FGG/SST/AKR1C3/F2/FGA | 134 | BP |
| GO:0005501 | retinoid binding | 7/372 | 9.11E-06 | 0.00017977 | 0.00013779 | C8G/LCN12/CYP26A1/UGT1A3/UGT1A1/UGT1A7/UGT1A8 | 7 | MF |
| GO:0005244 | voltage-gated ion channel activity | 14/372 | 1.02E-05 | 0.00018107 | 0.00013879 | KCNC2/KCNK2/SCN3A/ANO1/KCNC1/KCNK3/KCNK9/KCNJ18/KCNK10/CACNA1S/KCNQ2/KCNQ5/GRIN2A/SNAP25 | 14 | MF |
| GO:0022832 | voltage-gated channel activity | 14/372 | 1.02E-05 | 0.00018107 | 0.00013879 | KCNC2/KCNK2/SCN3A/ANO1/KCNC1/KCNK3/KCNK9/KCNJ18/KCNK10/CACNA1S/KCNQ2/KCNQ5/GRIN2A/SNAP25 | 14 | MF |
| GO:0018636 | phenanthrene 9,10-monooxygenase activity | 3/372 | 1.05E-05 | 0.00018107 | 0.00013879 | AKR1C2/AKR1C1/AKR1C3 | 3 | MF |
| GO:0047086 | ketosteroid monooxygenase activity | 3/372 | 1.05E-05 | 0.00018107 | 0.00013879 | AKR1C2/AKR1C1/AKR1C3 | 3 | MF |
| GO:0047718 | indanol dehydrogenase activity | 3/372 | 1.05E-05 | 0.00018107 | 0.00013879 | AKR1B10/AKR1C1/AKR1C3 | 3 | MF |
| GO:0099536 | synaptic signaling | 32/397 | 4.70E-06 | 0.00018482 | 0.00014396 | CPLX2/GRIK3/LRFN2/NETO1/STXBP5L/SYBU/SYT5/KCNK3/S100B/SYT4/ADORA1/CHRNA3/CHRNA5/CHRNB2/GABRA1/SLC18A3/CDH11/PLAT/CHAT/CHRNA9/GABBR2/GDNF/KCNQ2/WNT7A/GRIN2A/NQO1/SYP/SNAP25/KIT/TAC1/TH/SST | 32 | BP |
| GO:0048483 | autonomic nervous system development | 8/397 | 4.79E-06 | 0.00018631 | 0.00014512 | RET/TP63/INSM1/PHOX2A/HAND2/GDNF/PHOX2B/ASCL1 | 8 | BP |
| GO:0051049 | regulation of transport | 68/397 | 4.81E-06 | 0.00018631 | 0.00014512 | CPLX2/GALR1/INHA/KCNC2/KCNU1/NETO1/PTX3/SCN3A/SPINK1/SPX/STXBP5L/SYBU/VSNL1/ANO1/AQP1/AZU1/BMP6/CASR/GLP1R/KCNC1/NKX2-5/SFRP4/SH3GL2/SYT4/TP63/ABCC8/ADORA1/BPIFA1/CCL1/CHRNA3/CHRNA5/CHRNB2/G6PD/H1-1/KCNJ18/KCNK10/NEFL/CACNA1S/FGF21/GREM1/HNF4A/RFX6/TF/APOA4/GDNF/KCNQ2/KCNQ5/PPARGC1A/WNT7A/APOA5/KRT20/ORM2/PTH/CHGA/GRIN2A/GAL/NEUROD1/ORM1/SYP/SNAP25/VTN/APOC3/FGB/TAC1/ITGB3/FGG/F2/FGA | 68 | BP |
| GO:0019840 | isoprenoid binding | 7/372 | 1.11E-05 | 0.0001877 | 0.00014387 | C8G/LCN12/CYP26A1/UGT1A3/UGT1A1/UGT1A7/UGT1A8 | 7 | MF |
| GO:0051239 | regulation of multicellular organismal process | 105/397 | 5.09E-06 | 0.0001953 | 0.00015213 | ADGRV1/CLDN18/GALR1/INHA/KCNK2/LHX1/MAG/NETO1/NKX3-2/ODAPH/OMD/PTH2R/SPX/SRPX2/SRY/TRPM8/ANGPTL3/AQP1/AZU1/BMP6/CASR/CXCL6/ELANE/EREG/GLP1R/KCNK3/KLK3/LHX2/LMX1A/NKX2-5/RET/S100B/SFRP4/SOX3/SYT4/TDGF1/TP63/ADORA1/CALCA/CCL1/CHRNA3/CHRNB2/CSF2/F7/FGFBP1/FOXN4/G6PD/HMGA2/LIN28A/MYBPH/NEFL/PHOX2A/PTF1A/SNAI2/THPO/CACNA1S/FGF21/GDF5/GREM1/H4C1/H4C13/HAND2/HNF4A/PLAT/PLAU/APOA4/DMP1/FGF16/GDNF/OTX2/PHOX2B/PPARGC1A/WNT7A/APOA5/COMP/FGF4/H4C6/KL/ORM2/PTH/CDX2/CHGA/GRIN2A/ASCL1/ASPN/COL5A1/GAL/NEUROD1/NEUROG3/ORM1/PROC/THBS2/SNAP25/KIT/VTN/APOC3/FGB/TAC1/KNG1/TH/COL1A1/ITGB3/FGG/F2/FGA | 105 | BP |
| GO:0030193 | regulation of blood coagulation | 10/397 | 5.19E-06 | 0.00019773 | 0.00015402 | F7/PLAT/PLAU/PROC/VTN/FGB/KNG1/FGG/F2/FGA | 10 | BP |
| GO:0008106 | alcohol dehydrogenase (NADP+) activity | 5/372 | 1.24E-05 | 0.00020404 | 0.0001564 | ALDH3A1/AKR1B10/AKR1C2/AKR1C1/AKR1C3 | 5 | MF |
| GO:0055082 | cellular chemical homeostasis | 34/397 | 5.83E-06 | 0.00022036 | 0.00017165 | CALCB/CCKBR/CCL15/GALR1/RHAG/SYBU/TRPM8/VSNL1/ANO1/BMP6/CASR/ELANE/FOXA3/GLP1R/KCNK3/ADORA1/CALCA/CCL1/LIN28A/PCK1/FGF21/RFX6/TF/CHRNA9/GCLC/PPARGC1A/ABCC2/PTH/GRIN2A/NEUROD1/TAC1/KNG1/TH/F2 | 34 | BP |
| GO:0001758 | retinal dehydrogenase activity | 4/372 | 1.48E-05 | 0.00023622 | 0.00018106 | ALDH1A3/AKR1B10/AKR1C4/AKR1C3 | 4 | MF |
| GO:0046982 | protein heterodimerization activity | 27/372 | 1.50E-05 | 0.00023622 | 0.00018106 | H2BW1/HAND1/INHA/SYT5/BMP6/ENO3/H2AW/NKX2-5/SYT4/ADORA1/CFHR5/CHRNB2/NEFL/H4C1/H4C13/HAND2/GABBR2/GCLC/H4C6/NEUROD1/TTR/UGT1A3/UGT1A1/UGT1A4/UGT1A6/UGT1A7/UGT1A8 | 27 | MF |
| GO:0031640 | killing of cells of other organism | 9/397 | 6.42E-06 | 0.00023883 | 0.00018603 | AZU1/CXCL6/ELANE/DEFA6/DEFA5/LCE3A/DEFB4A/CHGA/F2 | 9 | BP |
| GO:0044364 | disruption of cells of other organism | 9/397 | 6.42E-06 | 0.00023883 | 0.00018603 | AZU1/CXCL6/ELANE/DEFA6/DEFA5/LCE3A/DEFB4A/CHGA/F2 | 9 | BP |
| GO:0044297 | cell body | 28/403 | 1.01E-05 | 0.00026726 | 0.00020892 | CTNND2/DPYSL5/GRIK3/INHA/KCNC2/KCNK2/PPP1R1B/SYT5/CASR/GNAT1/KCNC1/KIF5C/PCSK2/RET/S100B/SH3GL2/SYT4/ADORA1/CHRNA3/KIF1A/DDC/PPARGC1A/ASCL1/GAL/NQO1/TAC1/TH/SST | 28 | CC |
| GO:0030072 | peptide hormone secretion | 18/397 | 7.65E-06 | 0.00028213 | 0.00021976 | KCNC2/STXBP5L/SYBU/VGF/VSNL1/ANO1/CASR/GLP1R/ABCC8/HNF4A/RFX6/GAL/HNF1A/NEUROD1/SNAP25/FGB/FGG/FGA | 18 | BP |
| GO:0022857 | transmembrane transporter activity | 38/372 | 1.84E-05 | 0.00028434 | 0.00021794 | CLCA2/GRIK3/KCNC2/KCNK2/RHAG/SCN3A/SLC10A2/SLC17A2/SLC5A5/SLC6A19/SLC7A9/TRPM8/ANO1/AQP1/KCNC1/KCNK3/KCNK9/OCA2/ABCC8/CDH17/CHRNA3/CHRNA5/CHRNB2/GABRA1/KCNJ18/KCNK10/SLC18A3/SLCO1A2/CACNA1S/PPBP/TF/CHRNA9/KCNQ2/KCNQ5/ABCC2/GRIN2A/SNAP25/AKR1C4 | 38 | MF |
| GO:0035989 | tendon development | 4/397 | 7.89E-06 | 0.00028464 | 0.00022172 | TNMD/COMP/COL5A1/COL11A1 | 4 | BP |
| GO:0070944 | neutrophil mediated killing of bacterium | 4/397 | 7.89E-06 | 0.00028464 | 0.00022172 | AZU1/CXCL6/ELANE/F2 | 4 | BP |
| GO:2000848 | positive regulation of corticosteroid hormone secretion | 4/397 | 7.89E-06 | 0.00028464 | 0.00022172 | GALR1/BMP6/GAL/TAC1 | 4 | BP |
| GO:0031045 | dense core granule | 5/403 | 1.15E-05 | 0.00028612 | 0.00022366 | SPX/STXBP5L/SYT5/SYT4/SCG2 | 5 | CC |
| GO:0022843 | voltage-gated cation channel activity | 11/372 | 2.10E-05 | 0.00031541 | 0.00024176 | KCNC2/KCNK2/KCNC1/KCNK3/KCNK9/KCNJ18/CACNA1S/KCNQ2/KCNQ5/GRIN2A/SNAP25 | 11 | MF |
| GO:0005215 | transporter activity | 40/372 | 2.13E-05 | 0.00031541 | 0.00024176 | CLCA2/GRIK3/KCNC2/KCNK2/RHAG/SCN3A/SLC10A2/SLC17A2/SLC5A5/SLC6A19/SLC7A9/TRPM8/ANO1/AQP1/KCNC1/KCNK3/KCNK9/OCA2/ABCC8/APOF/CDH17/CHRNA3/CHRNA5/CHRNB2/GABRA1/KCNJ18/KCNK10/SLC18A3/SLCO1A2/CACNA1S/PPBP/TF/APOA4/CHRNA9/KCNQ2/KCNQ5/ABCC2/GRIN2A/SNAP25/AKR1C4 | 40 | MF |
| GO:0018958 | phenol-containing compound metabolic process | 11/397 | 9.01E-06 | 0.00032244 | 0.00025116 | IYD/SLC5A5/OCA2/CHRNB2/INSM1/TPH2/DDC/HAND2/PAH/GRIN2A/TH | 11 | BP |
| GO:0005577 | fibrinogen complex | 4/403 | 1.40E-05 | 0.00033094 | 0.0002587 | FGL1/FGB/FGG/FGA | 4 | CC |
| GO:0050818 | regulation of coagulation | 10/397 | 9.44E-06 | 0.00033506 | 0.00026099 | F7/PLAT/PLAU/PROC/VTN/FGB/KNG1/FGG/F2/FGA | 10 | BP |
| GO:0030900 | forebrain development | 23/397 | 9.81E-06 | 0.0003453 | 0.00026897 | EMX2/KCNC2/LHX1/NR0B1/SSTR1/ALDH1A3/AQP1/CCKAR/KCNC1/LHX2/LMX1A/SOX1/SOX3/CHRNB2/KIF1A/NEFL/OTX2/PPARGC1A/WNT7A/ASCL1/NEUROD1/NEUROG3/TH | 23 | BP |
| GO:0008544 | epidermis development | 26/397 | 9.87E-06 | 0.0003453 | 0.00026897 | DSC3/EREG/LHX2/SFRP4/TP63/KRT5/KRT6B/KRT14/LCE3A/S100A7/WNT16/KRT20/CALML5/DSG3/GAL/IVL/SPRR2A/SPRR2F/SPRR2G/SPRR2B/SPRR2E/SPRR2D/SPRR3/SPRR1A/SPRR1B/AKR1C3 | 26 | BP |
| GO:0015077 | monovalent inorganic cation transmembrane transporter activity | 18/372 | 2.49E-05 | 0.00036053 | 0.00027634 | GRIK3/KCNC2/KCNK2/SCN3A/SLC10A2/SLC17A2/SLC5A5/SLC6A19/AQP1/KCNC1/KCNK3/KCNK9/ABCC8/KCNJ18/KCNK10/KCNQ2/KCNQ5/SNAP25 | 18 | MF |
| GO:0003358 | noradrenergic neuron development | 3/397 | 1.08E-05 | 0.00037271 | 0.00029032 | INSM1/PHOX2B/ASCL1 | 3 | BP |
| GO:1904640 | response to methionine | 3/397 | 1.08E-05 | 0.00037271 | 0.00029032 | PCK1/FGF21/PPARGC1A | 3 | BP |
| GO:0051707 | response to other organism | 41/397 | 1.11E-05 | 0.00037648 | 0.00029325 | CD207/GKN2/PTGFR/PTX3/REG4/SLC10A2/VGF/WFDC12/AZU1/BMP6/BPIFB2/CXCL6/DEFB126/ELANE/KLK3/RNASE7/SPAG11B/BPIFA1/CSF2/DEFA6/HMGA2/ODC1/PCK1/PRSS2/BPIFA2/DEFA5/PPBP/LCE3A/PPARGC1A/S100A7/ABCC2/DEFB1/DEFB4A/CHGA/FGB/TAC1/TH/F2/UGT1A1/CYP1A1/FGA | 41 | BP |
| GO:0031424 | keratinization | 17/397 | 1.11E-05 | 0.00037648 | 0.00029325 | DSC3/KRT5/KRT6B/KRT14/LCE3A/KRT20/DSG3/IVL/SPRR2A/SPRR2F/SPRR2G/SPRR2B/SPRR2E/SPRR2D/SPRR3/SPRR1A/SPRR1B | 17 | BP |
| GO:0043207 | response to external biotic stimulus | 41/397 | 1.17E-05 | 0.00039315 | 0.00030624 | CD207/GKN2/PTGFR/PTX3/REG4/SLC10A2/VGF/WFDC12/AZU1/BMP6/BPIFB2/CXCL6/DEFB126/ELANE/KLK3/RNASE7/SPAG11B/BPIFA1/CSF2/DEFA6/HMGA2/ODC1/PCK1/PRSS2/BPIFA2/DEFA5/PPBP/LCE3A/PPARGC1A/S100A7/ABCC2/DEFB1/DEFB4A/CHGA/FGB/TAC1/TH/F2/UGT1A1/CYP1A1/FGA | 41 | BP |
| GO:0012505 | endomembrane system | 127/403 | 1.78E-05 | 0.00039892 | 0.00031184 | ANKS4B/B4GALNT2/CD207/CLCA2/CLGN/GH2/GUCY2C/H2BW1/IGFBP6/KCNK2/KERA/MTMR7/NMNAT2/OMD/PGA5/PTX3/RAB3C/SERPINA10/SPX/STXBP5L/SYBU/SYT5/TNC/TNMD/TRPM8/VGF/ZG16/ANGPTL3/AOC1/AQP1/AZU1/BPIFB2/CD1A/CGA/CLDN8/COL8A2/CXCL14/ELANE/ENAM/KCNK9/NOTUM/OCA2/PCSK2/RET/SH3GL2/SYT4/TP63/UMOD/ADORA1/DEFA6/F7/KIF1A/LIN28A/MATN3/MMP11/PRSS2/SCG3/SLC18A3/TGFBI/CHGB/COL12A1/DDC/DEFA5/G6PC/IGFBP1/PLAT/PLAU/PPBP/SCG2/TF/TFF3/ALDH3A1/APOA4/DMP1/MUC13/MUC19/MUC21/S100A7/WNT7A/APOA5/DEFB1/DEFB4A/ORM2/CHGA/COL10A1/GRIN2A/CALML5/COL5A1/GAL/MUC6/ORM1/PROC/SYP/THBS2/CES1/MUC2/MUC5AC/NTS/SERPIND1/SNAP25/COL11A1/COL2A1/KIT/SPRR3/TTR/UGT2B11/UGT2B4/AFP/COL1A2/CYP26A1/VTN/APOC3/FGB/KNG1/TH/COL1A1/ITGB3/FGG/UGT1A3/F2/UGT1A1/UGT1A4/UGT1A6/UGT1A7/UGT1A8/CYP1A1/FGA | 127 | CC |
| GO:0072562 | blood microparticle | 13/403 | 1.85E-05 | 0.00039892 | 0.00031184 | C8G/C8A/TF/APOA4/ORM2/ORM1/AMBP/VTN/FGB/KNG1/FGG/F2/FGA | 13 | CC |
| GO:0005509 | calcium ion binding | 31/372 | 2.84E-05 | 0.00040395 | 0.00030963 | ADGRV1/CDHR2/CLGN/EDIL3/S100A7A/SYT5/VSNL1/AOC1/CASR/DLK1/DSC3/MYL1/RET/S100A2/S100B/SYT4/UMOD/F7/MATN3/PRSS2/MMP13/DMP1/S100A7/COMP/ASPN/CALML3/CALML5/DSG3/PROC/THBS2/F2 | 31 | MF |
| GO:0044432 | endoplasmic reticulum part | 51/403 | 1.97E-05 | 0.00040573 | 0.00031716 | ANKS4B/CLGN/GUCY2C/KCNK2/SERPINA10/TNC/TRPM8/VGF/BPIFB2/COL8A2/ENAM/NOTUM/OCA2/F7/MATN3/SCG3/CHGB/COL12A1/G6PC/IGFBP1/SCG2/TF/APOA4/DMP1/WNT7A/APOA5/COL10A1/COL5A1/PROC/CES1/SERPIND1/COL11A1/COL2A1/UGT2B11/UGT2B4/AFP/COL1A2/CYP26A1/VTN/KNG1/COL1A1/FGG/UGT1A3/F2/UGT1A1/UGT1A4/UGT1A6/UGT1A7/UGT1A8/CYP1A1/FGA | 51 | CC |
| GO:0009986 | cell surface | 32/403 | 2.26E-05 | 0.0004317 | 0.00033746 | ADGRV1/FCER1A/KCNK2/LRFN2/SRPX2/TRPM8/ADAM2/ANGPTL3/CASR/ELANE/FZD10/KCNC1/SFRP4/TDGF1/CHRNB2/FGFBP1/H1-1/GREM1/NT5E/PLAT/PLAU/TF/APOA4/WNT7A/ABCC2/GRIN2A/KIT/FGB/ITGB3/FGG/F2/FGA | 32 | CC |
| GO:0005887 | integral component of plasma membrane | 47/403 | 2.28E-05 | 0.0004317 | 0.00033746 | C8G/CDHR2/CRHR2/FCER1A/GRIK3/IYD/KCNC2/KCNK2/KLRF2/MAG/NETO1/NTSR2/PTGFR/PTH2R/SLC10A2/SLC6A19/SLC7A9/ADAM2/AQP1/ATP4A/CASR/CD1A/EREG/FZD10/GABRG3/GLP1R/KCNC1/RET/ABCC8/ADORA1/C8A/CHRNA3/CHRNA5/CHRNB2/GABRA1/CDH9/CHRNA9/GABBR2/KCNQ2/KCNQ5/ABCC2/KL/GRIN2A/ITGA11/SNAP25/ITGB3/UGT1A1 | 47 | CC |
| GO:0045596 | negative regulation of cell differentiation | 35/397 | 1.41E-05 | 0.00047074 | 0.00036668 | ADGRV1/CLDN18/GLIS1/INHA/MAG/NKX3-2/CXCL14/EREG/LHX2/LMX1A/NKX2-5/PRAME/S100B/SOX3/SYT4/TP63/CCL17/G6PD/LIN28A/MMP11/SNAI2/GDF5/GREM1/H4C1/H4C13/HAND2/PHOX2B/WNT7A/H4C6/PTH/ASCL1/COL5A1/GAL/ITGB3/F2 | 35 | BP |
| GO:0042730 | fibrinolysis | 6/397 | 1.42E-05 | 0.00047074 | 0.00036668 | PLAT/PLAU/FGB/FGG/F2/FGA | 6 | BP |
| GO:0051046 | regulation of secretion | 36/397 | 1.47E-05 | 0.00048438 | 0.0003773 | CPLX2/GALR1/INHA/KCNC2/SPX/STXBP5L/SYBU/VSNL1/ANO1/AQP1/BMP6/CASR/GLP1R/SYT4/ABCC8/ADORA1/CCL1/CHRNA3/CHRNA5/CHRNB2/HNF4A/RFX6/GDNF/WNT7A/KRT20/ORM2/CHGA/GAL/NEUROD1/ORM1/SYP/SNAP25/FGB/TAC1/FGG/FGA | 36 | BP |
| GO:0022610 | biological adhesion | 54/397 | 1.51E-05 | 0.00049463 | 0.00038529 | ADGRV1/B4GALNT2/CDHR2/CLCA2/CLDN18/CLDN9/CTNND2/EDIL3/MAG/MYBPC1/OMD/SRPX2/TNC/ADAM2/ANGPTL3/AZU1/CLDN8/COL8A2/DSC3/ELANE/RET/UMOD/BPIFA1/CDH17/FGL1/MYBPH/PRSS2/SNAI2/TGFBI/CDH11/CDH7/COL12A1/GREM1/HAPLN1/NT5E/PLAU/APOA4/CDH9/DMP1/COMP/IBSP/COL5A1/DSG3/ITGA11/THBS2/KIT/AMBP/VTN/FGB/KNG1/COL1A1/ITGB3/FGG/FGA | 54 | BP |
| GO:0035881 | amacrine cell differentiation | 4/397 | 1.55E-05 | 0.00050184 | 0.0003909 | NEUROD4/FOXN4/PTF1A/NEUROD1 | 4 | BP |
| GO:0070943 | neutrophil mediated killing of symbiont cell | 4/397 | 1.55E-05 | 0.00050184 | 0.0003909 | AZU1/CXCL6/ELANE/F2 | 4 | BP |
| GO:0001678 | cellular glucose homeostasis | 13/397 | 1.68E-05 | 0.0005413 | 0.00042164 | SYBU/VSNL1/ANO1/CASR/FOXA3/LIN28A/PCK1/FGF21/RFX6/GCLC/PPARGC1A/NEUROD1/TH | 13 | BP |
| GO:0042127 | regulation of cell proliferation | 63/397 | 1.71E-05 | 0.00054547 | 0.00042489 | CCKBR/GLP2R/GUCY2C/IGFBP6/INHA/KCNK2/LHX1/PAX7/PTGFR/SSTR1/TNC/AQP1/BMP6/CASR/CGA/ELANE/EREG/LHX2/NKX2-5/PRAME/S100B/SFRP4/TDGF1/TP63/UMOD/ADORA1/CHRNB2/CSF2/FGFBP1/HMGA2/INSM1/LIN28A/ODC1/SNAI2/THPO/FGF21/GDF5/GREM1/HAND2/HNF4A/KLB/PLAU/SCG2/ALDH3A1/FGF16/FGF5/GDNF/PHOX2B/PPARGC1A/TFF1/WNT7A/FGF4/PTH/CDX2/ASCL1/GAL/KIT/AKR1C2/TAC1/ITGB3/SST/AKR1C3/F2 | 63 | BP |
| GO:0098644 | complex of collagen trimers | 5/403 | 3.10E-05 | 0.00056548 | 0.00044204 | COL5A1/COL11A1/COL2A1/COL1A2/COL1A1 | 5 | CC |
| GO:0047115 | trans-1,2-dihydrobenzene-1,2-diol dehydrogenase activity | 3/372 | 4.11E-05 | 0.00057278 | 0.00043903 | AKR1C2/AKR1C1/AKR1C3 | 3 | MF |
| GO:1903530 | regulation of secretion by cell | 34/397 | 1.81E-05 | 0.00057409 | 0.00044718 | CPLX2/GALR1/INHA/KCNC2/STXBP5L/SYBU/VSNL1/ANO1/BMP6/CASR/GLP1R/SYT4/ABCC8/ADORA1/CCL1/CHRNA3/CHRNA5/CHRNB2/HNF4A/RFX6/GDNF/WNT7A/KRT20/ORM2/CHGA/GAL/NEUROD1/ORM1/SYP/SNAP25/FGB/TAC1/FGG/FGA | 34 | BP |
| GO:0042448 | progesterone metabolic process | 5/397 | 1.86E-05 | 0.00058668 | 0.00045699 | PPARGC1A/AFP/AKR1C2/AKR1C1/AKR1C3 | 5 | BP |
| GO:0009887 | animal organ morphogenesis | 43/397 | 1.91E-05 | 0.00059861 | 0.00046628 | EMX2/HAND1/LHX1/NKX3-2/ODAPH/PAX7/TNC/ZIC3/ALDH1A3/AQP1/BMP6/COL8A2/EREG/GNAT1/NKX2-5/SFRP4/SOX1/TP63/FGL1/FOXN4/PTF1A/SNAI2/GREM1/HAND2/MMP13/CHRNA9/DMP1/FGF16/GDNF/WNT16/WNT7A/COMP/FGF4/PAX4/CDX2/ASPN/COL5A1/NEUROD1/COL11A1/COL2A1/COL1A2/TH/COL1A1 | 43 | BP |
| GO:0009628 | response to abiotic stimulus | 49/397 | 2.08E-05 | 0.00064764 | 0.00050447 | ADGRV1/KCNC2/KCNK2/MAG/NETO1/PPP1R1B/RGR/TNC/TRPM8/VGF/ADAM2/ANO1/AQP1/BMP6/CASR/ELANE/GNAT1/GRK1/H2AW/KCNC1/KCNK3/S100B/ADORA1/CHRNB2/F7/HMGA2/PCK1/SNAI2/GDF5/PLAT/PLAU/ALDH3A1/CHRNA9/FGF16/GCLC/KRT14/PPARGC1A/ABCC2/GRIN2A/ASCL1/IVL/NQO1/COL11A1/KIT/TAC1/TH/COL1A1/SST/CYP1A1 | 49 | BP |
| GO:0072378 | blood coagulation, fibrin clot formation | 6/397 | 2.28E-05 | 0.00069877 | 0.00054431 | F7/FGB/KNG1/FGG/F2/FGA | 6 | BP |
| GO:1901661 | quinone metabolic process | 6/397 | 2.28E-05 | 0.00069877 | 0.00054431 | AKR1B10/AKR1C4/CBR3/AKR1C2/AKR1C1/AKR1C3 | 6 | BP |
| GO:0070167 | regulation of biomineral tissue development | 10/397 | 2.48E-05 | 0.00075368 | 0.00058707 | ADGRV1/ODAPH/OMD/BMP6/GREM1/DMP1/COMP/KL/PTH/ASPN | 10 | BP |
| GO:0007417 | central nervous system development | 41/397 | 2.49E-05 | 0.00075368 | 0.00058707 | EMX2/KCNC2/LHX1/MAG/NR0B1/PAX7/SSTR1/ALDH1A3/AQP1/CCKAR/KCNC1/KCNK3/LHX2/LMX1A/S100B/SH3GL2/SOX1/SOX3/SYT4/CHRNB2/FOXN4/G6PD/KIF1A/NEFL/PHOX2A/PTF1A/CDH11/FOXG1/GDNF/OTX2/PHOX2B/PPARGC1A/WNT7A/GRIN2A/ASCL1/NEUROD1/NEUROG3/COL2A1/VTN/TH/F2 | 41 | BP |
| GO:0007584 | response to nutrient | 16/397 | 2.55E-05 | 0.00076756 | 0.00059789 | SLC6A19/TNC/CASR/F7/SNAI2/ALDH3A1/GCLC/PTH/ASCL1/NQO1/CYP26A1/COL1A1/SST/AKR1C3/UGT1A1/CYP1A1 | 16 | BP |
| GO:0007155 | cell adhesion | 53/397 | 2.64E-05 | 0.00078774 | 0.00061361 | ADGRV1/B4GALNT2/CDHR2/CLCA2/CLDN18/CLDN9/CTNND2/EDIL3/MAG/MYBPC1/OMD/SRPX2/TNC/ADAM2/ANGPTL3/AZU1/CLDN8/COL8A2/DSC3/ELANE/RET/UMOD/CDH17/FGL1/MYBPH/PRSS2/SNAI2/TGFBI/CDH11/CDH7/COL12A1/GREM1/HAPLN1/NT5E/PLAU/APOA4/CDH9/DMP1/COMP/IBSP/COL5A1/DSG3/ITGA11/THBS2/KIT/AMBP/VTN/FGB/KNG1/COL1A1/ITGB3/FGG/FGA | 53 | BP |
| GO:0009607 | response to biotic stimulus | 41/397 | 2.68E-05 | 0.00079578 | 0.00061987 | CD207/GKN2/PTGFR/PTX3/REG4/SLC10A2/VGF/WFDC12/AZU1/BMP6/BPIFB2/CXCL6/DEFB126/ELANE/KLK3/RNASE7/SPAG11B/BPIFA1/CSF2/DEFA6/HMGA2/ODC1/PCK1/PRSS2/BPIFA2/DEFA5/PPBP/LCE3A/PPARGC1A/S100A7/ABCC2/DEFB1/DEFB4A/CHGA/FGB/TAC1/TH/F2/UGT1A1/CYP1A1/FGA | 41 | BP |
| GO:2000833 | positive regulation of steroid hormone secretion | 4/397 | 2.74E-05 | 0.00080984 | 0.00063082 | GALR1/BMP6/GAL/TAC1 | 4 | BP |
| GO:0030282 | bone mineralization | 11/397 | 2.78E-05 | 0.0008155 | 0.00063523 | ADGRV1/OMD/BMP6/GREM1/MMP13/COMP/IBSP/KL/PTH/ASPN/COL1A2 | 11 | BP |
| GO:0071333 | cellular response to glucose stimulus | 12/397 | 2.80E-05 | 0.0008155 | 0.00063523 | SYBU/VSNL1/ANO1/CASR/LIN28A/PCK1/FGF21/RFX6/GCLC/PPARGC1A/NEUROD1/TH | 12 | BP |
| GO:0036122 | BMP binding | 4/372 | 6.64E-05 | 0.00087618 | 0.00067158 | GREM2/GDF5/GREM1/COMP | 4 | MF |
| GO:0048407 | platelet-derived growth factor binding | 4/372 | 6.64E-05 | 0.00087618 | 0.00067158 | COL5A1/COL2A1/COL1A2/COL1A1 | 4 | MF |
| GO:0016614 | oxidoreductase activity, acting on CH-OH group of donors | 11/372 | 6.66E-05 | 0.00087618 | 0.00067158 | HAO1/G6PD/ALDH3A1/AKR1B10/UGDH/AKR1C4/CBR3/AKR1C2/CBR1/AKR1C1/AKR1C3 | 11 | MF |
| GO:0032940 | secretion by cell | 56/397 | 3.04E-05 | 0.00088135 | 0.00068652 | CPLX2/GALR1/INHA/KCNC2/KLRF2/PTX3/STXBP5L/SYBU/VGF/VSNL1/ANO1/AOC1/AZU1/BMP6/CASR/ELANE/GLP1R/SYT4/ABCC8/ADORA1/CCL1/CHRNA3/CHRNA5/CHRNB2/PRSS2/SCG3/SLC18A3/HNF4A/PLAU/PPBP/RFX6/SCG2/TF/CHAT/GDNF/S100A7/WNT7A/COMP/KRT20/ORM2/CHGA/CALML5/GAL/HNF1A/NEUROD1/ORM1/SYP/SNAP25/KIT/TTR/FGB/TAC1/KNG1/ITGB3/FGG/FGA | 56 | BP |
| GO:0019838 | growth factor binding | 11/372 | 7.19E-05 | 0.00092802 | 0.00071132 | IGFBP6/SRPX2/FGFBP1/IGFBP1/KLB/KL/COL5A1/COL2A1/COL1A2/COL1A1/ITGB3 | 11 | MF |
| GO:0051897 | positive regulation of protein kinase B signaling | 14/397 | 3.25E-05 | 0.00093113 | 0.0007253 | NRG4/EREG/RET/F7/LIN28A/THPO/KLB/FGF16/FGF5/FGF4/KL/KIT/AKR1C2/AKR1C3 | 14 | BP |
| GO:0071331 | cellular response to hexose stimulus | 12/397 | 3.27E-05 | 0.00093113 | 0.0007253 | SYBU/VSNL1/ANO1/CASR/LIN28A/PCK1/FGF21/RFX6/GCLC/PPARGC1A/NEUROD1/TH | 12 | BP |
| GO:0061045 | negative regulation of wound healing | 9/397 | 3.27E-05 | 0.00093113 | 0.0007253 | PLAT/PLAU/PROC/VTN/FGB/KNG1/FGG/F2/FGA | 9 | BP |
| GO:0005184 | neuropeptide hormone activity | 5/372 | 7.51E-05 | 0.00095251 | 0.00073009 | CALCB/SPX/VGF/GAL/NTS | 5 | MF |
| GO:0046189 | phenol-containing compound biosynthetic process | 7/397 | 3.44E-05 | 0.00097414 | 0.0007588 | OCA2/INSM1/TPH2/DDC/HAND2/PAH/TH | 7 | BP |
| GO:1902644 | tertiary alcohol metabolic process | 5/397 | 3.52E-05 | 0.00098433 | 0.00076673 | AKR1B10/AKR1C4/AKR1C2/AKR1C1/AKR1C3 | 5 | BP |
| GO:0071326 | cellular response to monosaccharide stimulus | 12/397 | 3.52E-05 | 0.00098433 | 0.00076673 | SYBU/VSNL1/ANO1/CASR/LIN28A/PCK1/FGF21/RFX6/GCLC/PPARGC1A/NEUROD1/TH | 12 | BP |
| GO:0009611 | response to wounding | 32/397 | 3.54E-05 | 0.00098433 | 0.00076673 | KCNK2/MAG/PAX7/SERPINA10/TNC/ENO3/EREG/F7/NEFL/HNF4A/IGFBP1/PLAT/PLAU/WNT7A/APOA5/COMP/F13B/GRIN2A/COL5A1/PROC/SERPIND1/SPRR3/COL1A2/VTN/FGB/KNG1/COL1A1/ITGB3/FGG/F2/CYP1A1/FGA | 32 | BP |
| GO:0044420 | extracellular matrix component | 7/403 | 6.18E-05 | 0.00105841 | 0.00082736 | TNC/COL12A1/COL5A1/COL11A1/COL2A1/COL1A2/COL1A1 | 7 | CC |
| GO:0005892 | acetylcholine-gated channel complex | 4/403 | 6.25E-05 | 0.00105841 | 0.00082736 | CHRNA3/CHRNA5/CHRNB2/CHRNA9 | 4 | CC |
| GO:0042802 | identical protein binding | 60/372 | 8.61E-05 | 0.00107196 | 0.00082165 | CLDN18/CLDN9/CLEC2A/HAND1/KLRF2/MAG/NR0B1/PLPP4/PTX3/SRPX2/TRPM8/ALDH1A3/AOC1/AQP1/CASR/CLDN8/ENO3/GLDC/NKX2-5/S100A2/S100B/SH3GL2/SYT4/TP63/CALCA/CFHR5/DEFA6/G6PD/GREM2/KIF1A/NEFL/ODC1/DEFA5/GDF5/GREM1/HAND2/HNF4A/APOA4/GDNF/HGD/MUC13/DEFB1/ASCL1/NQO1/SYP/COL2A1/KIT/TTR/AMBP/COL1A2/VTN/COL1A1/ITGB3/FGG/UGT1A3/UGT1A1/UGT1A4/UGT1A6/UGT1A7/UGT1A8 | 60 | MF |
| GO:0007613 | memory | 11/397 | 3.91E-05 | 0.00107744 | 0.00083927 | KCNK2/NETO1/PPP1R1B/LMX1A/S100B/SYT4/CHRNB2/KCNK10/GRIN2A/TAC1/TH | 11 | BP |
| GO:0090276 | regulation of peptide hormone secretion | 15/397 | 3.92E-05 | 0.00107744 | 0.00083927 | KCNC2/STXBP5L/SYBU/VSNL1/ANO1/CASR/GLP1R/ABCC8/HNF4A/RFX6/NEUROD1/SNAP25/FGB/FGG/FGA | 15 | BP |
| GO:0031018 | endocrine pancreas development | 7/397 | 4.04E-05 | 0.00110201 | 0.0008584 | BMP6/INSM1/HNF4A/RFX6/PAX4/NEUROD1/NEUROG3 | 7 | BP |
| GO:0071310 | cellular response to organic substance | 86/397 | 4.15E-05 | 0.00112213 | 0.00087408 | CLDN18/CRHR2/GH2/GLP2R/LHX1/NR0B1/PTGFR/SLC5A5/SSTR1/SYBU/TNC/TNMD/UCN2/VSNL1/ANO1/AOC1/AQP1/BMP6/CASR/CILP/CXCL6/EREG/FZD10/GLDC/GLP1R/NKX2-5/RET/SFRP4/SH3GL2/SOX1/TDGF1/TP63/CHRNA3/CHRNB2/CSF2/FGFBP1/GABRA1/GREM2/LIN28A/PCK1/SNAI2/THPO/CACNA1S/DDC/FGF21/GDF5/GREM1/GSTA2/HNF4A/IGFBP1/KLB/RFX6/FGF16/FGF5/GCLC/MMP3/OTX2/PHOX2B/PPARGC1A/WNT7A/ABCC2/COMP/FGF4/IBSP/KL/PTH/ASPN/NEUROD1/SYP/COL2A1/KIT/AKR1C4/COL1A2/VTN/AKR1C2/FGB/TAC1/TH/AKR1C1/COL1A1/ITGB3/FGG/SST/AKR1C3/UGT1A1/CYP1A1 | 86 | BP |
| GO:0030073 | insulin secretion | 15/397 | 4.16E-05 | 0.00112213 | 0.00087408 | KCNC2/STXBP5L/SYBU/VGF/VSNL1/ANO1/CASR/GLP1R/ABCC8/HNF4A/RFX6/GAL/HNF1A/NEUROD1/SNAP25 | 15 | BP |
| GO:0070942 | neutrophil mediated cytotoxicity | 4/397 | 4.49E-05 | 0.00120556 | 0.00093906 | AZU1/CXCL6/ELANE/F2 | 4 | BP |
| GO:0009713 | catechol-containing compound biosynthetic process | 5/397 | 4.70E-05 | 0.00123576 | 0.00096259 | INSM1/DDC/HAND2/PAH/TH | 5 | BP |
| GO:0032331 | negative regulation of chondrocyte differentiation | 5/397 | 4.70E-05 | 0.00123576 | 0.00096259 | NKX3-2/SNAI2/GDF5/GREM1/PTH | 5 | BP |
| GO:0042423 | catecholamine biosynthetic process | 5/397 | 4.70E-05 | 0.00123576 | 0.00096259 | INSM1/DDC/HAND2/PAH/TH | 5 | BP |
| GO:0019731 | antibacterial humoral response | 7/397 | 4.71E-05 | 0.00123576 | 0.00096259 | ELANE/KLK3/RNASE7/BPIFA1/DEFB1/FGB/FGA | 7 | BP |
| GO:0032870 | cellular response to hormone stimulus | 31/397 | 4.95E-05 | 0.00129169 | 0.00100616 | CRHR2/GH2/GLP2R/NR0B1/PTGFR/SLC5A5/SSTR1/TNC/UCN2/AQP1/GLP1R/TP63/CHRNA3/CHRNB2/PCK1/FGF21/HNF4A/IGFBP1/GCLC/PPARGC1A/ABCC2/KL/PTH/KIT/AKR1C4/AKR1C2/FGB/AKR1C1/SST/AKR1C3/UGT1A1 | 31 | BP |
| GO:0010243 | response to organonitrogen compound | 40/397 | 5.06E-05 | 0.00131262 | 0.00102246 | CRHR2/GH2/GLP2R/KCNC2/PPP1R1B/SLC5A5/VGF/AOC1/AQP1/CASR/EREG/GLDC/GLP1R/KCNC1/CHRNB2/F7/GABRA1/NEFL/PCK1/CACNA1S/DDC/FGF21/IGFBP1/MMP13/ALDH3A1/GCLC/MMP3/PPARGC1A/TFF1/ABCC2/KL/PAX4/GRIN2A/ASCL1/GAL/COL1A2/TAC1/TH/COL1A1/SST | 40 | BP |
| GO:0030594 | neurotransmitter receptor activity | 9/372 | 0.00011044 | 0.00135198 | 0.00103629 | GRIK3/ADORA1/CHRNA3/CHRNA5/CHRNB2/GABRA1/CHRNA9/GABBR2/GRIN2A | 9 | MF |
| GO:0002067 | glandular epithelial cell differentiation | 7/397 | 5.47E-05 | 0.00141226 | 0.00110007 | BMP6/TP63/INSM1/HNF4A/RFX6/ASCL1/NEUROD1 | 7 | BP |
| GO:1901701 | cellular response to oxygen-containing compound | 44/397 | 5.50E-05 | 0.00141241 | 0.00110018 | CRHR2/GH2/GLP2R/KCNC2/PTGFR/SLC5A5/SYBU/TNC/VSNL1/ANO1/AOC1/AQP1/BMP6/CASR/CXCL6/FZD10/GLP1R/RET/CHRNA3/CHRNB2/CSF2/LIN28A/PCK1/SNAI2/FGF21/IGFBP1/RFX6/APOA4/GCLC/MMP3/PPARGC1A/ABCC2/KL/AKR1B10/NEUROD1/NQO1/AKR1C4/COL1A2/AKR1C2/TH/AKR1C1/COL1A1/AKR1C3/UGT1A1 | 44 | BP |
| GO:0034308 | primary alcohol metabolic process | 9/397 | 5.58E-05 | 0.00142385 | 0.0011091 | HAO1/ALDH1A3/BMP6/AKR1B10/TTR/AKR1C4/AKR1C2/AKR1C1/AKR1C3 | 9 | BP |
| GO:0044344 | cellular response to fibroblast growth factor stimulus | 12/397 | 5.89E-05 | 0.00149469 | 0.00116428 | LHX1/TDGF1/FGFBP1/FGF21/KLB/FGF16/FGF5/GCLC/OTX2/FGF4/KL/COL1A1 | 12 | BP |
| GO:0035821 | modification of morphology or physiology of other organism | 13/397 | 5.93E-05 | 0.00149774 | 0.00116666 | PTX3/AQP1/AZU1/CXCL6/ELANE/RNASE7/DEFA6/HMGA2/DEFA5/LCE3A/DEFB4A/CHGA/F2 | 13 | BP |
| GO:1901698 | response to nitrogen compound | 42/397 | 6.10E-05 | 0.00153157 | 0.00119301 | CRHR2/GH2/GLP2R/KCNC2/PPP1R1B/SLC5A5/VGF/AOC1/AQP1/CASR/EREG/GLDC/GLP1R/KCNC1/CHRNA3/CHRNB2/F7/GABRA1/NEFL/PCK1/CACNA1S/DDC/FGF21/IGFBP1/MMP13/ALDH3A1/GCLC/MMP3/PPARGC1A/TFF1/ABCC2/KL/PAX4/GRIN2A/ASCL1/GAL/NQO1/COL1A2/TAC1/TH/COL1A1/SST | 42 | BP |
| GO:0016616 | oxidoreductase activity, acting on the CH-OH group of donors, NAD or NADP as acceptor | 10/372 | 0.00013081 | 0.00157412 | 0.00120656 | G6PD/ALDH3A1/AKR1B10/UGDH/AKR1C4/CBR3/AKR1C2/CBR1/AKR1C1/AKR1C3 | 10 | MF |
| GO:0071322 | cellular response to carbohydrate stimulus | 12/397 | 6.32E-05 | 0.00157836 | 0.00122945 | SYBU/VSNL1/ANO1/CASR/LIN28A/PCK1/FGF21/RFX6/GCLC/PPARGC1A/NEUROD1/TH | 12 | BP |
| GO:0045202 | synapse | 43/403 | 0.00010097 | 0.00159626 | 0.0012478 | ADGRV1/CEL/GRIK3/KCNC2/KCNK2/LRFN2/NETO1/NMNAT2/PPP1R1B/RAB3C/RIMBP2/SRPX2/STXBP5L/CASR/GABRG3/KCNC1/KCNK9/KIF5C/SH3GL2/SYT4/ADORA1/CHRNA3/CHRNA5/CHRNB2/GABRA1/KIF1A/NEFL/CDH11/DDC/HAPLN1/PLAT/CDH9/CHAT/CHRNA9/GABBR2/WNT7A/GRIN2A/SYP/SNAP25/FGB/TH/ITGB3/FGA | 43 | CC |
| GO:0031226 | intrinsic component of plasma membrane | 47/403 | 0.00010103 | 0.00159626 | 0.0012478 | C8G/CDHR2/CRHR2/FCER1A/GRIK3/IYD/KCNC2/KCNK2/KLRF2/MAG/NETO1/NTSR2/PTGFR/PTH2R/SLC10A2/SLC6A19/SLC7A9/ADAM2/AQP1/ATP4A/CASR/CD1A/EREG/FZD10/GABRG3/GLP1R/KCNC1/RET/ABCC8/ADORA1/C8A/CHRNA3/CHRNA5/CHRNB2/GABRA1/CDH9/CHRNA9/GABBR2/KCNQ2/KCNQ5/ABCC2/KL/GRIN2A/ITGA11/SNAP25/ITGB3/UGT1A1 | 47 | CC |
| GO:0048857 | neural nucleus development | 8/397 | 6.45E-05 | 0.00160367 | 0.00124916 | KCNC2/ALDH1A3/KCNC1/CHRNB2/G6PD/PHOX2A/PHOX2B/ASCL1 | 8 | BP |
| GO:0034702 | ion channel complex | 16/403 | 0.00010815 | 0.0016536 | 0.00129262 | KCNC2/KCNK2/ANO1/GABRG3/KCNC1/ABCC8/CHRNA3/CHRNA5/CHRNB2/GABRA1/CACNA1S/CHRNA9/KCNQ2/KCNQ5/GRIN2A/SNAP25 | 16 | CC |
| GO:0010896 | regulation of triglyceride catabolic process | 4/397 | 6.94E-05 | 0.00170622 | 0.00132905 | FGF21/APOA4/APOA5/APOC3 | 4 | BP |
| GO:0050910 | detection of mechanical stimulus involved in sensory perception of sound | 4/397 | 6.94E-05 | 0.00170622 | 0.00132905 | ADGRV1/CHRNA9/COL11A1/KIT | 4 | BP |
| GO:0033993 | response to lipid | 38/397 | 7.42E-05 | 0.00181648 | 0.00141494 | CPN1/NR0B1/PTGFR/TNC/AQP1/BMP6/CASR/CXCL6/ELANE/FZD10/GLDC/RET/S100B/TP63/CSF2/F7/NEFL/PCK1/SNAI2/TPH2/HNF4A/ALDH3A1/PPARGC1A/S100A7/WNT7A/ABCC2/PTH/ASCL1/NQO1/CYP26A1/AKR1C2/TAC1/TH/COL1A1/SST/AKR1C3/UGT1A1/CYP1A1 | 38 | BP |
| GO:0022824 | transmitter-gated ion channel activity | 7/372 | 0.0001679 | 0.00190823 | 0.00146265 | GRIK3/CHRNA3/CHRNA5/CHRNB2/GABRA1/CHRNA9/GRIN2A | 7 | MF |
| GO:0022835 | transmitter-gated channel activity | 7/372 | 0.0001679 | 0.00190823 | 0.00146265 | GRIK3/CHRNA3/CHRNA5/CHRNB2/GABRA1/CHRNA9/GRIN2A | 7 | MF |
| GO:0004252 | serine-type endopeptidase activity | 11/372 | 0.00016821 | 0.00190823 | 0.00146265 | AZU1/ELANE/KLK3/PCSK2/F7/PRSS2/PLAT/PLAU/MMP3/PROC/F2 | 11 | MF |
| GO:0098772 | molecular function regulator | 54/372 | 0.00016932 | 0.00190823 | 0.00146265 | ADGRV1/CALCB/CCKBR/CCL15/GUCA2B/INHA/NRG4/PPP1R1B/SPINK1/SPINK4/SPX/UCN2/VGF/WFDC12/WFDC5/ANGPTL3/BMP6/CGA/CXCL14/CXCL6/TDGF1/CALCA/CCL1/CSF2/GREM2/THPO/CHGB/FGF21/GDF5/GREM1/PPBP/SCG2/APOA4/FGF16/FGF5/GDNF/TFF1/WNT7A/APOA5/FGF4/KL/PTH/GAL/NTS/SERPIND1/TTR/AMBP/APOC3/KNG1/SST/F2/UGT1A1/UGT1A7/UGT1A8 | 54 | MF |
| GO:0015721 | bile acid and bile salt transport | 5/397 | 7.92E-05 | 0.00192853 | 0.00150222 | SLC10A2/SLCO1A2/ABCC2/AKR1C4/AKR1C1 | 5 | BP |
| GO:0032879 | regulation of localization | 88/397 | 8.02E-05 | 0.00194209 | 0.00151278 | CLDN18/CPLX2/GALR1/INHA/KCNC2/KCNU1/NETO1/NRG4/PTX3/SCN3A/SPINK1/SPX/SRPX2/STXBP5L/SYBU/VSNL1/ANGPTL3/ANO1/AQP1/AZU1/BMP6/CASR/CGA/CXCL14/ELANE/EREG/GLP1R/KCNC1/NKX2-5/RET/SFRP4/SH3GL2/SYT4/TDGF1/TP63/ABCC8/ADORA1/BPIFA1/CCL1/CHRNA3/CHRNA5/CHRNB2/F7/FGFBP1/G6PD/H1-1/INSM1/KCNJ18/KCNK10/NEFL/SNAI2/CACNA1S/FGF21/GREM1/HNF4A/PLAU/RFX6/TF/APOA4/FGF16/GDNF/KCNQ2/KCNQ5/PPARGC1A/S100A7/WNT7A/APOA5/DEFB1/FGF4/KRT20/ORM2/PTH/CHGA/GRIN2A/GAL/NEUROD1/ORM1/SYP/SNAP25/VTN/APOC3/FGB/TAC1/COL1A1/ITGB3/FGG/F2/FGA | 88 | BP |
| GO:0007588 | excretion | 8/397 | 8.13E-05 | 0.00195815 | 0.00152528 | GUCA2B/SPX/UMOD/ADORA1/CHRNA3/CHRNB2/TAC1/UGT1A7 | 8 | BP |
| GO:0005104 | fibroblast growth factor receptor binding | 5/372 | 0.0001825 | 0.0020246 | 0.00155184 | FGF21/KLB/FGF16/FGF5/FGF4 | 5 | MF |
| GO:0035270 | endocrine system development | 11/397 | 8.58E-05 | 0.00205735 | 0.00160256 | NR0B1/BMP6/NKX2-5/SOX3/INSM1/HNF4A/RFX6/PAX4/ASCL1/NEUROD1/NEUROG3 | 11 | BP |
| GO:0004032 | alditol:NADP+ 1-oxidoreductase activity | 3/372 | 0.00019907 | 0.00213771 | 0.00163854 | AKR1C2/AKR1C1/AKR1C3 | 3 | MF |
| GO:0047023 | androsterone dehydrogenase activity | 3/372 | 0.00019907 | 0.00213771 | 0.00163854 | AKR1C4/AKR1C1/AKR1C3 | 3 | MF |
| GO:0015276 | ligand-gated ion channel activity | 10/372 | 0.00020474 | 0.00213771 | 0.00163854 | CLCA2/GRIK3/AQP1/CHRNA3/CHRNA5/CHRNB2/GABRA1/KCNJ18/CHRNA9/GRIN2A | 10 | MF |
| GO:0022834 | ligand-gated channel activity | 10/372 | 0.00020474 | 0.00213771 | 0.00163854 | CLCA2/GRIK3/AQP1/CHRNA3/CHRNA5/CHRNB2/GABRA1/KCNJ18/CHRNA9/GRIN2A | 10 | MF |
| GO:0031091 | platelet alpha granule | 9/403 | 0.00015272 | 0.00226223 | 0.00176839 | PPBP/ORM2/ORM1/THBS2/FGB/KNG1/ITGB3/FGG/FGA | 9 | CC |
| GO:0030141 | secretory granule | 34/403 | 0.00015936 | 0.00228893 | 0.00178926 | PTX3/SPX/STXBP5L/SYT5/ZG16/AOC1/AZU1/ELANE/PCSK2/SYT4/PRSS2/SCG3/DEFA5/PLAT/PLAU/PPBP/SCG2/TF/TFF3/S100A7/ORM2/CHGA/CALML5/ORM1/THBS2/SNAP25/KIT/TTR/FGB/KNG1/COL1A1/ITGB3/FGG/FGA | 34 | CC |
| GO:0006836 | neurotransmitter transport | 16/397 | 0.00010018 | 0.00238901 | 0.0018609 | SLC6A19/STXBP5L/AQP1/SYT4/CHRNA3/CHRNA5/CHRNB2/CSF2/SLC18A3/DDC/CHAT/GDNF/WNT7A/SYP/SNAP25/TH | 16 | BP |
| GO:0031639 | plasminogen activation | 5/397 | 0.00010066 | 0.00238901 | 0.0018609 | PLAT/PLAU/FGB/FGG/FGA | 5 | BP |
| GO:0009306 | protein secretion | 27/397 | 0.00010163 | 0.00240003 | 0.00186949 | KCNC2/KLRF2/STXBP5L/SYBU/VGF/VSNL1/ANO1/BMP6/CASR/GLP1R/SYT4/ABCC8/CCL1/HNF4A/RFX6/SCG2/COMP/KRT20/ORM2/GAL/HNF1A/NEUROD1/ORM1/SNAP25/FGB/FGG/FGA | 27 | BP |
| GO:0051873 | killing by host of symbiont cells | 4/397 | 0.00010222 | 0.0024012 | 0.0018704 | AZU1/CXCL6/ELANE/F2 | 4 | BP |
| GO:0042421 | norepinephrine biosynthetic process | 3/397 | 0.00010457 | 0.0024012 | 0.0018704 | INSM1/HAND2/TH | 3 | BP |
| GO:0070945 | neutrophil mediated killing of gram-negative bacterium | 3/397 | 0.00010457 | 0.0024012 | 0.0018704 | CXCL6/ELANE/F2 | 3 | BP |
| GO:0090080 | positive regulation of MAPKKK cascade by fibroblast growth factor receptor signaling pathway | 3/397 | 0.00010457 | 0.0024012 | 0.0018704 | FGF21/KLB/KL | 3 | BP |
| GO:2000851 | positive regulation of glucocorticoid secretion | 3/397 | 0.00010457 | 0.0024012 | 0.0018704 | GALR1/GAL/TAC1 | 3 | BP |
| GO:0042060 | wound healing | 27/397 | 0.00010473 | 0.0024012 | 0.0018704 | PAX7/SERPINA10/TNC/ENO3/EREG/F7/HNF4A/IGFBP1/PLAT/PLAU/WNT7A/APOA5/COMP/F13B/COL5A1/PROC/SERPIND1/SPRR3/COL1A2/VTN/FGB/KNG1/COL1A1/ITGB3/FGG/F2/FGA | 27 | BP |
| GO:0001505 | regulation of neurotransmitter levels | 19/397 | 0.00010713 | 0.00244428 | 0.00190395 | PTX3/STXBP5L/GLDC/SYT4/CHRNA3/CHRNA5/CHRNB2/SLC18A3/TPH2/DDC/CHAT/GDNF/PAH/WNT7A/GRIN2A/NQO1/SYP/SNAP25/TH | 19 | BP |
| GO:0002790 | peptide secretion | 28/397 | 0.00011002 | 0.00248819 | 0.00193816 | KCNC2/KLRF2/STXBP5L/SYBU/VGF/VSNL1/ANO1/BMP6/CASR/GLP1R/SYT4/ABCC8/ADORA1/CCL1/HNF4A/RFX6/SCG2/COMP/KRT20/ORM2/GAL/HNF1A/NEUROD1/ORM1/SNAP25/FGB/FGG/FGA | 28 | BP |
| GO:0060322 | head development | 33/397 | 0.00011011 | 0.00248819 | 0.00193816 | EMX2/KCNC2/LHX1/NR0B1/SSTR1/ALDH1A3/AQP1/CCKAR/KCNC1/KCNK3/LHX2/LMX1A/SOX1/SOX3/SYT4/CHRNB2/G6PD/KIF1A/NEFL/PHOX2A/PTF1A/FOXG1/OTX2/PHOX2B/PPARGC1A/WNT7A/GRIN2A/ASCL1/NEUROD1/NEUROG3/COL2A1/TH/COL1A1 | 33 | BP |
| GO:0006813 | potassium ion transport | 14/397 | 0.00011268 | 0.00252232 | 0.00196474 | KCNC2/KCNK2/KCNU1/NETO1/AQP1/KCNC1/KCNK3/KCNK9/ABCC8/ADORA1/KCNQ2/KCNQ5/GAL/SNAP25 | 14 | BP |
| GO:0071241 | cellular response to inorganic substance | 14/397 | 0.00011268 | 0.00252232 | 0.00196474 | ADGRV1/KCNC2/AOC1/AQP1/BMP6/KCNK3/TPH2/TF/MMP3/PPARGC1A/NQO1/TH/AKR1C3/CYP1A1 | 14 | BP |
| GO:0009790 | embryo development | 40/397 | 0.00011453 | 0.00254609 | 0.00198326 | HAND1/LHX1/NKX3-2/PAX7/PLPP4/ZIC3/ALDH1A3/DSC3/LHX2/NKX2-5/RET/TDGF1/TP63/CSF2/FOXN4/GREM2/HMGA2/COL12A1/GDF5/GREM1/HAND2/HNF4A/TXNRD1/CHRNA9/GDNF/OTX2/WNT16/WNT7A/FGF4/CDX2/COL5A1/NEUROD1/UGDH/COL11A1/COL2A1/KIT/VTN/TH/COL1A1/ITGB3 | 40 | BP |
| GO:0042476 | odontogenesis | 11/397 | 0.00011482 | 0.00254609 | 0.00198326 | HAND1/ODAPH/TNC/AQP1/TP63/HAND2/DMP1/FGF4/ASPN/COL1A2/COL1A1 | 11 | BP |
| GO:0009966 | regulation of signal transduction | 102/397 | 0.00011835 | 0.00261199 | 0.00203459 | ADGRV1/CALCB/CCL15/CTNND2/GH2/IGFBP6/INHA/IRS4/NETO1/NR0B1/NRG4/SPINK1/SPX/UCN2/VGF/ANGPTL3/BMP6/CASR/CGA/CILP/CXCL14/CXCL6/DLK1/ELANE/EREG/FZD10/GNAT1/GNG4/GRK1/NKX2-5/NOTUM/PRAME/RET/S100B/SFRP4/SH3GL2/TAF1L/TDGF1/TP63/WIF1/ADORA1/CALCA/CCL1/CCL17/CSF2/F7/FGFBP1/GREM2/LIN28A/NEFL/SNAI2/THPO/CHGB/FGF21/GDF5/GREM1/HAND2/IGFBP1/KLB/MAGEA1/PLAU/PPBP/SCG2/CHRNA9/FGF16/FGF5/GCLC/GDNF/OTX2/PPARGC1A/S100A7/TFF1/WNT16/WNT7A/FGF4/KL/PTH/CHGA/GRIN2A/ASCL1/ASPN/GAL/NEUROD1/SYP/NTS/COL2A1/GAST/KIT/TTR/AMBP/CYP26A1/VTN/AKR1C2/APOC3/FGB/COL1A1/ITGB3/FGG/SST/AKR1C3/F2/FGA | 102 | BP |
| GO:0048880 | sensory system development | 20/397 | 0.00011908 | 0.00261585 | 0.0020376 | KERA/LHX1/ALDH1A3/BMP6/COL8A2/GNAT1/LHX2/NEUROD4/RET/SOX1/FOXN4/PTF1A/PHOX2B/WNT16/WNT7A/PAX4/COL5A1/NEUROD1/TH/CYP1A1 | 20 | BP |
| GO:0006584 | catecholamine metabolic process | 7/397 | 0.00012455 | 0.00271075 | 0.00211152 | CHRNB2/INSM1/DDC/HAND2/PAH/GRIN2A/TH | 7 | BP |
| GO:0009712 | catechol-containing compound metabolic process | 7/397 | 0.00012455 | 0.00271075 | 0.00211152 | CHRNB2/INSM1/DDC/HAND2/PAH/GRIN2A/TH | 7 | BP |
| GO:0030902 | hindbrain development | 12/397 | 0.00013144 | 0.00283852 | 0.00221105 | LHX1/SSTR1/KCNC1/LMX1A/PHOX2A/PTF1A/PHOX2B/PPARGC1A/WNT7A/ASCL1/NEUROD1/NEUROG3 | 12 | BP |
| GO:1903035 | negative regulation of response to wounding | 9/397 | 0.00013168 | 0.00283852 | 0.00221105 | PLAT/PLAU/PROC/VTN/FGB/KNG1/FGG/F2/FGA | 9 | BP |
| GO:0009582 | detection of abiotic stimulus | 11/397 | 0.00013222 | 0.00283852 | 0.00221105 | ADGRV1/RGR/TRPM8/ANO1/GNAT1/GRK1/ADORA1/CHRNA9/COL11A1/KIT/TAC1 | 11 | BP |
| GO:0022037 | metencephalon development | 10/397 | 0.00013285 | 0.00283916 | 0.00221155 | LHX1/SSTR1/KCNC1/LMX1A/PHOX2A/PTF1A/PPARGC1A/WNT7A/ASCL1/NEUROD1 | 10 | BP |
| GO:0050878 | regulation of body fluid levels | 25/397 | 0.00013758 | 0.00292707 | 0.00228002 | CEL/GUCA2B/SERPINA10/AQP1/TP63/ADORA1/BPIFA1/F7/HNF4A/PLAT/PLAU/COMP/F13B/PROC/SERPIND1/COL1A2/VTN/FGB/TAC1/KNG1/COL1A1/ITGB3/FGG/F2/FGA | 25 | BP |
| GO:0051240 | positive regulation of multicellular organismal process | 62/397 | 0.00013884 | 0.00294049 | 0.00229047 | ADGRV1/GALR1/INHA/LHX1/MAG/NETO1/ODAPH/PTH2R/SPX/SRPX2/SRY/TRPM8/ANGPTL3/AQP1/AZU1/BMP6/CASR/ELANE/EREG/NKX2-5/RET/S100B/SYT4/TDGF1/TP63/CCL1/CSF2/F7/FGFBP1/HMGA2/LIN28A/NEFL/THPO/FGF21/GDF5/GREM1/HAND2/FGF16/GDNF/OTX2/PHOX2B/PPARGC1A/WNT7A/APOA5/KL/ORM2/PTH/CHGA/ASCL1/GAL/NEUROD1/NEUROG3/ORM1/THBS2/KIT/FGB/TAC1/COL1A1/ITGB3/FGG/F2/FGA | 62 | BP |
| GO:1904035 | regulation of epithelial cell apoptotic process | 9/397 | 0.00014386 | 0.00303326 | 0.00236274 | SFRP4/FGF21/SCG2/PPARGC1A/NEUROD1/FGB/FGG/AKR1C3/FGA | 9 | BP |
| GO:0019725 | cellular homeostasis | 35/397 | 0.00014627 | 0.00306788 | 0.0023897 | CALCB/CCKBR/CCL15/GALR1/RHAG/SYBU/TRPM8/VSNL1/ANO1/AQP1/BMP6/CASR/ELANE/FOXA3/GLP1R/KCNK3/ADORA1/CALCA/CCL1/LIN28A/PCK1/FGF21/RFX6/TF/CHRNA9/GCLC/PPARGC1A/ABCC2/PTH/GRIN2A/NEUROD1/TAC1/KNG1/TH/F2 | 35 | BP |
| GO:0044281 | small molecule metabolic process | 69/397 | 0.00014729 | 0.00306788 | 0.0023897 | ACOT12/B4GALNT2/CA6/CEL/ENTPD8/FADS6/GUCA2B/HAO1/IYD/KERA/MTMR7/NMNAT2/OMD/ALDH1A3/ANGPTL3/BMP6/ENO3/GLDC/HAL/OGDHL/APOF/G6PD/ODC1/PCK1/SNAI2/TPH2/DDC/G6PC/GPT2/HNF4A/NT5E/TFF3/TXNRD1/APOA4/GCLC/GPT/HGD/PAH/PPARGC1A/ABCC2/APOA5/PTH/AKR1B10/NQO1/CES1/UGDH/GSTA1/KIT/TTR/UGT2A3/UGT2B11/UGT2B4/AFP/AKR1C4/CBR3/CYP26A1/AKR1C2/APOC3/CBR1/TH/AKR1C1/UGT1A3/AKR1C3/UGT1A1/UGT1A4/UGT1A6/UGT1A7/UGT1A8/CYP1A1 | 69 | BP |
| GO:0071229 | cellular response to acid chemical | 14/397 | 0.00014745 | 0.00306788 | 0.0023897 | PTGFR/TNC/AQP1/FZD10/RET/PCK1/GCLC/PPARGC1A/AKR1C4/COL1A2/AKR1C2/AKR1C1/COL1A1/AKR1C3 | 14 | BP |
| GO:0008207 | C21-steroid hormone metabolic process | 6/397 | 0.0001484 | 0.00307405 | 0.00239451 | BMP6/PPARGC1A/AFP/AKR1C2/AKR1C1/AKR1C3 | 6 | BP |
| GO:0016999 | antibiotic metabolic process | 11/397 | 0.00015181 | 0.00313098 | 0.00243886 | HAL/APOA4/MMP3/ABCC2/AKR1B10/AKR1C4/AKR1C2/AKR1C1/AKR1C3/UGT1A1/CYP1A1 | 11 | BP |
| GO:0007200 | phospholipase C-activating G protein-coupled receptor signaling pathway | 8/397 | 0.00015441 | 0.00317086 | 0.00246992 | CCKBR/NTSR2/ANO1/CASR/CCKAR/CALCA/CHGA/F2 | 8 | BP |
| GO:0050996 | positive regulation of lipid catabolic process | 5/397 | 0.00015664 | 0.00319548 | 0.0024891 | ANGPTL3/ADORA1/FGF21/APOA4/APOA5 | 5 | BP |
| GO:0009953 | dorsal/ventral pattern formation | 9/397 | 0.00015697 | 0.00319548 | 0.0024891 | LHX1/PAX7/LHX2/SOX1/FOXN4/GREM2/GREM1/WNT7A/ASCL1 | 9 | BP |
| GO:0051093 | negative regulation of developmental process | 41/397 | 0.00015921 | 0.00321568 | 0.00250483 | ADGRV1/CLDN18/GLIS1/INHA/KCNK2/MAG/NKX3-2/CXCL14/EREG/KLK3/LHX2/LMX1A/NKX2-5/PRAME/S100B/SOX3/SYT4/TP63/CCL17/G6PD/HMGA2/LIN28A/MMP11/SNAI2/GDF5/GREM1/H4C1/H4C13/HAND2/PHOX2B/PPARGC1A/WNT7A/H4C6/PTH/ASCL1/ASPN/COL5A1/GAL/THBS2/ITGB3/F2 | 41 | BP |
| GO:0005996 | monosaccharide metabolic process | 17/397 | 0.00015932 | 0.00321568 | 0.00250483 | ENO3/G6PD/PCK1/G6PC/TFF3/GCLC/PPARGC1A/PTH/UGT2A3/UGT2B11/UGT2B4/UGT1A3/UGT1A1/UGT1A4/UGT1A6/UGT1A7/UGT1A8 | 17 | BP |
| GO:0098793 | presynapse | 22/403 | 0.00023089 | 0.00321888 | 0.0025162 | CEL/GRIK3/KCNC2/KCNK2/RAB3C/CASR/KCNC1/KCNK9/SH3GL2/SYT4/ADORA1/CHRNB2/KIF1A/NEFL/DDC/CDH9/CHAT/WNT7A/GRIN2A/SYP/SNAP25/TH | 22 | CC |
| GO:0040011 | locomotion | 64/397 | 0.00016655 | 0.00334399 | 0.00260478 | CCL15/DPYSL5/EMX2/LHX1/MAG/NRG4/PIK3C2G/PSG2/SLC7A9/SRPX2/ANGPTL3/AZU1/CASR/CCKAR/CGA/CXCL14/CXCL6/ELANE/EREG/KIF5C/LHX2/LMX1A/NEUROD4/RET/S100A2/SOX1/TDGF1/UMOD/ADORA1/CALCA/CCL1/CCL17/F7/FGFBP1/INSM1/NEFL/SNAI2/GREM1/HAND2/PLAU/SCG2/FGF16/GDNF/OTX2/PHOX2B/PPARGC1A/S100A7/WNT7A/DEFB1/DEFB4A/FGF4/CHGA/GRIN2A/ASCL1/COL5A1/ITGA11/SERPIND1/KIT/COL1A2/VTN/TAC1/COL1A1/ITGB3/F2 | 64 | BP |
| GO:0043687 | post-translational protein modification | 20/397 | 0.00016709 | 0.00334399 | 0.00260478 | SERPINA10/TNC/VGF/BPIFB2/ENAM/NOTUM/MATN3/SCG3/CHGB/IGFBP1/SCG2/TF/DMP1/APOA5/PROC/SERPIND1/AFP/KNG1/FGG/FGA | 20 | BP |
| GO:0022848 | acetylcholine-gated cation-selective channel activity | 4/372 | 0.00033578 | 0.00342705 | 0.00262681 | CHRNA3/CHRNA5/CHRNB2/CHRNA9 | 4 | MF |
| GO:0022840 | leak channel activity | 3/372 | 0.0003427 | 0.00342705 | 0.00262681 | KCNK2/RHAG/KCNK3 | 3 | MF |
| GO:0022842 | narrow pore channel activity | 3/372 | 0.0003427 | 0.00342705 | 0.00262681 | KCNK2/RHAG/KCNK3 | 3 | MF |
| GO:0007187 | G protein-coupled receptor signaling pathway, coupled to cyclic nucleotide second messenger | 13/397 | 0.00017461 | 0.00347661 | 0.00270808 | CRHR2/GALR1/GLP2R/GPR26/GRIK3/SSTR1/UCN2/CASR/GLP1R/ADORA1/CALCA/PTH/CHGA | 13 | BP |
| GO:0048646 | anatomical structure formation involved in morphogenesis | 44/397 | 0.00017519 | 0.00347661 | 0.00270808 | HAND1/LHX1/MYPN/NKX3-2/ODAPH/SRPX2/TNMD/ALDH1A3/ANGPTL3/AQP1/COL8A2/EREG/KLK3/LHX2/NKX2-5/RET/TDGF1/TP63/FGFBP1/FOXN4/HMGA2/PHOX2A/PTF1A/SNAI2/TGFBI/COL12A1/GREM1/HAND2/SCG2/TXNRD1/DMP1/GDNF/OTX2/S100A7/WNT16/WNT7A/CDX2/COL5A1/THBS2/COL11A1/COL2A1/VTN/COL1A1/ITGB3 | 44 | BP |
| GO:0031960 | response to corticosteroid | 12/397 | 0.00017912 | 0.00353969 | 0.00275722 | CPN1/AQP1/BMP6/S100B/NEFL/TPH2/ALDH3A1/ABCC2/TH/COL1A1/AKR1C3/UGT1A1 | 12 | BP |
| GO:0007420 | brain development | 31/397 | 0.00018133 | 0.00356846 | 0.00277963 | EMX2/KCNC2/LHX1/NR0B1/SSTR1/ALDH1A3/AQP1/CCKAR/KCNC1/KCNK3/LHX2/LMX1A/SOX1/SOX3/SYT4/CHRNB2/G6PD/KIF1A/NEFL/PHOX2A/PTF1A/FOXG1/OTX2/PHOX2B/PPARGC1A/WNT7A/GRIN2A/ASCL1/NEUROD1/NEUROG3/TH | 31 | BP |
| GO:0007399 | nervous system development | 74/397 | 0.00018621 | 0.00364934 | 0.00284263 | ADGRV1/CPLX2/CTNND2/DPYSL5/EMX2/KCNC2/LHX1/MAG/NR0B1/NRG4/PAX7/SRPX2/SSTR1/SYBU/TNC/ZIC3/ALDH1A3/AQP1/AZU1/BEX1/BMP6/CCKAR/FZD10/GNAT1/KCNC1/KCNK3/KIF5C/LHX2/LMX1A/NEUROD4/NKX2-5/PCSK2/RET/S100B/SH3GL2/SOX1/SOX3/SYT4/TP63/ADORA1/CHRNA3/CHRNB2/FOXN4/G6PD/GABRA1/INSM1/KIF1A/LIN28A/NEFL/PHOX2A/PTF1A/CDH11/FOXG1/GDF5/HAND2/CDH9/FGF5/GDNF/KCNQ2/OTX2/PHOX2B/PPARGC1A/WNT7A/GRIN2A/ASCL1/NEUROD1/NEUROG3/THBS2/SNAP25/COL2A1/KIT/VTN/TH/F2 | 74 | BP |
| GO:1902495 | transmembrane transporter complex | 16/403 | 0.0002842 | 0.00384887 | 0.00300866 | KCNC2/KCNK2/ANO1/GABRG3/KCNC1/ABCC8/CHRNA3/CHRNA5/CHRNB2/GABRA1/CACNA1S/CHRNA9/KCNQ2/KCNQ5/GRIN2A/SNAP25 | 16 | CC |
| GO:0050804 | modulation of chemical synaptic transmission | 21/397 | 0.00019725 | 0.00384962 | 0.00299864 | CPLX2/GRIK3/LRFN2/NETO1/STXBP5L/SYBU/S100B/SYT4/ADORA1/CHRNA3/CHRNA5/CHRNB2/CDH11/PLAT/GDNF/WNT7A/GRIN2A/SYP/SNAP25/KIT/TAC1 | 21 | BP |
| GO:0051852 | disruption by host of symbiont cells | 4/397 | 0.00019957 | 0.00386295 | 0.00300901 | AZU1/CXCL6/ELANE/F2 | 4 | BP |
| GO:2000846 | regulation of corticosteroid hormone secretion | 4/397 | 0.00019957 | 0.00386295 | 0.00300901 | GALR1/BMP6/GAL/TAC1 | 4 | BP |
| GO:0009072 | aromatic amino acid family metabolic process | 6/397 | 0.00020063 | 0.00386763 | 0.00301266 | IYD/HAL/TPH2/HGD/PAH/TH | 6 | BP |
| GO:0042562 | hormone binding | 8/372 | 0.00039505 | 0.00389566 | 0.002986 | CCKBR/GALR1/MLNR/UCN2/ALDH1A3/CCKAR/CHRNA3/CHRNB2 | 8 | MF |
| GO:0005230 | extracellular ligand-gated ion channel activity | 7/372 | 0.00040133 | 0.00390339 | 0.00299193 | GRIK3/CHRNA3/CHRNA5/CHRNB2/GABRA1/CHRNA9/GRIN2A | 7 | MF |
| GO:0099177 | regulation of trans-synaptic signaling | 21/397 | 0.00020419 | 0.00392025 | 0.00305365 | CPLX2/GRIK3/LRFN2/NETO1/STXBP5L/SYBU/S100B/SYT4/ADORA1/CHRNA3/CHRNA5/CHRNB2/CDH11/PLAT/GDNF/WNT7A/GRIN2A/SYP/SNAP25/KIT/TAC1 | 21 | BP |
| GO:0016115 | terpenoid catabolic process | 3/397 | 0.0002057 | 0.00393336 | 0.00306386 | AKR1B10/CYP26A1/AKR1C3 | 3 | BP |
| GO:0097305 | response to alcohol | 15/397 | 0.00021909 | 0.00417242 | 0.00325008 | CLDN18/KCNC2/PTGFR/TNC/CHRNB2/F7/G6PD/NEFL/PTH/GRIN2A/NQO1/AKR1C2/TH/AKR1C3/UGT1A1 | 15 | BP |
| GO:0098797 | plasma membrane protein complex | 22/403 | 0.0003456 | 0.00434463 | 0.0033962 | C8G/INHA/KCNC2/KCNK2/KCNC1/RET/ABCC8/C8A/CHRNA3/CHRNA5/CHRNB2/CACNA1S/TF/CHRNA9/GABBR2/KCNQ2/KCNQ5/GRIN2A/ITGA11/SNAP25/VTN/ITGB3 | 22 | CC |
| GO:0044459 | plasma membrane part | 73/403 | 0.00034734 | 0.00434463 | 0.0033962 | ADGRV1/C8G/CDHR2/CLCA2/CRHR2/FCER1A/GRIK3/INHA/IYD/KCNC2/KCNE4/KCNK2/KLRF2/LRFN2/MAG/NETO1/NTSR2/PTGFR/PTH2R/SLC10A2/SLC6A19/SLC7A9/SRPX2/TRPM8/ADAM2/ANO1/AQP1/ATP4A/CASR/CD1A/CLDN8/EREG/FZD10/GABRG3/GLP1R/GNAT1/GRK1/KCNC1/RET/TDGF1/UMOD/ABCC8/ADORA1/C8A/CDH17/CHRNA3/CHRNA5/CHRNB2/G6PD/GABRA1/CACNA1S/TF/CDH9/CHRNA9/GABBR2/KCNQ2/KCNQ5/MUC13/ABCC2/KL/GRIN2A/ITGA11/SYP/SNAP25/KIT/VTN/FGB/TH/ITGB3/FGG/F2/UGT1A1/FGA | 73 | CC |
| GO:1990351 | transporter complex | 16/403 | 0.0003483 | 0.00434463 | 0.0033962 | KCNC2/KCNK2/ANO1/GABRG3/KCNC1/ABCC8/CHRNA3/CHRNA5/CHRNB2/GABRA1/CACNA1S/CHRNA9/KCNQ2/KCNQ5/GRIN2A/SNAP25 | 16 | CC |
| GO:0005783 | endoplasmic reticulum | 60/403 | 0.00036184 | 0.00439776 | 0.00343774 | ANKS4B/CLGN/GUCY2C/KCNK2/SERPINA10/TNC/TRPM8/VGF/BPIFB2/CLDN8/COL8A2/ENAM/NOTUM/OCA2/TP63/ADORA1/F7/LIN28A/MATN3/SCG3/CHGB/COL12A1/G6PC/IGFBP1/SCG2/TF/ALDH3A1/APOA4/DMP1/S100A7/WNT7A/APOA5/COL10A1/GRIN2A/COL5A1/PROC/CES1/SERPIND1/COL11A1/COL2A1/UGT2B11/UGT2B4/AFP/COL1A2/CYP26A1/VTN/FGB/KNG1/TH/COL1A1/FGG/UGT1A3/F2/UGT1A1/UGT1A4/UGT1A6/UGT1A7/UGT1A8/CYP1A1/FGA | 60 | CC |
| GO:0050890 | cognition | 17/397 | 0.00023272 | 0.00441429 | 0.00343848 | KCNK2/NETO1/PPP1R1B/ADAM2/GLP1R/LMX1A/S100B/SYT4/ADORA1/CHRNB2/KCNK10/GRIN2A/SNAP25/KIT/CBR3/TAC1/TH | 17 | BP |
| GO:0061037 | negative regulation of cartilage development | 5/397 | 0.00023375 | 0.00441591 | 0.00343974 | NKX3-2/SNAI2/GDF5/GREM1/PTH | 5 | BP |
| GO:0022600 | digestive system process | 9/397 | 0.00023838 | 0.00448553 | 0.00349397 | CCKBR/CEL/AQP1/MUC13/MUC6/NEUROD1/MUC2/TAC1/AKR1C1 | 9 | BP |
| GO:0001654 | eye development | 19/397 | 0.00024198 | 0.00453517 | 0.00353264 | KERA/LHX1/ALDH1A3/BMP6/COL8A2/GNAT1/LHX2/NEUROD4/RET/SOX1/FOXN4/PTF1A/WNT16/WNT7A/PAX4/COL5A1/NEUROD1/TH/CYP1A1 | 19 | BP |
| GO:0006812 | cation transport | 41/397 | 0.00024432 | 0.00456096 | 0.00355273 | KCNC2/KCNK2/KCNU1/NETO1/RHAG/SCN3A/SLC10A2/SLC17A2/SLC5A5/SPINK1/TRPM8/ANO1/AQP1/CASR/KCNC1/KCNK3/KCNK9/NKX2-5/OCA2/ABCC8/ADORA1/BPIFA1/CHRNA3/CHRNB2/CSF2/G6PD/NEFL/SLC18A3/CACNA1S/TF/CHRNA9/GDNF/KCNQ2/KCNQ5/PPARGC1A/ABCC2/PTH/GRIN2A/GAL/SNAP25/F2 | 41 | BP |
| GO:0033273 | response to vitamin | 9/397 | 0.00025827 | 0.00480237 | 0.00374077 | TNC/CASR/F7/SNAI2/PTH/ASCL1/CYP26A1/COL1A1/CYP1A1 | 9 | BP |
| GO:0035930 | corticosteroid hormone secretion | 4/397 | 0.00026739 | 0.0049332 | 0.00384268 | GALR1/BMP6/GAL/TAC1 | 4 | BP |
| GO:0051883 | killing of cells in other organism involved in symbiotic interaction | 4/397 | 0.00026739 | 0.0049332 | 0.00384268 | AZU1/CXCL6/ELANE/F2 | 4 | BP |
| GO:0007188 | adenylate cyclase-modulating G protein-coupled receptor signaling pathway | 12/397 | 0.00027017 | 0.00496512 | 0.00386755 | CRHR2/GALR1/GLP2R/GPR26/GRIK3/UCN2/CASR/GLP1R/ADORA1/CALCA/PTH/CHGA | 12 | BP |
| GO:0060205 | cytoplasmic vesicle lumen | 18/403 | 0.00042447 | 0.00502999 | 0.00393195 | PTX3/ZG16/AOC1/AZU1/ELANE/PRSS2/SCG3/DEFA5/PPBP/TF/S100A7/ORM2/ORM1/TTR/FGB/KNG1/FGG/FGA | 18 | CC |
| GO:0015837 | amine transport | 8/397 | 0.00027483 | 0.00503112 | 0.00391895 | SYT4/ADORA1/CHRNA3/CHRNB2/DDC/GDNF/CHGA/TH | 8 | BP |
| GO:0003002 | regionalization | 18/397 | 0.00027984 | 0.00507796 | 0.00395544 | EMX2/LHX1/PAX7/ZIC3/LHX2/NKX2-5/SOX1/TDGF1/TP63/FOXN4/GREM2/GREM1/GDNF/OTX2/WNT7A/CDX2/ASCL1/NEUROD1 | 18 | BP |
| GO:0150063 | visual system development | 19/397 | 0.00028012 | 0.00507796 | 0.00395544 | KERA/LHX1/ALDH1A3/BMP6/COL8A2/GNAT1/LHX2/NEUROD4/RET/SOX1/FOXN4/PTF1A/WNT16/WNT7A/PAX4/COL5A1/NEUROD1/TH/CYP1A1 | 19 | BP |
| GO:0035883 | enteroendocrine cell differentiation | 5/397 | 0.00028169 | 0.00507796 | 0.00395544 | BMP6/INSM1/HNF4A/RFX6/NEUROD1 | 5 | BP |
| GO:0035902 | response to immobilization stress | 5/397 | 0.00028169 | 0.00507796 | 0.00395544 | NR0B1/TFF1/GAL/TH/CYP1A1 | 5 | BP |
| GO:0031983 | vesicle lumen | 18/403 | 0.00043959 | 0.0050821 | 0.00397268 | PTX3/ZG16/AOC1/AZU1/ELANE/PRSS2/SCG3/DEFA5/PPBP/TF/S100A7/ORM2/ORM1/TTR/FGB/KNG1/FGG/FGA | 18 | CC |
| GO:0042379 | chemokine receptor binding | 6/372 | 0.00053618 | 0.00514444 | 0.00394318 | CXCL14/CXCL6/CCL1/CCL17/DEFB1/DEFB4A | 6 | MF |
| GO:0097458 | neuron part | 52/403 | 0.0004571 | 0.00515876 | 0.00403261 | ADGRV1/CEL/CTNND2/DPYSL5/GRIK3/GUCA2B/INHA/KCNC2/KCNK2/MAG/NETO1/NMNAT2/PPP1R1B/RAB3C/STXBP5L/SYBU/SYT5/CASR/GNAT1/GRK1/KCNC1/KCNK9/KIF5C/PCSK2/RET/S100B/SH3GL2/SYT4/TP63/ADORA1/CHRNA3/CHRNB2/KIF1A/NEFL/DDC/SCG2/CDH9/CHAT/GABBR2/KCNQ2/OTX2/PPARGC1A/WNT7A/GRIN2A/ASCL1/GAL/NQO1/SYP/SNAP25/TAC1/TH/SST | 52 | CC |
| GO:0071804 | cellular potassium ion transport | 11/397 | 0.0002909 | 0.00520424 | 0.0040538 | KCNC2/KCNK2/KCNU1/NETO1/AQP1/KCNC1/ABCC8/KCNQ2/KCNQ5/GAL/SNAP25 | 11 | BP |
| GO:0071805 | potassium ion transmembrane transport | 11/397 | 0.0002909 | 0.00520424 | 0.0040538 | KCNC2/KCNK2/KCNU1/NETO1/AQP1/KCNC1/ABCC8/KCNQ2/KCNQ5/GAL/SNAP25 | 11 | BP |
| GO:0007166 | cell surface receptor signaling pathway | 89/397 | 0.00030037 | 0.00534201 | 0.00416112 | ADGRV1/CCKBR/CLDN18/CRHR2/CTNND2/FCER1A/GH2/GLP2R/GRIK3/IGFBP6/INHA/NETO1/NRG4/NTSR2/PLPP4/PTH2R/SSTR1/ANGPTL3/BMP6/CILP/DLK1/EREG/FZD10/GLP1R/NEUROD4/NKX2-5/NOTUM/RET/SFRP4/SH3GL2/TP63/WIF1/ADORA1/CDH17/CHRNA3/CHRNA5/CHRNB2/CSF2/F7/FGFBP1/GREM2/NEFL/SNAI2/THPO/FGF21/GDF5/GREM1/GSTA2/HAND2/HNF4A/IGFBP1/KLB/MAGEA1/SCG2/CHRNA9/FGF16/FGF5/GCLC/GDNF/MMP3/MUC13/MUC19/MUC21/OTX2/PPARGC1A/WNT16/WNT7A/COMP/FGF4/KL/GRIN2A/ASCL1/ASPN/ITGA11/MUC6/MUC2/MUC5AC/COL2A1/KIT/AFP/COL1A2/VTN/FGB/COL1A1/ITGB3/FGG/SST/F2/FGA | 89 | BP |
| GO:0008306 | associative learning | 8/397 | 0.00030086 | 0.00534201 | 0.00416112 | NETO1/PPP1R1B/ADAM2/CHRNB2/GRIN2A/SNAP25/KIT/TAC1 | 8 | BP |
| GO:0036477 | somatodendritic compartment | 30/403 | 0.00051977 | 0.0057296 | 0.00447884 | CTNND2/DPYSL5/GRIK3/INHA/KCNC2/KCNK2/PPP1R1B/SYT5/CASR/GNAT1/KCNC1/KIF5C/PCSK2/RET/S100B/SH3GL2/SYT4/TP63/ADORA1/CHRNA3/KIF1A/DDC/PPARGC1A/ASCL1/GAL/NQO1/SNAP25/TAC1/TH/SST | 30 | CC |
| GO:0007389 | pattern specification process | 21/397 | 0.00032654 | 0.00577614 | 0.00449928 | EMX2/HAND1/LHX1/NKX3-2/PAX7/ZIC3/LHX2/NKX2-5/SOX1/TDGF1/TP63/FOXN4/GREM2/GREM1/HAND2/GDNF/OTX2/WNT7A/CDX2/ASCL1/NEUROD1 | 21 | BP |
| GO:0005581 | collagen trimer | 8/403 | 0.00053642 | 0.00577872 | 0.00451724 | COL8A2/COL12A1/COL10A1/COL5A1/COL11A1/COL2A1/COL1A2/COL1A1 | 8 | CC |
| GO:0042180 | cellular ketone metabolic process | 15/397 | 0.00032972 | 0.00578656 | 0.0045074 | BMP6/ODC1/APOA4/PPARGC1A/APOA5/AKR1B10/NQO1/AFP/AKR1C4/CBR3/AKR1C2/APOC3/AKR1C1/AKR1C3/UGT1A8 | 15 | BP |
| GO:1901617 | organic hydroxy compound biosynthetic process | 15/397 | 0.00032972 | 0.00578656 | 0.0045074 | BMP6/OCA2/G6PD/INSM1/PCK1/SNAI2/TPH2/DDC/HAND2/PAH/PTH/CES1/AKR1C4/TH/AKR1C3 | 15 | BP |
| GO:0009749 | response to glucose | 13/397 | 0.0003308 | 0.00578656 | 0.0045074 | SYBU/VSNL1/ANO1/CASR/LIN28A/PCK1/FGF21/HNF4A/RFX6/GCLC/PPARGC1A/NEUROD1/TH | 13 | BP |
| GO:0001664 | G protein-coupled receptor binding | 13/372 | 0.00061207 | 0.00579428 | 0.00444128 | CCKBR/PPP1R1B/UCN2/CXCL14/CXCL6/ADORA1/CALCA/CCL1/CCL17/DEFB1/DEFB4A/PTH/GAL | 13 | MF |
| GO:0016485 | protein processing | 17/397 | 0.00033397 | 0.0057991 | 0.00451717 | C8G/CPN1/CGA/PCSK2/C8A/CFHR5/F7/PLAT/PLAU/COMP/GRIN2A/CES1/VTN/FGB/FGG/F2/FGA | 17 | BP |
| GO:0043010 | camera-type eye development | 17/397 | 0.00033397 | 0.0057991 | 0.00451717 | KERA/LHX1/ALDH1A3/COL8A2/GNAT1/LHX2/NEUROD4/RET/SOX1/FOXN4/PTF1A/WNT16/WNT7A/PAX4/NEUROD1/TH/CYP1A1 | 17 | BP |
| GO:0051047 | positive regulation of secretion | 21/397 | 0.00033733 | 0.00582621 | 0.00453828 | GALR1/INHA/SYBU/VSNL1/ANO1/AQP1/BMP6/CASR/SYT4/ADORA1/CCL1/CHRNB2/RFX6/GDNF/ORM2/GAL/ORM1/FGB/TAC1/FGG/FGA | 21 | BP |
| GO:0019935 | cyclic-nucleotide-mediated signaling | 12/397 | 0.000338 | 0.00582621 | 0.00453828 | GALR1/GPR26/GUCA2B/KCNC2/UCN2/AQP1/GLP1R/CALCA/DEFB1/PTH/CHGA/GAL | 12 | BP |
| GO:0055123 | digestive system development | 11/397 | 0.0003493 | 0.00586747 | 0.00457043 | CCKBR/CLDN18/NKX3-2/ZIC3/RET/TP63/PTF1A/CDX2/ASCL1/KIT/CYP1A1 | 11 | BP |
| GO:0003310 | pancreatic A cell differentiation | 3/397 | 0.00035406 | 0.00586747 | 0.00457043 | INSM1/RFX6/NEUROD1 | 3 | BP |
| GO:0006787 | porphyrin-containing compound catabolic process | 3/397 | 0.00035406 | 0.00586747 | 0.00457043 | AMBP/UGT1A1/UGT1A4 | 3 | BP |
| GO:0009698 | phenylpropanoid metabolic process | 3/397 | 0.00035406 | 0.00586747 | 0.00457043 | UGT1A7/UGT1A8/CYP1A1 | 3 | BP |
| GO:0009804 | coumarin metabolic process | 3/397 | 0.00035406 | 0.00586747 | 0.00457043 | UGT1A7/UGT1A8/CYP1A1 | 3 | BP |
| GO:0010898 | positive regulation of triglyceride catabolic process | 3/397 | 0.00035406 | 0.00586747 | 0.00457043 | FGF21/APOA4/APOA5 | 3 | BP |
| GO:0033015 | tetrapyrrole catabolic process | 3/397 | 0.00035406 | 0.00586747 | 0.00457043 | AMBP/UGT1A1/UGT1A4 | 3 | BP |
| GO:0035095 | behavioral response to nicotine | 3/397 | 0.00035406 | 0.00586747 | 0.00457043 | CHRNA3/CHRNA5/CHRNB2 | 3 | BP |
| GO:0042167 | heme catabolic process | 3/397 | 0.00035406 | 0.00586747 | 0.00457043 | AMBP/UGT1A1/UGT1A4 | 3 | BP |
| GO:0046149 | pigment catabolic process | 3/397 | 0.00035406 | 0.00586747 | 0.00457043 | AMBP/UGT1A1/UGT1A4 | 3 | BP |
| GO:0099505 | regulation of presynaptic membrane potential | 3/397 | 0.00035406 | 0.00586747 | 0.00457043 | GRIK3/KCNC2/KCNC1 | 3 | BP |
| GO:0006629 | lipid metabolic process | 49/397 | 0.00035569 | 0.00587385 | 0.00457539 | ALPI/B4GALNT2/CCKBR/CEL/FADS6/HAO1/MTMR7/NR0B1/PIK3C2G/PLPP4/ALDH1A3/ANGPTL3/BMP6/CGA/ADORA1/APOF/G6PD/PCK1/SNAI2/FGF21/G6PC/HNF4A/TXNRD1/APOA4/CHAT/PPARGC1A/APOA5/AKR1B10/GAL/CES1/GSTA1/KIT/TTR/UGT2B11/AFP/AKR1C4/CYP26A1/AKR1C2/APOC3/CBR1/TH/AKR1C1/UGT1A3/AKR1C3/F2/UGT1A1/UGT1A7/UGT1A8/CYP1A1 | 49 | BP |
| GO:0071248 | cellular response to metal ion | 12/397 | 0.00035705 | 0.00587574 | 0.00457687 | ADGRV1/AOC1/AQP1/BMP6/KCNK3/TPH2/TF/PPARGC1A/NQO1/TH/AKR1C3/CYP1A1 | 12 | BP |
| GO:0048259 | regulation of receptor-mediated endocytosis | 9/397 | 0.0003795 | 0.00620199 | 0.004831 | SFRP4/SH3GL2/H1-1/GREM1/TF/APOA5/VTN/APOC3/ITGB3 | 9 | BP |
| GO:2001237 | negative regulation of extrinsic apoptotic signaling pathway | 9/397 | 0.0003795 | 0.00620199 | 0.004831 | CSF2/SNAI2/SCG2/GCLC/GDNF/COL2A1/FGB/FGG/FGA | 9 | BP |
| GO:0031093 | platelet alpha granule lumen | 7/403 | 0.00059043 | 0.00621916 | 0.00486152 | PPBP/ORM2/ORM1/FGB/KNG1/FGG/FGA | 7 | CC |
| GO:0097164 | ammonium ion metabolic process | 13/397 | 0.00038466 | 0.00626463 | 0.00487979 | CHRNB2/ODC1/TPH2/DDC/APOA4/CHAT/AKR1B10/GRIN2A/AKR1C4/AKR1C2/TH/AKR1C1/AKR1C3 | 13 | BP |
| GO:0030133 | transport vesicle | 18/403 | 0.00061831 | 0.00637126 | 0.00498042 | RAB3C/SPX/VGF/KCNK9/PCSK2/SH3GL2/SYT4/KIF1A/SCG3/SLC18A3/DDC/DEFA5/CHGA/GRIN2A/SYP/NTS/SNAP25/TH | 18 | CC |
| GO:0050796 | regulation of insulin secretion | 12/397 | 0.00039788 | 0.00645765 | 0.00503014 | KCNC2/STXBP5L/SYBU/VSNL1/ANO1/CASR/GLP1R/ABCC8/HNF4A/RFX6/NEUROD1/SNAP25 | 12 | BP |
| GO:0034774 | secretory granule lumen | 17/403 | 0.00064756 | 0.0065307 | 0.00510505 | PTX3/AOC1/AZU1/ELANE/PRSS2/SCG3/DEFA5/PPBP/TF/S100A7/ORM2/ORM1/TTR/FGB/KNG1/FGG/FGA | 17 | CC |
| GO:0016709 | oxidoreductase activity, acting on paired donors, with incorporation or reduction of molecular oxygen, NAD(P)H as one donor, and incorporation of one atom of oxygen | 5/372 | 0.00070602 | 0.00659569 | 0.00505555 | CYP26A1/AKR1C2/AKR1C1/AKR1C3/CYP1A1 | 5 | MF |
| GO:0008236 | serine-type peptidase activity | 11/372 | 0.00073784 | 0.00680342 | 0.00521478 | AZU1/ELANE/KLK3/PCSK2/F7/PRSS2/PLAT/PLAU/MMP3/PROC/F2 | 11 | MF |
| GO:0009746 | response to hexose | 13/397 | 0.00042456 | 0.00686717 | 0.00534913 | SYBU/VSNL1/ANO1/CASR/LIN28A/PCK1/FGF21/HNF4A/RFX6/GCLC/PPARGC1A/NEUROD1/TH | 13 | BP |
| GO:0007611 | learning or memory | 15/397 | 0.00042768 | 0.00689401 | 0.00537004 | KCNK2/NETO1/PPP1R1B/ADAM2/GLP1R/LMX1A/S100B/SYT4/CHRNB2/KCNK10/GRIN2A/SNAP25/KIT/TAC1/TH | 15 | BP |
| GO:0021510 | spinal cord development | 9/397 | 0.00043958 | 0.00703777 | 0.00548202 | LHX1/PAX7/SOX1/FOXN4/NEFL/PHOX2A/GDNF/ASCL1/NEUROG3 | 9 | BP |
| GO:0032963 | collagen metabolic process | 9/397 | 0.00043958 | 0.00703777 | 0.00548202 | MMP10/MMP11/PRSS2/MMP13/MMP3/COL5A1/COL1A2/COL1A1/F2 | 9 | BP |
| GO:0019218 | regulation of steroid metabolic process | 10/397 | 0.00044255 | 0.00706143 | 0.00550045 | BMP6/CGA/SNAI2/APOA4/PPARGC1A/GAL/KIT/AKR1C3/UGT1A1/UGT1A8 | 10 | BP |
| GO:0017085 | response to insecticide | 4/397 | 0.00045013 | 0.00708652 | 0.00552 | NEFL/DDC/TH/CYP1A1 | 4 | BP |
| GO:0030277 | maintenance of gastrointestinal epithelium | 4/397 | 0.00045013 | 0.00708652 | 0.00552 | MUC13/MUC6/NEUROD1/MUC2 | 4 | BP |
| GO:0051818 | disruption of cells of other organism involved in symbiotic interaction | 4/397 | 0.00045013 | 0.00708652 | 0.00552 | AZU1/CXCL6/ELANE/F2 | 4 | BP |
| GO:2000831 | regulation of steroid hormone secretion | 4/397 | 0.00045013 | 0.00708652 | 0.00552 | GALR1/BMP6/GAL/TAC1 | 4 | BP |
| GO:0007596 | blood coagulation | 18/397 | 0.00045204 | 0.00709292 | 0.00552498 | SERPINA10/F7/HNF4A/PLAT/PLAU/COMP/F13B/PROC/SERPIND1/COL1A2/VTN/FGB/KNG1/COL1A1/ITGB3/FGG/F2/FGA | 18 | BP |
| GO:0065009 | regulation of molecular function | 94/397 | 0.0004596 | 0.00718772 | 0.00559882 | ADGRV1/CALCB/CCKBR/CCL15/GALR1/GH2/GUCA2B/HAND1/INHA/KCNC2/NETO1/NR0B1/NRG4/PAX7/PPP1R1B/PTX3/RIMBP2/SPINK1/SPINK4/SPX/STXBP5L/UCN2/VGF/WFDC12/WFDC5/ANGPTL3/AQP1/AZU1/BEX1/BMP6/CASR/CGA/CXCL14/CXCL6/ELANE/EREG/FZD10/GLP1R/GNAT1/KCNC1/LHX2/RET/SFRP4/TDGF1/TP63/ADORA1/CALCA/CCL1/CCL17/CFHR5/CHRNA3/CSF2/GREM2/HMGA2/NEFL/THPO/CHGB/FGF21/GDF5/GREM1/HAND2/PLAU/PPBP/SCG2/APOA4/FGF16/FGF5/GABBR2/GDNF/PPARGC1A/TFF1/WNT7A/APOA5/FGF4/KL/PTH/GRIN2A/GAL/NEUROD1/NEUROG3/NQO1/NTS/GAST/KIT/TTR/AMBP/VTN/APOC3/ITGB3/SST/F2/UGT1A1/UGT1A7/UGT1A8 | 94 | BP |
| GO:0006869 | lipid transport | 18/397 | 0.00046844 | 0.00730186 | 0.00568773 | CEL/GALR1/LCN12/SLC10A2/SPX/BMP6/APOF/SLCO1A2/APOA4/ABCC2/APOA5/GAL/CES1/AKR1C4/APOC3/TAC1/AKR1C1/ITGB3 | 18 | BP |
| GO:0009581 | detection of external stimulus | 10/397 | 0.00047174 | 0.00731575 | 0.00569855 | ADGRV1/RGR/TRPM8/ANO1/GNAT1/GRK1/ADORA1/CHRNA9/COL11A1/KIT | 10 | BP |
| GO:0032526 | response to retinoic acid | 9/397 | 0.00047243 | 0.00731575 | 0.00569855 | TNC/AQP1/BMP6/FZD10/RET/PCK1/ASCL1/CYP26A1/COL1A1 | 9 | BP |
| GO:0050801 | ion homeostasis | 29/397 | 0.0004763 | 0.00734762 | 0.00572338 | CALCB/CCKBR/CCL15/GALR1/RHAG/SPX/TRPM8/ANGPTL3/BMP6/CASR/ELANE/GLP1R/KCNK3/SFRP4/UMOD/ADORA1/CALCA/CCL1/HNF4A/TF/CHRNA9/GCLC/ABCC2/KL/PTH/GRIN2A/TAC1/KNG1/F2 | 29 | BP |
| GO:0006820 | anion transport | 25/397 | 0.0004776 | 0.00734762 | 0.00572338 | CA6/CLCA2/LCN12/RHAG/SLC10A2/SLC5A5/SLC6A19/SLC7A9/SPX/ANO1/AQP1/CASR/OCA2/SFRP4/SYT4/ADORA1/GABRA1/SLCO1A2/G6PC/APOA4/ABCC2/SNAP25/AKR1C4/APOC3/AKR1C1 | 25 | BP |
| GO:0016620 | oxidoreductase activity, acting on the aldehyde or oxo group of donors, NAD or NADP as acceptor | 5/372 | 0.00081303 | 0.00740065 | 0.00567255 | ALDH1A3/ALDH3A1/AKR1B10/AKR1C4/AKR1C3 | 5 | MF |
| GO:0098542 | defense response to other organism | 23/397 | 0.00048775 | 0.00747934 | 0.00582598 | CD207/VGF/WFDC12/AZU1/CXCL6/DEFB126/ELANE/KLK3/RNASE7/SPAG11B/BPIFA1/DEFA6/BPIFA2/DEFA5/PPBP/LCE3A/S100A7/DEFB1/DEFB4A/CHGA/FGB/F2/FGA | 23 | BP |
| GO:0014075 | response to amine | 6/397 | 0.00050554 | 0.00765281 | 0.0059611 | KCNC2/PPP1R1B/GLDC/KCNC1/GRIN2A/TH | 6 | BP |
| GO:0031638 | zymogen activation | 6/397 | 0.00050554 | 0.00765281 | 0.0059611 | PLAT/PLAU/GRIN2A/FGB/FGG/FGA | 6 | BP |
| GO:0032330 | regulation of chondrocyte differentiation | 6/397 | 0.00050554 | 0.00765281 | 0.0059611 | NKX3-2/BMP6/SNAI2/GDF5/GREM1/PTH | 6 | BP |
| GO:1904036 | negative regulation of epithelial cell apoptotic process | 6/397 | 0.00050554 | 0.00765281 | 0.0059611 | FGF21/SCG2/NEUROD1/FGB/FGG/FGA | 6 | BP |
| GO:0099094 | ligand-gated cation channel activity | 8/372 | 0.00086386 | 0.00776383 | 0.00595093 | GRIK3/AQP1/CHRNA3/CHRNA5/CHRNB2/KCNJ18/CHRNA9/GRIN2A | 8 | MF |
| GO:0045471 | response to ethanol | 10/397 | 0.00053493 | 0.00807084 | 0.00628673 | CLDN18/KCNC2/TNC/CHRNB2/G6PD/PTH/GRIN2A/NQO1/TH/UGT1A1 | 10 | BP |
| GO:0007599 | hemostasis | 18/397 | 0.00053934 | 0.00807084 | 0.00628673 | SERPINA10/F7/HNF4A/PLAT/PLAU/COMP/F13B/PROC/SERPIND1/COL1A2/VTN/FGB/KNG1/COL1A1/ITGB3/FGG/F2/FGA | 18 | BP |
| GO:0009612 | response to mechanical stimulus | 13/397 | 0.00053999 | 0.00807084 | 0.00628673 | ADGRV1/KCNK2/MAG/TNC/AQP1/BMP6/KCNC1/GDF5/CHRNA9/GCLC/COL11A1/KIT/COL1A1 | 13 | BP |
| GO:0034284 | response to monosaccharide | 13/397 | 0.00053999 | 0.00807084 | 0.00628673 | SYBU/VSNL1/ANO1/CASR/LIN28A/PCK1/FGF21/HNF4A/RFX6/GCLC/PPARGC1A/NEUROD1/TH | 13 | BP |
| GO:0051050 | positive regulation of transport | 37/397 | 0.00054818 | 0.00811544 | 0.00632146 | GALR1/INHA/KCNC2/PTX3/SYBU/VSNL1/ANO1/AQP1/AZU1/BMP6/CASR/KCNC1/NKX2-5/SFRP4/SYT4/TP63/ADORA1/CCL1/CHRNB2/G6PD/H1-1/FGF21/GREM1/RFX6/TF/GDNF/APOA5/ORM2/PTH/GAL/ORM1/VTN/FGB/TAC1/FGG/F2/FGA | 37 | BP |
| GO:0072376 | protein activation cascade | 11/397 | 0.00055504 | 0.00811544 | 0.00632146 | C8G/CPN1/C8A/CFHR5/F7/VTN/FGB/KNG1/FGG/F2/FGA | 11 | BP |
| GO:0006570 | tyrosine metabolic process | 3/397 | 0.00055719 | 0.00811544 | 0.00632146 | IYD/HGD/TH | 3 | BP |
| GO:0008300 | isoprenoid catabolic process | 3/397 | 0.00055719 | 0.00811544 | 0.00632146 | AKR1B10/CYP26A1/AKR1C3 | 3 | BP |
| GO:0070170 | regulation of tooth mineralization | 3/397 | 0.00055719 | 0.00811544 | 0.00632146 | ODAPH/DMP1/ASPN | 3 | BP |
| GO:0070327 | thyroid hormone transport | 3/397 | 0.00055719 | 0.00811544 | 0.00632146 | ABCC2/SERPINA7/TTR | 3 | BP |
| GO:1903011 | negative regulation of bone development | 3/397 | 0.00055719 | 0.00811544 | 0.00632146 | CLDN18/GREM1/PTH | 3 | BP |
| GO:2000849 | regulation of glucocorticoid secretion | 3/397 | 0.00055719 | 0.00811544 | 0.00632146 | GALR1/GAL/TAC1 | 3 | BP |
| GO:0050817 | coagulation | 18/397 | 0.00055844 | 0.00811544 | 0.00632146 | SERPINA10/F7/HNF4A/PLAT/PLAU/COMP/F13B/PROC/SERPIND1/COL1A2/VTN/FGB/KNG1/COL1A1/ITGB3/FGG/F2/FGA | 18 | BP |
| GO:0010876 | lipid localization | 19/397 | 0.00056025 | 0.00811673 | 0.00632247 | CEL/GALR1/LCN12/SLC10A2/SPX/ANGPTL3/BMP6/APOF/SLCO1A2/APOA4/ABCC2/APOA5/GAL/CES1/AKR1C4/APOC3/TAC1/AKR1C1/ITGB3 | 19 | BP |
| GO:0007271 | synaptic transmission, cholinergic | 4/397 | 0.00056867 | 0.00816913 | 0.00636328 | CHRNA3/CHRNB2/NQO1/TAC1 | 4 | BP |
| GO:0008542 | visual learning | 6/397 | 0.00056884 | 0.00816913 | 0.00636328 | NETO1/PPP1R1B/ADAM2/CHRNB2/GRIN2A/KIT | 6 | BP |
| GO:0002576 | platelet degranulation | 10/397 | 0.00056905 | 0.00816913 | 0.00636328 | SCG3/PPBP/TF/ORM2/ORM1/FGB/KNG1/ITGB3/FGG/FGA | 10 | BP |
| GO:0014823 | response to activity | 7/397 | 0.00057432 | 0.00821968 | 0.00640266 | BMP6/PCK1/TPH2/FGF21/GCLC/PPARGC1A/TH | 7 | BP |
| GO:0008194 | UDP-glycosyltransferase activity | 10/372 | 0.0009365 | 0.00831145 | 0.00637067 | B4GALNT2/UGT2A3/UGT2B11/UGT2B4/UGT1A3/UGT1A1/UGT1A4/UGT1A6/UGT1A7/UGT1A8 | 10 | MF |
| GO:0002065 | columnar/cuboidal epithelial cell differentiation | 9/397 | 0.0005833 | 0.00832298 | 0.00648313 | NKX3-2/BMP6/TP63/INSM1/HNF4A/RFX6/CDX2/ASCL1/NEUROD1 | 9 | BP |
| GO:0017171 | serine hydrolase activity | 11/372 | 0.00095415 | 0.00836357 | 0.00641062 | AZU1/ELANE/KLK3/PCSK2/F7/PRSS2/PLAT/PLAU/MMP3/PROC/F2 | 11 | MF |
| GO:0016324 | apical plasma membrane | 16/403 | 0.00085923 | 0.00848494 | 0.00663269 | CDHR2/KCNC2/KCNE4/KCNK2/SLC6A19/SLC7A9/ANO1/AQP1/CASR/GNAT1/TDGF1/UMOD/TF/MUC13/ABCC2/KL | 16 | CC |
| GO:0006066 | alcohol metabolic process | 18/397 | 0.00059841 | 0.00851292 | 0.00663108 | CEL/HAO1/MTMR7/ALDH1A3/ANGPTL3/BMP6/APOF/G6PD/PCK1/APOA4/PTH/AKR1B10/CES1/TTR/AKR1C4/AKR1C2/AKR1C1/AKR1C3 | 18 | BP |
| GO:0017134 | fibroblast growth factor binding | 4/372 | 0.00101255 | 0.00876716 | 0.00671997 | FGFBP1/KLB/KL/ITGB3 | 4 | MF |
| GO:0042440 | pigment metabolic process | 7/397 | 0.00063042 | 0.00894132 | 0.00696478 | OCA2/PPARGC1A/AMBP/UGT1A1/UGT1A4/UGT1A7/UGT1A8 | 7 | BP |
| GO:0007369 | gastrulation | 12/397 | 0.00063389 | 0.00896361 | 0.00698214 | HAND1/LHX1/HMGA2/COL12A1/HNF4A/TXNRD1/OTX2/COL5A1/UGDH/COL11A1/VTN/ITGB3 | 12 | BP |
| GO:0043069 | negative regulation of programmed cell death | 36/397 | 0.00064432 | 0.00908396 | 0.00707588 | MAG/NKX3-2/PAX7/PTGFR/AQP1/AZU1/NKX2-5/PRAME/TDGF1/ADORA1/CSF2/HMGA2/NEFL/SNAI2/FGF21/GDF5/GREM1/HAND2/SCG2/GCLC/GDNF/PPARGC1A/WNT7A/COMP/FGF4/PAX4/PTH/ASCL1/NEUROD1/NQO1/PROC/COL2A1/KIT/FGB/FGG/FGA | 36 | BP |
| GO:0043394 | proteoglycan binding | 5/372 | 0.00106274 | 0.00909091 | 0.00696813 | TNC/AZU1/COMP/COL5A1/COL2A1 | 5 | MF |
| GO:0008015 | blood circulation | 24/397 | 0.00066298 | 0.00931918 | 0.00725911 | GUCA2B/KCNE4/SPX/AZU1/CASR/GLP1R/KCNK3/MYL1/NKX2-5/ADORA1/FOXN4/CACNA1S/GCLC/COMP/CHGA/CES1/NTS/COL1A2/FGB/TAC1/KNG1/TH/FGG/FGA | 24 | BP |
| GO:0019216 | regulation of lipid metabolic process | 20/397 | 0.00068949 | 0.00965252 | 0.00751877 | CCKBR/ANGPTL3/BMP6/CGA/ADORA1/SNAI2/FGF21/HNF4A/TXNRD1/APOA4/PPARGC1A/APOA5/GAL/KIT/APOC3/AKR1C3/F2/UGT1A1/UGT1A8/CYP1A1 | 20 | BP |
| GO:0050918 | positive chemotaxis | 7/397 | 0.00069078 | 0.00965252 | 0.00751877 | CCL15/AZU1/CASR/F7/SCG2/GDNF/DEFB4A | 7 | BP |
| GO:0001502 | cartilage condensation | 4/397 | 0.00070779 | 0.0098321 | 0.00765864 | WNT7A/FGF4/COL11A1/COL2A1 | 4 | BP |
| GO:1902170 | cellular response to reactive nitrogen species | 4/397 | 0.00070779 | 0.0098321 | 0.00765864 | KCNC2/AQP1/MMP3/PPARGC1A | 4 | BP |
| GO:0048260 | positive regulation of receptor-mediated endocytosis | 6/397 | 0.00071377 | 0.00988604 | 0.00770066 | SFRP4/H1-1/GREM1/TF/APOA5/VTN | 6 | BP |
| GO:0048565 | digestive tract development | 10/397 | 0.0007241 | 0.00999984 | 0.0077893 | CCKBR/CLDN18/NKX3-2/ZIC3/RET/TP63/CDX2/ASCL1/KIT/CYP1A1 | 10 | BP |
| GO:0001906 | cell killing | 11/397 | 0.0007289 | 0.01003675 | 0.00781806 | CLEC2A/KLRF2/AZU1/CXCL6/ELANE/DEFA6/DEFA5/LCE3A/DEFB4A/CHGA/F2 | 11 | BP |
| GO:0010039 | response to iron ion | 5/397 | 0.00074255 | 0.01016538 | 0.00791825 | BMP6/G6PD/TF/TFF1/CYP1A1 | 5 | BP |
| GO:2000352 | negative regulation of endothelial cell apoptotic process | 5/397 | 0.00074255 | 0.01016538 | 0.00791825 | FGF21/SCG2/FGB/FGG/FGA | 5 | BP |
| GO:0051241 | negative regulation of multicellular organismal process | 46/397 | 0.00075857 | 0.01035471 | 0.00806573 | CLDN18/INHA/KCNK2/MAG/NKX3-2/SPX/ELANE/KLK3/LHX2/LMX1A/S100B/SOX3/SYT4/TP63/ADORA1/CALCA/G6PD/LIN28A/SNAI2/GDF5/GREM1/H4C1/H4C13/HAND2/PLAT/PLAU/APOA4/PHOX2B/WNT7A/H4C6/PTH/ASCL1/ASPN/COL5A1/GAL/ORM1/PROC/THBS2/VTN/APOC3/FGB/TAC1/KNG1/FGG/F2/FGA | 46 | BP |
| GO:0050880 | regulation of blood vessel size | 10/397 | 0.00076787 | 0.01045144 | 0.00814107 | CASR/ADORA1/GCLC/COMP/CHGA/NTS/FGB/KNG1/FGG/FGA | 10 | BP |
| GO:0007568 | aging | 17/397 | 0.00078129 | 0.01060355 | 0.00825956 | ENO3/TP63/HMGA2/PCK1/DDC/FOXG1/IGFBP1/ALDH3A1/GCLC/KRT14/PPARGC1A/WNT16/COMP/KL/NQO1/TH/CYP1A1 | 17 | BP |
| GO:0006952 | defense response | 57/397 | 0.00078758 | 0.01065825 | 0.00830217 | C8G/CD207/CLEC2A/CPN1/KLRF2/PTX3/VGF/WFDC12/AZU1/BMP6/CXCL6/DEFB126/ELANE/EREG/KLK3/RNASE7/S100B/SPAG11B/TDGF1/UMOD/ADORA1/BPIFA1/C8A/CCL1/CFHR5/DEFA6/BPIFA2/DEFA5/NT5E/PPBP/SCG2/APOA4/LCE3A/MMP3/MUC13/MUC19/MUC21/S100A7/DEFB1/DEFB4A/ORM2/CHGA/GAL/MUC6/ORM1/PROC/MUC2/MUC5AC/KIT/VTN/FGB/TAC1/KNG1/FGG/F2/UGT1A1/FGA | 57 | BP |
| GO:0001706 | endoderm formation | 6/397 | 0.00079617 | 0.01071313 | 0.00834491 | LHX1/HMGA2/COL12A1/COL5A1/COL11A1/VTN | 6 | BP |
| GO:0055081 | anion homeostasis | 6/397 | 0.00079617 | 0.01071313 | 0.00834491 | ANGPTL3/SFRP4/ADORA1/HNF4A/ABCC2/PTH | 6 | BP |
| GO:0035150 | regulation of tube size | 10/397 | 0.00081379 | 0.01078506 | 0.00840095 | CASR/ADORA1/GCLC/COMP/CHGA/NTS/FGB/KNG1/FGG/FGA | 10 | BP |
| GO:1904019 | epithelial cell apoptotic process | 9/397 | 0.00081484 | 0.01078506 | 0.00840095 | SFRP4/FGF21/SCG2/PPARGC1A/NEUROD1/FGB/FGG/AKR1C3/FGA | 9 | BP |
| GO:0043066 | negative regulation of apoptotic process | 35/397 | 0.00081911 | 0.01078506 | 0.00840095 | MAG/NKX3-2/PAX7/PTGFR/AQP1/AZU1/NKX2-5/PRAME/TDGF1/ADORA1/CSF2/HMGA2/NEFL/SNAI2/FGF21/GDF5/GREM1/HAND2/SCG2/GCLC/GDNF/PPARGC1A/WNT7A/COMP/FGF4/PAX4/PTH/ASCL1/NEUROD1/NQO1/PROC/COL2A1/FGB/FGG/FGA | 35 | BP |
| GO:0002138 | retinoic acid biosynthetic process | 3/397 | 0.00082207 | 0.01078506 | 0.00840095 | ALDH1A3/AKR1C3/CYP1A1 | 3 | BP |
| GO:0003211 | cardiac ventricle formation | 3/397 | 0.00082207 | 0.01078506 | 0.00840095 | HAND1/NKX2-5/HAND2 | 3 | BP |
| GO:0035933 | glucocorticoid secretion | 3/397 | 0.00082207 | 0.01078506 | 0.00840095 | GALR1/GAL/TAC1 | 3 | BP |
| GO:0042428 | serotonin metabolic process | 3/397 | 0.00082207 | 0.01078506 | 0.00840095 | TPH2/DDC/GRIN2A | 3 | BP |
| GO:0043589 | skin morphogenesis | 3/397 | 0.00082207 | 0.01078506 | 0.00840095 | TP63/COL1A2/COL1A1 | 3 | BP |
| GO:0061549 | sympathetic ganglion development | 3/397 | 0.00082207 | 0.01078506 | 0.00840095 | INSM1/PHOX2B/ASCL1 | 3 | BP |
| GO:0043195 | terminal bouton | 5/403 | 0.00112676 | 0.01089972 | 0.00852032 | GRIK3/KCNC2/ADORA1/SYP/TH | 5 | CC |
| GO:0003013 | circulatory system process | 24/397 | 0.00084 | 0.01098982 | 0.00856044 | GUCA2B/KCNE4/SPX/AZU1/CASR/GLP1R/KCNK3/MYL1/NKX2-5/ADORA1/FOXN4/CACNA1S/GCLC/COMP/CHGA/CES1/NTS/COL1A2/FGB/TAC1/KNG1/TH/FGG/FGA | 24 | BP |
| GO:0044306 | neuron projection terminus | 9/403 | 0.00117966 | 0.01118313 | 0.00874187 | GRIK3/KCNC2/KCNK2/CASR/KCNC1/SYT4/ADORA1/SYP/TH | 9 | CC |
| GO:0034505 | tooth mineralization | 4/397 | 0.00086938 | 0.01128039 | 0.00878678 | ODAPH/DMP1/ASPN/COL1A1 | 4 | BP |
| GO:0035929 | steroid hormone secretion | 4/397 | 0.00086938 | 0.01128039 | 0.00878678 | GALR1/BMP6/GAL/TAC1 | 4 | BP |
| GO:0098743 | cell aggregation | 4/397 | 0.00086938 | 0.01128039 | 0.00878678 | WNT7A/FGF4/COL11A1/COL2A1 | 4 | BP |
| GO:0009620 | response to fungus | 6/397 | 0.00088572 | 0.0113985 | 0.00887878 | PTX3/ELANE/RNASE7/DEFA6/DEFA5/CHGA | 6 | BP |
| GO:0021515 | cell differentiation in spinal cord | 6/397 | 0.00088572 | 0.0113985 | 0.00887878 | LHX1/PAX7/SOX1/FOXN4/PHOX2A/ASCL1 | 6 | BP |
| GO:0097366 | response to bronchodilator | 6/397 | 0.00088572 | 0.0113985 | 0.00887878 | KCNC2/PPP1R1B/AQP1/MMP3/GRIN2A/TH | 6 | BP |
| GO:0048871 | multicellular organismal homeostasis | 22/397 | 0.00089938 | 0.01154292 | 0.00899127 | ADGRV1/CLDN18/PTH2R/RHAG/TRPM8/AQP1/BMP6/TP63/ADORA1/BPIFA1/CALCA/FGF21/TF/MUC13/PHOX2B/PPARGC1A/PTH/MUC6/NEUROD1/MUC2/COL2A1/ITGB3 | 22 | BP |
| GO:1903532 | positive regulation of secretion by cell | 19/397 | 0.00090594 | 0.01156477 | 0.0090083 | GALR1/INHA/SYBU/VSNL1/ANO1/BMP6/CASR/SYT4/CCL1/CHRNB2/RFX6/GDNF/ORM2/GAL/ORM1/FGB/TAC1/FGG/FGA | 19 | BP |
| GO:0060548 | negative regulation of cell death | 38/397 | 0.00090598 | 0.01156477 | 0.0090083 | MAG/NKX3-2/PAX7/PTGFR/AQP1/AZU1/NKX2-5/PRAME/TDGF1/ADORA1/CSF2/HMGA2/NEFL/SNAI2/FGF21/GDF5/GREM1/HAND2/SCG2/GCLC/GDNF/PPARGC1A/WNT16/WNT7A/COMP/FGF4/PAX4/PTH/CHGA/ASCL1/NEUROD1/NQO1/PROC/COL2A1/KIT/FGB/FGG/FGA | 38 | BP |
| GO:0070851 | growth factor receptor binding | 9/372 | 0.00138925 | 0.01156729 | 0.00886626 | EREG/CSF2/FGF21/GREM1/KLB/FGF16/FGF5/FGF4/ITGB3 | 9 | MF |
| GO:0015111 | iodide transmembrane transporter activity | 2/372 | 0.0014174 | 0.01156729 | 0.00886626 | SLC5A5/ANO1 | 2 | MF |
| GO:0031731 | CCR6 chemokine receptor binding | 2/372 | 0.0014174 | 0.01156729 | 0.00886626 | DEFB1/DEFB4A | 2 | MF |
| GO:0099508 | voltage-gated ion channel activity involved in regulation of presynaptic membrane potential | 2/372 | 0.0014174 | 0.01156729 | 0.00886626 | KCNC2/KCNC1 | 2 | MF |
| GO:0098754 | detoxification | 9/397 | 0.00092623 | 0.01179132 | 0.00918476 | IYD/SRXN1/TXNRD1/APOA4/ABCC2/AKR1B10/GPX2/NQO1/GSTA1 | 9 | BP |
| GO:0090277 | positive regulation of peptide hormone secretion | 8/397 | 0.00092924 | 0.01179779 | 0.00918981 | SYBU/VSNL1/ANO1/CASR/RFX6/FGB/FGG/FGA | 8 | BP |
| GO:0000977 | RNA polymerase II regulatory region sequence-specific DNA binding | 25/372 | 0.0014746 | 0.01189736 | 0.00911925 | ESX1/GLIS1/HAND1/MYCN/ZIC3/LHX2/LMX1A/NKX2-5/SOX1/SOX3/HMGA2/INSM1/PHOX2A/PTF1A/SNAI2/HAND2/HNF4A/RFX6/OTX2/PHOX2B/PAX4/CDX2/ASCL1/NEUROD1/NEUROG3 | 25 | MF |
| GO:0003018 | vascular process in circulatory system | 11/397 | 0.00094581 | 0.01197608 | 0.00932868 | AZU1/CASR/ADORA1/GCLC/COMP/CHGA/NTS/FGB/KNG1/FGG/FGA | 11 | BP |
| GO:0030001 | metal ion transport | 32/397 | 0.00096251 | 0.01215005 | 0.00946419 | KCNC2/KCNK2/KCNU1/NETO1/SCN3A/SLC10A2/SLC17A2/SLC5A5/SPINK1/TRPM8/AQP1/CASR/KCNC1/KCNK3/KCNK9/NKX2-5/ABCC8/ADORA1/BPIFA1/CHRNB2/G6PD/CACNA1S/TF/CHRNA9/KCNQ2/KCNQ5/ABCC2/PTH/GRIN2A/GAL/SNAP25/F2 | 32 | BP |
| GO:0045595 | regulation of cell differentiation | 60/397 | 0.0009647 | 0.01215005 | 0.00946419 | ADGRV1/CLDN18/GLIS1/INHA/LHX1/MAG/NKX3-2/PAX7/BMP6/CXCL14/EREG/LHX2/LMX1A/NEUROD4/NKX2-5/PRAME/RET/S100B/SFRP4/SOX3/SYT4/TP63/WIF1/CCL17/CHRNA3/CHRNB2/CSF2/G6PD/INSM1/LIN28A/MMP11/NEFL/SNAI2/THPO/GDF5/GREM1/H4C1/H4C13/HAND2/GDNF/PHOX2B/WNT7A/H4C6/PAX4/PTH/CDX2/ASCL1/COL5A1/GAL/NEUROD1/NEUROG3/PROC/SNAP25/KIT/FGB/COL1A1/ITGB3/FGG/F2/FGA | 60 | BP |
| GO:0016903 | oxidoreductase activity, acting on the aldehyde or oxo group of donors | 5/372 | 0.00153821 | 0.01217897 | 0.0093351 | ALDH1A3/ALDH3A1/AKR1B10/AKR1C4/AKR1C3 | 5 | MF |
| GO:0001012 | RNA polymerase II regulatory region DNA binding | 25/372 | 0.00154381 | 0.01217897 | 0.0093351 | ESX1/GLIS1/HAND1/MYCN/ZIC3/LHX2/LMX1A/NKX2-5/SOX1/SOX3/HMGA2/INSM1/PHOX2A/PTF1A/SNAI2/HAND2/HNF4A/RFX6/OTX2/PHOX2B/PAX4/CDX2/ASCL1/NEUROD1/NEUROG3 | 25 | MF |
| GO:0030500 | regulation of bone mineralization | 7/397 | 0.00097947 | 0.01230331 | 0.00958358 | ADGRV1/OMD/BMP6/GREM1/COMP/KL/PTH | 7 | BP |
| GO:0007632 | visual behavior | 6/397 | 0.00098283 | 0.01231273 | 0.00959091 | NETO1/PPP1R1B/ADAM2/CHRNB2/GRIN2A/KIT | 6 | BP |
| GO:0046660 | female sex differentiation | 9/397 | 0.00098639 | 0.01232461 | 0.00960017 | INHA/LHX1/PTX3/VGF/ZFP42/EREG/TP63/KIT/AFP | 9 | BP |
| GO:0032127 | dense core granule membrane | 2/403 | 0.0013737 | 0.0125218 | 0.00978831 | SYT5/SYT4 | 2 | CC |
| GO:0071062 | alphav-beta3 integrin-vitronectin complex | 2/403 | 0.0013737 | 0.0125218 | 0.00978831 | VTN/ITGB3 | 2 | CC |
| GO:0051896 | regulation of protein kinase B signaling | 14/397 | 0.00101433 | 0.01264027 | 0.00984605 | NRG4/EREG/RET/F7/LIN28A/THPO/KLB/FGF16/FGF5/FGF4/KL/KIT/AKR1C2/AKR1C3 | 14 | BP |
| GO:0007548 | sex differentiation | 15/397 | 0.00102848 | 0.01278287 | 0.00995713 | INHA/LHX1/NR0B1/PTX3/SRY/VGF/ZFP42/BMP6/EREG/TP63/HNF4A/WNT7A/KIT/AFP/AKR1C3 | 15 | BP |
| GO:0098981 | cholinergic synapse | 3/403 | 0.00144671 | 0.0129385 | 0.01011404 | CHRNB2/NEFL/CHRNA9 | 3 | CC |
| GO:0071379 | cellular response to prostaglandin stimulus | 4/397 | 0.0010553 | 0.0130818 | 0.01018997 | PTGFR/TNC/AKR1C2/AKR1C3 | 4 | BP |
| GO:0032101 | regulation of response to external stimulus | 34/397 | 0.00106086 | 0.01311633 | 0.01021687 | C8G/CPN1/SPX/AZU1/BMP6/CASR/CXCL14/CXCL6/ELANE/GNAT1/GRK1/ADORA1/C8A/CCL1/CFHR5/F7/SNAI2/GREM1/NT5E/PLAT/PLAU/SCG2/FGF16/MMP3/S100A7/FGF4/PROC/VTN/FGB/TAC1/KNG1/FGG/F2/FGA | 34 | BP |
| GO:0035019 | somatic stem cell population maintenance | 7/397 | 0.00106467 | 0.01312902 | 0.01022676 | ZIC3/TDGF1/TP63/LIN28A/WNT7A/CDX2/KIT | 7 | BP |
| GO:0060348 | bone development | 12/397 | 0.00107198 | 0.01318476 | 0.01027018 | CLDN18/BMP6/SFRP4/THPO/GREM1/MMP13/COMP/FGF4/PTH/COL2A1/KIT/COL1A1 | 12 | BP |
| GO:0051384 | response to glucocorticoid | 10/397 | 0.00107857 | 0.01323139 | 0.0103065 | CPN1/AQP1/BMP6/S100B/NEFL/TPH2/ALDH3A1/ABCC2/TH/UGT1A1 | 10 | BP |
| GO:0043269 | regulation of ion transport | 27/397 | 0.00108348 | 0.01325714 | 0.01032655 | KCNC2/KCNU1/NETO1/SCN3A/SPINK1/CASR/KCNC1/NKX2-5/SFRP4/SYT4/ADORA1/BPIFA1/CHRNA3/CHRNB2/G6PD/KCNJ18/KCNK10/NEFL/CACNA1S/TF/GDNF/KCNQ2/KCNQ5/PPARGC1A/GRIN2A/GAL/F2 | 27 | BP |
| GO:0050994 | regulation of lipid catabolic process | 6/397 | 0.00108792 | 0.01327708 | 0.01034209 | ANGPTL3/ADORA1/FGF21/APOA4/APOA5/APOC3 | 6 | BP |
| GO:0033762 | response to glucagon | 5/397 | 0.00111703 | 0.01349339 | 0.01051058 | GLP2R/GLP1R/PCK1/FGF21/ABCC2 | 5 | BP |
| GO:0055090 | acylglycerol homeostasis | 5/397 | 0.00111703 | 0.01349339 | 0.01051058 | ANGPTL3/ADORA1/HNF4A/APOA5/APOC3 | 5 | BP |
| GO:0070328 | triglyceride homeostasis | 5/397 | 0.00111703 | 0.01349339 | 0.01051058 | ANGPTL3/ADORA1/HNF4A/APOA5/APOC3 | 5 | BP |
| GO:0060627 | regulation of vesicle-mediated transport | 22/397 | 0.00111707 | 0.01349339 | 0.01051058 | CPLX2/PTX3/STXBP5L/VSNL1/AZU1/SFRP4/SH3GL2/SYT4/CHRNA5/CHRNB2/H1-1/GREM1/TF/WNT7A/APOA5/SYP/VTN/APOC3/FGB/ITGB3/FGG/FGA | 22 | BP |
| GO:0055074 | calcium ion homeostasis | 19/397 | 0.00112171 | 0.0135149 | 0.01052733 | CALCB/CCKBR/CCL15/GALR1/TRPM8/CASR/ELANE/GLP1R/KCNK3/ADORA1/CALCA/CCL1/CHRNA9/KL/PTH/GRIN2A/TAC1/KNG1/F2 | 19 | BP |
| GO:0043679 | axon terminus | 8/403 | 0.00154531 | 0.01356443 | 0.01060333 | GRIK3/KCNC2/KCNK2/CASR/KCNC1/ADORA1/SYP/TH | 8 | CC |
| GO:0016102 | diterpenoid biosynthetic process | 3/397 | 0.00115513 | 0.01377694 | 0.01073145 | ALDH1A3/AKR1C3/CYP1A1 | 3 | BP |
| GO:0042415 | norepinephrine metabolic process | 3/397 | 0.00115513 | 0.01377694 | 0.01073145 | INSM1/HAND2/TH | 3 | BP |
| GO:0048484 | enteric nervous system development | 3/397 | 0.00115513 | 0.01377694 | 0.01073145 | RET/GDNF/PHOX2B | 3 | BP |
| GO:0060536 | cartilage morphogenesis | 3/397 | 0.00115513 | 0.01377694 | 0.01073145 | HAND1/SNAI2/HAND2 | 3 | BP |
| GO:0001704 | formation of primary germ layer | 9/397 | 0.00118611 | 0.01411088 | 0.01099157 | HAND1/LHX1/HMGA2/COL12A1/TXNRD1/COL5A1/COL11A1/VTN/ITGB3 | 9 | BP |
| GO:0007610 | behavior | 25/397 | 0.00119192 | 0.01414428 | 0.01101758 | KCNK2/NETO1/PPP1R1B/SRPX2/ADAM2/ALDH1A3/CASR/GLP1R/LMX1A/S100B/SYT4/ADORA1/CHRNA3/CHRNA5/CHRNB2/CSF2/KCNK10/HAND2/GDNF/GRIN2A/GAL/SNAP25/KIT/TAC1/TH | 25 | BP |
| GO:0051094 | positive regulation of developmental process | 48/397 | 0.00120695 | 0.01424753 | 0.01109801 | ADGRV1/LHX1/MAG/ODAPH/SRPX2/SRY/ANGPTL3/AQP1/BMP6/NEUROD4/NKX2-5/RET/S100B/SFRP4/SYT4/TP63/WIF1/CSF2/FGFBP1/HMGA2/INSM1/LIN28A/NEFL/SNAI2/THPO/GDF5/GREM1/HAND2/GDNF/OTX2/PHOX2B/PPARGC1A/WNT7A/KL/PAX4/PTH/CDX2/ASCL1/GAL/NEUROD1/NEUROG3/PROC/THBS2/KIT/FGB/COL1A1/FGG/FGA | 48 | BP |
| GO:0050793 | regulation of developmental process | 80/397 | 0.00120843 | 0.01424753 | 0.01109801 | ADGRV1/CLDN18/GLIS1/INHA/KCNK2/LHX1/MAG/NKX3-2/ODAPH/OMD/PAX7/SRPX2/SRY/ANGPTL3/AQP1/BMP6/CXCL14/ENAM/EREG/KLK3/LHX2/LMX1A/NEUROD4/NKX2-5/PRAME/RET/S100B/SFRP4/SOX3/SYT4/TP63/WIF1/CCL17/CHRNA3/CHRNB2/CSF2/FGFBP1/G6PD/HMGA2/INSM1/LIN28A/MMP11/NEFL/PTF1A/SNAI2/THPO/GDF5/GREM1/H4C1/H4C13/HAND2/HNF4A/DMP1/GDNF/OTX2/PHOX2B/PPARGC1A/WNT7A/COMP/H4C6/KL/PAX4/PTH/CDX2/ASCL1/ASPN/COL5A1/GAL/NEUROD1/NEUROG3/PROC/THBS2/SNAP25/KIT/FGB/COL1A1/ITGB3/FGG/F2/FGA | 80 | BP |
| GO:0002791 | regulation of peptide secretion | 22/397 | 0.00120967 | 0.01424753 | 0.01109801 | KCNC2/STXBP5L/SYBU/VSNL1/ANO1/BMP6/CASR/GLP1R/SYT4/ABCC8/ADORA1/CCL1/HNF4A/RFX6/KRT20/ORM2/NEUROD1/ORM1/SNAP25/FGB/FGG/FGA | 22 | BP |
| GO:0044212 | transcription regulatory region DNA binding | 29/372 | 0.00183766 | 0.01433781 | 0.01098984 | ESX1/GLIS1/HAND1/MYCN/ZIC3/FOXA3/LHX2/LMX1A/NKX2-5/SOX1/SOX3/TP63/FOXN4/HMGA2/INSM1/PHOX2A/PTF1A/SNAI2/HAND2/HNF4A/RFX6/OTX2/PHOX2B/PAX4/CDX2/ASCL1/HNF1A/NEUROD1/NEUROG3 | 29 | MF |
| GO:0001067 | regulatory region nucleic acid binding | 29/372 | 0.00187474 | 0.01446813 | 0.01108973 | ESX1/GLIS1/HAND1/MYCN/ZIC3/FOXA3/LHX2/LMX1A/NKX2-5/SOX1/SOX3/TP63/FOXN4/HMGA2/INSM1/PHOX2A/PTF1A/SNAI2/HAND2/HNF4A/RFX6/OTX2/PHOX2B/PAX4/CDX2/ASCL1/HNF1A/NEUROD1/NEUROG3 | 29 | MF |
| GO:0048732 | gland development | 20/397 | 0.0012459 | 0.01463778 | 0.011402 | CCKBR/NR0B1/TNC/ZIC3/ALDH1A3/NKX2-5/SOX3/TDGF1/TP63/FGL1/INSM1/PTF1A/SNAI2/HAND2/HNF4A/ASCL1/HNF1A/VTN/UGT1A1/CYP1A1 | 20 | BP |
| GO:0034705 | potassium channel complex | 7/403 | 0.00169849 | 0.01463788 | 0.01144244 | KCNC2/KCNK2/KCNC1/ABCC8/KCNQ2/KCNQ5/SNAP25 | 7 | CC |
| GO:0021516 | dorsal spinal cord development | 4/397 | 0.00126744 | 0.01479146 | 0.0115217 | LHX1/PAX7/GDNF/ASCL1 | 4 | BP |
| GO:0050927 | positive regulation of positive chemotaxis | 4/397 | 0.00126744 | 0.01479146 | 0.0115217 | AZU1/CASR/F7/SCG2 | 4 | BP |
| GO:0030574 | collagen catabolic process | 5/397 | 0.00126838 | 0.01479146 | 0.0115217 | MMP10/MMP11/PRSS2/MMP13/MMP3 | 5 | BP |
| GO:0035295 | tube development | 40/397 | 0.00127851 | 0.01487291 | 0.01158514 | CCKBR/CLDN18/EMX2/HAND1/LHX1/NKX3-2/PAX7/SRPX2/TNC/ZIC3/ANGPTL3/AQP1/CASR/COL8A2/EREG/KLK3/LHX2/NKX2-5/RET/TDGF1/TP63/UMOD/FGFBP1/FOXN4/HMGA2/TGFBI/GREM1/HAND2/SCG2/GDNF/S100A7/WNT7A/COMP/CDX2/ASCL1/THBS2/COL2A1/KIT/ITGB3/CYP1A1 | 40 | BP |
| GO:0015125 | bile acid transmembrane transporter activity | 3/372 | 0.00198513 | 0.01499405 | 0.01149285 | SLC10A2/SLCO1A2/AKR1C4 | 3 | MF |
| GO:0015464 | acetylcholine receptor activity | 3/372 | 0.00198513 | 0.01499405 | 0.01149285 | CHRNA3/CHRNA5/CHRNB2 | 3 | MF |
| GO:0061041 | regulation of wound healing | 10/397 | 0.00133782 | 0.0155246 | 0.01209278 | F7/PLAT/PLAU/PROC/VTN/FGB/KNG1/FGG/F2/FGA | 10 | BP |
| GO:0008289 | lipid binding | 26/372 | 0.00208385 | 0.01554072 | 0.01191186 | ACOT12/C8G/LCN12/MAG/PIK3C2G/SYT5/BPIFB2/BPIFB6/RNASE7/SH3GL2/SYT4/APOF/BPIFA1/BPIFA2/HNF4A/APOA4/APOA5/KL/SYP/CYP26A1/APOC3/UGT1A3/F2/UGT1A1/UGT1A7/UGT1A8 | 26 | MF |
| GO:0000976 | transcription regulatory region sequence-specific DNA binding | 26/372 | 0.0021286 | 0.01554072 | 0.01191186 | ESX1/GLIS1/HAND1/MYCN/ZIC3/LHX2/LMX1A/NKX2-5/SOX1/SOX3/FOXN4/HMGA2/INSM1/PHOX2A/PTF1A/SNAI2/HAND2/HNF4A/RFX6/OTX2/PHOX2B/PAX4/CDX2/ASCL1/NEUROD1/NEUROG3 | 26 | MF |
| GO:0005125 | cytokine activity | 10/372 | 0.0021376 | 0.01554072 | 0.01191186 | INHA/CXCL14/CXCL6/CCL1/CSF2/GREM2/THPO/GREM1/SCG2/WNT7A | 10 | MF |
| GO:0005518 | collagen binding | 6/372 | 0.00214506 | 0.01554072 | 0.01191186 | TGFBI/MMP13/COMP/ASPN/ITGA11/VTN | 6 | MF |
| GO:0008528 | G protein-coupled peptide receptor activity | 8/372 | 0.00217637 | 0.0156083 | 0.01196366 | CCKBR/GALR1/MLNR/NTSR2/SSTR1/CCKAR/GLP1R/GAL | 8 | MF |
| GO:0050708 | regulation of protein secretion | 21/397 | 0.00135607 | 0.01569789 | 0.01222776 | KCNC2/STXBP5L/SYBU/VSNL1/ANO1/BMP6/CASR/GLP1R/SYT4/ABCC8/CCL1/HNF4A/RFX6/KRT20/ORM2/NEUROD1/ORM1/SNAP25/FGB/FGG/FGA | 21 | BP |
| GO:0045834 | positive regulation of lipid metabolic process | 10/397 | 0.00140998 | 0.01602494 | 0.01248252 | ANGPTL3/BMP6/CGA/ADORA1/FGF21/APOA4/PPARGC1A/APOA5/KIT/F2 | 10 | BP |
| GO:0034220 | ion transmembrane transport | 38/397 | 0.00141351 | 0.01602494 | 0.01248252 | CLCA2/GRIK3/KCNC2/KCNE4/KCNK2/KCNU1/NETO1/RHAG/SCN3A/SLC17A2/SLC6A19/SLC7A9/TRPM8/ANO1/AQP1/ATP4A/CASR/KCNC1/KCNK3/UNC80/ABCC8/BPIFA1/G6PD/GABRA1/KCNJ18/KCNK10/NEFL/SLC18A3/CACNA1S/TF/CHRNA9/KCNQ2/KCNQ5/PPARGC1A/GRIN2A/GAL/SNAP25/F2 | 38 | BP |
| GO:0009416 | response to light stimulus | 16/397 | 0.00143774 | 0.01602494 | 0.01248252 | KCNC2/NETO1/PPP1R1B/RGR/ADAM2/AQP1/ELANE/GNAT1/GRK1/H2AW/KCNC1/CHRNB2/GRIN2A/IVL/KIT/TH | 16 | BP |
| GO:0090596 | sensory organ morphogenesis | 14/397 | 0.00143777 | 0.01602494 | 0.01248252 | LHX1/NKX3-2/ALDH1A3/COL8A2/GNAT1/SOX1/FOXN4/PTF1A/CHRNA9/WNT16/COL5A1/COL11A1/COL2A1/TH | 14 | BP |
| GO:0001550 | ovarian cumulus expansion | 2/397 | 0.00144879 | 0.01602494 | 0.01248252 | PTX3/EREG | 2 | BP |
| GO:0003219 | cardiac right ventricle formation | 2/397 | 0.00144879 | 0.01602494 | 0.01248252 | HAND1/HAND2 | 2 | BP |
| GO:0016095 | polyprenol catabolic process | 2/397 | 0.00144879 | 0.01602494 | 0.01248252 | AKR1B10/AKR1C3 | 2 | BP |
| GO:0033076 | isoquinoline alkaloid metabolic process | 2/397 | 0.00144879 | 0.01602494 | 0.01248252 | DDC/TH | 2 | BP |
| GO:0042427 | serotonin biosynthetic process | 2/397 | 0.00144879 | 0.01602494 | 0.01248252 | TPH2/DDC | 2 | BP |
| GO:0046878 | positive regulation of saliva secretion | 2/397 | 0.00144879 | 0.01602494 | 0.01248252 | AQP1/TAC1 | 2 | BP |
| GO:0048165 | fused antrum stage | 2/397 | 0.00144879 | 0.01602494 | 0.01248252 | PTX3/EREG | 2 | BP |
| GO:0060066 | oviduct development | 2/397 | 0.00144879 | 0.01602494 | 0.01248252 | LHX1/WNT7A | 2 | BP |
| GO:0060084 | synaptic transmission involved in micturition | 2/397 | 0.00144879 | 0.01602494 | 0.01248252 | CHRNA3/CHRNB2 | 2 | BP |
| GO:0060166 | olfactory pit development | 2/397 | 0.00144879 | 0.01602494 | 0.01248252 | ALDH1A3/ASCL1 | 2 | BP |
| GO:0070173 | regulation of enamel mineralization | 2/397 | 0.00144879 | 0.01602494 | 0.01248252 | ODAPH/DMP1 | 2 | BP |
| GO:0099183 | trans-synaptic signaling by BDNF, modulating synaptic transmission | 2/397 | 0.00144879 | 0.01602494 | 0.01248252 | SYT4/PLAT | 2 | BP |
| GO:0099191 | trans-synaptic signaling by BDNF | 2/397 | 0.00144879 | 0.01602494 | 0.01248252 | SYT4/PLAT | 2 | BP |
| GO:1900157 | regulation of bone mineralization involved in bone maturation | 2/397 | 0.00144879 | 0.01602494 | 0.01248252 | GREM1/PTH | 2 | BP |
| GO:2001260 | regulation of semaphorin-plexin signaling pathway | 2/397 | 0.00144879 | 0.01602494 | 0.01248252 | HAND2/GDNF | 2 | BP |
| GO:0042627 | chylomicron | 3/403 | 0.00189822 | 0.01606706 | 0.01255963 | APOA4/APOA5/APOC3 | 3 | CC |
| GO:0098685 | Schaffer collateral - CA1 synapse | 7/403 | 0.00195952 | 0.01629495 | 0.01273778 | LRFN2/SH3GL2/NEFL/CDH11/PLAT/WNT7A/SYP | 7 | CC |
| GO:0006810 | transport | 133/397 | 0.0014833 | 0.01635191 | 0.01273721 | AQP12A/CA6/CCKBR/CD207/CEL/CLCA2/CPLX2/GALR1/GRIK3/GUCA2B/HAO1/INHA/KCNC2/KCNE4/KCNK2/KCNU1/KLRF2/LCN12/NETO1/PLPP4/PTX3/RHAG/SCN3A/SLC10A2/SLC17A2/SLC5A5/SLC6A19/SLC7A9/SPINK1/SPX/STXBP5L/SYBU/TRPM8/VGF/VSNL1/ZG16/ANO1/AOC1/AQP1/ATP4A/AZU1/BMP6/CASR/ELANE/GLP1R/KCNC1/KCNK3/KCNK9/KIF5C/NKX2-5/OCA2/SFRP4/SH3GL2/SYT4/TP63/UNC80/ABCC8/ADORA1/APOF/BPIFA1/CCL1/CDH17/CHRNA3/CHRNA5/CHRNB2/CSF2/F7/G6PD/GABRA1/H1-1/KCNJ18/KCNK10/KIF1A/NEFL/PRSS2/SCG3/SLC18A3/SLCO1A2/CACNA1S/DDC/FGF21/G6PC/GREM1/HNF4A/PLAU/PPBP/RFX6/SCG2/TF/APOA4/CHAT/CHRNA9/GDNF/KCNQ2/KCNQ5/PPARGC1A/S100A7/WNT7A/ABCC2/APOA5/COMP/KRT20/ORM2/PTH/CDX2/CHGA/GRIN2A/CALML5/GAL/HNF1A/NEUROD1/ORM1/PROC/SERPINA7/SYP/CES1/SNAP25/KIT/TTR/AKR1C4/AMBP/VTN/APOC3/FGB/TAC1/KNG1/TH/AKR1C1/COL1A1/ITGB3/FGG/F2/FGA | 133 | BP |
| GO:0048592 | eye morphogenesis | 10/397 | 0.00148528 | 0.01635191 | 0.01273721 | LHX1/ALDH1A3/COL8A2/GNAT1/SOX1/FOXN4/PTF1A/WNT16/COL5A1/TH | 10 | BP |
| GO:0001653 | peptide receptor activity | 8/372 | 0.00231014 | 0.01640199 | 0.01257202 | CCKBR/GALR1/MLNR/NTSR2/SSTR1/CCKAR/GLP1R/GAL | 8 | MF |
| GO:0021549 | cerebellum development | 8/397 | 0.00150523 | 0.01648326 | 0.01283952 | LHX1/SSTR1/KCNC1/LMX1A/PTF1A/PPARGC1A/WNT7A/NEUROD1 | 8 | BP |
| GO:0050926 | regulation of positive chemotaxis | 4/397 | 0.00150768 | 0.01648326 | 0.01283952 | AZU1/CASR/F7/SCG2 | 4 | BP |
| GO:0051004 | regulation of lipoprotein lipase activity | 4/397 | 0.00150768 | 0.01648326 | 0.01283952 | ANGPTL3/APOA4/APOA5/APOC3 | 4 | BP |
| GO:0009743 | response to carbohydrate | 13/397 | 0.00153093 | 0.01669882 | 0.01300743 | SYBU/VSNL1/ANO1/CASR/LIN28A/PCK1/FGF21/HNF4A/RFX6/GCLC/PPARGC1A/NEUROD1/TH | 13 | BP |
| GO:0044456 | synapse part | 32/403 | 0.00204423 | 0.01670632 | 0.01305935 | CEL/GRIK3/KCNC2/KCNK2/LRFN2/NETO1/PPP1R1B/RAB3C/SRPX2/CASR/GABRG3/KCNC1/KCNK9/SH3GL2/SYT4/ADORA1/CHRNA3/CHRNA5/CHRNB2/GABRA1/KIF1A/NEFL/DDC/CDH9/CHAT/CHRNA9/GABBR2/WNT7A/GRIN2A/SYP/SNAP25/TH | 32 | CC |
| GO:0050840 | extracellular matrix binding | 5/372 | 0.00239438 | 0.01683177 | 0.01290144 | TGFBI/DMP1/COL11A1/VTN/ITGB3 | 5 | MF |
| GO:0008021 | synaptic vesicle | 10/403 | 0.00210505 | 0.01691177 | 0.01321995 | RAB3C/KCNK9/SH3GL2/SYT4/KIF1A/DDC/GRIN2A/SYP/SNAP25/TH | 10 | CC |
| GO:0015711 | organic anion transport | 20/397 | 0.0015577 | 0.01695166 | 0.01320437 | CA6/LCN12/RHAG/SLC10A2/SLC6A19/SLC7A9/SPX/AQP1/CASR/OCA2/SYT4/ADORA1/SLCO1A2/G6PC/APOA4/ABCC2/SNAP25/AKR1C4/APOC3/AKR1C1 | 20 | BP |
| GO:0034372 | very-low-density lipoprotein particle remodeling | 3/397 | 0.00156229 | 0.01696252 | 0.01321284 | APOA4/APOA5/APOC3 | 3 | BP |
| GO:0051851 | modification by host of symbiont morphology or physiology | 7/397 | 0.00158165 | 0.01713338 | 0.01334593 | PTX3/AQP1/AZU1/CXCL6/ELANE/HMGA2/F2 | 7 | BP |
| GO:0034364 | high-density lipoprotein particle | 4/403 | 0.00219249 | 0.01732069 | 0.0135396 | APOF/APOA4/APOA5/APOC3 | 4 | CC |
| GO:0001228 | DNA-binding transcription activator activity, RNA polymerase II-specific | 18/372 | 0.00252151 | 0.01755169 | 0.01345326 | GLIS1/MYCN/ZIC3/LHX2/LMX1A/NKX2-5/SOX1/TP63/HMGA2/PTF1A/HAND2/HNF4A/RFX6/OTX2/PHOX2B/HNF1A/NEUROD1/NEUROG3 | 18 | MF |
| GO:0071772 | response to BMP | 10/397 | 0.00164568 | 0.01774551 | 0.01382274 | TNMD/BMP6/NKX2-5/SFRP4/GREM2/GDF5/GREM1/PHOX2B/COMP/COL2A1 | 10 | BP |
| GO:0071773 | cellular response to BMP stimulus | 10/397 | 0.00164568 | 0.01774551 | 0.01382274 | TNMD/BMP6/NKX2-5/SFRP4/GREM2/GDF5/GREM1/PHOX2B/COMP/COL2A1 | 10 | BP |
| GO:0004090 | carbonyl reductase (NADPH) activity | 2/372 | 0.00279366 | 0.01860171 | 0.01425809 | CBR3/CBR1 | 2 | MF |
| GO:0004771 | sterol esterase activity | 2/372 | 0.00279366 | 0.01860171 | 0.01425809 | CEL/CES1 | 2 | MF |
| GO:0004966 | galanin receptor activity | 2/372 | 0.00279366 | 0.01860171 | 0.01425809 | GALR1/GAL | 2 | MF |
| GO:0022841 | potassium ion leak channel activity | 2/372 | 0.00279366 | 0.01860171 | 0.01425809 | KCNK2/KCNK3 | 2 | MF |
| GO:0004497 | monooxygenase activity | 7/372 | 0.00280336 | 0.01860171 | 0.01425809 | PAH/CYP26A1/AKR1C2/TH/AKR1C1/AKR1C3/CYP1A1 | 7 | MF |
| GO:0007623 | circadian rhythm | 12/397 | 0.00181107 | 0.01935829 | 0.015079 | PASD1/PIWIL2/ADORA1/CHRNB2/CSF2/F7/TPH2/DDC/HNF4A/PPARGC1A/PAX4/TH | 12 | BP |
| GO:0050900 | leukocyte migration | 21/397 | 0.00181277 | 0.01935829 | 0.015079 | MAG/SLC7A9/AZU1/CXCL14/CXCL6/ELANE/RET/UMOD/ADORA1/CALCA/CCL1/F7/GREM1/SCG2/S100A7/CHGA/KIT/COL1A2/COL1A1/ITGB3/F2 | 21 | BP |
| GO:0019835 | cytolysis | 5/397 | 0.00181351 | 0.01935829 | 0.015079 | C8G/C8A/CFHR5/CSF2/F2 | 5 | BP |
| GO:0050832 | defense response to fungus | 5/397 | 0.00181351 | 0.01935829 | 0.015079 | ELANE/RNASE7/DEFA6/DEFA5/CHGA | 5 | BP |
| GO:0055065 | metal ion homeostasis | 23/397 | 0.00181796 | 0.01935829 | 0.015079 | CALCB/CCKBR/CCL15/GALR1/RHAG/SPX/TRPM8/BMP6/CASR/ELANE/GLP1R/KCNK3/ADORA1/CALCA/CCL1/TF/CHRNA9/KL/PTH/GRIN2A/TAC1/KNG1/F2 | 23 | BP |
| GO:0007269 | neurotransmitter secretion | 10/397 | 0.00181984 | 0.01935829 | 0.015079 | STXBP5L/SYT4/CHRNA3/CHRNA5/CHRNB2/SLC18A3/CHAT/WNT7A/SYP/SNAP25 | 10 | BP |
| GO:0097756 | negative regulation of blood vessel diameter | 7/397 | 0.00183605 | 0.01948687 | 0.01517916 | CASR/ADORA1/COMP/CHGA/FGB/FGG/FGA | 7 | BP |
| GO:0043005 | neuron projection | 38/403 | 0.00251774 | 0.01956407 | 0.01529325 | ADGRV1/CTNND2/DPYSL5/GRIK3/GUCA2B/INHA/KCNC2/KCNK2/MAG/NMNAT2/PPP1R1B/SYBU/SYT5/CASR/GNAT1/GRK1/KCNC1/KIF5C/PCSK2/RET/SH3GL2/SYT4/TP63/ADORA1/CHRNA3/KIF1A/NEFL/DDC/GABBR2/KCNQ2/OTX2/PPARGC1A/GRIN2A/NQO1/SYP/SNAP25/TAC1/TH | 38 | CC |
| GO:0048584 | positive regulation of response to stimulus | 72/397 | 0.00184905 | 0.01958083 | 0.01525235 | ADGRV1/C8G/CPN1/GALR1/IRS4/KCNK2/NETO1/NRG4/PLPP4/SYBU/VSNL1/ANO1/AZU1/BMP6/CASR/CXCL14/ELANE/EREG/FZD10/RET/S100B/SFRP4/TDGF1/TP63/ADORA1/C8A/CCL1/CFHR5/CHRNB2/CSF2/F7/FGFBP1/HMGA2/LIN28A/THPO/FGF21/GDF5/GREM1/HAND2/KLB/RFX6/SCG2/FGF16/FGF5/GDNF/MMP3/MUC13/MUC19/MUC21/S100A7/WNT16/WNT7A/FGF4/KL/PTH/CHGA/ASCL1/GAL/MUC6/MUC2/MUC5AC/KIT/VTN/AKR1C2/FGB/TAC1/COL1A1/ITGB3/FGG/AKR1C3/F2/FGA | 72 | BP |
| GO:0045177 | apical part of cell | 17/403 | 0.00257333 | 0.01967354 | 0.01537882 | CDHR2/KCNC2/KCNE4/KCNK2/SLC6A19/SLC7A9/ANO1/AQP1/CASR/GNAT1/TDGF1/UMOD/PLAT/TF/MUC13/ABCC2/KL | 17 | CC |
| GO:0006874 | cellular calcium ion homeostasis | 18/397 | 0.00187031 | 0.01976165 | 0.0153932 | CALCB/CCKBR/CCL15/GALR1/TRPM8/CASR/ELANE/GLP1R/KCNK3/ADORA1/CALCA/CCL1/CHRNA9/PTH/GRIN2A/TAC1/KNG1/F2 | 18 | BP |
| GO:2000351 | regulation of endothelial cell apoptotic process | 6/397 | 0.00191052 | 0.02011575 | 0.01566902 | FGF21/SCG2/FGB/FGG/AKR1C3/FGA | 6 | BP |
| GO:0099643 | signal release from synapse | 10/397 | 0.00191234 | 0.02011575 | 0.01566902 | STXBP5L/SYT4/CHRNA3/CHRNA5/CHRNB2/SLC18A3/CHAT/WNT7A/SYP/SNAP25 | 10 | BP |
| GO:0016491 | oxidoreductase activity | 26/372 | 0.00308665 | 0.02029186 | 0.01555358 | FADS6/HAO1/IYD/PIR/ALDH1A3/AOC1/GLDC/G6PD/SRXN1/TXNRD1/ALDH3A1/PAH/AKR1B10/GPX2/NQO1/UGDH/GSTA1/AKR1C4/CBR3/CYP26A1/AKR1C2/CBR1/TH/AKR1C1/AKR1C3/CYP1A1 | 26 | MF |
| GO:0043565 | sequence-specific DNA binding | 33/372 | 0.00311524 | 0.0202919 | 0.01555361 | EMX2/ESX1/GLIS1/HAND1/MYCN/NR0B1/PAX7/ZIC3/FOXA3/LHX2/LMX1A/NKX2-5/SOX1/SOX3/TP63/FOXN4/HMGA2/INSM1/PHOX2A/PTF1A/SNAI2/FOXG1/HAND2/HNF4A/RFX6/OTX2/PHOX2B/PPARGC1A/PAX4/CDX2/ASCL1/NEUROD1/NEUROG3 | 33 | MF |
| GO:0048511 | rhythmic process | 15/397 | 0.00194827 | 0.02044821 | 0.01592799 | PASD1/PTX3/EREG/PIWIL2/ADORA1/CHRNB2/CSF2/F7/TPH2/DDC/HNF4A/PPARGC1A/PAX4/AFP/TH | 15 | BP |
| GO:0045178 | basal part of cell | 5/403 | 0.0027434 | 0.0206408 | 0.01613494 | CLCA2/GKN2/AQP1/TF/KRT14 | 5 | CC |
| GO:0045055 | regulated exocytosis | 30/397 | 0.00197831 | 0.02071054 | 0.01613233 | CPLX2/KLRF2/PTX3/STXBP5L/AOC1/AZU1/ELANE/SYT4/CHRNA5/CHRNB2/PRSS2/SCG3/PLAU/PPBP/TF/S100A7/WNT7A/ORM2/CHGA/CALML5/ORM1/SYP/SNAP25/KIT/TTR/FGB/KNG1/ITGB3/FGG/FGA | 30 | BP |
| GO:0098771 | inorganic ion homeostasis | 25/397 | 0.00198204 | 0.02071054 | 0.01613233 | CALCB/CCKBR/CCL15/GALR1/RHAG/SPX/TRPM8/BMP6/CASR/ELANE/GLP1R/KCNK3/SFRP4/ADORA1/CALCA/CCL1/TF/CHRNA9/ABCC2/KL/PTH/GRIN2A/TAC1/KNG1/F2 | 25 | BP |
| GO:2001236 | regulation of extrinsic apoptotic signaling pathway | 10/397 | 0.0020086 | 0.02094176 | 0.01631244 | RET/CSF2/SNAI2/SCG2/GCLC/GDNF/COL2A1/FGB/FGG/FGA | 10 | BP |
| GO:0021953 | central nervous system neuron differentiation | 11/397 | 0.00202535 | 0.0210551 | 0.01640072 | LHX1/PAX7/LMX1A/SOX1/CHRNB2/FOXN4/PHOX2A/CDH11/PHOX2B/WNT7A/ASCL1 | 11 | BP |
| GO:0090184 | positive regulation of kidney development | 5/397 | 0.00202839 | 0.0210551 | 0.01640072 | LHX1/RET/LIN28A/GREM1/GDNF | 5 | BP |
| GO:0003207 | cardiac chamber formation | 3/397 | 0.00204898 | 0.02109301 | 0.01643025 | HAND1/NKX2-5/HAND2 | 3 | BP |
| GO:0072488 | ammonium transmembrane transport | 3/397 | 0.00204898 | 0.02109301 | 0.01643025 | RHAG/AQP1/SLC18A3 | 3 | BP |
| GO:1901160 | primary amino compound metabolic process | 3/397 | 0.00204898 | 0.02109301 | 0.01643025 | TPH2/DDC/GRIN2A | 3 | BP |
| GO:0048705 | skeletal system morphogenesis | 12/397 | 0.0020499 | 0.02109301 | 0.01643025 | LHX1/NKX3-2/BMP6/SFRP4/GREM1/MMP13/WNT7A/COMP/FGF4/COL11A1/COL2A1/COL1A1 | 12 | BP |
| GO:0003309 | type B pancreatic cell differentiation | 4/397 | 0.00207991 | 0.02125742 | 0.01655832 | BMP6/INSM1/HNF4A/RFX6 | 4 | BP |
| GO:0010669 | epithelial structure maintenance | 4/397 | 0.00207991 | 0.02125742 | 0.01655832 | MUC13/MUC6/NEUROD1/MUC2 | 4 | BP |
| GO:0097066 | response to thyroid hormone | 4/397 | 0.00207991 | 0.02125742 | 0.01655832 | F7/GCLC/PPARGC1A/KIT | 4 | BP |
| GO:0016266 | O-glycan processing | 6/397 | 0.00208388 | 0.02125742 | 0.01655832 | MUC13/MUC19/MUC21/MUC6/MUC2/MUC5AC | 6 | BP |
| GO:0040017 | positive regulation of locomotion | 24/397 | 0.00211518 | 0.02140606 | 0.0166741 | SRPX2/ANGPTL3/AZU1/CASR/CGA/CXCL14/ELANE/RET/TDGF1/CCL1/F7/FGFBP1/INSM1/SNAI2/PLAU/SCG2/FGF16/S100A7/WNT7A/DEFB1/VTN/TAC1/COL1A1/ITGB3 | 24 | BP |
| GO:0015696 | ammonium transport | 7/397 | 0.00212108 | 0.02140606 | 0.0166741 | RHAG/AQP1/CHRNA3/CHRNB2/CSF2/SLC18A3/GDNF | 7 | BP |
| GO:0046849 | bone remodeling | 7/397 | 0.00212108 | 0.02140606 | 0.0166741 | CLDN18/CALCA/GDF5/GREM1/WNT16/PTH/ITGB3 | 7 | BP |
| GO:0051702 | interaction with symbiont | 7/397 | 0.00212108 | 0.02140606 | 0.0166741 | PTX3/AQP1/AZU1/CXCL6/ELANE/HMGA2/F2 | 7 | BP |
| GO:0008219 | cell death | 68/397 | 0.00212112 | 0.02140606 | 0.0166741 | KCNK2/MAG/NKX3-2/PAX7/PTGFR/ALDH1A3/AQP1/AZU1/CASR/DSC3/NKX2-5/PRAME/RET/S100B/SFRP4/TDGF1/TP63/ADORA1/CSF2/G6PD/HMGA2/NEFL/SNAI2/FGF21/GDF5/GREM1/HAND2/KRT5/KRT6B/SCG2/GCLC/GDNF/KRT14/MMP3/PPARGC1A/WNT16/WNT7A/COMP/FGF4/KRT20/PAX4/PTH/CHGA/GRIN2A/ASCL1/DSG3/GAL/IVL/NEUROD1/NQO1/PROC/SPRR2A/SPRR2F/SPRR2G/SPRR2B/SPRR2E/COL2A1/KIT/SPRR2D/SPRR3/SPRR1A/FGB/SPRR1B/KNG1/FGG/SST/AKR1C3/FGA | 68 | BP |
| GO:0021700 | developmental maturation | 14/397 | 0.00222329 | 0.02236364 | 0.01742 | SPINK1/SYBU/EREG/RET/SYT4/G6PD/NEFL/GREM1/DEFB1/PTH/ASCL1/GAL/SYP/FGG | 14 | BP |
| GO:0072507 | divalent inorganic cation homeostasis | 19/397 | 0.00222547 | 0.02236364 | 0.01742 | CALCB/CCKBR/CCL15/GALR1/TRPM8/CASR/ELANE/GLP1R/KCNK3/ADORA1/CALCA/CCL1/CHRNA9/KL/PTH/GRIN2A/TAC1/KNG1/F2 | 19 | BP |
| GO:0005231 | excitatory extracellular ligand-gated ion channel activity | 4/372 | 0.0035003 | 0.02259284 | 0.01731727 | CHRNA3/CHRNA5/CHRNB2/CHRNA9 | 4 | MF |
| GO:0033627 | cell adhesion mediated by integrin | 6/397 | 0.00226887 | 0.02275129 | 0.01772196 | RET/SNAI2/PLAU/ITGA11/VTN/ITGB3 | 6 | BP |
| GO:0015672 | monovalent inorganic cation transport | 21/397 | 0.00233558 | 0.02337065 | 0.01820441 | KCNC2/KCNK2/KCNU1/NETO1/RHAG/SCN3A/SLC10A2/SLC17A2/SLC5A5/AQP1/KCNC1/KCNK3/KCNK9/NKX2-5/ABCC8/ADORA1/BPIFA1/KCNQ2/KCNQ5/GAL/SNAP25 | 21 | BP |
| GO:0055085 | transmembrane transport | 49/397 | 0.00237886 | 0.02375344 | 0.01850258 | AQP12A/CLCA2/GRIK3/KCNC2/KCNE4/KCNK2/KCNU1/NETO1/RHAG/SCN3A/SLC10A2/SLC17A2/SLC5A5/SLC6A19/SLC7A9/TRPM8/ANO1/AQP1/ATP4A/CASR/KCNC1/KCNK3/OCA2/UNC80/ABCC8/BPIFA1/CDH17/G6PD/GABRA1/KCNJ18/KCNK10/NEFL/SLC18A3/SLCO1A2/CACNA1S/FGF21/PPBP/TF/CHRNA9/KCNQ2/KCNQ5/PPARGC1A/ABCC2/PTH/GRIN2A/GAL/HNF1A/SNAP25/F2 | 49 | BP |
| GO:0051604 | protein maturation | 17/397 | 0.00239218 | 0.02383597 | 0.01856686 | C8G/CPN1/CGA/PCSK2/C8A/CFHR5/F7/PLAT/PLAU/COMP/GRIN2A/CES1/VTN/FGB/FGG/F2/FGA | 17 | BP |
| GO:0002376 | immune system process | 90/397 | 0.00239741 | 0.02383787 | 0.01856834 | C8G/CD207/CLDN18/CLEC2A/CPLX2/CPN1/FCER1A/INHA/KLRF2/MAG/NKX3-2/PIR/PLPP4/PTX3/RAB3C/RHAG/SLC7A9/AOC1/AZU1/BMP6/BPIFB2/CD1A/CXCL14/CXCL6/DEFB126/ELANE/EREG/KLK3/NKX2-5/RET/RNASE7/S100B/SH3GL2/TDGF1/UMOD/ADORA1/BPIFA1/C8A/CALCA/CCL1/CDH17/CFHR5/CHRNB2/CSF2/DEFA6/F7/FGL1/G6PD/PRSS2/THPO/BPIFA2/DEFA5/GREM1/H4C1/H4C13/HAND2/PLAU/PPBP/SCG2/APOA4/MUC13/MUC19/MUC21/S100A7/DEFB1/DEFB4A/H4C6/ORM2/CHGA/CALML5/GAL/HNF1A/MUC6/ORM1/MUC2/MUC5AC/SNAP25/COL2A1/KIT/TTR/AMBP/COL1A2/VTN/FGB/TAC1/COL1A1/ITGB3/FGG/F2/FGA | 90 | BP |
| GO:0051186 | cofactor metabolic process | 23/397 | 0.002412 | 0.0238677 | 0.01859157 | ACOT12/NMNAT2/ENO3/OGDHL/G6PD/GPT2/GSTA2/NT5E/APOA4/GCLC/MMP3/PPARGC1A/AKR1B10/GSTA1/AKR1C4/AMBP/CBR3/AKR1C2/AKR1C1/AKR1C3/UGT1A1/UGT1A4/CYP1A1 | 23 | BP |
| GO:0060351 | cartilage development involved in endochondral bone morphogenesis | 4/397 | 0.00241557 | 0.0238677 | 0.01859157 | MMP13/COMP/COL2A1/COL1A1 | 4 | BP |
| GO:0071377 | cellular response to glucagon stimulus | 4/397 | 0.00241557 | 0.0238677 | 0.01859157 | GLP2R/GLP1R/PCK1/FGF21 | 4 | BP |
| GO:0022414 | reproductive process | 46/397 | 0.00246193 | 0.02427492 | 0.01890877 | ADAM20/CLGN/GTSF1/HAND1/INHA/KCNU1/LHX1/NR0B1/PPP1R1B/PSG2/PSG4/PTGFR/PTX3/SPINK1/SRY/SSTR1/TNC/VGF/ZFP42/ADAM2/BMP6/DEFB126/EREG/FOXA3/OCA2/PIWIL2/SOX3/SPAG11B/TAC3/TAF1L/TP63/CALCA/CSF2/LIN28A/HNF4A/WNT7A/ABCC2/DEFB1/CDX2/KIT/AFP/AMBP/TAC1/TH/AKR1C3/CYP1A1 | 46 | BP |
| GO:0043200 | response to amino acid | 8/397 | 0.00247663 | 0.02435125 | 0.01896823 | F7/PCK1/FGF21/GCLC/PPARGC1A/COL1A2/COL1A1/SST | 8 | BP |
| GO:0000003 | reproduction | 46/397 | 0.0024972 | 0.02435125 | 0.01896823 | ADAM20/CLGN/GTSF1/HAND1/INHA/KCNU1/LHX1/NR0B1/PPP1R1B/PSG2/PSG4/PTGFR/PTX3/SPINK1/SRY/SSTR1/TNC/VGF/ZFP42/ADAM2/BMP6/DEFB126/EREG/FOXA3/OCA2/PIWIL2/SOX3/SPAG11B/TAC3/TAF1L/TP63/CALCA/CSF2/LIN28A/HNF4A/WNT7A/ABCC2/DEFB1/CDX2/KIT/AFP/AMBP/TAC1/TH/AKR1C3/CYP1A1 | 46 | BP |
| GO:0010647 | positive regulation of cell communication | 56/397 | 0.00251168 | 0.02435125 | 0.01896823 | ADGRV1/GALR1/INHA/IRS4/NETO1/NRG4/SYBU/VSNL1/ANO1/BMP6/CASR/ELANE/EREG/FZD10/RET/S100B/SFRP4/TDGF1/TP63/ADORA1/CHRNB2/CSF2/F7/FGFBP1/LIN28A/THPO/FGF21/GDF5/GREM1/HAND2/KLB/RFX6/FGF16/FGF5/S100A7/WNT16/WNT7A/FGF4/KL/PTH/CHGA/GRIN2A/ASCL1/GAL/SNAP25/KIT/VTN/AKR1C2/FGB/TAC1/COL1A1/ITGB3/FGG/AKR1C3/F2/FGA | 56 | BP |
| GO:0035094 | response to nicotine | 5/397 | 0.00251303 | 0.02435125 | 0.01896823 | PPP1R1B/CHRNA3/CHRNA5/CHRNB2/TH | 5 | BP |
| GO:0044060 | regulation of endocrine process | 5/397 | 0.00251303 | 0.02435125 | 0.01896823 | GALR1/INHA/BMP6/GAL/TAC1 | 5 | BP |
| GO:2001239 | regulation of extrinsic apoptotic signaling pathway in absence of ligand | 5/397 | 0.00251303 | 0.02435125 | 0.01896823 | RET/CSF2/SNAI2/GDNF/COL2A1 | 5 | BP |
| GO:0030182 | neuron differentiation | 43/397 | 0.00251459 | 0.02435125 | 0.01896823 | ADGRV1/CTNND2/DPYSL5/EMX2/LHX1/MAG/PAX7/TNC/BMP6/CCKAR/FZD10/GNAT1/KIF5C/LHX2/LMX1A/NEUROD4/NKX2-5/RET/S100B/SH3GL2/SOX1/SOX3/SYT4/CHRNA3/CHRNB2/FOXN4/INSM1/LIN28A/NEFL/PHOX2A/PTF1A/CDH11/GDF5/HAND2/GDNF/OTX2/PHOX2B/WNT7A/ASCL1/NEUROD1/NEUROG3/SNAP25/TH | 43 | BP |
| GO:0048863 | stem cell differentiation | 13/397 | 0.0025158 | 0.02435125 | 0.01896823 | NKX2-5/RET/TP63/HMGA2/LIN28A/SNAI2/GREM1/HAND2/GDNF/PHOX2B/WNT7A/ASCL1/KIT | 13 | BP |
| GO:0044255 | cellular lipid metabolic process | 36/397 | 0.00251607 | 0.02435125 | 0.01896823 | ALPI/B4GALNT2/CCKBR/CEL/FADS6/HAO1/MTMR7/PIK3C2G/PLPP4/ALDH1A3/ANGPTL3/PCK1/FGF21/G6PC/APOA4/CHAT/PPARGC1A/APOA5/AKR1B10/CES1/GSTA1/KIT/TTR/AKR1C4/CYP26A1/AKR1C2/APOC3/CBR1/TH/AKR1C1/UGT1A3/AKR1C3/UGT1A1/UGT1A7/UGT1A8/CYP1A1 | 36 | BP |
| GO:0070382 | exocytic vesicle | 10/403 | 0.00330379 | 0.02446871 | 0.01912722 | RAB3C/KCNK9/SH3GL2/SYT4/KIF1A/DDC/GRIN2A/SYP/SNAP25/TH | 10 | CC |
| GO:0070405 | ammonium ion binding | 5/372 | 0.0038962 | 0.02479249 | 0.01900329 | CHRNA3/CHRNB2/APOA4/APOA5/TH | 5 | MF |
| GO:1990837 | sequence-specific double-stranded DNA binding | 26/372 | 0.00391093 | 0.02479249 | 0.01900329 | ESX1/GLIS1/HAND1/MYCN/ZIC3/LHX2/LMX1A/NKX2-5/SOX1/SOX3/FOXN4/HMGA2/INSM1/PHOX2A/PTF1A/SNAI2/HAND2/HNF4A/RFX6/OTX2/PHOX2B/PAX4/CDX2/ASCL1/NEUROD1/NEUROG3 | 26 | MF |
| GO:1904315 | transmitter-gated ion channel activity involved in regulation of postsynaptic membrane potential | 4/372 | 0.00396998 | 0.02494412 | 0.01911951 | CHRNA3/CHRNA5/CHRNB2/CHRNA9 | 4 | MF |
| GO:0009635 | response to herbicide | 3/397 | 0.00262015 | 0.02505053 | 0.01951294 | NEFL/TH/CYP1A1 | 3 | BP |
| GO:0009642 | response to light intensity | 3/397 | 0.00262015 | 0.02505053 | 0.01951294 | KCNC2/GNAT1/KCNC1 | 3 | BP |
| GO:0016114 | terpenoid biosynthetic process | 3/397 | 0.00262015 | 0.02505053 | 0.01951294 | ALDH1A3/AKR1C3/CYP1A1 | 3 | BP |
| GO:0032536 | regulation of cell projection size | 3/397 | 0.00262015 | 0.02505053 | 0.01951294 | CDHR2/NEFL/WNT7A | 3 | BP |
| GO:0034370 | triglyceride-rich lipoprotein particle remodeling | 3/397 | 0.00262015 | 0.02505053 | 0.01951294 | APOA4/APOA5/APOC3 | 3 | BP |
| GO:0042574 | retinal metabolic process | 3/397 | 0.00262015 | 0.02505053 | 0.01951294 | ALDH1A3/AKR1C1/AKR1C3 | 3 | BP |
| GO:1990748 | cellular detoxification | 8/397 | 0.00262628 | 0.02505841 | 0.01951907 | IYD/SRXN1/TXNRD1/APOA4/AKR1B10/GPX2/NQO1/GSTA1 | 8 | BP |
| GO:0099055 | integral component of postsynaptic membrane | 8/403 | 0.00348884 | 0.02541369 | 0.01986591 | NETO1/KCNC1/ADORA1/CHRNB2/GABRA1/CDH9/CHRNA9/GRIN2A | 8 | CC |
| GO:0005604 | basement membrane | 7/403 | 0.00353862 | 0.02541369 | 0.01986591 | TNC/COL8A2/TGFBI/COL5A1/THBS2/COL2A1/VTN | 7 | CC |
| GO:0023056 | positive regulation of signaling | 56/397 | 0.0027076 | 0.02578221 | 0.02008287 | ADGRV1/GALR1/INHA/IRS4/NETO1/NRG4/SYBU/VSNL1/ANO1/BMP6/CASR/ELANE/EREG/FZD10/RET/S100B/SFRP4/TDGF1/TP63/ADORA1/CHRNB2/CSF2/F7/FGFBP1/LIN28A/THPO/FGF21/GDF5/GREM1/HAND2/KLB/RFX6/FGF16/FGF5/S100A7/WNT16/WNT7A/FGF4/KL/PTH/CHGA/GRIN2A/ASCL1/GAL/SNAP25/KIT/VTN/AKR1C2/FGB/TAC1/COL1A1/ITGB3/FGG/AKR1C3/F2/FGA | 56 | BP |
| GO:0043491 | protein kinase B signaling | 14/397 | 0.00273269 | 0.02596885 | 0.02022825 | NRG4/EREG/RET/F7/LIN28A/THPO/KLB/FGF16/FGF5/FGF4/KL/KIT/AKR1C2/AKR1C3 | 14 | BP |
| GO:0046164 | alcohol catabolic process | 5/397 | 0.00278451 | 0.02611797 | 0.02034441 | CEL/HAO1/MTMR7/AKR1B10/AKR1C3 | 5 | BP |
| GO:0042745 | circadian sleep/wake cycle | 4/397 | 0.00278668 | 0.02611797 | 0.02034441 | ADORA1/CHRNB2/CSF2/TH | 4 | BP |
| GO:0045907 | positive regulation of vasoconstriction | 4/397 | 0.00278668 | 0.02611797 | 0.02034441 | CASR/FGB/FGG/FGA | 4 | BP |
| GO:2001025 | positive regulation of response to drug | 4/397 | 0.00278668 | 0.02611797 | 0.02034441 | GALR1/CHRNB2/GDNF/GAL | 4 | BP |
| GO:0042475 | odontogenesis of dentin-containing tooth | 7/397 | 0.00279274 | 0.02611797 | 0.02034441 | HAND1/ODAPH/TNC/TP63/HAND2/DMP1/FGF4 | 7 | BP |
| GO:0019827 | stem cell population maintenance | 10/397 | 0.0027972 | 0.02611797 | 0.02034441 | ZIC3/PIWIL2/TDGF1/TP63/HMGA2/LIN28A/WNT7A/FGF4/CDX2/KIT | 10 | BP |
| GO:0001878 | response to yeast | 2/397 | 0.00285506 | 0.02611797 | 0.02034441 | PTX3/ELANE | 2 | BP |
| GO:0006591 | ornithine metabolic process | 2/397 | 0.00285506 | 0.02611797 | 0.02034441 | ODC1/HNF4A | 2 | BP |
| GO:0015670 | carbon dioxide transport | 2/397 | 0.00285506 | 0.02611797 | 0.02034441 | RHAG/AQP1 | 2 | BP |
| GO:0021759 | globus pallidus development | 2/397 | 0.00285506 | 0.02611797 | 0.02034441 | KCNC2/KCNC1 | 2 | BP |
| GO:0033366 | protein localization to secretory granule | 2/397 | 0.00285506 | 0.02611797 | 0.02034441 | SCG3/CHGA | 2 | BP |
| GO:0035814 | negative regulation of renal sodium excretion | 2/397 | 0.00285506 | 0.02611797 | 0.02034441 | SPX/ADORA1 | 2 | BP |
| GO:0042851 | L-alanine metabolic process | 2/397 | 0.00285506 | 0.02611797 | 0.02034441 | GPT2/GPT | 2 | BP |
| GO:0042853 | L-alanine catabolic process | 2/397 | 0.00285506 | 0.02611797 | 0.02034441 | GPT2/GPT | 2 | BP |
| GO:0046684 | response to pyrethroid | 2/397 | 0.00285506 | 0.02611797 | 0.02034441 | DDC/TH | 2 | BP |
| GO:0051464 | positive regulation of cortisol secretion | 2/397 | 0.00285506 | 0.02611797 | 0.02034441 | GALR1/GAL | 2 | BP |
| GO:0097068 | response to thyroxine | 2/397 | 0.00285506 | 0.02611797 | 0.02034441 | F7/GCLC | 2 | BP |
| GO:1900738 | positive regulation of phospholipase C-activating G protein-coupled receptor signaling pathway | 2/397 | 0.00285506 | 0.02611797 | 0.02034441 | CHGA/F2 | 2 | BP |
| GO:1904386 | response to L-phenylalanine derivative | 2/397 | 0.00285506 | 0.02611797 | 0.02034441 | F7/GCLC | 2 | BP |
| GO:0048468 | cell development | 62/397 | 0.00285899 | 0.02611797 | 0.02034441 | ADGRV1/CLDN18/CTNND2/DPYSL5/LHX1/MAG/MYPN/NKX3-2/PTX3/RHAG/SPINK1/TNC/BMP6/CCKAR/EREG/GNAT1/KIF5C/LHX2/LMX1A/NEUROD4/NKX2-5/OCA2/PIWIL2/RET/S100B/SH3GL2/SOX1/SOX3/SYT4/TP63/CHRNA3/CHRNB2/G6PD/INSM1/LIN28A/NEFL/SNAI2/THPO/CDH11/GDF5/HAND2/HNF4A/GDNF/OTX2/PHOX2B/WNT7A/COMP/DEFB1/ASCL1/GAL/NEUROD1/NEUROG3/PROC/SNAP25/COL11A1/KIT/FGB/TH/ITGB3/FGG/F2/FGA | 62 | BP |
| GO:0012501 | programmed cell death | 64/397 | 0.00287419 | 0.0261641 | 0.02038034 | MAG/NKX3-2/PAX7/PTGFR/ALDH1A3/AQP1/AZU1/CASR/DSC3/NKX2-5/PRAME/RET/S100B/SFRP4/TDGF1/TP63/ADORA1/CSF2/G6PD/HMGA2/NEFL/SNAI2/FGF21/GDF5/GREM1/HAND2/KRT5/KRT6B/SCG2/GCLC/GDNF/KRT14/PPARGC1A/WNT7A/COMP/FGF4/KRT20/PAX4/PTH/GRIN2A/ASCL1/DSG3/GAL/IVL/NEUROD1/NQO1/PROC/SPRR2A/SPRR2F/SPRR2G/SPRR2B/SPRR2E/COL2A1/KIT/SPRR2D/SPRR3/SPRR1A/FGB/SPRR1B/KNG1/FGG/SST/AKR1C3/FGA | 64 | BP |
| GO:0051234 | establishment of localization | 134/397 | 0.00287511 | 0.0261641 | 0.02038034 | ADGRV1/AQP12A/CA6/CCKBR/CD207/CEL/CLCA2/CPLX2/GALR1/GRIK3/GUCA2B/HAO1/INHA/KCNC2/KCNE4/KCNK2/KCNU1/KLRF2/LCN12/NETO1/PLPP4/PTX3/RHAG/SCN3A/SLC10A2/SLC17A2/SLC5A5/SLC6A19/SLC7A9/SPINK1/SPX/STXBP5L/SYBU/TRPM8/VGF/VSNL1/ZG16/ANO1/AOC1/AQP1/ATP4A/AZU1/BMP6/CASR/ELANE/GLP1R/KCNC1/KCNK3/KCNK9/KIF5C/NKX2-5/OCA2/SFRP4/SH3GL2/SYT4/TP63/UNC80/ABCC8/ADORA1/APOF/BPIFA1/CCL1/CDH17/CHRNA3/CHRNA5/CHRNB2/CSF2/F7/G6PD/GABRA1/H1-1/KCNJ18/KCNK10/KIF1A/NEFL/PRSS2/SCG3/SLC18A3/SLCO1A2/CACNA1S/DDC/FGF21/G6PC/GREM1/HNF4A/PLAU/PPBP/RFX6/SCG2/TF/APOA4/CHAT/CHRNA9/GDNF/KCNQ2/KCNQ5/PPARGC1A/S100A7/WNT7A/ABCC2/APOA5/COMP/KRT20/ORM2/PTH/CDX2/CHGA/GRIN2A/CALML5/GAL/HNF1A/NEUROD1/ORM1/PROC/SERPINA7/SYP/CES1/SNAP25/KIT/TTR/AKR1C4/AMBP/VTN/APOC3/FGB/TAC1/KNG1/TH/AKR1C1/COL1A1/ITGB3/FGG/F2/FGA | 134 | BP |
| GO:0097237 | cellular response to toxic substance | 13/397 | 0.00290666 | 0.02640027 | 0.0205643 | IYD/KCNC2/AQP1/SRXN1/TXNRD1/APOA4/PPARGC1A/AKR1B10/GPX2/NQO1/GSTA1/TH/UGT1A1 | 13 | BP |
| GO:0030673 | axolemma | 3/403 | 0.00374161 | 0.02647046 | 0.02069199 | KCNC2/KCNC1/ADORA1 | 3 | CC |
| GO:0019932 | second-messenger-mediated signaling | 17/397 | 0.00292711 | 0.026535 | 0.02066925 | GALR1/GPR26/GUCA2B/KCNC2/PTGFR/SPINK1/UCN2/AQP1/AZU1/GLP1R/CALCA/DEFB1/PTH/CHGA/GRIN2A/GAL/NEUROD1 | 17 | BP |
| GO:0070888 | E-box binding | 5/372 | 0.00426247 | 0.02654693 | 0.02034806 | PTF1A/SNAI2/HAND2/ASCL1/NEUROD1 | 5 | MF |
| GO:0006928 | movement of cell or subcellular component | 63/397 | 0.00293483 | 0.02655399 | 0.02068404 | DPYSL5/EMX2/KCNE4/LHX1/MAG/MYBPC1/NRG4/PSG2/SLC7A9/SRPX2/SYBU/ANGPTL3/AZU1/CCKAR/CGA/CXCL14/CXCL6/ELANE/EREG/KIF5C/LHX2/LMX1A/MYL1/NEB/NEUROD4/RET/S100A2/SOX1/TDGF1/UMOD/ADORA1/CALCA/CCL1/F7/FGFBP1/INSM1/KIF1A/NEFL/SNAI2/GREM1/HAND2/PLAU/SCG2/FGF16/GDNF/OTX2/PHOX2B/PPARGC1A/S100A7/WNT7A/DEFB1/FGF4/CHGA/ASCL1/COL5A1/ITGA11/KIT/COL1A2/VTN/TAC1/COL1A1/ITGB3/F2 | 63 | BP |
| GO:0008543 | fibroblast growth factor receptor signaling pathway | 8/397 | 0.00294674 | 0.02655998 | 0.02068871 | FGFBP1/FGF21/KLB/FGF16/FGF5/OTX2/FGF4/KL | 8 | BP |
| GO:0048593 | camera-type eye morphogenesis | 8/397 | 0.00294674 | 0.02655998 | 0.02068871 | LHX1/ALDH1A3/COL8A2/SOX1/FOXN4/PTF1A/WNT16/TH | 8 | BP |
| GO:0050920 | regulation of chemotaxis | 11/397 | 0.00297816 | 0.02679207 | 0.02086949 | AZU1/CASR/CXCL14/ELANE/CCL1/F7/GREM1/SCG2/FGF16/S100A7/FGF4 | 11 | BP |
| GO:0072503 | cellular divalent inorganic cation homeostasis | 18/397 | 0.00301075 | 0.02703378 | 0.02105777 | CALCB/CCKBR/CCL15/GALR1/TRPM8/CASR/ELANE/GLP1R/KCNK3/ADORA1/CALCA/CCL1/CHRNA9/PTH/GRIN2A/TAC1/KNG1/F2 | 18 | BP |
| GO:0098727 | maintenance of cell number | 10/397 | 0.00306293 | 0.02745011 | 0.02138207 | ZIC3/PIWIL2/TDGF1/TP63/HMGA2/LIN28A/WNT7A/FGF4/CDX2/KIT | 10 | BP |
| GO:0035987 | endodermal cell differentiation | 5/397 | 0.00307659 | 0.02752037 | 0.0214368 | HMGA2/COL12A1/COL5A1/COL11A1/VTN | 5 | BP |
| GO:0035690 | cellular response to drug | 17/397 | 0.00309703 | 0.02765083 | 0.02153842 | KCNC2/AOC1/AQP1/KCNC1/CHRNA3/CHRNB2/HMGA2/CACNA1S/DDC/FGF21/GCLC/MMP3/PPARGC1A/ABCC2/NQO1/TH/UGT1A1 | 17 | BP |
| GO:0017157 | regulation of exocytosis | 11/397 | 0.00310319 | 0.02765355 | 0.02154054 | CPLX2/STXBP5L/VSNL1/SYT4/CHRNA5/CHRNB2/WNT7A/SYP/FGB/FGG/FGA | 11 | BP |
| GO:0048864 | stem cell development | 6/397 | 0.00313474 | 0.02777743 | 0.02163703 | RET/SNAI2/HAND2/GDNF/PHOX2B/WNT7A | 6 | BP |
| GO:0071300 | cellular response to retinoic acid | 6/397 | 0.00313474 | 0.02777743 | 0.02163703 | TNC/AQP1/FZD10/RET/PCK1/COL1A1 | 6 | BP |
| GO:0072577 | endothelial cell apoptotic process | 6/397 | 0.00313474 | 0.02777743 | 0.02163703 | FGF21/SCG2/FGB/FGG/AKR1C3/FGA | 6 | BP |
| GO:0031526 | brush border membrane | 5/403 | 0.0039917 | 0.02782452 | 0.02175046 | CDHR2/SLC6A19/SLC7A9/AQP1/ABCC2 | 5 | CC |
| GO:0050662 | coenzyme binding | 13/372 | 0.00454689 | 0.02784561 | 0.02134349 | HAO1/IYD/ALDH1A3/OGDHL/G6PD/DDC/GPT2/TXNRD1/GCLC/GPT/UGDH/CBR3/TH | 13 | MF |
| GO:0016714 | oxidoreductase activity, acting on paired donors, with incorporation or reduction of molecular oxygen, reduced pteridine as one donor, and incorporation of one atom of oxygen | 2/372 | 0.00458864 | 0.02784561 | 0.02134349 | PAH/TH | 2 | MF |
| GO:0031995 | insulin-like growth factor II binding | 2/372 | 0.00458864 | 0.02784561 | 0.02134349 | IGFBP6/IGFBP1 | 2 | MF |
| GO:0048384 | retinoic acid receptor signaling pathway | 4/397 | 0.00319501 | 0.02820566 | 0.0219706 | PRAME/PTF1A/CYP26A1/AKR1C3 | 4 | BP |
| GO:0050974 | detection of mechanical stimulus involved in sensory perception | 4/397 | 0.00319501 | 0.02820566 | 0.0219706 | ADGRV1/CHRNA9/COL11A1/KIT | 4 | BP |
| GO:0005975 | carbohydrate metabolic process | 24/397 | 0.00321752 | 0.02835141 | 0.02208413 | MTMR7/ANGPTL3/ENO3/OGDHL/G6PD/PCK1/G6PC/KLB/TFF3/GCLC/PPARGC1A/TFF1/KL/PTH/UGDH/UGT2A3/UGT2B11/UGT2B4/UGT1A3/UGT1A1/UGT1A4/UGT1A6/UGT1A7/UGT1A8 | 24 | BP |
| GO:0030595 | leukocyte chemotaxis | 11/397 | 0.00323242 | 0.02842962 | 0.02214505 | AZU1/CXCL14/CXCL6/CALCA/CCL1/F7/GREM1/SCG2/S100A7/CHGA/KIT | 11 | BP |
| GO:1901652 | response to peptide | 21/397 | 0.00327766 | 0.02869054 | 0.02234829 | CRHR2/GH2/GLP2R/VGF/CASR/EREG/GLP1R/F7/NEFL/PCK1/FGF21/IGFBP1/MMP13/GCLC/MMP3/TFF1/ABCC2/KL/GAL/TH/COL1A1 | 21 | BP |
| GO:0021527 | spinal cord association neuron differentiation | 3/397 | 0.00328031 | 0.02869054 | 0.02234829 | LHX1/PAX7/ASCL1 | 3 | BP |
| GO:0046851 | negative regulation of bone remodeling | 3/397 | 0.00328031 | 0.02869054 | 0.02234829 | CLDN18/CALCA/GREM1 | 3 | BP |
| GO:0060041 | retina development in camera-type eye | 9/397 | 0.00333438 | 0.02910958 | 0.0226747 | LHX1/GNAT1/LHX2/NEUROD4/RET/FOXN4/PTF1A/PAX4/NEUROD1 | 9 | BP |
| GO:0099504 | synaptic vesicle cycle | 10/397 | 0.0033484 | 0.02917806 | 0.02272804 | STXBP5L/SH3GL2/SYT4/CHRNA5/CHRNB2/DDC/WNT7A/SYP/SNAP25/TH | 10 | BP |
| GO:0061035 | regulation of cartilage development | 6/397 | 0.00338517 | 0.02932531 | 0.02284274 | NKX3-2/BMP6/SNAI2/GDF5/GREM1/PTH | 6 | BP |
| GO:0021517 | ventral spinal cord development | 5/397 | 0.00339014 | 0.02932531 | 0.02284274 | LHX1/SOX1/FOXN4/PHOX2A/ASCL1 | 5 | BP |
| GO:0060986 | endocrine hormone secretion | 5/397 | 0.00339014 | 0.02932531 | 0.02284274 | GALR1/INHA/BMP6/GAL/TAC1 | 5 | BP |
| GO:0070169 | positive regulation of biomineral tissue development | 5/397 | 0.00339014 | 0.02932531 | 0.02284274 | ADGRV1/ODAPH/BMP6/KL/PTH | 5 | BP |
| GO:0098936 | intrinsic component of postsynaptic membrane | 8/403 | 0.00451864 | 0.03028124 | 0.02367088 | NETO1/KCNC1/ADORA1/CHRNB2/GABRA1/CDH9/CHRNA9/GRIN2A | 8 | CC |
| GO:0098590 | plasma membrane region | 38/403 | 0.00452089 | 0.03028124 | 0.02367088 | ADGRV1/CDHR2/CLCA2/KCNC2/KCNE4/KCNK2/LRFN2/NETO1/SLC6A19/SLC7A9/SRPX2/ANO1/AQP1/CASR/CLDN8/GABRG3/GNAT1/GRK1/KCNC1/TDGF1/UMOD/ADORA1/CDH17/CHRNA3/CHRNA5/CHRNB2/GABRA1/TF/CDH9/CHRNA9/GABBR2/MUC13/ABCC2/KL/GRIN2A/SYP/SNAP25/ITGB3 | 38 | CC |
| GO:0033267 | axon part | 16/403 | 0.0045358 | 0.03028124 | 0.02367088 | GRIK3/KCNC2/KCNK2/MAG/SYBU/CASR/KCNC1/KIF5C/ADORA1/KIF1A/NEFL/KCNQ2/OTX2/SYP/SNAP25/TH | 16 | CC |
| GO:0022008 | neurogenesis | 49/397 | 0.00355478 | 0.03066141 | 0.02388348 | ADGRV1/CTNND2/DPYSL5/EMX2/LHX1/MAG/PAX7/TNC/AZU1/BMP6/CCKAR/FZD10/GNAT1/KIF5C/LHX2/LMX1A/NEUROD4/NKX2-5/RET/S100B/SH3GL2/SOX1/SOX3/SYT4/CHRNA3/CHRNB2/FOXN4/INSM1/LIN28A/NEFL/PHOX2A/PTF1A/CDH11/GDF5/HAND2/FGF5/GDNF/OTX2/PHOX2B/WNT7A/GRIN2A/ASCL1/NEUROD1/NEUROG3/SNAP25/KIT/VTN/TH/F2 | 49 | BP |
| GO:0048562 | embryonic organ morphogenesis | 14/397 | 0.00355758 | 0.03066141 | 0.02388348 | HAND1/LHX1/NKX3-2/ZIC3/ALDH1A3/NKX2-5/FOXN4/HAND2/CHRNA9/WNT16/NEUROD1/COL11A1/COL2A1/TH | 14 | BP |
| GO:0004175 | endopeptidase activity | 15/372 | 0.00513973 | 0.03092548 | 0.02370419 | ADAM20/ADAM2/AZU1/ELANE/KLK3/MMP10/PCSK2/F7/PRSS2/MMP13/PLAT/PLAU/MMP3/PROC/F2 | 15 | MF |
| GO:0045940 | positive regulation of steroid metabolic process | 4/397 | 0.00364228 | 0.03133423 | 0.02440758 | BMP6/CGA/APOA4/PPARGC1A | 4 | BP |
| GO:0048168 | regulation of neuronal synaptic plasticity | 5/397 | 0.00372601 | 0.03199623 | 0.02492324 | NETO1/S100B/SYT4/SYP/KIT | 5 | BP |
| GO:0060349 | bone morphogenesis | 7/397 | 0.0038487 | 0.03295498 | 0.02567005 | BMP6/SFRP4/MMP13/COMP/FGF4/COL2A1/COL1A1 | 7 | BP |
| GO:0007605 | sensory perception of sound | 9/397 | 0.00385369 | 0.03295498 | 0.02567005 | ADGRV1/CHRNB2/SNAI2/CHRNA9/COL11A1/COL2A1/KIT/TH/COL1A1 | 9 | BP |
| GO:0006631 | fatty acid metabolic process | 16/397 | 0.00385859 | 0.03295498 | 0.02567005 | CEL/FADS6/HAO1/ANGPTL3/APOA4/PPARGC1A/APOA5/CES1/GSTA1/AKR1C2/APOC3/CBR1/TH/AKR1C3/UGT1A8/CYP1A1 | 16 | BP |
| GO:0006873 | cellular ion homeostasis | 22/397 | 0.00386593 | 0.03295812 | 0.02567249 | CALCB/CCKBR/CCL15/GALR1/RHAG/TRPM8/BMP6/CASR/ELANE/GLP1R/KCNK3/ADORA1/CALCA/CCL1/TF/CHRNA9/ABCC2/PTH/GRIN2A/TAC1/KNG1/F2 | 22 | BP |
| GO:1903524 | positive regulation of blood circulation | 6/397 | 0.00393031 | 0.0334466 | 0.02605299 | CASR/NKX2-5/CHGA/FGB/FGG/FGA | 6 | BP |
| GO:0099529 | neurotransmitter receptor activity involved in regulation of postsynaptic membrane potential | 4/372 | 0.00563034 | 0.03359278 | 0.02574865 | CHRNA3/CHRNA5/CHRNB2/CHRNA9 | 4 | MF |
| GO:0006875 | cellular metal ion homeostasis | 20/397 | 0.00400322 | 0.03383728 | 0.02635731 | CALCB/CCKBR/CCL15/GALR1/TRPM8/BMP6/CASR/ELANE/GLP1R/KCNK3/ADORA1/CALCA/CCL1/TF/CHRNA9/PTH/GRIN2A/TAC1/KNG1/F2 | 20 | BP |
| GO:0009314 | response to radiation | 19/397 | 0.00403025 | 0.03383728 | 0.02635731 | KCNC2/NETO1/PPP1R1B/RGR/ADAM2/AQP1/ELANE/GNAT1/GRK1/H2AW/KCNC1/CHRNB2/HMGA2/SNAI2/KRT14/GRIN2A/IVL/KIT/TH | 19 | BP |
| GO:0010566 | regulation of ketone biosynthetic process | 3/397 | 0.00403354 | 0.03383728 | 0.02635731 | BMP6/PPARGC1A/AKR1C3 | 3 | BP |
| GO:0021542 | dentate gyrus development | 3/397 | 0.00403354 | 0.03383728 | 0.02635731 | EMX2/LMX1A/NEUROD1 | 3 | BP |
| GO:0034310 | primary alcohol catabolic process | 3/397 | 0.00403354 | 0.03383728 | 0.02635731 | HAO1/AKR1B10/AKR1C3 | 3 | BP |
| GO:0042754 | negative regulation of circadian rhythm | 3/397 | 0.00403354 | 0.03383728 | 0.02635731 | PASD1/PIWIL2/ADORA1 | 3 | BP |
| GO:0060579 | ventral spinal cord interneuron fate commitment | 3/397 | 0.00403354 | 0.03383728 | 0.02635731 | SOX1/FOXN4/ASCL1 | 3 | BP |
| GO:0060581 | cell fate commitment involved in pattern specification | 3/397 | 0.00403354 | 0.03383728 | 0.02635731 | SOX1/FOXN4/ASCL1 | 3 | BP |
| GO:0046943 | carboxylic acid transmembrane transporter activity | 7/372 | 0.00577945 | 0.03419507 | 0.02621031 | SLC10A2/SLC6A19/SLC7A9/OCA2/SLCO1A2/ABCC2/AKR1C4 | 7 | MF |
| GO:0042632 | cholesterol homeostasis | 7/397 | 0.00409199 | 0.03426682 | 0.02669189 | ANGPTL3/G6PC/HNF4A/APOA4/APOA5/APOC3/AKR1C1 | 7 | BP |
| GO:0031099 | regeneration | 11/397 | 0.00410136 | 0.03428446 | 0.02670564 | MAG/PAX7/TNC/ENO3/F7/NEFL/IGFBP1/WNT7A/APOA5/VTN/UGT1A1 | 11 | BP |
| GO:0043434 | response to peptide hormone | 18/397 | 0.00412485 | 0.03434317 | 0.02675137 | CRHR2/GH2/GLP2R/VGF/EREG/GLP1R/F7/NEFL/PCK1/FGF21/IGFBP1/GCLC/TFF1/ABCC2/KL/GAL/TH/COL1A1 | 18 | BP |
| GO:0019433 | triglyceride catabolic process | 4/397 | 0.0041302 | 0.03434317 | 0.02675137 | FGF21/APOA4/APOA5/APOC3 | 4 | BP |
| GO:0034694 | response to prostaglandin | 4/397 | 0.0041302 | 0.03434317 | 0.02675137 | PTGFR/TNC/AKR1C2/AKR1C3 | 4 | BP |
| GO:0048545 | response to steroid hormone | 17/397 | 0.0042973 | 0.03566989 | 0.02778481 | CPN1/NR0B1/AQP1/BMP6/S100B/TP63/NEFL/TPH2/HNF4A/ALDH3A1/PPARGC1A/ABCC2/TH/COL1A1/SST/AKR1C3/UGT1A1 | 17 | BP |
| GO:0005342 | organic acid transmembrane transporter activity | 7/372 | 0.00610534 | 0.03582474 | 0.02745944 | SLC10A2/SLC6A19/SLC7A9/OCA2/SLCO1A2/ABCC2/AKR1C4 | 7 | MF |
| GO:0055092 | sterol homeostasis | 7/397 | 0.00434676 | 0.03601706 | 0.02805523 | ANGPTL3/G6PC/HNF4A/APOA4/APOA5/APOC3/AKR1C1 | 7 | BP |
| GO:0060326 | cell chemotaxis | 13/397 | 0.00438594 | 0.03627808 | 0.02825855 | AZU1/CXCL14/CXCL6/CALCA/CCL1/F7/GREM1/SCG2/FGF16/S100A7/FGF4/CHGA/KIT | 13 | BP |
| GO:0098960 | postsynaptic neurotransmitter receptor activity | 4/372 | 0.00627265 | 0.03650475 | 0.02798066 | CHRNA3/CHRNA5/CHRNB2/CHRNA9 | 4 | MF |
| GO:0007612 | learning | 9/397 | 0.0044343 | 0.03661395 | 0.02852018 | NETO1/PPP1R1B/ADAM2/CHRNB2/GRIN2A/SNAP25/KIT/TAC1/TH | 9 | BP |
| GO:0060395 | SMAD protein signal transduction | 5/397 | 0.00446816 | 0.03682921 | 0.02868785 | BMP6/CILP/GDF5/HNF4A/AFP | 5 | BP |
| GO:0097060 | synaptic membrane | 17/403 | 0.00566818 | 0.0369068 | 0.02885009 | KCNC2/LRFN2/NETO1/SRPX2/GABRG3/KCNC1/ADORA1/CHRNA3/CHRNA5/CHRNB2/GABRA1/CDH9/CHRNA9/GABBR2/GRIN2A/SYP/SNAP25 | 17 | CC |
| GO:0045211 | postsynaptic membrane | 14/403 | 0.00568396 | 0.0369068 | 0.02885009 | KCNC2/LRFN2/NETO1/GABRG3/KCNC1/ADORA1/CHRNA3/CHRNA5/CHRNB2/GABRA1/CDH9/CHRNA9/GABBR2/GRIN2A | 14 | CC |
| GO:0016477 | cell migration | 48/397 | 0.00458608 | 0.03696958 | 0.02879719 | EMX2/LHX1/MAG/PSG2/SLC7A9/SRPX2/ANGPTL3/AZU1/CCKAR/CGA/CXCL14/CXCL6/ELANE/NEUROD4/RET/S100A2/SOX1/TDGF1/UMOD/ADORA1/CALCA/CCL1/F7/FGFBP1/INSM1/SNAI2/GREM1/HAND2/PLAU/SCG2/FGF16/GDNF/PHOX2B/PPARGC1A/S100A7/WNT7A/FGF4/CHGA/ASCL1/COL5A1/ITGA11/KIT/COL1A2/VTN/TAC1/COL1A1/ITGB3/F2 | 48 | BP |
| GO:0051591 | response to cAMP | 7/397 | 0.00461333 | 0.03696958 | 0.02879719 | SLC5A5/VGF/AQP1/PCK1/ALDH3A1/PAX4/COL1A1 | 7 | BP |
| GO:0019933 | cAMP-mediated signaling | 9/397 | 0.00464227 | 0.03696958 | 0.02879719 | GALR1/GPR26/UCN2/GLP1R/CALCA/DEFB1/PTH/CHGA/GAL | 9 | BP |
| GO:0030431 | sleep | 4/397 | 0.00466041 | 0.03696958 | 0.02879719 | ADORA1/CHRNB2/CSF2/GRIN2A | 4 | BP |
| GO:0035774 | positive regulation of insulin secretion involved in cellular response to glucose stimulus | 4/397 | 0.00466041 | 0.03696958 | 0.02879719 | SYBU/VSNL1/ANO1/RFX6 | 4 | BP |
| GO:0042572 | retinol metabolic process | 4/397 | 0.00466041 | 0.03696958 | 0.02879719 | ALDH1A3/AKR1B10/TTR/AKR1C3 | 4 | BP |
| GO:0006522 | alanine metabolic process | 2/397 | 0.00468871 | 0.03696958 | 0.02879719 | GPT2/GPT | 2 | BP |
| GO:0006524 | alanine catabolic process | 2/397 | 0.00468871 | 0.03696958 | 0.02879719 | GPT2/GPT | 2 | BP |
| GO:0008628 | hormone-mediated apoptotic signaling pathway | 2/397 | 0.00468871 | 0.03696958 | 0.02879719 | PTH/SST | 2 | BP |
| GO:0009078 | pyruvate family amino acid metabolic process | 2/397 | 0.00468871 | 0.03696958 | 0.02879719 | GPT2/GPT | 2 | BP |
| GO:0009080 | pyruvate family amino acid catabolic process | 2/397 | 0.00468871 | 0.03696958 | 0.02879719 | GPT2/GPT | 2 | BP |
| GO:0010041 | response to iron(III) ion | 2/397 | 0.00468871 | 0.03696958 | 0.02879719 | G6PD/CYP1A1 | 2 | BP |
| GO:0010901 | regulation of very-low-density lipoprotein particle remodeling | 2/397 | 0.00468871 | 0.03696958 | 0.02879719 | APOA5/APOC3 | 2 | BP |
| GO:0015705 | iodide transport | 2/397 | 0.00468871 | 0.03696958 | 0.02879719 | SLC5A5/ANO1 | 2 | BP |
| GO:0031133 | regulation of axon diameter | 2/397 | 0.00468871 | 0.03696958 | 0.02879719 | NEFL/WNT7A | 2 | BP |
| GO:0042435 | indole-containing compound biosynthetic process | 2/397 | 0.00468871 | 0.03696958 | 0.02879719 | TPH2/DDC | 2 | BP |
| GO:0043400 | cortisol secretion | 2/397 | 0.00468871 | 0.03696958 | 0.02879719 | GALR1/GAL | 2 | BP |
| GO:0046219 | indolalkylamine biosynthetic process | 2/397 | 0.00468871 | 0.03696958 | 0.02879719 | TPH2/DDC | 2 | BP |
| GO:0051462 | regulation of cortisol secretion | 2/397 | 0.00468871 | 0.03696958 | 0.02879719 | GALR1/GAL | 2 | BP |
| GO:0060591 | chondroblast differentiation | 2/397 | 0.00468871 | 0.03696958 | 0.02879719 | GDF5/FGF4 | 2 | BP |
| GO:0061302 | smooth muscle cell-matrix adhesion | 2/397 | 0.00468871 | 0.03696958 | 0.02879719 | PLAU/VTN | 2 | BP |
| GO:0071332 | cellular response to fructose stimulus | 2/397 | 0.00468871 | 0.03696958 | 0.02879719 | PCK1/PPARGC1A | 2 | BP |
| GO:0090289 | regulation of osteoclast proliferation | 2/397 | 0.00468871 | 0.03696958 | 0.02879719 | GREM1/PTH | 2 | BP |
| GO:1900736 | regulation of phospholipase C-activating G protein-coupled receptor signaling pathway | 2/397 | 0.00468871 | 0.03696958 | 0.02879719 | CHGA/F2 | 2 | BP |
| GO:1901162 | primary amino compound biosynthetic process | 2/397 | 0.00468871 | 0.03696958 | 0.02879719 | TPH2/DDC | 2 | BP |
| GO:1904970 | brush border assembly | 2/397 | 0.00468871 | 0.03696958 | 0.02879719 | ANKS4B/CDHR2 | 2 | BP |
| GO:0097529 | myeloid leukocyte migration | 10/397 | 0.00470846 | 0.03706344 | 0.02887031 | AZU1/CXCL6/UMOD/CALCA/CCL1/GREM1/SCG2/S100A7/CHGA/KIT | 10 | BP |
| GO:0019955 | cytokine binding | 7/372 | 0.00644476 | 0.03720143 | 0.02851466 | ELANE/GREM2/GDF5/GREM1/COMP/KIT/ITGB3 | 7 | MF |
| GO:0099699 | integral component of synaptic membrane | 9/403 | 0.00581212 | 0.03722897 | 0.02910193 | NETO1/GABRG3/KCNC1/ADORA1/CHRNB2/GABRA1/CDH9/CHRNA9/GRIN2A | 9 | CC |
| GO:0051952 | regulation of amine transport | 6/397 | 0.00486635 | 0.03768761 | 0.0293565 | SYT4/ADORA1/CHRNA3/CHRNB2/GDNF/CHGA | 6 | BP |
| GO:2000300 | regulation of synaptic vesicle exocytosis | 6/397 | 0.00486635 | 0.03768761 | 0.0293565 | STXBP5L/SYT4/CHRNA5/CHRNB2/WNT7A/SYP | 6 | BP |
| GO:0051187 | cofactor catabolic process | 5/397 | 0.00487616 | 0.03768761 | 0.0293565 | APOA4/AMBP/CBR3/UGT1A1/UGT1A4 | 5 | BP |
| GO:0070741 | response to interleukin-6 | 5/397 | 0.00487616 | 0.03768761 | 0.0293565 | TDGF1/PCK1/PPARGC1A/ABCC2/FGG | 5 | BP |
| GO:0006957 | complement activation, alternative pathway | 3/397 | 0.00488351 | 0.03768761 | 0.0293565 | C8G/C8A/CFHR5 | 3 | BP |
| GO:0034116 | positive regulation of heterotypic cell-cell adhesion | 3/397 | 0.00488351 | 0.03768761 | 0.0293565 | FGB/FGG/FGA | 3 | BP |
| GO:0048385 | regulation of retinoic acid receptor signaling pathway | 3/397 | 0.00488351 | 0.03768761 | 0.0293565 | PRAME/CYP26A1/AKR1C3 | 3 | BP |
| GO:0061548 | ganglion development | 3/397 | 0.00488351 | 0.03768761 | 0.0293565 | INSM1/PHOX2B/ASCL1 | 3 | BP |
| GO:0071732 | cellular response to nitric oxide | 3/397 | 0.00488351 | 0.03768761 | 0.0293565 | KCNC2/AQP1/MMP3 | 3 | BP |
| GO:0072189 | ureter development | 3/397 | 0.00488351 | 0.03768761 | 0.0293565 | EMX2/LHX1/RET | 3 | BP |
| GO:0072283 | metanephric renal vesicle morphogenesis | 3/397 | 0.00488351 | 0.03768761 | 0.0293565 | LHX1/GREM1/GDNF | 3 | BP |
| GO:0097067 | cellular response to thyroid hormone stimulus | 3/397 | 0.00488351 | 0.03768761 | 0.0293565 | GCLC/PPARGC1A/KIT | 3 | BP |
| GO:0007189 | adenylate cyclase-activating G protein-coupled receptor signaling pathway | 7/397 | 0.00489204 | 0.03769187 | 0.02935982 | GALR1/GPR26/UCN2/GLP1R/CALCA/PTH/CHGA | 7 | BP |
| GO:0008076 | voltage-gated potassium channel complex | 6/403 | 0.00597192 | 0.03774257 | 0.0295034 | KCNC2/KCNK2/KCNC1/KCNQ2/KCNQ5/SNAP25 | 6 | CC |
| GO:0098802 | plasma membrane receptor complex | 9/403 | 0.00606139 | 0.03780391 | 0.02955135 | CHRNA3/CHRNA5/CHRNB2/TF/CHRNA9/GABBR2/GRIN2A/ITGA11/ITGB3 | 9 | CC |
| GO:0030278 | regulation of ossification | 11/397 | 0.00495959 | 0.03815004 | 0.02971671 | ADGRV1/OMD/BMP6/TP63/SNAI2/GREM1/HAND2/COMP/KL/PTH/TAC1 | 11 | BP |
| GO:0004030 | aldehyde dehydrogenase [NAD(P)+] activity | 2/372 | 0.00678343 | 0.0382241 | 0.02929853 | ALDH1A3/ALDH3A1 | 2 | MF |
| GO:0008199 | ferric iron binding | 2/372 | 0.00678343 | 0.0382241 | 0.02929853 | TF/TH | 2 | MF |
| GO:1905538 | polysome binding | 2/372 | 0.00678343 | 0.0382241 | 0.02929853 | PIWIL2/LIN28A | 2 | MF |
| GO:0014074 | response to purine-containing compound | 9/397 | 0.00508093 | 0.03901991 | 0.03039429 | SLC5A5/VGF/AQP1/PCK1/CACNA1S/ALDH3A1/PPARGC1A/PAX4/COL1A1 | 9 | BP |
| GO:1901605 | alpha-amino acid metabolic process | 11/397 | 0.00514716 | 0.03946434 | 0.03074047 | IYD/GLDC/HAL/ODC1/GPT2/HNF4A/GCLC/GPT/HGD/PAH/TH | 11 | BP |
| GO:0048568 | embryonic organ development | 18/397 | 0.00516747 | 0.03954842 | 0.03080596 | HAND1/LHX1/NKX3-2/ZIC3/ALDH1A3/NKX2-5/CSF2/FOXN4/HAND2/CHRNA9/GDNF/WNT16/CDX2/NEUROD1/COL11A1/COL2A1/KIT/TH | 18 | BP |
| GO:0008585 | female gonad development | 7/397 | 0.00518325 | 0.03954842 | 0.03080596 | INHA/PTX3/VGF/ZFP42/EREG/KIT/AFP | 7 | BP |
| GO:0060079 | excitatory postsynaptic potential | 7/397 | 0.00518325 | 0.03954842 | 0.03080596 | NETO1/ADORA1/CHRNA3/CHRNA5/CHRNB2/CHRNA9/WNT7A | 7 | BP |
| GO:0006081 | cellular aldehyde metabolic process | 6/397 | 0.00521195 | 0.03961967 | 0.03086146 | ALDH1A3/BMP6/G6PD/ALDH3A1/AKR1C1/AKR1C3 | 6 | BP |
| GO:0046685 | response to arsenic-containing substance | 4/397 | 0.00523453 | 0.03961967 | 0.03086146 | NEFL/GCLC/ABCC2/CYP1A1 | 4 | BP |
| GO:0071295 | cellular response to vitamin | 4/397 | 0.00523453 | 0.03961967 | 0.03086146 | TNC/CASR/SNAI2/COL1A1 | 4 | BP |
| GO:1901099 | negative regulation of signal transduction in absence of ligand | 4/397 | 0.00523453 | 0.03961967 | 0.03086146 | CSF2/SNAI2/GDNF/COL2A1 | 4 | BP |
| GO:2001240 | negative regulation of extrinsic apoptotic signaling pathway in absence of ligand | 4/397 | 0.00523453 | 0.03961967 | 0.03086146 | CSF2/SNAI2/GDNF/COL2A1 | 4 | BP |
| GO:0003014 | renal system process | 8/397 | 0.00528874 | 0.03996596 | 0.0311312 | SPX/AQP1/ADORA1/CHRNA3/CHRNB2/HNF1A/TAC1/AKR1C3 | 8 | BP |
| GO:0097433 | dense body | 2/403 | 0.00657872 | 0.03997839 | 0.03125115 | SYBU/PIWIL2 | 2 | CC |
| GO:1990742 | microvesicle | 2/403 | 0.00657872 | 0.03997839 | 0.03125115 | SYT4/DEFB1 | 2 | CC |
| GO:2000677 | regulation of transcription regulatory region DNA binding | 5/397 | 0.00530991 | 0.04005927 | 0.03120388 | HAND1/LHX2/HMGA2/HAND2/NEUROD1 | 5 | BP |
| GO:1903034 | regulation of response to wounding | 10/397 | 0.00531805 | 0.04005927 | 0.03120388 | F7/PLAT/PLAU/PROC/VTN/FGB/KNG1/FGG/F2/FGA | 10 | BP |
| GO:0003006 | developmental process involved in reproduction | 25/397 | 0.00545132 | 0.04099773 | 0.03193489 | HAND1/INHA/LHX1/NR0B1/PTX3/SPINK1/SRY/TNC/VGF/ZFP42/BMP6/EREG/OCA2/PIWIL2/SOX3/TP63/CSF2/LIN28A/HNF4A/WNT7A/DEFB1/CDX2/KIT/AFP/AKR1C3 | 25 | BP |
| GO:0003690 | double-stranded DNA binding | 27/372 | 0.00735825 | 0.04113668 | 0.03153101 | ESX1/GLIS1/HAND1/MYCN/ZIC3/LHX2/LMX1A/NKX2-5/SOX1/SOX3/TP63/FOXN4/HMGA2/INSM1/PHOX2A/PTF1A/SNAI2/HAND2/HNF4A/RFX6/OTX2/PHOX2B/PAX4/CDX2/ASCL1/NEUROD1/NEUROG3 | 27 | MF |
| GO:0046928 | regulation of neurotransmitter secretion | 7/397 | 0.00548728 | 0.04120256 | 0.03209444 | STXBP5L/SYT4/CHRNA3/CHRNA5/CHRNB2/WNT7A/SYP | 7 | BP |
| GO:0007492 | endoderm development | 6/397 | 0.00557521 | 0.04179638 | 0.03255699 | LHX1/HMGA2/COL12A1/COL5A1/COL11A1/VTN | 6 | BP |
| GO:0006887 | exocytosis | 31/397 | 0.00568251 | 0.04253326 | 0.03313098 | CPLX2/KLRF2/PTX3/STXBP5L/VSNL1/AOC1/AZU1/ELANE/SYT4/CHRNA5/CHRNB2/PRSS2/SCG3/PLAU/PPBP/TF/S100A7/WNT7A/ORM2/CHGA/CALML5/ORM1/SYP/SNAP25/KIT/TTR/FGB/KNG1/ITGB3/FGG/FGA | 31 | BP |
| GO:1901618 | organic hydroxy compound transmembrane transporter activity | 4/372 | 0.00769919 | 0.04270642 | 0.0327342 | SLC10A2/AQP1/SLCO1A2/AKR1C4 | 4 | MF |
| GO:0017075 | syntaxin-1 binding | 3/372 | 0.00780442 | 0.04295454 | 0.03292438 | SYBU/SYT4/SNAP25 | 3 | MF |
| GO:0060350 | endochondral bone morphogenesis | 5/397 | 0.00577023 | 0.04304956 | 0.03353315 | BMP6/MMP13/COMP/COL2A1/COL1A1 | 5 | BP |
| GO:0017144 | drug metabolic process | 29/397 | 0.00579056 | 0.04304956 | 0.03353315 | CPN1/HAO1/PTX3/ENO3/GLDC/HAL/OGDHL/CHRNB2/DDC/NT5E/APOA4/HGD/MMP3/PAH/PPARGC1A/ABCC2/AKR1B10/GRIN2A/NQO1/AFP/AKR1C4/AKR1C2/CBR1/TH/AKR1C1/AKR1C3/UGT1A1/UGT1A7/CYP1A1 | 29 | BP |
| GO:1903522 | regulation of blood circulation | 14/397 | 0.00581725 | 0.04304956 | 0.03353315 | SPX/CASR/GLP1R/KCNK3/NKX2-5/ADORA1/FOXN4/CACNA1S/CHGA/FGB/TAC1/TH/FGG/FGA | 14 | BP |
| GO:0007194 | negative regulation of adenylate cyclase activity | 3/397 | 0.00583352 | 0.04304956 | 0.03353315 | ADGRV1/GALR1/GABBR2 | 3 | BP |
| GO:0021514 | ventral spinal cord interneuron differentiation | 3/397 | 0.00583352 | 0.04304956 | 0.03353315 | SOX1/FOXN4/ASCL1 | 3 | BP |
| GO:0035729 | cellular response to hepatocyte growth factor stimulus | 3/397 | 0.00583352 | 0.04304956 | 0.03353315 | CASR/TDGF1/GCLC | 3 | BP |
| GO:0042401 | cellular biogenic amine biosynthetic process | 3/397 | 0.00583352 | 0.04304956 | 0.03353315 | ODC1/TPH2/DDC | 3 | BP |
| GO:0045187 | regulation of circadian sleep/wake cycle, sleep | 3/397 | 0.00583352 | 0.04304956 | 0.03353315 | ADORA1/CHRNB2/CSF2 | 3 | BP |
| GO:0071280 | cellular response to copper ion | 3/397 | 0.00583352 | 0.04304956 | 0.03353315 | AOC1/AQP1/CYP1A1 | 3 | BP |
| GO:0032094 | response to food | 4/397 | 0.00585411 | 0.04313411 | 0.03359901 | SPX/G6PD/G6PC/CYP1A1 | 4 | BP |
| GO:0015849 | organic acid transport | 14/397 | 0.00598988 | 0.0439216 | 0.03421242 | LCN12/SLC10A2/SLC6A19/SLC7A9/SPX/CASR/OCA2/SYT4/ADORA1/SLCO1A2/ABCC2/SNAP25/AKR1C4/AKR1C1 | 14 | BP |
| GO:0046942 | carboxylic acid transport | 14/397 | 0.00598988 | 0.0439216 | 0.03421242 | LCN12/SLC10A2/SLC6A19/SLC7A9/SPX/CASR/OCA2/SYT4/ADORA1/SLCO1A2/ABCC2/SNAP25/AKR1C4/AKR1C1 | 14 | BP |
| GO:2000026 | regulation of multicellular organismal development | 63/397 | 0.00599182 | 0.0439216 | 0.03421242 | ADGRV1/CLDN18/INHA/KCNK2/LHX1/MAG/NKX3-2/ODAPH/OMD/SRPX2/SRY/ANGPTL3/AQP1/BMP6/KLK3/LHX2/LMX1A/NKX2-5/RET/S100B/SFRP4/SOX3/SYT4/TP63/CHRNA3/CHRNB2/CSF2/FGFBP1/G6PD/HMGA2/LIN28A/NEFL/PTF1A/SNAI2/THPO/GDF5/GREM1/H4C1/H4C13/HAND2/HNF4A/DMP1/GDNF/OTX2/PHOX2B/WNT7A/COMP/H4C6/KL/PTH/CDX2/ASCL1/ASPN/COL5A1/GAL/NEUROD1/NEUROG3/PROC/THBS2/SNAP25/KIT/COL1A1/F2 | 63 | BP |
| GO:0048523 | negative regulation of cellular process | 124/397 | 0.00599819 | 0.0439216 | 0.03421242 | ADGRV1/B4GALNT2/CDHR2/CLDN18/ESX1/GLIS1/GRIK3/HAND1/IGFBP6/INHA/KCNK2/LHX1/MAG/NKX3-2/NR0B1/PASD1/PAX7/PPP1R1B/PTGFR/PTX3/RIMBP2/SPINK1/SPINK4/SSTR1/SSX1/STXBP5L/TNC/VSNL1/WFDC12/WFDC5/AQP1/AZU1/BMP6/CILP/CXCL14/DLK1/ELANE/EREG/GNG4/LHX2/LMX1A/NKX2-5/NOTUM/PIWIL2/PRAME/S100B/SFRP4/SH3GL2/SOX3/SYT4/TDGF1/TP63/UMOD/WIF1/ADORA1/BPIFA1/CALCA/CCL17/CSF2/FGFBP1/FGL1/G6PD/GREM2/HMGA2/INSM1/LIN28A/MMP11/NEFL/SNAI2/TGFBI/FGF21/FOXG1/GDF5/GREM1/H4C1/H4C13/HAND2/HNF4A/IGFBP1/MAGEA1/PLAT/SCG2/APOA4/CHRNA9/GCLC/GDNF/MMP3/PHOX2B/PPARGC1A/TFF1/WNT16/WNT7A/COMP/FGF4/H4C6/PAX4/PTH/CDX2/CHGA/F13B/ASCL1/ASPN/COL5A1/GAL/NEUROD1/NEUROG3/NQO1/PROC/COL2A1/KIT/AMBP/CYP26A1/VTN/APOC3/FGB/KNG1/COL1A1/ITGB3/FGG/SST/AKR1C3/F2/UGT1A8/FGA | 124 | BP |
| GO:1901699 | cellular response to nitrogen compound | 24/397 | 0.00604146 | 0.04412578 | 0.03437146 | CRHR2/GH2/GLP2R/KCNC2/SLC5A5/AOC1/AQP1/CASR/GLP1R/CHRNA3/CHRNB2/GABRA1/PCK1/CACNA1S/DDC/FGF21/IGFBP1/GCLC/MMP3/PPARGC1A/KL/COL1A2/TH/COL1A1 | 24 | BP |
| GO:0048870 | cell motility | 51/397 | 0.0060541 | 0.04412578 | 0.03437146 | EMX2/LHX1/MAG/NRG4/PSG2/SLC7A9/SRPX2/ANGPTL3/AZU1/CCKAR/CGA/CXCL14/CXCL6/ELANE/EREG/NEUROD4/RET/S100A2/SOX1/TDGF1/UMOD/ADORA1/CALCA/CCL1/F7/FGFBP1/INSM1/SNAI2/GREM1/HAND2/PLAU/SCG2/FGF16/GDNF/PHOX2B/PPARGC1A/S100A7/WNT7A/DEFB1/FGF4/CHGA/ASCL1/COL5A1/ITGA11/KIT/COL1A2/VTN/TAC1/COL1A1/ITGB3/F2 | 51 | BP |
| GO:0051674 | localization of cell | 51/397 | 0.0060541 | 0.04412578 | 0.03437146 | EMX2/LHX1/MAG/NRG4/PSG2/SLC7A9/SRPX2/ANGPTL3/AZU1/CCKAR/CGA/CXCL14/CXCL6/ELANE/EREG/NEUROD4/RET/S100A2/SOX1/TDGF1/UMOD/ADORA1/CALCA/CCL1/F7/FGFBP1/INSM1/SNAI2/GREM1/HAND2/PLAU/SCG2/FGF16/GDNF/PHOX2B/PPARGC1A/S100A7/WNT7A/DEFB1/FGF4/CHGA/ASCL1/COL5A1/ITGA11/KIT/COL1A2/VTN/TAC1/COL1A1/ITGB3/F2 | 51 | BP |
| GO:0016655 | oxidoreductase activity, acting on NAD(P)H, quinone or similar compound as acceptor | 5/372 | 0.00812126 | 0.04435457 | 0.0339975 | AKR1C4/AKR1C2/CBR1/AKR1C1/AKR1C3 | 5 | MF |
| GO:0009855 | determination of bilateral symmetry | 8/397 | 0.00612398 | 0.04456632 | 0.03471462 | HAND1/NKX3-2/ZIC3/NKX2-5/FOXN4/GREM2/GREM1/HAND2 | 8 | BP |
| GO:0048037 | cofactor binding | 18/372 | 0.00826557 | 0.04479814 | 0.0343375 | HAO1/IYD/ALDH1A3/AOC1/GLDC/OGDHL/G6PD/DDC/GPT2/TXNRD1/GCLC/GPT/UGDH/AMBP/CBR3/CYP26A1/TH/CYP1A1 | 18 | MF |
| GO:0055080 | cation homeostasis | 23/397 | 0.00630771 | 0.0458328 | 0.03570114 | CALCB/CCKBR/CCL15/GALR1/RHAG/SPX/TRPM8/BMP6/CASR/ELANE/GLP1R/KCNK3/ADORA1/CALCA/CCL1/TF/CHRNA9/KL/PTH/GRIN2A/TAC1/KNG1/F2 | 23 | BP |
| GO:0009799 | specification of symmetry | 8/397 | 0.00642352 | 0.0466026 | 0.03630077 | HAND1/NKX3-2/ZIC3/NKX2-5/FOXN4/GREM2/GREM1/HAND2 | 8 | BP |
| GO:0098869 | cellular oxidant detoxification | 7/397 | 0.00647975 | 0.04693846 | 0.03656238 | IYD/SRXN1/TXNRD1/APOA4/GPX2/NQO1/GSTA1 | 7 | BP |
| GO:0010259 | multicellular organism aging | 4/397 | 0.00652067 | 0.0470904 | 0.03668073 | TP63/DDC/COMP/TH | 4 | BP |
| GO:0018149 | peptide cross-linking | 4/397 | 0.00652067 | 0.0470904 | 0.03668073 | IVL/SPRR2E/SPRR1A/SPRR1B | 4 | BP |
| GO:0034358 | plasma lipoprotein particle | 4/403 | 0.00804212 | 0.04764958 | 0.03724773 | APOF/APOA4/APOA5/APOC3 | 4 | CC |
| GO:1990777 | lipoprotein particle | 4/403 | 0.00804212 | 0.04764958 | 0.03724773 | APOF/APOA4/APOA5/APOC3 | 4 | CC |
| GO:0046677 | response to antibiotic | 15/397 | 0.00671457 | 0.04813441 | 0.03749396 | CLDN18/KCNC2/TNC/AOC1/AQP1/CHRNB2/G6PD/NEFL/PTH/GRIN2A/NQO1/TH/COL1A1/UGT1A1/CYP1A1 | 15 | BP |
| GO:0035296 | regulation of tube diameter | 8/397 | 0.00673405 | 0.04813441 | 0.03749396 | CASR/ADORA1/COMP/CHGA/FGB/KNG1/FGG/FGA | 8 | BP |
| GO:0097746 | regulation of blood vessel diameter | 8/397 | 0.00673405 | 0.04813441 | 0.03749396 | CASR/ADORA1/COMP/CHGA/FGB/KNG1/FGG/FGA | 8 | BP |
| GO:0001541 | ovarian follicle development | 5/397 | 0.00677395 | 0.04813441 | 0.03749396 | INHA/PTX3/VGF/EREG/KIT | 5 | BP |
| GO:1902803 | regulation of synaptic vesicle transport | 6/397 | 0.00677599 | 0.04813441 | 0.03749396 | STXBP5L/SYT4/CHRNA5/CHRNB2/WNT7A/SYP | 6 | BP |
| GO:0016079 | synaptic vesicle exocytosis | 7/397 | 0.00683852 | 0.04813441 | 0.03749396 | STXBP5L/SYT4/CHRNA5/CHRNB2/WNT7A/SYP/SNAP25 | 7 | BP |
| GO:0046545 | development of primary female sexual characteristics | 7/397 | 0.00683852 | 0.04813441 | 0.03749396 | INHA/PTX3/VGF/ZFP42/EREG/KIT/AFP | 7 | BP |
| GO:0009309 | amine biosynthetic process | 3/397 | 0.00688649 | 0.04813441 | 0.03749396 | ODC1/TPH2/DDC | 3 | BP |
| GO:0021533 | cell differentiation in hindbrain | 3/397 | 0.00688649 | 0.04813441 | 0.03749396 | LHX1/PHOX2B/WNT7A | 3 | BP |
| GO:0045653 | negative regulation of megakaryocyte differentiation | 3/397 | 0.00688649 | 0.04813441 | 0.03749396 | H4C1/H4C13/H4C6 | 3 | BP |
| GO:0055083 | monovalent inorganic anion homeostasis | 3/397 | 0.00688649 | 0.04813441 | 0.03749396 | SFRP4/ABCC2/PTH | 3 | BP |
| GO:0071371 | cellular response to gonadotropin stimulus | 3/397 | 0.00688649 | 0.04813441 | 0.03749396 | SLC5A5/GCLC/PPARGC1A | 3 | BP |
| GO:0002775 | antimicrobial peptide production | 2/397 | 0.00693021 | 0.04813441 | 0.03749396 | ELANE/KLK3 | 2 | BP |
| GO:0002778 | antibacterial peptide production | 2/397 | 0.00693021 | 0.04813441 | 0.03749396 | ELANE/KLK3 | 2 | BP |
| GO:0003278 | apoptotic process involved in heart morphogenesis | 2/397 | 0.00693021 | 0.04813441 | 0.03749396 | NKX2-5/HAND2 | 2 | BP |
| GO:0016093 | polyprenol metabolic process | 2/397 | 0.00693021 | 0.04813441 | 0.03749396 | AKR1B10/AKR1C3 | 2 | BP |
| GO:0033603 | positive regulation of dopamine secretion | 2/397 | 0.00693021 | 0.04813441 | 0.03749396 | CHRNB2/GDNF | 2 | BP |
| GO:0035634 | response to stilbenoid | 2/397 | 0.00693021 | 0.04813441 | 0.03749396 | APOA4/PPARGC1A | 2 | BP |
| GO:0043152 | induction of bacterial agglutination | 2/397 | 0.00693021 | 0.04813441 | 0.03749396 | FGB/FGA | 2 | BP |
| GO:0045110 | intermediate filament bundle assembly | 2/397 | 0.00693021 | 0.04813441 | 0.03749396 | NEFL/KRT14 | 2 | BP |
| GO:0046137 | negative regulation of vitamin metabolic process | 2/397 | 0.00693021 | 0.04813441 | 0.03749396 | SNAI2/AKR1C3 | 2 | BP |
| GO:0060676 | ureteric bud formation | 2/397 | 0.00693021 | 0.04813441 | 0.03749396 | GREM1/GDNF | 2 | BP |
| GO:0098700 | neurotransmitter loading into synaptic vesicle | 2/397 | 0.00693021 | 0.04813441 | 0.03749396 | DDC/TH | 2 | BP |
| GO:1901374 | acetate ester transport | 2/397 | 0.00693021 | 0.04813441 | 0.03749396 | CHRNA3/SLC18A3 | 2 | BP |
| GO:1904304 | regulation of gastro-intestinal system smooth muscle contraction | 2/397 | 0.00693021 | 0.04813441 | 0.03749396 | SPX/KIT | 2 | BP |
| GO:1904306 | positive regulation of gastro-intestinal system smooth muscle contraction | 2/397 | 0.00693021 | 0.04813441 | 0.03749396 | SPX/KIT | 2 | BP |
| GO:0001848 | complement binding | 3/372 | 0.00903449 | 0.04822924 | 0.03696741 | C8G/PTX3/C8A | 3 | MF |
| GO:0008519 | ammonium transmembrane transporter activity | 3/372 | 0.00903449 | 0.04822924 | 0.03696741 | RHAG/AQP1/SLC18A3 | 3 | MF |
| GO:0048608 | reproductive structure development | 18/397 | 0.0070464 | 0.04886293 | 0.03806144 | HAND1/INHA/LHX1/NR0B1/PTX3/SRY/TNC/VGF/ZFP42/BMP6/EREG/TP63/CSF2/WNT7A/CDX2/KIT/AFP/AKR1C3 | 18 | BP |
| GO:0060359 | response to ammonium ion | 8/397 | 0.0070558 | 0.04886293 | 0.03806144 | KCNC2/PPP1R1B/AOC1/GLDC/CHRNA3/CHRNB2/GABRA1/TAC1 | 8 | BP |
| GO:0007601 | visual perception | 11/397 | 0.00709965 | 0.04909462 | 0.0382419 | ADGRV1/KERA/RGR/GNAT1/GRK1/CHRNB2/TGFBI/COL11A1/COL2A1/TH/COL1A1 | 11 | BP |
| GO:0008188 | neuropeptide receptor activity | 4/372 | 0.00932387 | 0.04940261 | 0.03786679 | GALR1/NTSR2/SSTR1/GAL | 4 | MF |
| GO:0035137 | hindlimb morphogenesis | 4/397 | 0.00723566 | 0.04981637 | 0.03880411 | TP63/GDF5/WNT7A/FGF4 | 4 | BP |
| GO:0071312 | cellular response to alkaloid | 4/397 | 0.00723566 | 0.04981637 | 0.03880411 | CACNA1S/DDC/PPARGC1A/TH | 4 | BP |
| GO:0071542 | dopaminergic neuron differentiation | 4/397 | 0.00723566 | 0.04981637 | 0.03880411 | LMX1A/PHOX2A/OTX2/PHOX2B | 4 | BP |
| GO:0034361 | very-low-density lipoprotein particle | 3/403 | 0.0086559 | 0.05003532 | 0.03911266 | APOA4/APOA5/APOC3 | 3 | CC |
| GO:0034385 | triglyceride-rich plasma lipoprotein particle | 3/403 | 0.0086559 | 0.05003532 | 0.03911266 | APOA4/APOA5/APOC3 | 3 | CC |
| GO:0000788 | nuclear nucleosome | 4/403 | 0.00883828 | 0.05047402 | 0.0394556 | H2AW/H4C1/H4C13/H4C6 | 4 | CC |
| GO:0048699 | generation of neurons | 45/397 | 0.00736603 | 0.0506401 | 0.03944574 | ADGRV1/CTNND2/DPYSL5/EMX2/LHX1/MAG/PAX7/TNC/BMP6/CCKAR/FZD10/GNAT1/KIF5C/LHX2/LMX1A/NEUROD4/NKX2-5/RET/S100B/SH3GL2/SOX1/SOX3/SYT4/CHRNA3/CHRNB2/FOXN4/INSM1/LIN28A/NEFL/PHOX2A/PTF1A/CDH11/GDF5/HAND2/GDNF/OTX2/PHOX2B/WNT7A/ASCL1/NEUROD1/NEUROG3/SNAP25/KIT/TH/F2 | 45 | BP |
| GO:0060078 | regulation of postsynaptic membrane potential | 8/397 | 0.00738902 | 0.05072433 | 0.03951136 | GRIK3/NETO1/ADORA1/CHRNA3/CHRNA5/CHRNB2/CHRNA9/WNT7A | 8 | BP |
| GO:0005579 | membrane attack complex | 2/403 | 0.00907929 | 0.05123316 | 0.04004901 | C8G/C8A | 2 | CC |
| GO:0005496 | steroid binding | 6/372 | 0.00977367 | 0.05140227 | 0.03939952 | APOF/KL/SYP/APOC3/UGT1A1/UGT1A8 | 6 | MF |
| GO:0061458 | reproductive system development | 18/397 | 0.00755048 | 0.05175749 | 0.04031613 | HAND1/INHA/LHX1/NR0B1/PTX3/SRY/TNC/VGF/ZFP42/BMP6/EREG/TP63/CSF2/WNT7A/CDX2/KIT/AFP/AKR1C3 | 18 | BP |
| GO:0099240 | intrinsic component of synaptic membrane | 9/403 | 0.00937979 | 0.0523061 | 0.04088774 | NETO1/GABRG3/KCNC1/ADORA1/CHRNB2/GABRA1/CDH9/CHRNA9/GRIN2A | 9 | CC |
| GO:0022617 | extracellular matrix disassembly | 6/397 | 0.0076741 | 0.05252866 | 0.04091683 | ELANE/MMP10/MMP11/PRSS2/MMP13/MMP3 | 6 | BP |
| GO:0032355 | response to estradiol | 8/397 | 0.00773397 | 0.05286184 | 0.04117635 | PTGFR/F7/PPARGC1A/WNT7A/NQO1/TH/COL1A1/UGT1A1 | 8 | BP |
| GO:0032994 | protein-lipid complex | 4/403 | 0.00968417 | 0.05337554 | 0.04172371 | APOF/APOA4/APOA5/APOC3 | 4 | CC |
| GO:0043583 | ear development | 11/397 | 0.00786529 | 0.05368174 | 0.04181501 | ADGRV1/KCNK2/NKX3-2/ALDH1A3/CXCL14/KCNK3/CHRNA9/PHOX2B/NEUROD1/COL11A1/COL2A1 | 11 | BP |
| GO:0001871 | pattern binding | 3/372 | 0.01037148 | 0.05375002 | 0.04119905 | PTX3/REG4/VTN | 3 | MF |
| GO:0030247 | polysaccharide binding | 3/372 | 0.01037148 | 0.05375002 | 0.04119905 | PTX3/REG4/VTN | 3 | MF |
| GO:0071398 | cellular response to fatty acid | 5/397 | 0.00789384 | 0.05379883 | 0.04190621 | PTGFR/TNC/PPARGC1A/AKR1C2/AKR1C3 | 5 | BP |
| GO:0042417 | dopamine metabolic process | 4/397 | 0.00800049 | 0.05381943 | 0.04192226 | CHRNB2/DDC/GRIN2A/TH | 4 | BP |
| GO:0050982 | detection of mechanical stimulus | 4/397 | 0.00800049 | 0.05381943 | 0.04192226 | ADGRV1/CHRNA9/COL11A1/KIT | 4 | BP |
| GO:0099565 | chemical synaptic transmission, postsynaptic | 7/397 | 0.00800344 | 0.05381943 | 0.04192226 | NETO1/ADORA1/CHRNA3/CHRNA5/CHRNB2/CHRNA9/WNT7A | 7 | BP |
| GO:0003215 | cardiac right ventricle morphogenesis | 3/397 | 0.008045 | 0.05381943 | 0.04192226 | HAND1/NKX2-5/HAND2 | 3 | BP |
| GO:0016048 | detection of temperature stimulus | 3/397 | 0.008045 | 0.05381943 | 0.04192226 | TRPM8/ANO1/ADORA1 | 3 | BP |
| GO:0023019 | signal transduction involved in regulation of gene expression | 3/397 | 0.008045 | 0.05381943 | 0.04192226 | HNF4A/FGF5/NEUROD1 | 3 | BP |
| GO:0031280 | negative regulation of cyclase activity | 3/397 | 0.008045 | 0.05381943 | 0.04192226 | ADGRV1/GALR1/GABBR2 | 3 | BP |
| GO:0034104 | negative regulation of tissue remodeling | 3/397 | 0.008045 | 0.05381943 | 0.04192226 | CLDN18/CALCA/GREM1 | 3 | BP |
| GO:0035728 | response to hepatocyte growth factor | 3/397 | 0.008045 | 0.05381943 | 0.04192226 | CASR/TDGF1/GCLC | 3 | BP |
| GO:0048486 | parasympathetic nervous system development | 3/397 | 0.008045 | 0.05381943 | 0.04192226 | PHOX2A/GDNF/PHOX2B | 3 | BP |
| GO:0071731 | response to nitric oxide | 3/397 | 0.008045 | 0.05381943 | 0.04192226 | KCNC2/AQP1/MMP3 | 3 | BP |
| GO:0072077 | renal vesicle morphogenesis | 3/397 | 0.008045 | 0.05381943 | 0.04192226 | LHX1/GREM1/GDNF | 3 | BP |
| GO:0090190 | positive regulation of branching involved in ureteric bud morphogenesis | 3/397 | 0.008045 | 0.05381943 | 0.04192226 | LHX1/GREM1/GDNF | 3 | BP |
| GO:0051588 | regulation of neurotransmitter transport | 8/397 | 0.00809089 | 0.05404988 | 0.04210177 | STXBP5L/SYT4/CHRNA3/CHRNA5/CHRNB2/GDNF/WNT7A/SYP | 8 | BP |
| GO:0050953 | sensory perception of light stimulus | 11/397 | 0.00813429 | 0.05426308 | 0.04226784 | ADGRV1/KERA/RGR/GNAT1/GRK1/CHRNB2/TGFBI/COL11A1/COL2A1/TH/COL1A1 | 11 | BP |
| GO:0016758 | transferase activity, transferring hexosyl groups | 10/372 | 0.01067731 | 0.05474007 | 0.04195792 | B4GALNT2/UGT2A3/UGT2B11/UGT2B4/UGT1A3/UGT1A1/UGT1A4/UGT1A6/UGT1A7/UGT1A8 | 10 | MF |
| GO:0001227 | DNA-binding transcription repressor activity, RNA polymerase II-specific | 11/372 | 0.01071672 | 0.05474007 | 0.04195792 | ESX1/GLIS1/HAND1/NKX3-2/HMGA2/INSM1/SNAI2/PAX4/CDX2/ASCL1/NEUROG3 | 11 | MF |
| GO:0048839 | inner ear development | 10/397 | 0.00839675 | 0.05593489 | 0.04357008 | ADGRV1/KCNK2/ALDH1A3/CXCL14/KCNK3/CHRNA9/PHOX2B/NEUROD1/COL11A1/COL2A1 | 10 | BP |
| GO:0001894 | tissue homeostasis | 11/397 | 0.00841039 | 0.05594685 | 0.0435794 | ADGRV1/CLDN18/CALCA/TF/MUC13/PTH/MUC6/NEUROD1/MUC2/COL2A1/ITGB3 | 11 | BP |
| GO:0046683 | response to organophosphorus | 8/397 | 0.00846003 | 0.05611901 | 0.0437135 | SLC5A5/VGF/AQP1/PCK1/ALDH3A1/PAX4/AKR1C1/COL1A1 | 8 | BP |
| GO:0050921 | positive regulation of chemotaxis | 8/397 | 0.00846003 | 0.05611901 | 0.0437135 | AZU1/CASR/CXCL14/CCL1/F7/SCG2/FGF16/S100A7 | 8 | BP |
| GO:0090183 | regulation of kidney development | 5/397 | 0.00849933 | 0.05630059 | 0.04385494 | LHX1/RET/LIN28A/GREM1/GDNF | 5 | BP |
| GO:0002793 | positive regulation of peptide secretion | 13/397 | 0.00863623 | 0.05709136 | 0.04447091 | SYBU/VSNL1/ANO1/BMP6/CASR/ADORA1/CCL1/RFX6/ORM2/ORM1/FGB/FGG/FGA | 13 | BP |
| GO:0050886 | endocrine process | 6/397 | 0.00865497 | 0.05709136 | 0.04447091 | GALR1/INHA/BMP6/GAL/CES1/TAC1 | 6 | BP |
| GO:2001023 | regulation of response to drug | 6/397 | 0.00865497 | 0.05709136 | 0.04447091 | GALR1/CHRNA3/CHRNB2/HMGA2/GDNF/GAL | 6 | BP |
| GO:0001755 | neural crest cell migration | 4/397 | 0.0088165 | 0.05783382 | 0.04504925 | RET/HAND2/GDNF/PHOX2B | 4 | BP |
| GO:0006778 | porphyrin-containing compound metabolic process | 4/397 | 0.0088165 | 0.05783382 | 0.04504925 | AMBP/UGT1A1/UGT1A4/CYP1A1 | 4 | BP |
| GO:0046461 | neutral lipid catabolic process | 4/397 | 0.0088165 | 0.05783382 | 0.04504925 | FGF21/APOA4/APOA5/APOC3 | 4 | BP |
| GO:0046464 | acylglycerol catabolic process | 4/397 | 0.0088165 | 0.05783382 | 0.04504925 | FGF21/APOA4/APOA5/APOC3 | 4 | BP |
| GO:0006939 | smooth muscle contraction | 7/397 | 0.00885734 | 0.05802109 | 0.04519512 | SPX/ADORA1/CHRNA3/CHRNB2/GDNF/COMP/KIT | 7 | BP |
| GO:0035580 | specific granule lumen | 5/403 | 0.01079627 | 0.05882104 | 0.04598047 | PTX3/AOC1/ELANE/ORM2/ORM1 | 5 | CC |
| GO:0044282 | small molecule catabolic process | 17/397 | 0.00902992 | 0.05906972 | 0.04601194 | CEL/ENTPD8/HAO1/MTMR7/ENO3/GLDC/HAL/GPT2/NT5E/GPT/HGD/PAH/AKR1B10/CBR3/CYP26A1/AKR1C3/CYP1A1 | 17 | BP |
| GO:0050954 | sensory perception of mechanical stimulus | 9/397 | 0.00911621 | 0.05955166 | 0.04638735 | ADGRV1/CHRNB2/SNAI2/CHRNA9/COL11A1/COL2A1/KIT/TH/COL1A1 | 9 | BP |
| GO:0070527 | platelet aggregation | 5/397 | 0.00913622 | 0.05959993 | 0.04642494 | COMP/FGB/ITGB3/FGG/FGA | 5 | BP |
| GO:0055088 | lipid homeostasis | 8/397 | 0.00923599 | 0.06016767 | 0.04686718 | ANGPTL3/ADORA1/G6PC/HNF4A/APOA4/APOA5/APOC3/AKR1C1 | 8 | BP |
| GO:0021761 | limbic system development | 7/397 | 0.0093085 | 0.06032536 | 0.04699001 | EMX2/NR0B1/ALDH1A3/LMX1A/SOX3/NEFL/NEUROD1 | 7 | BP |
| GO:0043279 | response to alkaloid | 7/397 | 0.0093085 | 0.06032536 | 0.04699001 | PPP1R1B/CHRNB2/CACNA1S/DDC/PPARGC1A/TAC1/TH | 7 | BP |
| GO:0003323 | type B pancreatic cell development | 3/397 | 0.00931128 | 0.06032536 | 0.04699001 | BMP6/INSM1/HNF4A | 3 | BP |
| GO:0072087 | renal vesicle development | 3/397 | 0.00931128 | 0.06032536 | 0.04699001 | LHX1/GREM1/GDNF | 3 | BP |
| GO:2000147 | positive regulation of cell motility | 21/397 | 0.00940543 | 0.06085185 | 0.04740011 | SRPX2/ANGPTL3/CGA/CXCL14/ELANE/RET/TDGF1/CCL1/F7/FGFBP1/INSM1/SNAI2/PLAU/FGF16/S100A7/WNT7A/DEFB1/VTN/TAC1/COL1A1/ITGB3 | 21 | BP |
| GO:0042629 | mast cell granule | 3/403 | 0.01132732 | 0.06101309 | 0.047694 | CPLX2/CHGA/KIT | 3 | CC |
| GO:0009750 | response to fructose | 2/397 | 0.00956067 | 0.06102036 | 0.04753137 | PCK1/PPARGC1A | 2 | BP |
| GO:0009820 | alkaloid metabolic process | 2/397 | 0.00956067 | 0.06102036 | 0.04753137 | DDC/TH | 2 | BP |
| GO:0022605 | mammalian oogenesis stage | 2/397 | 0.00956067 | 0.06102036 | 0.04753137 | PTX3/EREG | 2 | BP |
| GO:0032222 | regulation of synaptic transmission, cholinergic | 2/397 | 0.00956067 | 0.06102036 | 0.04753137 | CHRNA3/TAC1 | 2 | BP |
| GO:0032762 | mast cell cytokine production | 2/397 | 0.00956067 | 0.06102036 | 0.04753137 | CHGA/KIT | 2 | BP |
| GO:0035630 | bone mineralization involved in bone maturation | 2/397 | 0.00956067 | 0.06102036 | 0.04753137 | GREM1/PTH | 2 | BP |
| GO:0051005 | negative regulation of lipoprotein lipase activity | 2/397 | 0.00956067 | 0.06102036 | 0.04753137 | ANGPTL3/APOC3 | 2 | BP |
| GO:0072197 | ureter morphogenesis | 2/397 | 0.00956067 | 0.06102036 | 0.04753137 | EMX2/LHX1 | 2 | BP |
| GO:0097065 | anterior head development | 2/397 | 0.00956067 | 0.06102036 | 0.04753137 | LHX1/COL2A1 | 2 | BP |
| GO:1990048 | anterograde neuronal dense core vesicle transport | 2/397 | 0.00956067 | 0.06102036 | 0.04753137 | SYBU/KIF1A | 2 | BP |
| GO:0044242 | cellular lipid catabolic process | 10/397 | 0.00968039 | 0.06148149 | 0.04789057 | CEL/HAO1/ANGPTL3/FGF21/APOA4/APOA5/AKR1B10/CYP26A1/APOC3/AKR1C3 | 10 | BP |
| GO:0032350 | regulation of hormone metabolic process | 4/397 | 0.00968499 | 0.06148149 | 0.04789057 | BMP6/PPARGC1A/GAL/AKR1C3 | 4 | BP |
| GO:0032941 | secretion by tissue | 4/397 | 0.00968499 | 0.06148149 | 0.04789057 | CEL/AQP1/ADORA1/TAC1 | 4 | BP |
| GO:0033280 | response to vitamin D | 4/397 | 0.00968499 | 0.06148149 | 0.04789057 | TNC/CASR/SNAI2/PTH | 4 | BP |
| GO:0042734 | presynaptic membrane | 8/403 | 0.0115637 | 0.06158645 | 0.0481422 | KCNC2/KCNC1/ADORA1/CHRNB2/CDH9/GRIN2A/SYP/SNAP25 | 8 | CC |
| GO:0001947 | heart looping | 5/397 | 0.00980526 | 0.06207809 | 0.04835529 | HAND1/ZIC3/NKX2-5/FOXN4/HAND2 | 5 | BP |
| GO:1901616 | organic hydroxy compound catabolic process | 5/397 | 0.00980526 | 0.06207809 | 0.04835529 | CEL/HAO1/MTMR7/AKR1B10/AKR1C3 | 5 | BP |
| GO:0150034 | distal axon | 12/403 | 0.01182327 | 0.06216151 | 0.04859172 | GRIK3/KCNC2/KCNK2/CASR/KCNC1/KIF5C/ADORA1/NEFL/OTX2/SYP/SNAP25/TH | 12 | CC |
| GO:1902710 | GABA receptor complex | 2/403 | 0.01193396 | 0.06216151 | 0.04859172 | GABRA1/GABBR2 | 2 | CC |
| GO:0043184 | vascular endothelial growth factor receptor 2 binding | 2/372 | 0.01229982 | 0.06237767 | 0.04781209 | GREM1/ITGB3 | 2 | MF |
| GO:0006936 | muscle contraction | 15/397 | 0.00990563 | 0.06254587 | 0.04871966 | KCNE4/MYBPC1/SPX/MYL1/NEB/NKX2-5/ADORA1/CHRNA3/CHRNB2/MYBPH/CACNA1S/GDNF/COMP/CHGA/KIT | 15 | BP |
| GO:0032102 | negative regulation of response to external stimulus | 15/397 | 0.00990563 | 0.06254587 | 0.04871966 | SPX/ELANE/ADORA1/SNAI2/GREM1/NT5E/PLAT/PLAU/PROC/VTN/FGB/KNG1/FGG/F2/FGA | 15 | BP |
| GO:0002526 | acute inflammatory response | 11/397 | 0.0102228 | 0.06446231 | 0.05021246 | C8G/CPN1/ADORA1/C8A/CFHR5/ORM2/ORM1/VTN/TAC1/F2/UGT1A1 | 11 | BP |
| GO:0044305 | calyx of Held | 3/403 | 0.01282144 | 0.06605828 | 0.05163783 | KCNK2/KCNC1/ADORA1 | 3 | CC |
| GO:0030501 | positive regulation of bone mineralization | 4/397 | 0.01060719 | 0.06650332 | 0.05180229 | ADGRV1/BMP6/KL/PTH | 4 | BP |
| GO:0050433 | regulation of catecholamine secretion | 4/397 | 0.01060719 | 0.06650332 | 0.05180229 | SYT4/CHRNB2/GDNF/CHGA | 4 | BP |
| GO:0090207 | regulation of triglyceride metabolic process | 4/397 | 0.01060719 | 0.06650332 | 0.05180229 | FGF21/APOA4/APOA5/APOC3 | 4 | BP |
| GO:0010893 | positive regulation of steroid biosynthetic process | 3/397 | 0.01068728 | 0.06650332 | 0.05180229 | BMP6/CGA/PPARGC1A | 3 | BP |
| GO:0021513 | spinal cord dorsal/ventral patterning | 3/397 | 0.01068728 | 0.06650332 | 0.05180229 | SOX1/FOXN4/ASCL1 | 3 | BP |
| GO:0042749 | regulation of circadian sleep/wake cycle | 3/397 | 0.01068728 | 0.06650332 | 0.05180229 | ADORA1/CHRNB2/CSF2 | 3 | BP |
| GO:0050802 | circadian sleep/wake cycle, sleep | 3/397 | 0.01068728 | 0.06650332 | 0.05180229 | ADORA1/CHRNB2/CSF2 | 3 | BP |
| GO:0050951 | sensory perception of temperature stimulus | 3/397 | 0.01068728 | 0.06650332 | 0.05180229 | TRPM8/ANO1/ADORA1 | 3 | BP |
| GO:0090208 | positive regulation of triglyceride metabolic process | 3/397 | 0.01068728 | 0.06650332 | 0.05180229 | FGF21/APOA4/APOA5 | 3 | BP |
| GO:1903010 | regulation of bone development | 3/397 | 0.01068728 | 0.06650332 | 0.05180229 | CLDN18/GREM1/PTH | 3 | BP |
| GO:0044304 | main axon | 5/403 | 0.01308122 | 0.06667201 | 0.05211758 | KCNC2/MAG/KCNC1/ADORA1/KCNQ2 | 5 | CC |
| GO:0019865 | immunoglobulin binding | 3/372 | 0.01337251 | 0.06686255 | 0.05124972 | FCER1A/UMOD/AMBP | 3 | MF |
| GO:0071855 | neuropeptide receptor binding | 3/372 | 0.01337251 | 0.06686255 | 0.05124972 | CCKBR/UCN2/GAL | 3 | MF |
| GO:0042737 | drug catabolic process | 7/397 | 0.0107629 | 0.06688574 | 0.05210018 | CPN1/HAO1/GLDC/NT5E/APOA4/HGD/PAH | 7 | BP |
| GO:0032589 | neuron projection membrane | 4/403 | 0.01358699 | 0.06851311 | 0.05355678 | ADGRV1/KCNC2/KCNC1/ADORA1 | 4 | CC |
| GO:0035578 | azurophil granule lumen | 6/403 | 0.01393629 | 0.06953474 | 0.05435538 | AZU1/ELANE/PRSS2/S100A7/ORM2/TTR | 6 | CC |
| GO:0003407 | neural retina development | 5/397 | 0.01124277 | 0.06966703 | 0.05426664 | LHX1/NEUROD4/FOXN4/PTF1A/NEUROD1 | 5 | BP |
| GO:0014032 | neural crest cell development | 5/397 | 0.01124277 | 0.06966703 | 0.05426664 | RET/SNAI2/HAND2/GDNF/PHOX2B | 5 | BP |
| GO:0044703 | multi-organism reproductive process | 33/397 | 0.0112547 | 0.06966703 | 0.05426664 | ADAM20/CLGN/GTSF1/KCNU1/NR0B1/PPP1R1B/PSG2/PSG4/PTGFR/PTX3/SPINK1/SSTR1/VGF/ADAM2/DEFB126/EREG/FOXA3/OCA2/PIWIL2/SPAG11B/TAC3/TAF1L/TP63/CALCA/LIN28A/ABCC2/DEFB1/KIT/AFP/AMBP/TAC1/TH/CYP1A1 | 33 | BP |
| GO:0015718 | monocarboxylic acid transport | 8/397 | 0.01140722 | 0.0705187 | 0.05493004 | LCN12/SLC10A2/SPX/CASR/SLCO1A2/ABCC2/AKR1C4/AKR1C1 | 8 | BP |
| GO:0003012 | muscle system process | 18/397 | 0.01145996 | 0.07075213 | 0.05511187 | KCNE4/MYBPC1/SPX/MYL1/NEB/NKX2-5/ADORA1/CHRNA3/CHRNB2/G6PD/MYBPH/CACNA1S/HAND2/GDNF/PPARGC1A/COMP/CHGA/KIT | 18 | BP |
| GO:0030335 | positive regulation of cell migration | 20/397 | 0.01148866 | 0.07078375 | 0.0551365 | SRPX2/ANGPTL3/CGA/CXCL14/ELANE/RET/TDGF1/CCL1/F7/FGFBP1/INSM1/SNAI2/PLAU/FGF16/S100A7/WNT7A/VTN/TAC1/COL1A1/ITGB3 | 20 | BP |
| GO:0070542 | response to fatty acid | 6/397 | 0.01149505 | 0.07078375 | 0.0551365 | PTGFR/TNC/GLDC/PPARGC1A/AKR1C2/AKR1C3 | 6 | BP |
| GO:0031279 | regulation of cyclase activity | 4/397 | 0.01158427 | 0.07124022 | 0.05549206 | ADGRV1/GALR1/GUCA2B/GABBR2 | 4 | BP |
| GO:0030003 | cellular cation homeostasis | 20/397 | 0.01194404 | 0.07335721 | 0.05714107 | CALCB/CCKBR/CCL15/GALR1/TRPM8/BMP6/CASR/ELANE/GLP1R/KCNK3/ADORA1/CALCA/CCL1/TF/CHRNA9/PTH/GRIN2A/TAC1/KNG1/F2 | 20 | BP |
| GO:0038034 | signal transduction in absence of ligand | 5/397 | 0.01201267 | 0.07353037 | 0.05727596 | RET/CSF2/SNAI2/GDNF/COL2A1 | 5 | BP |
| GO:0097192 | extrinsic apoptotic signaling pathway in absence of ligand | 5/397 | 0.01201267 | 0.07353037 | 0.05727596 | RET/CSF2/SNAI2/GDNF/COL2A1 | 5 | BP |
| GO:0050714 | positive regulation of protein secretion | 12/397 | 0.01210874 | 0.07353037 | 0.05727596 | SYBU/VSNL1/ANO1/BMP6/CASR/CCL1/RFX6/ORM2/ORM1/FGB/FGG/FGA | 12 | BP |
| GO:0050830 | defense response to Gram-positive bacterium | 6/397 | 0.01213341 | 0.07353037 | 0.05727596 | RNASE7/DEFA5/LCE3A/DEFB1/DEFB4A/CHGA | 6 | BP |
| GO:0008299 | isoprenoid biosynthetic process | 3/397 | 0.01217462 | 0.07353037 | 0.05727596 | ALDH1A3/AKR1C3/CYP1A1 | 3 | BP |
| GO:0010842 | retina layer formation | 3/397 | 0.01217462 | 0.07353037 | 0.05727596 | LHX1/FOXN4/PTF1A | 3 | BP |
| GO:0035584 | calcium-mediated signaling using intracellular calcium source | 3/397 | 0.01217462 | 0.07353037 | 0.05727596 | PTGFR/AZU1/DEFB1 | 3 | BP |
| GO:0035813 | regulation of renal sodium excretion | 3/397 | 0.01217462 | 0.07353037 | 0.05727596 | SPX/ADORA1/TAC1 | 3 | BP |
| GO:0045109 | intermediate filament organization | 3/397 | 0.01217462 | 0.07353037 | 0.05727596 | NEFL/KRT14/KRT20 | 3 | BP |
| GO:0048799 | animal organ maturation | 3/397 | 0.01217462 | 0.07353037 | 0.05727596 | RET/GREM1/PTH | 3 | BP |
| GO:0051350 | negative regulation of lyase activity | 3/397 | 0.01217462 | 0.07353037 | 0.05727596 | ADGRV1/GALR1/GABBR2 | 3 | BP |
| GO:0060390 | regulation of SMAD protein signal transduction | 3/397 | 0.01217462 | 0.07353037 | 0.05727596 | BMP6/CILP/GDF5 | 3 | BP |
| GO:0061213 | positive regulation of mesonephros development | 3/397 | 0.01217462 | 0.07353037 | 0.05727596 | LHX1/GREM1/GDNF | 3 | BP |
| GO:0051272 | positive regulation of cellular component movement | 21/397 | 0.01251748 | 0.07430383 | 0.05787844 | SRPX2/ANGPTL3/CGA/CXCL14/ELANE/RET/TDGF1/CCL1/F7/FGFBP1/INSM1/SNAI2/PLAU/FGF16/S100A7/WNT7A/DEFB1/VTN/TAC1/COL1A1/ITGB3 | 21 | BP |
| GO:0001547 | antral ovarian follicle growth | 2/397 | 0.01256184 | 0.07430383 | 0.05787844 | PTX3/EREG | 2 | BP |
| GO:0002072 | optic cup morphogenesis involved in camera-type eye development | 2/397 | 0.01256184 | 0.07430383 | 0.05787844 | ALDH1A3/WNT16 | 2 | BP |
| GO:0032253 | dense core granule localization | 2/397 | 0.01256184 | 0.07430383 | 0.05787844 | SYBU/KIF1A | 2 | BP |
| GO:0042045 | epithelial fluid transport | 2/397 | 0.01256184 | 0.07430383 | 0.05787844 | AQP1/CSF2 | 2 | BP |
| GO:0045188 | regulation of circadian sleep/wake cycle, non-REM sleep | 2/397 | 0.01256184 | 0.07430383 | 0.05787844 | ADORA1/CHRNB2 | 2 | BP |
| GO:0046877 | regulation of saliva secretion | 2/397 | 0.01256184 | 0.07430383 | 0.05787844 | AQP1/TAC1 | 2 | BP |
| GO:0060073 | micturition | 2/397 | 0.01256184 | 0.07430383 | 0.05787844 | CHRNA3/CHRNB2 | 2 | BP |
| GO:0060290 | transdifferentiation | 2/397 | 0.01256184 | 0.07430383 | 0.05787844 | INSM1/NEUROG3 | 2 | BP |
| GO:0061101 | neuroendocrine cell differentiation | 2/397 | 0.01256184 | 0.07430383 | 0.05787844 | INSM1/ASCL1 | 2 | BP |
| GO:0061476 | response to anticoagulant | 2/397 | 0.01256184 | 0.07430383 | 0.05787844 | AOC1/F7 | 2 | BP |
| GO:0071420 | cellular response to histamine | 2/397 | 0.01256184 | 0.07430383 | 0.05787844 | AOC1/GABRA1 | 2 | BP |
| GO:0071864 | positive regulation of cell proliferation in bone marrow | 2/397 | 0.01256184 | 0.07430383 | 0.05787844 | HMGA2/PTH | 2 | BP |
| GO:0099519 | dense core granule cytoskeletal transport | 2/397 | 0.01256184 | 0.07430383 | 0.05787844 | SYBU/KIF1A | 2 | BP |
| GO:1901950 | dense core granule transport | 2/397 | 0.01256184 | 0.07430383 | 0.05787844 | SYBU/KIF1A | 2 | BP |
| GO:2000698 | positive regulation of epithelial cell differentiation involved in kidney development | 2/397 | 0.01256184 | 0.07430383 | 0.05787844 | LHX1/GDNF | 2 | BP |
| GO:0006692 | prostanoid metabolic process | 4/397 | 0.01261733 | 0.07430383 | 0.05787844 | GSTA1/AKR1C2/CBR1/AKR1C3 | 4 | BP |
| GO:0006693 | prostaglandin metabolic process | 4/397 | 0.01261733 | 0.07430383 | 0.05787844 | GSTA1/AKR1C2/CBR1/AKR1C3 | 4 | BP |
| GO:0050432 | catecholamine secretion | 4/397 | 0.01261733 | 0.07430383 | 0.05787844 | SYT4/CHRNB2/GDNF/CHGA | 4 | BP |
| GO:0072210 | metanephric nephron development | 4/397 | 0.01261733 | 0.07430383 | 0.05787844 | LHX1/RET/GREM1/GDNF | 4 | BP |
| GO:0000978 | RNA polymerase II proximal promoter sequence-specific DNA binding | 17/372 | 0.01496798 | 0.07431657 | 0.05696318 | HAND1/MYCN/ZIC3/LHX2/NKX2-5/SOX1/HMGA2/INSM1/PTF1A/SNAI2/HAND2/HNF4A/OTX2/PHOX2B/ASCL1/NEUROD1/NEUROG3 | 17 | MF |
| GO:0007215 | glutamate receptor signaling pathway | 6/397 | 0.0127963 | 0.07526394 | 0.05862631 | GRIK3/NETO1/SSTR1/NEFL/PPARGC1A/GRIN2A | 6 | BP |
| GO:0003680 | AT DNA binding | 2/372 | 0.01558663 | 0.07528235 | 0.05770345 | HMGA2/HAND2 | 2 | MF |
| GO:0010851 | cyclase regulator activity | 2/372 | 0.01558663 | 0.07528235 | 0.05770345 | ADGRV1/GUCA2B | 2 | MF |
| GO:0032052 | bile acid binding | 2/372 | 0.01558663 | 0.07528235 | 0.05770345 | AKR1C2/AKR1C1 | 2 | MF |
| GO:0042166 | acetylcholine binding | 2/372 | 0.01558663 | 0.07528235 | 0.05770345 | CHRNA3/CHRNB2 | 2 | MF |
| GO:0022603 | regulation of anatomical structure morphogenesis | 36/397 | 0.01288186 | 0.07567294 | 0.0589449 | LHX1/MAG/ODAPH/SRPX2/ANGPTL3/AQP1/ENAM/KLK3/RET/S100B/SYT4/CHRNA3/CHRNB2/FGFBP1/HMGA2/NEFL/SNAI2/GREM1/HAND2/HNF4A/DMP1/GDNF/OTX2/PPARGC1A/WNT7A/CDX2/ASPN/COL5A1/GAL/NEUROG3/THBS2/KIT/FGB/FGG/F2/FGA | 36 | BP |
| GO:0001666 | response to hypoxia | 15/397 | 0.01292875 | 0.075854 | 0.05908594 | KCNK2/AQP1/CASR/KCNK3/S100B/ADORA1/CHRNB2/F7/PCK1/PLAT/PLAU/ALDH3A1/PPARGC1A/TH/CYP1A1 | 15 | BP |
| GO:0051817 | modification of morphology or physiology of other organism involved in symbiotic interaction | 7/397 | 0.01295005 | 0.07588471 | 0.05910986 | PTX3/AQP1/AZU1/CXCL6/ELANE/HMGA2/F2 | 7 | BP |
| GO:0098609 | cell-cell adhesion | 27/397 | 0.01316817 | 0.07706724 | 0.06003098 | ADGRV1/B4GALNT2/CDHR2/CLDN18/CLDN9/MAG/SRPX2/CLDN8/COL8A2/DSC3/ELANE/RET/UMOD/CDH17/FGL1/SNAI2/CDH7/NT5E/APOA4/CDH9/COMP/DSG3/KIT/FGB/ITGB3/FGG/FGA | 27 | BP |
| GO:0048167 | regulation of synaptic plasticity | 9/397 | 0.01322796 | 0.07732137 | 0.06022894 | CPLX2/NETO1/S100B/SYT4/ADORA1/GRIN2A/SYP/SNAP25/KIT | 9 | BP |
| GO:0045807 | positive regulation of endocytosis | 8/397 | 0.01339849 | 0.07822134 | 0.06092996 | PTX3/AZU1/SFRP4/H1-1/GREM1/TF/APOA5/VTN | 8 | BP |
| GO:0048812 | neuron projection morphogenesis | 21/397 | 0.01347298 | 0.07855911 | 0.06119306 | CTNND2/DPYSL5/LHX1/MAG/CCKAR/KIF5C/LHX2/LMX1A/RET/S100B/SH3GL2/SYT4/CHRNA3/CHRNB2/NEFL/CDH11/GDNF/OTX2/PHOX2B/WNT7A/NEUROG3 | 21 | BP |
| GO:0042752 | regulation of circadian rhythm | 7/397 | 0.01354331 | 0.07885787 | 0.06142578 | PASD1/PIWIL2/ADORA1/CHRNB2/CSF2/HNF4A/PPARGC1A | 7 | BP |
| GO:0070482 | response to oxygen levels | 16/397 | 0.01355921 | 0.07885787 | 0.06142578 | KCNK2/AQP1/CASR/KCNK3/S100B/ADORA1/CHRNB2/F7/PCK1/PLAT/PLAU/ALDH3A1/PPARGC1A/TH/COL1A1/CYP1A1 | 16 | BP |
| GO:0045944 | positive regulation of transcription by RNA polymerase II | 34/397 | 0.01357731 | 0.07885787 | 0.06142578 | GALR1/GLIS1/HAND1/MYCN/PAX7/ZIC3/BEX1/BMP6/CGA/FOXA3/LMX1A/NKX2-5/SOX1/TP63/HMGA2/PCK1/PHOX2A/PTF1A/GREM1/HAND2/HNF4A/RFX6/GDNF/OTX2/PHOX2B/PPARGC1A/WNT7A/FGF4/PTH/ASCL1/GAL/HNF1A/NEUROD1/NEUROG3 | 34 | BP |
| GO:0048663 | neuron fate commitment | 5/397 | 0.01365819 | 0.07885787 | 0.06142578 | PAX7/SOX1/FOXN4/PTF1A/ASCL1 | 5 | BP |
| GO:0061371 | determination of heart left/right asymmetry | 5/397 | 0.01365819 | 0.07885787 | 0.06142578 | HAND1/ZIC3/NKX2-5/FOXN4/HAND2 | 5 | BP |
| GO:0017001 | antibiotic catabolic process | 4/397 | 0.01370745 | 0.07885787 | 0.06142578 | APOA4/AKR1B10/AKR1C3/UGT1A1 | 4 | BP |
| GO:0007530 | sex determination | 3/397 | 0.01377467 | 0.07885787 | 0.06142578 | NR0B1/SRY/SOX3 | 3 | BP |
| GO:0009404 | toxin metabolic process | 3/397 | 0.01377467 | 0.07885787 | 0.06142578 | DDC/TH/CYP1A1 | 3 | BP |
| GO:0016338 | calcium-independent cell-cell adhesion via plasma membrane cell-adhesion molecules | 3/397 | 0.01377467 | 0.07885787 | 0.06142578 | CLDN18/CLDN9/CLDN8 | 3 | BP |
| GO:0021511 | spinal cord patterning | 3/397 | 0.01377467 | 0.07885787 | 0.06142578 | SOX1/FOXN4/ASCL1 | 3 | BP |
| GO:0042430 | indole-containing compound metabolic process | 3/397 | 0.01377467 | 0.07885787 | 0.06142578 | TPH2/DDC/GRIN2A | 3 | BP |
| GO:0043576 | regulation of respiratory gaseous exchange | 3/397 | 0.01377467 | 0.07885787 | 0.06142578 | ADORA1/PHOX2A/PHOX2B | 3 | BP |
| GO:0051953 | negative regulation of amine transport | 3/397 | 0.01377467 | 0.07885787 | 0.06142578 | SYT4/ADORA1/CHGA | 3 | BP |
| GO:0071305 | cellular response to vitamin D | 3/397 | 0.01377467 | 0.07885787 | 0.06142578 | TNC/CASR/SNAI2 | 3 | BP |
| GO:0090189 | regulation of branching involved in ureteric bud morphogenesis | 3/397 | 0.01377467 | 0.07885787 | 0.06142578 | LHX1/GREM1/GDNF | 3 | BP |
| GO:0044463 | cell projection part | 40/403 | 0.01638997 | 0.08009122 | 0.06260739 | ADGRV1/CDHR2/CTNND2/DPYSL5/GRIK3/GUCA2B/INHA/KCNC2/KCNK2/MAG/PPP1R1B/SLC6A19/SLC7A9/SYBU/AQP1/CASR/GNAT1/GRK1/KCNC1/KIF5C/PCSK2/RET/SH3GL2/SYT4/TP63/UMOD/ADORA1/CHRNA3/KIF1A/NEFL/KCNQ2/OTX2/PPARGC1A/ABCC2/DEFB1/NQO1/SYP/SNAP25/TH/ITGB3 | 40 | CC |
| GO:0120038 | plasma membrane bounded cell projection part | 40/403 | 0.01638997 | 0.08009122 | 0.06260739 | ADGRV1/CDHR2/CTNND2/DPYSL5/GRIK3/GUCA2B/INHA/KCNC2/KCNK2/MAG/PPP1R1B/SLC6A19/SLC7A9/SYBU/AQP1/CASR/GNAT1/GRK1/KCNC1/KIF5C/PCSK2/RET/SH3GL2/SYT4/TP63/UMOD/ADORA1/CHRNA3/KIF1A/NEFL/KCNQ2/OTX2/PPARGC1A/ABCC2/DEFB1/NQO1/SYP/SNAP25/TH/ITGB3 | 40 | CC |
| GO:0070279 | vitamin B6 binding | 4/372 | 0.01670598 | 0.08014353 | 0.06142951 | GLDC/DDC/GPT2/GPT | 4 | MF |
| GO:0000904 | cell morphogenesis involved in differentiation | 23/397 | 0.01412765 | 0.08078074 | 0.06292358 | CTNND2/DPYSL5/LHX1/MAG/CCKAR/KIF5C/LHX2/LMX1A/RET/S100B/CHRNA3/CHRNB2/NEFL/CDH11/GDNF/OTX2/PHOX2B/WNT7A/NEUROG3/FGB/ITGB3/FGG/FGA | 23 | BP |
| GO:0008509 | anion transmembrane transporter activity | 11/372 | 0.01715321 | 0.08173678 | 0.06265072 | CLCA2/SLC10A2/SLC5A5/SLC6A19/SLC7A9/ANO1/OCA2/GABRA1/SLCO1A2/ABCC2/AKR1C4 | 11 | MF |
| GO:0030100 | regulation of endocytosis | 12/397 | 0.01431567 | 0.08175683 | 0.0636839 | PTX3/AZU1/SFRP4/SH3GL2/SYT4/H1-1/GREM1/TF/APOA5/VTN/APOC3/ITGB3 | 12 | BP |
| GO:0002020 | protease binding | 7/372 | 0.01727764 | 0.08178081 | 0.06268448 | ALPI/ELANE/COMP/KIT/COL1A2/COL1A1/ITGB3 | 7 | MF |
| GO:0005903 | brush border | 6/403 | 0.01692611 | 0.08186712 | 0.06399562 | ANKS4B/CDHR2/SLC6A19/SLC7A9/AQP1/ABCC2 | 6 | CC |
| GO:0003143 | embryonic heart tube morphogenesis | 5/397 | 0.01453515 | 0.08261072 | 0.06434904 | HAND1/ZIC3/NKX2-5/FOXN4/HAND2 | 5 | BP |
| GO:0014031 | mesenchymal cell development | 5/397 | 0.01453515 | 0.08261072 | 0.06434904 | RET/SNAI2/HAND2/GDNF/PHOX2B | 5 | BP |
| GO:0030858 | positive regulation of epithelial cell differentiation | 5/397 | 0.01453515 | 0.08261072 | 0.06434904 | LHX1/BMP6/SFRP4/GDNF/PROC | 5 | BP |
| GO:0071242 | cellular response to ammonium ion | 5/397 | 0.01453515 | 0.08261072 | 0.06434904 | KCNC2/AOC1/CHRNA3/CHRNB2/GABRA1 | 5 | BP |
| GO:0007409 | axonogenesis | 16/397 | 0.01480452 | 0.08356317 | 0.06509094 | DPYSL5/LHX1/MAG/CCKAR/KIF5C/LHX2/LMX1A/RET/S100B/CHRNB2/NEFL/CDH11/GDNF/OTX2/PHOX2B/WNT7A | 16 | BP |
| GO:0032496 | response to lipopolysaccharide | 13/397 | 0.01483823 | 0.08356317 | 0.06509094 | PTGFR/BMP6/CXCL6/ELANE/CSF2/PCK1/PPARGC1A/S100A7/ABCC2/TAC1/TH/UGT1A1/CYP1A1 | 13 | BP |
| GO:0006775 | fat-soluble vitamin metabolic process | 4/397 | 0.01485559 | 0.08356317 | 0.06509094 | SNAI2/CYP26A1/CBR1/CYP1A1 | 4 | BP |
| GO:0008089 | anterograde axonal transport | 4/397 | 0.01485559 | 0.08356317 | 0.06509094 | SYBU/KIF5C/KIF1A/NEFL | 4 | BP |
| GO:0010470 | regulation of gastrulation | 4/397 | 0.01485559 | 0.08356317 | 0.06509094 | LHX1/HNF4A/OTX2/COL5A1 | 4 | BP |
| GO:0043616 | keratinocyte proliferation | 4/397 | 0.01485559 | 0.08356317 | 0.06509094 | EREG/TP63/SNAI2/WNT16 | 4 | BP |
| GO:0051339 | regulation of lyase activity | 4/397 | 0.01485559 | 0.08356317 | 0.06509094 | ADGRV1/GALR1/GUCA2B/GABBR2 | 4 | BP |
| GO:0051602 | response to electrical stimulus | 4/397 | 0.01485559 | 0.08356317 | 0.06509094 | GNAT1/PPARGC1A/NQO1/TH | 4 | BP |
| GO:0048729 | tissue morphogenesis | 23/397 | 0.01486197 | 0.08356317 | 0.06509094 | HAND1/LHX1/PAX7/TNC/ZIC3/ALDH1A3/CASR/LHX2/NKX2-5/RET/TP63/FOXN4/HMGA2/SNAI2/GREM1/HAND2/TXNRD1/GDNF/WNT16/WNT7A/COL5A1/COL11A1/ITGB3 | 23 | BP |
| GO:0034703 | cation channel complex | 9/403 | 0.01754611 | 0.08400863 | 0.06566964 | KCNC2/KCNK2/KCNC1/ABCC8/CACNA1S/KCNQ2/KCNQ5/GRIN2A/SNAP25 | 9 | CC |
| GO:0098742 | cell-cell adhesion via plasma-membrane adhesion molecules | 11/397 | 0.01516779 | 0.08518129 | 0.06635136 | CDHR2/CLDN18/CLDN9/MAG/CLDN8/DSC3/RET/UMOD/CDH17/CDH9/DSG3 | 11 | BP |
| GO:0051704 | multi-organism process | 71/397 | 0.01519079 | 0.08520916 | 0.06637307 | ADAM20/CD207/CLDN9/CLGN/GKN2/GTSF1/KCNU1/NR0B1/PIK3C2G/PPP1R1B/PSG2/PSG4/PTGFR/PTX3/REG4/SLC10A2/SPINK1/SSTR1/VGF/WFDC12/ADAM2/AQP1/AZU1/BMP6/BPIFB2/CXCL6/DEFB126/ELANE/EREG/FOXA3/KLK3/OCA2/PIWIL2/RNASE7/SPAG11B/TAC3/TAF1L/TP63/BPIFA1/CALCA/CCL1/CHRNB2/CSF2/DEFA6/HMGA2/LIN28A/ODC1/PCK1/PRSS2/BPIFA2/DEFA5/HAND2/PPBP/LCE3A/PPARGC1A/S100A7/ABCC2/DEFB1/DEFB4A/CHGA/KIT/AFP/AMBP/FGB/TAC1/TH/ITGB3/F2/UGT1A1/CYP1A1/FGA | 71 | BP |
| GO:0071702 | organic substance transport | 69/397 | 0.01535389 | 0.08602185 | 0.06700611 | CA6/CEL/GALR1/HAO1/KCNC2/KLRF2/LCN12/RHAG/SLC10A2/SLC6A19/SLC7A9/SPX/STXBP5L/SYBU/VGF/VSNL1/ZG16/ANO1/AQP1/BMP6/CASR/GLP1R/KIF5C/OCA2/SYT4/ABCC8/ADORA1/APOF/CCL1/CDH17/CHRNA3/CHRNB2/CSF2/KIF1A/SLC18A3/SLCO1A2/DDC/FGF21/G6PC/HNF4A/PPBP/RFX6/SCG2/TF/APOA4/GDNF/ABCC2/APOA5/COMP/KRT20/ORM2/PTH/CHGA/GAL/HNF1A/NEUROD1/ORM1/CES1/SNAP25/AKR1C4/APOC3/FGB/TAC1/TH/AKR1C1/COL1A1/ITGB3/FGG/FGA | 69 | BP |
| GO:0021871 | forebrain regionalization | 3/397 | 0.01548849 | 0.08616271 | 0.06711583 | EMX2/LHX1/LHX2 | 3 | BP |
| GO:0022410 | circadian sleep/wake cycle process | 3/397 | 0.01548849 | 0.08616271 | 0.06711583 | ADORA1/CHRNB2/CSF2 | 3 | BP |
| GO:0030325 | adrenal gland development | 3/397 | 0.01548849 | 0.08616271 | 0.06711583 | NR0B1/INSM1/ASCL1 | 3 | BP |
| GO:0035812 | renal sodium excretion | 3/397 | 0.01548849 | 0.08616271 | 0.06711583 | SPX/ADORA1/TAC1 | 3 | BP |
| GO:0042481 | regulation of odontogenesis | 3/397 | 0.01548849 | 0.08616271 | 0.06711583 | ODAPH/DMP1/ASPN | 3 | BP |
| GO:0060343 | trabecula formation | 3/397 | 0.01548849 | 0.08616271 | 0.06711583 | NKX2-5/GREM1/COL1A1 | 3 | BP |
| GO:0042133 | neurotransmitter metabolic process | 8/397 | 0.0156327 | 0.08620588 | 0.06714946 | PTX3/GLDC/TPH2/DDC/PAH/GRIN2A/NQO1/TH | 8 | BP |
| GO:0050806 | positive regulation of synaptic transmission | 8/397 | 0.0156327 | 0.08620588 | 0.06714946 | NETO1/S100B/ADORA1/CHRNB2/WNT7A/GRIN2A/SNAP25/TAC1 | 8 | BP |
| GO:0017158 | regulation of calcium ion-dependent exocytosis | 6/397 | 0.01570222 | 0.08620588 | 0.06714946 | STXBP5L/SYT4/CHRNA5/CHRNB2/WNT7A/SYP | 6 | BP |
| GO:0045833 | negative regulation of lipid metabolic process | 6/397 | 0.01570222 | 0.08620588 | 0.06714946 | ADORA1/SNAI2/APOC3/AKR1C3/UGT1A1/UGT1A8 | 6 | BP |
| GO:1901655 | cellular response to ketone | 6/397 | 0.01570222 | 0.08620588 | 0.06714946 | PTGFR/TNC/AQP1/ABCC2/AKR1C2/AKR1C3 | 6 | BP |
| GO:0099003 | vesicle-mediated transport in synapse | 9/397 | 0.01573694 | 0.08620588 | 0.06714946 | STXBP5L/SH3GL2/SYT4/CHRNA5/CHRNB2/WNT7A/SYP/SNAP25/ITGB3 | 9 | BP |
| GO:0010941 | regulation of cell death | 50/397 | 0.01575139 | 0.08620588 | 0.06714946 | KCNK2/MAG/NKX3-2/PAX7/PTGFR/ALDH1A3/AQP1/AZU1/NKX2-5/PRAME/RET/S100B/SFRP4/TDGF1/TP63/ADORA1/CSF2/G6PD/HMGA2/NEFL/SNAI2/FGF21/GDF5/GREM1/HAND2/SCG2/GCLC/GDNF/MMP3/PPARGC1A/WNT16/WNT7A/COMP/FGF4/PAX4/PTH/CHGA/GRIN2A/ASCL1/GAL/NEUROD1/NQO1/PROC/COL2A1/KIT/FGB/KNG1/FGG/AKR1C3/FGA | 50 | BP |
| GO:0007167 | enzyme linked receptor protein signaling pathway | 32/397 | 0.01587427 | 0.08620588 | 0.06714946 | GH2/NRG4/BMP6/CILP/EREG/NKX2-5/RET/SFRP4/SH3GL2/ADORA1/CHRNA3/F7/FGFBP1/GREM2/FGF21/GDF5/GREM1/HNF4A/IGFBP1/KLB/FGF16/FGF5/OTX2/COMP/FGF4/KL/ASPN/KIT/AFP/COL1A2/VTN/ITGB3 | 32 | BP |
| GO:0002158 | osteoclast proliferation | 2/397 | 0.01591606 | 0.08620588 | 0.06714946 | GREM1/PTH | 2 | BP |
| GO:0002551 | mast cell chemotaxis | 2/397 | 0.01591606 | 0.08620588 | 0.06714946 | CHGA/KIT | 2 | BP |
| GO:0014012 | peripheral nervous system axon regeneration | 2/397 | 0.01591606 | 0.08620588 | 0.06714946 | TNC/NEFL | 2 | BP |
| GO:0019896 | axonal transport of mitochondrion | 2/397 | 0.01591606 | 0.08620588 | 0.06714946 | SYBU/NEFL | 2 | BP |
| GO:0034371 | chylomicron remodeling | 2/397 | 0.01591606 | 0.08620588 | 0.06714946 | APOA4/APOC3 | 2 | BP |
| GO:0042748 | circadian sleep/wake cycle, non-REM sleep | 2/397 | 0.01591606 | 0.08620588 | 0.06714946 | ADORA1/CHRNB2 | 2 | BP |
| GO:0045714 | regulation of low-density lipoprotein particle receptor biosynthetic process | 2/397 | 0.01591606 | 0.08620588 | 0.06714946 | FGF21/ITGB3 | 2 | BP |
| GO:0046838 | phosphorylated carbohydrate dephosphorylation | 2/397 | 0.01591606 | 0.08620588 | 0.06714946 | MTMR7/G6PC | 2 | BP |
| GO:0048262 | determination of dorsal/ventral asymmetry | 2/397 | 0.01591606 | 0.08620588 | 0.06714946 | GREM2/GREM1 | 2 | BP |
| GO:0048263 | determination of dorsal identity | 2/397 | 0.01591606 | 0.08620588 | 0.06714946 | GREM2/GREM1 | 2 | BP |
| GO:0048387 | negative regulation of retinoic acid receptor signaling pathway | 2/397 | 0.01591606 | 0.08620588 | 0.06714946 | PRAME/CYP26A1 | 2 | BP |
| GO:0051967 | negative regulation of synaptic transmission, glutamatergic | 2/397 | 0.01591606 | 0.08620588 | 0.06714946 | GRIK3/ADORA1 | 2 | BP |
| GO:0060586 | multicellular organismal iron ion homeostasis | 2/397 | 0.01591606 | 0.08620588 | 0.06714946 | RHAG/BMP6 | 2 | BP |
| GO:0072172 | mesonephric tubule formation | 2/397 | 0.01591606 | 0.08620588 | 0.06714946 | GREM1/GDNF | 2 | BP |
| GO:0090009 | primitive streak formation | 2/397 | 0.01591606 | 0.08620588 | 0.06714946 | LHX1/OTX2 | 2 | BP |
| GO:0005520 | insulin-like growth factor binding | 3/372 | 0.01870842 | 0.08625311 | 0.06611246 | IGFBP6/IGFBP1/ITGB3 | 3 | MF |
| GO:0008028 | monocarboxylic acid transmembrane transporter activity | 3/372 | 0.01870842 | 0.08625311 | 0.06611246 | SLC10A2/SLCO1A2/AKR1C4 | 3 | MF |
| GO:0016628 | oxidoreductase activity, acting on the CH-CH group of donors, NAD or NADP as acceptor | 3/372 | 0.01870842 | 0.08625311 | 0.06611246 | AKR1C2/AKR1C1/AKR1C3 | 3 | MF |
| GO:0048020 | CCR chemokine receptor binding | 3/372 | 0.01870842 | 0.08625311 | 0.06611246 | CCL17/DEFB1/DEFB4A | 3 | MF |
| GO:0016651 | oxidoreductase activity, acting on NAD(P)H | 6/372 | 0.01898877 | 0.08629469 | 0.06614434 | NQO1/AKR1C4/AKR1C2/CBR1/AKR1C1/AKR1C3 | 6 | MF |
| GO:0016917 | GABA receptor activity | 2/372 | 0.01920361 | 0.08629469 | 0.06614434 | GABRA1/GABBR2 | 2 | MF |
| GO:0031994 | insulin-like growth factor I binding | 2/372 | 0.01920361 | 0.08629469 | 0.06614434 | IGFBP1/ITGB3 | 2 | MF |
| GO:0052650 | NADP-retinol dehydrogenase activity | 2/372 | 0.01920361 | 0.08629469 | 0.06614434 | AKR1B10/AKR1C3 | 2 | MF |
| GO:0002548 | monocyte chemotaxis | 4/397 | 0.0160627 | 0.08680106 | 0.06761307 | CALCA/CCL1/GREM1/S100A7 | 4 | BP |
| GO:0043268 | positive regulation of potassium ion transport | 4/397 | 0.0160627 | 0.08680106 | 0.06761307 | KCNC2/KCNC1/ADORA1/GAL | 4 | BP |
| GO:0016331 | morphogenesis of embryonic epithelium | 8/397 | 0.016231 | 0.08761031 | 0.06824343 | HAND1/ALDH1A3/LHX2/RET/TP63/GREM1/GDNF/WNT16 | 8 | BP |
| GO:0015893 | drug transport | 9/397 | 0.01627798 | 0.0877636 | 0.06836283 | GALR1/RHAG/AQP1/CHRNA3/CHRNB2/SLC18A3/GDNF/ABCC2/GAL | 9 | BP |
| GO:0048667 | cell morphogenesis involved in neuron differentiation | 19/397 | 0.01635643 | 0.08808597 | 0.06861394 | CTNND2/DPYSL5/LHX1/MAG/CCKAR/KIF5C/LHX2/LMX1A/RET/S100B/CHRNA3/CHRNB2/NEFL/CDH11/GDNF/OTX2/PHOX2B/WNT7A/NEUROG3 | 19 | BP |
| GO:0099060 | integral component of postsynaptic specialization membrane | 5/403 | 0.01858459 | 0.08809097 | 0.06886081 | NETO1/CHRNB2/GABRA1/CHRNA9/GRIN2A | 5 | CC |
| GO:0036293 | response to decreased oxygen levels | 15/397 | 0.0166275 | 0.08944385 | 0.06967165 | KCNK2/AQP1/CASR/KCNK3/S100B/ADORA1/CHRNB2/F7/PCK1/PLAT/PLAU/ALDH3A1/PPARGC1A/TH/CYP1A1 | 15 | BP |
| GO:0120039 | plasma membrane bounded cell projection morphogenesis | 21/397 | 0.01728225 | 0.09217103 | 0.07179597 | CTNND2/DPYSL5/LHX1/MAG/CCKAR/KIF5C/LHX2/LMX1A/RET/S100B/SH3GL2/SYT4/CHRNA3/CHRNB2/NEFL/CDH11/GDNF/OTX2/PHOX2B/WNT7A/NEUROG3 | 21 | BP |
| GO:0001702 | gastrulation with mouth forming second | 3/397 | 0.01731693 | 0.09217103 | 0.07179597 | LHX1/OTX2/UGDH | 3 | BP |
| GO:0001963 | synaptic transmission, dopaminergic | 3/397 | 0.01731693 | 0.09217103 | 0.07179597 | CHRNB2/GDNF/TH | 3 | BP |
| GO:0002068 | glandular epithelial cell development | 3/397 | 0.01731693 | 0.09217103 | 0.07179597 | BMP6/INSM1/HNF4A | 3 | BP |
| GO:0044062 | regulation of excretion | 3/397 | 0.01731693 | 0.09217103 | 0.07179597 | SPX/ADORA1/TAC1 | 3 | BP |
| GO:0048169 | regulation of long-term neuronal synaptic plasticity | 3/397 | 0.01731693 | 0.09217103 | 0.07179597 | NETO1/SYP/KIT | 3 | BP |
| GO:0048596 | embryonic camera-type eye morphogenesis | 3/397 | 0.01731693 | 0.09217103 | 0.07179597 | ALDH1A3/WNT16/TH | 3 | BP |
| GO:0060561 | apoptotic process involved in morphogenesis | 3/397 | 0.01731693 | 0.09217103 | 0.07179597 | NKX2-5/HAND2/FGF4 | 3 | BP |
| GO:0090025 | regulation of monocyte chemotaxis | 3/397 | 0.01731693 | 0.09217103 | 0.07179597 | CCL1/GREM1/S100A7 | 3 | BP |
| GO:0046850 | regulation of bone remodeling | 4/397 | 0.01732964 | 0.09217103 | 0.07179597 | CLDN18/CALCA/GREM1/ITGB3 | 4 | BP |
| GO:0032024 | positive regulation of insulin secretion | 5/397 | 0.01739031 | 0.09228589 | 0.07188544 | SYBU/VSNL1/ANO1/CASR/RFX6 | 5 | BP |
| GO:0061515 | myeloid cell development | 5/397 | 0.01739031 | 0.09228589 | 0.07188544 | CLDN18/RHAG/G6PD/THPO/KIT | 5 | BP |
| GO:0019825 | oxygen binding | 3/372 | 0.02071235 | 0.0924891 | 0.07089231 | CYP26A1/TH/CYP1A1 | 3 | MF |
| GO:0010565 | regulation of cellular ketone metabolic process | 9/397 | 0.01798294 | 0.09532371 | 0.07425173 | BMP6/ODC1/APOA4/PPARGC1A/APOA5/NQO1/APOC3/AKR1C3/UGT1A8 | 9 | BP |
| GO:0045778 | positive regulation of ossification | 6/397 | 0.01816079 | 0.09605088 | 0.07481815 | ADGRV1/BMP6/TP63/KL/PTH/TAC1 | 6 | BP |
| GO:0060191 | regulation of lipase activity | 6/397 | 0.01816079 | 0.09605088 | 0.07481815 | ANGPTL3/APOA4/APOA5/PTH/KIT/APOC3 | 6 | BP |
| GO:0031253 | cell projection membrane | 13/403 | 0.02074838 | 0.09679096 | 0.0756616 | ADGRV1/CDHR2/KCNC2/SLC6A19/SLC7A9/AQP1/GNAT1/GRK1/KCNC1/UMOD/ADORA1/ABCC2/ITGB3 | 13 | CC |
| GO:0030424 | axon | 19/403 | 0.02082843 | 0.09679096 | 0.0756616 | GRIK3/KCNC2/KCNK2/MAG/NMNAT2/SYBU/CASR/KCNC1/KIF5C/ADORA1/KIF1A/NEFL/DDC/KCNQ2/OTX2/SYP/SNAP25/TAC1/TH | 19 | CC |
| GO:0004867 | serine-type endopeptidase inhibitor activity | 5/372 | 0.02198079 | 0.09729467 | 0.07457575 | SPINK1/SPINK4/WFDC12/WFDC5/AMBP | 5 | MF |
| GO:0000987 | proximal promoter sequence-specific DNA binding | 17/372 | 0.02216908 | 0.09729467 | 0.07457575 | HAND1/MYCN/ZIC3/LHX2/NKX2-5/SOX1/HMGA2/INSM1/PTF1A/SNAI2/HAND2/HNF4A/OTX2/PHOX2B/ASCL1/NEUROD1/NEUROG3 | 17 | MF |
| GO:0016597 | amino acid binding | 4/372 | 0.02219963 | 0.09729467 | 0.07457575 | CASR/DDC/GCLC/TH | 4 | MF |
| GO:0048858 | cell projection morphogenesis | 21/397 | 0.01851292 | 0.09780371 | 0.07618351 | CTNND2/DPYSL5/LHX1/MAG/CCKAR/KIF5C/LHX2/LMX1A/RET/S100B/SH3GL2/SYT4/CHRNA3/CHRNB2/NEFL/CDH11/GDNF/OTX2/PHOX2B/WNT7A/NEUROG3 | 21 | BP |
| GO:0035239 | tube morphogenesis | 30/397 | 0.01855189 | 0.09790009 | 0.07625858 | EMX2/HAND1/LHX1/SRPX2/TNC/ZIC3/ANGPTL3/AQP1/CASR/COL8A2/EREG/KLK3/LHX2/NKX2-5/RET/TDGF1/TP63/FGFBP1/FOXN4/HMGA2/TGFBI/GREM1/HAND2/SCG2/GDNF/S100A7/WNT7A/COMP/THBS2/ITGB3 | 30 | BP |
| GO:0071354 | cellular response to interleukin-6 | 4/397 | 0.01865721 | 0.09819504 | 0.07648833 | TDGF1/PPARGC1A/ABCC2/FGG | 4 | BP |
| GO:1990089 | response to nerve growth factor | 4/397 | 0.01865721 | 0.09819504 | 0.07648833 | KCNC2/KCNC1/SH3GL2/TAC1 | 4 | BP |
| GO:0061564 | axon development | 17/397 | 0.01867015 | 0.09819504 | 0.07648833 | DPYSL5/LHX1/MAG/TNC/CCKAR/KIF5C/LHX2/LMX1A/RET/S100B/CHRNB2/NEFL/CDH11/GDNF/OTX2/PHOX2B/WNT7A | 17 | BP |
| GO:0019842 | vitamin binding | 7/372 | 0.02275876 | 0.09894997 | 0.07584453 | C8G/GLDC/OGDHL/DDC/GPT2/GPT/KL | 7 | MF |
| GO:0005172 | vascular endothelial growth factor receptor binding | 2/372 | 0.02313478 | 0.09894997 | 0.07584453 | GREM1/ITGB3 | 2 | MF |
| GO:0015643 | toxic substance binding | 2/372 | 0.02313478 | 0.09894997 | 0.07584453 | GUCY2C/AZU1 | 2 | MF |
| GO:0050786 | RAGE receptor binding | 2/372 | 0.02313478 | 0.09894997 | 0.07584453 | S100B/S100A7 | 2 | MF |
| GO:0055114 | oxidation-reduction process | 31/397 | 0.01896048 | 0.09961108 | 0.07759135 | FADS6/HAO1/IYD/PIR/ALDH1A3/AOC1/ENO3/GLDC/G6PD/SRXN1/TPH2/G6PC/TXNRD1/ALDH3A1/HGD/PAH/PPARGC1A/KL/PTH/AKR1B10/GPX2/NQO1/UGDH/AKR1C4/CBR3/AKR1C2/CBR1/TH/AKR1C1/AKR1C3/CYP1A1 | 31 | BP |
| GO:0032368 | regulation of lipid transport | 7/397 | 0.01900807 | 0.0997501 | 0.07769964 | GALR1/BMP6/APOA4/GAL/APOC3/TAC1/ITGB3 | 7 | BP |
| GO:0006641 | triglyceride metabolic process | 6/397 | 0.01903569 | 0.09978418 | 0.07772618 | PCK1/FGF21/G6PC/APOA4/APOA5/APOC3 | 6 | BP |
| GO:0007263 | nitric oxide mediated signal transduction | 3/397 | 0.01926059 | 0.09978502 | 0.07772683 | KCNC2/SPINK1/NEUROD1 | 3 | BP |
| GO:0019934 | cGMP-mediated signaling | 3/397 | 0.01926059 | 0.09978502 | 0.07772683 | GUCA2B/KCNC2/AQP1 | 3 | BP |
| GO:0034114 | regulation of heterotypic cell-cell adhesion | 3/397 | 0.01926059 | 0.09978502 | 0.07772683 | FGB/FGG/FGA | 3 | BP |
| GO:0061217 | regulation of mesonephros development | 3/397 | 0.01926059 | 0.09978502 | 0.07772683 | LHX1/GREM1/GDNF | 3 | BP |
| GO:0070168 | negative regulation of biomineral tissue development | 3/397 | 0.01926059 | 0.09978502 | 0.07772683 | GREM1/PTH/ASPN | 3 | BP |
| GO:0072273 | metanephric nephron morphogenesis | 3/397 | 0.01926059 | 0.09978502 | 0.07772683 | LHX1/GREM1/GDNF | 3 | BP |
| GO:1901018 | positive regulation of potassium ion transmembrane transporter activity | 3/397 | 0.01926059 | 0.09978502 | 0.07772683 | KCNC2/KCNC1/GAL | 3 | BP |
| GO:2000679 | positive regulation of transcription regulatory region DNA binding | 3/397 | 0.01926059 | 0.09978502 | 0.07772683 | HMGA2/HAND2/NEUROD1 | 3 | BP |
| GO:0014033 | neural crest cell differentiation | 5/397 | 0.01948653 | 0.09978502 | 0.07772683 | RET/SNAI2/HAND2/GDNF/PHOX2B | 5 | BP |
| GO:0010587 | miRNA catabolic process | 2/397 | 0.01960629 | 0.09978502 | 0.07772683 | LIN28B/LIN28A | 2 | BP |
| GO:0021548 | pons development | 2/397 | 0.01960629 | 0.09978502 | 0.07772683 | PHOX2A/ASCL1 | 2 | BP |
| GO:0034378 | chylomicron assembly | 2/397 | 0.01960629 | 0.09978502 | 0.07772683 | APOA4/APOC3 | 2 | BP |
| GO:0035768 | endothelial cell chemotaxis to fibroblast growth factor | 2/397 | 0.01960629 | 0.09978502 | 0.07772683 | FGF16/FGF4 | 2 | BP |
| GO:0042178 | xenobiotic catabolic process | 2/397 | 0.01960629 | 0.09978502 | 0.07772683 | UGT1A1/CYP1A1 | 2 | BP |
| GO:0045713 | low-density lipoprotein particle receptor biosynthetic process | 2/397 | 0.01960629 | 0.09978502 | 0.07772683 | FGF21/ITGB3 | 2 | BP |
| GO:0045741 | positive regulation of epidermal growth factor-activated receptor activity | 2/397 | 0.01960629 | 0.09978502 | 0.07772683 | EREG/ADORA1 | 2 | BP |
| GO:0051006 | positive regulation of lipoprotein lipase activity | 2/397 | 0.01960629 | 0.09978502 | 0.07772683 | APOA4/APOA5 | 2 | BP |
| GO:0060346 | bone trabecula formation | 2/397 | 0.01960629 | 0.09978502 | 0.07772683 | GREM1/COL1A1 | 2 | BP |
| GO:0060456 | positive regulation of digestive system process | 2/397 | 0.01960629 | 0.09978502 | 0.07772683 | AQP1/TAC1 | 2 | BP |
| GO:0071281 | cellular response to iron ion | 2/397 | 0.01960629 | 0.09978502 | 0.07772683 | BMP6/TF | 2 | BP |
| GO:0071313 | cellular response to caffeine | 2/397 | 0.01960629 | 0.09978502 | 0.07772683 | CACNA1S/PPARGC1A | 2 | BP |
| GO:0071838 | cell proliferation in bone marrow | 2/397 | 0.01960629 | 0.09978502 | 0.07772683 | HMGA2/PTH | 2 | BP |
| GO:0071863 | regulation of cell proliferation in bone marrow | 2/397 | 0.01960629 | 0.09978502 | 0.07772683 | HMGA2/PTH | 2 | BP |
| GO:0097531 | mast cell migration | 2/397 | 0.01960629 | 0.09978502 | 0.07772683 | CHGA/KIT | 2 | BP |
| GO:0099550 | trans-synaptic signaling, modulating synaptic transmission | 2/397 | 0.01960629 | 0.09978502 | 0.07772683 | SYT4/PLAT | 2 | BP |
| GO:1903800 | positive regulation of production of miRNAs involved in gene silencing by miRNA | 2/397 | 0.01960629 | 0.09978502 | 0.07772683 | MYCN/LIN28A | 2 | BP |
| GO:2000544 | regulation of endothelial cell chemotaxis to fibroblast growth factor | 2/397 | 0.01960629 | 0.09978502 | 0.07772683 | FGF16/FGF4 | 2 | BP |
| GO:0098948 | intrinsic component of postsynaptic specialization membrane | 5/403 | 0.0218327 | 0.09988149 | 0.07807747 | NETO1/CHRNB2/GABRA1/CHRNA9/GRIN2A | 5 | CC |
| GO:0009925 | basal plasma membrane | 3/403 | 0.02191493 | 0.09988149 | 0.07807747 | CLCA2/AQP1/TF | 3 | CC |
